# Bioorthogonal, twice-clickable fluorogenic probes for two-photon live-cell microscopy

**DOI:** 10.1039/d6cb00227g

**Published:** 2026-10-12

**Authors:** Malo Gourvest, Emilie Plantiveau, Aurélie Rodriguez, Emma de Bastiani, Delphine Naud-Martin, Frédéric Bolze, Gilles Clavier, Kévin Renault, Frédéric Taran, Florence Mahuteau-Betzer

**Affiliations:** a CNRS UMR9187, Chemistry and Modeling for the Biology of Cancer Institut Curie, Université PSL 91400 Orsay France florence.mahuteau@curie.fr; b CNRS UMR9187, Chemistry and Modeling for the Biology of Cancer, Université Paris-Saclay 91400 Orsay France; c Inserm U1196, Actuatable Molecules for Diagnosis and Theranostics 91400 Orsay France; d Département Médicaments et Technologies pour la Sante, SCBM, Université Paris Saclay Gif-sur-Yvette 91191 France frederic.taran@cea.fr; e UMR7199 Faculté de Pharmacie 67401 Illkirch-Graffenstaden France; f PPSM ENS Paris-Saclay 91190 Gif-sur-Yvette France

## Abstract

Two-photon-excitable profluorogenic probes capable of undergoing bioorthogonal reactions in complex biological media remain exceedingly rare, despite their tremendous potential for live-cell imaging. We herein describe two new acridane-based probes capable of undergoing double bioorthogonal click reactions with cyclooctynes and exhibiting promising photophysical properties for live-cell imaging.

## Abstract 2

Two-photon-excitable profluorogenic probes capable of undergoing bioorthogonal reactions in complex biological media remain exceedingly rare, despite their tremendous potential for live-cell imaging.

## Graphical abstract

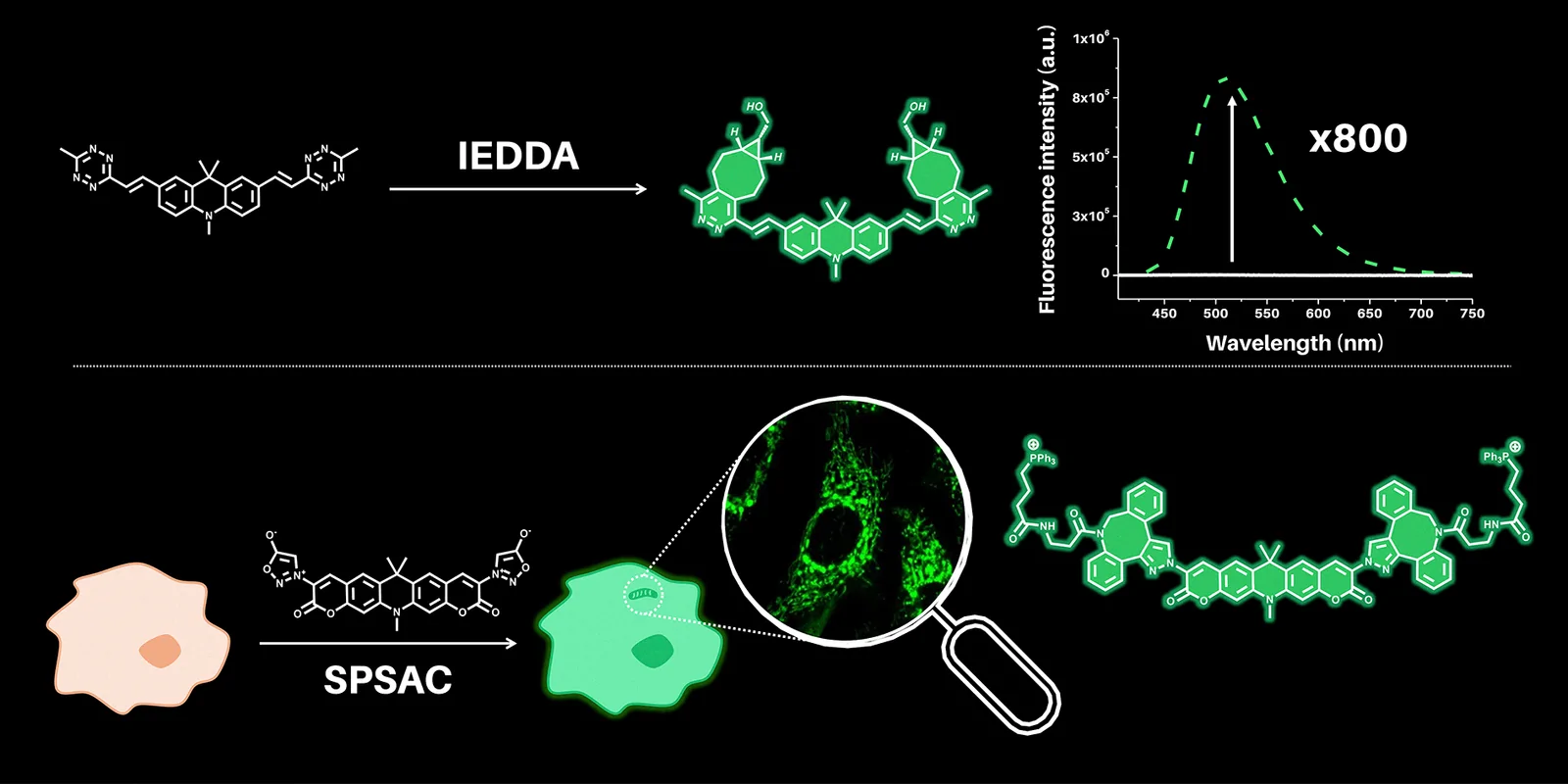

## Introduction

Bioorthogonal fluorogenic probes are efficient tools to understand and visualize cellular events.^1^ Indeed, they enable selective labelling of target molecules (*e.g.*, drugs or biomolecules) in live cells for fluorescence microscopy. Fluorogenic probes ensure a clear distinction between the fluorescence of labelled molecules and that of unreacted profluorophores. Various bioorthogonal reactions, such as SPAAC, iEDDA, or SPSAC, have been successfully employed in this strategy, as functional groups like azides,^2,3^ tetrazines,^4,5^ or sydnones^6^ effectively quench fluorescence until the reaction restores the fluorophore's emission. Fluorescence imaging offers high sensitivity, resolution, and dynamic monitoring capabilities, making it ideal for the development of “turn-on” probes. However, its limitations include poor tissue penetration and potential cell damage. Two-photon excitation (2PE) addresses these challenges by reducing autofluorescence, photodamage, and photobleaching due to its confined excitation volume and lower energy requirements.^7^ However, only a few studies have reported highly two-photon excitable fluorogenic probes, mostly with modest 2PA abilities (two-photon brightness: *B*_2P_ < 30 GM).^8,9^

Recently, we reported high turn-on capabilities of coumarins bearing a sydnone moiety but without any reported 2PA abilities.^6^ On the other hand, we previously described the probe Acri-*o*vi, which combines a strong two-photon absorber (2PA cross section> 3000 GM, *B*_2P_ = 360 GM) with a tetrazine moiety but exhibits quite modest turn-on properties (Scheme 1).^10^ To increase quenching effects, we propose a double-click strategy^11–13^ based on the installation of two quenching moieties on the fluorogenic probe. We herein describe 2PE profluorophores bearing either sydnones or tetrazines. As for Acri-*o*vi, we adopted a quadrupolar design (D–π–A–π–D or A–π–D–π–A) as these structures are predicted to possess high 2PE capabilities^14,15^ and selected the acridane core as we demonstrated that it drastically enhances the 2PE properties of fluorophores. We explored two fluorogenic strategies: quenching a pre-existing fluorophore through click handles^4–6,10^ and *in situ* formation of a fluorophore.^8^ In the first approach, sydnones are designed to quench a quadrupolar A–π–D–π–A fluorophore, comprising two coumarin units linked to a central acridane core. In the second, we propose conjugating the acridane core to two tetrazine moieties through a π-conjugated system. Upon undergoing the bioorthogonal reaction, the resulting pyridazines would serve as electron acceptors, enabling the *in situ* generation of a quadrupolar A–π–D–π–A fluorochrome. Here, we designed and synthesized two novel fluorogenic probes, assessed their reactivity for cycloaddition reactions, determined their photophysical properties under one- and two-photon excitation, analyzed them *via* TD-DFT calculations, and evaluated the most promising candidate for cellular fluorescence microscopy.

**Scheme 1:**
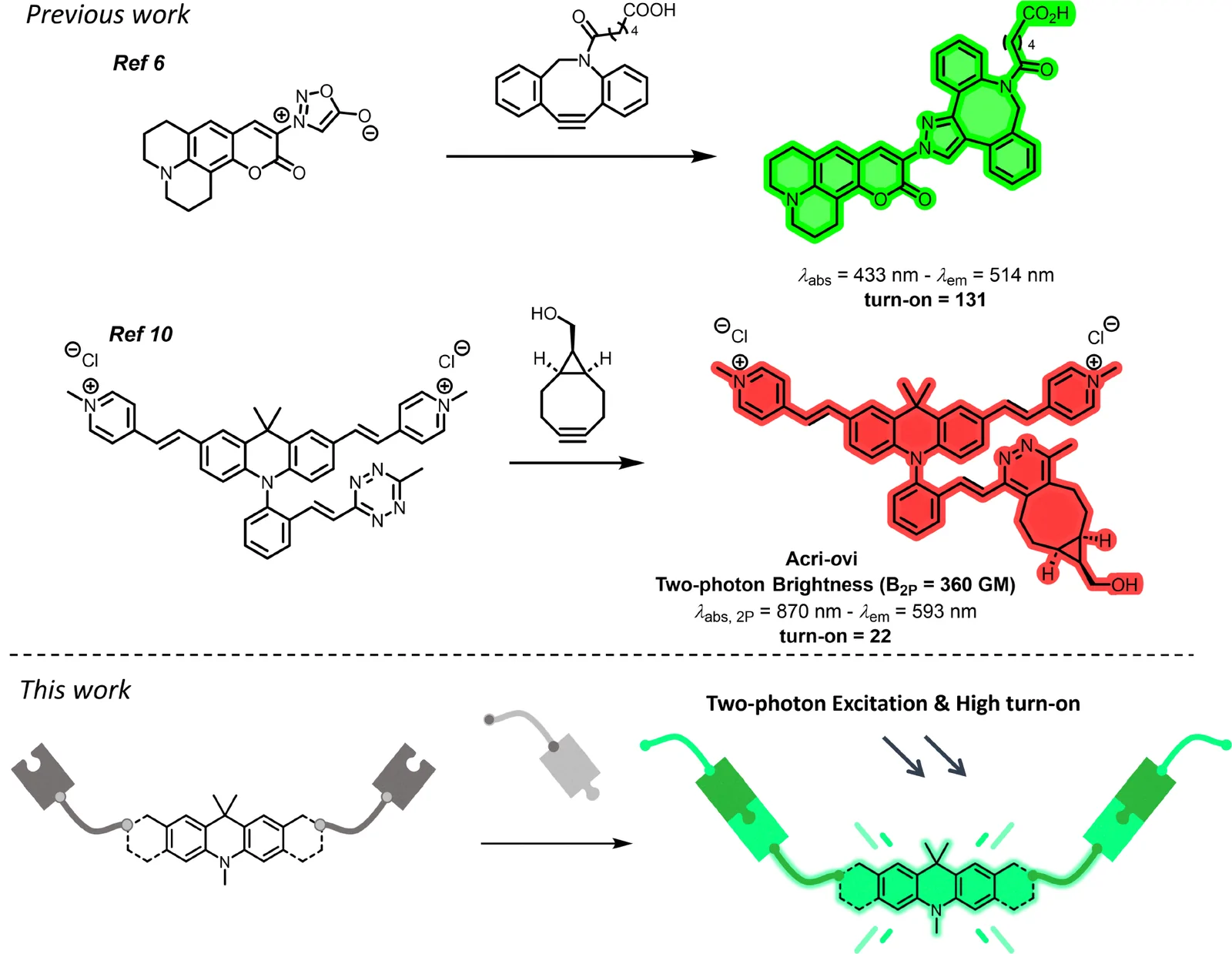
Design of two-photon excitable fluorogenic probes.

## Synthesis

The probe bearing two clickable vinyl-tetrazine moieties (TzProbe) was synthesized following a strategy inspired by the work of Wu and co-workers,^8^ which also incorporates a vinyl-tetrazine motif. Building block 1 was first prepared in 50% yield according to a previously reported procedure,^16^ while compound 2 was obtained in 59% yield using a synthetic route previously developed in our laboratory.^17^ Finally, a Horner–Wadsworth–Emmons reaction between these two intermediates afforded TzProbe in 24% yield.

The doubly clickable probe incorporating an acridane core, two coumarin units, and two sydnone moieties (SydProbe) was synthesized in 11 steps using compounds 5 and 9 as key building blocks. Sydnone 5 was prepared on a gram scale through optimization of previously reported procedures, affording an improved overall yield of 45%. In particular, the nitrosylation step leading to compound 3 was significantly enhanced by implementing a Soxhlet extraction protocol, which enabled more efficient product recovery and increased the isolated yield from 28% to 88% (see the SI).

Compound 9 was synthesized from intermediate 6, which had previously been optimized in the literature and was obtained in 32% overall yield over four steps.^18^ Subsequent *N*-methylation of the acridane core, followed by a Vilsmeier–Haack formylation and final demethylation using BBr_3_, afforded the key intermediate 9 in 44% overall yield over three steps, corresponding to an overall yield of 14% over seven steps.

SydProbe was subsequently obtained through a tandem Knoevenagel condensation/transesterification sequence between building block 9 and three equivalents of 5. Under the optimized reaction conditions, SydProbe was produced in near-quantitative yield and could be isolated by simple filtration (Scheme 2).

**Scheme 2:**
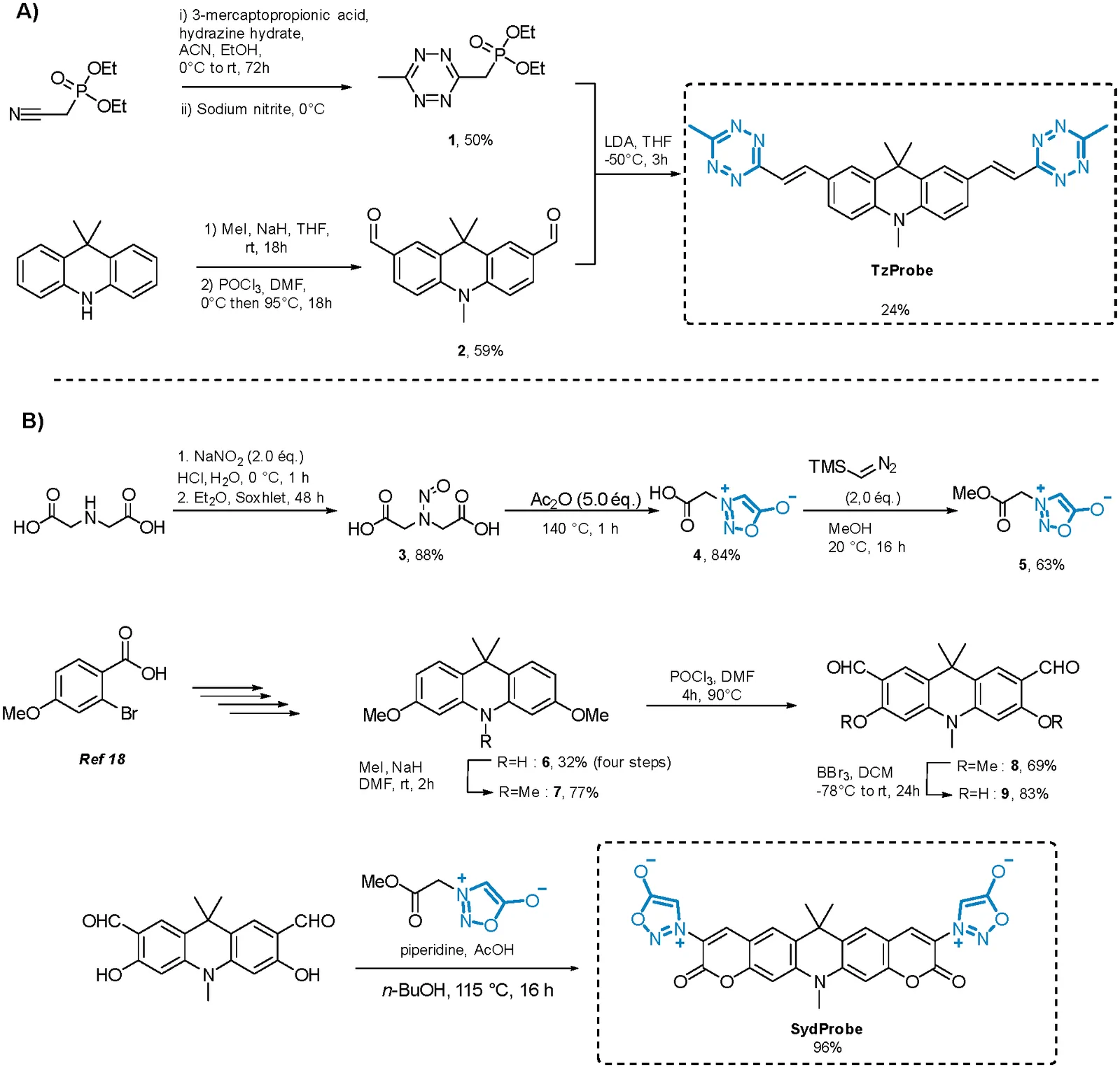
Synthesis of fluorogenic (A) TzProbe and (B) SydProbe.

As the probes contain two clickable moieties, we synthesized and isolated the mono-Click cycloadducts and the bi-Click cycloadducts (Scheme 3). Using a substoichiometric amount of cycloalkynes (0.5 equiv. for BCN and 0.7 equiv. for DBCO-acid), TzMonoClick and SydMonoClick were obtained, respectively, using iEDDA and SPSAC with 48 and 39% yields. In contrast, TzDiClick and SydDiClick were synthesized using an excess of the cycloalkynes (Scheme 3).

**Scheme 3:**
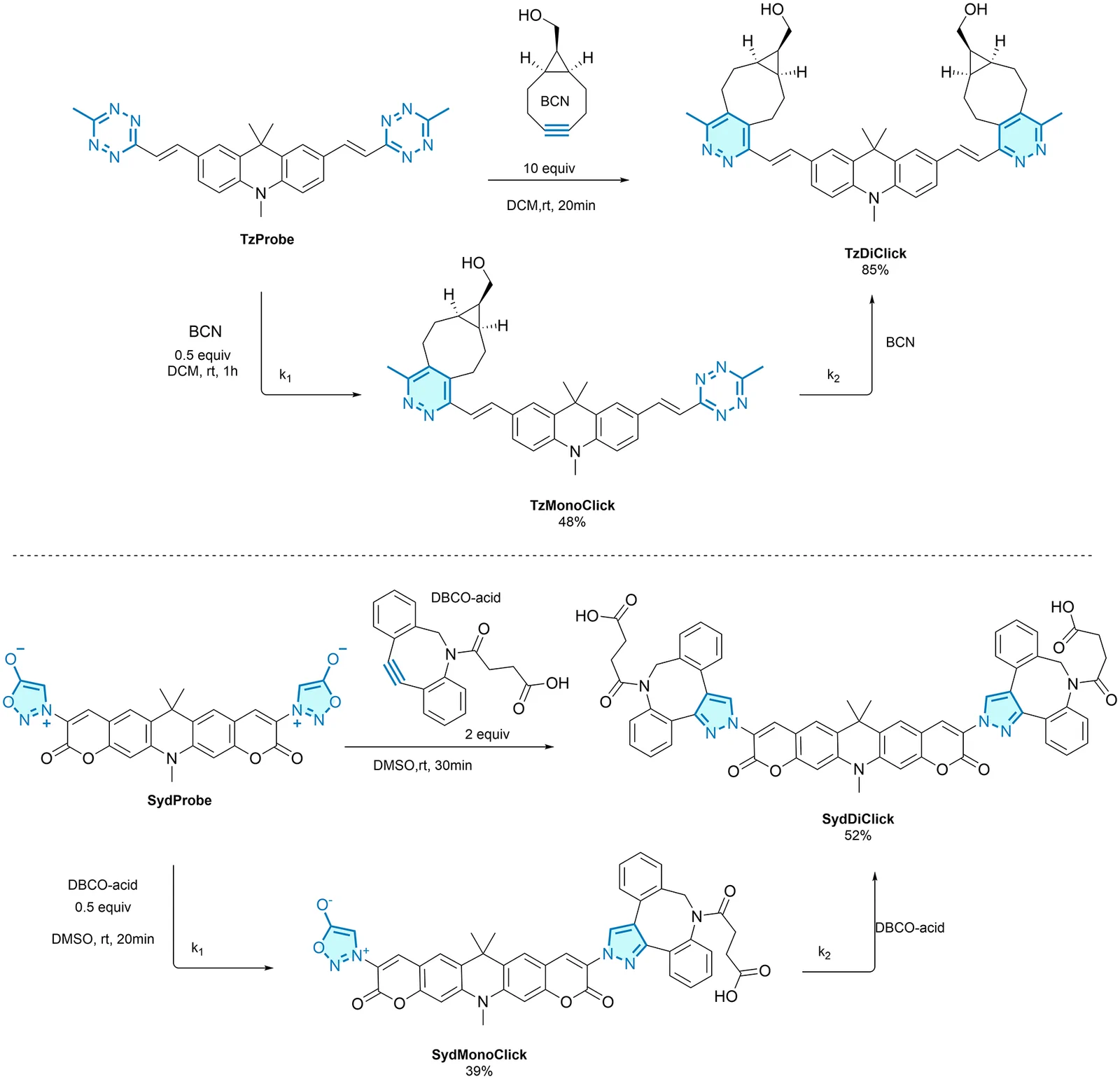
Synthesis of click products.

## Linear optical properties

The photophysical properties of the probes and their mono- and di-Click products were studied at room temperature in an organic solvent (DMSO) and in a viscous solvent mimicking the cellular environment (PBS containing 100 equivalents of BSA). All data are summarized in Table 1. Except for TzProbe, these dyes obeyed Beer–Lambert's law up to 20 µM in PBS/BSA (see Absorption Spectra in the SI), indicating that they are water soluble up to this concentration. TzProbe is less soluble in buffer and can be used only below 2 µM.

**Table 1:** Photophysical properties of TzProbe, TzMonoClick, TzDiClick, SydProbe, SydMonoClick, SydDiClick

Compounds	Solvents	*λ*_abs_/*λ*_em_ [nm]	*ε* [M^−1^ cm^−1^]	*Φ*_F_	*B* = *ε* × *Φ*_F_ [M^−1^ cm^−1^]
TzProbe	DMSO	436/—	38 400	NA	NA
	PBS/BSA	429/—	13 700	NA	NA

TzMonoClick	DMSO	420/510	34 600	0.01	346
	PBS/BSA^a^	420/492	24 700	0.005	NA

TzDiClick	DMSO	403/502	35 500	0.32	11 360
	PBS/BSA^a^	408/488	24 200	0.03	726

SydProbe	DMSO	444/592	41 400	0.22	9110
	PBS/BSA^a^	457/539	14 900	0.04	596

SydMonoClick	DMSO	446/535	32 400	0.02	648
	PBS/BSA^a^	453/497	27 140	0.09	2442

SydDiClick	DMSO	438/526	33 100	0.40	13 240
	PBS/BSA^a^	445/508	31 000	0.24	7440

a With 100 equiv. of BSA; *Φ*_F_ = quantum yield; *B* = brightness; NA = non applicable.

In DMSO, the maximum absorption wavelength of both probes ranged from 403 nm to 444 nm with a high molar absorption coefficient (from 33 100 M^−1^ cm^−1^ for SydDiClick to 41 400 M^−1^ cm^−1^ for SydProbe). In PBS/BSA, their molar extinction coefficients were marginally reduced, ranging from 24 200 for TzDiClick to 31 000 M^−1^ cm^−1^ for SydDiClick, except for TzProbe and SydProbe, which exhibited a pronounced decrease. This behavior is likely due to solubility issues for TzProbe and aggregation of SydProbe, as evidenced by the excitation spectrum (see the SI). As expected, TzProbe was not emissive either in DMSO or in PBS/BSA. Conversely, TzMonoClick exhibited weak fluorescence in DMSO, with a quantum yield of 1%, indicating that pyridazine formation resulted in a weakly emissive dipolar fluorophore. In contrast, the quadrupolar fluorophore TzDiClick emitted in the green region with a significantly higher fluorescence quantum yield of 40% in DMSO. A similar trend was observed in PBS/BSA: TzMonoClick remained poorly fluorescent, while TzDiClick exhibited a quantum yield of 3%. However, TzDiClick exhibited a slight bathochromic shift in emission in PBS/BSA compared to DMSO along with lower brightness. Since TzProbe is non-fluorescent in PBS/BSA, a high turn-on effect can be expected in cellular medium.

In contrast, SydProbe emitted in the yellow region (*λ*_max_ = 592 nm) in DMSO with a high fluorescence quantum yield, resulting in high brightness. Conversely, SydMonoClick displayed poor fluorescence, leading to a substantial decrease in brightness compared to SydProbe while the diclicked adduct showed a high fluorescence quantum yield (40%). A significant blue shift (up to 66 nm) between SydDiClick and SydProbe was observed in their emission, conferring a ratiometric behavior of this probe upon the SPSAC reaction in DMSO. In PBS/BSA, a blue shift was also observed between the click adducts and the probe (up to 42 nm between SydDiClick, and SydProbe), but it was less pronounced. Most notably, the fluorescence quantum yields increased substantially (4%, 9%, and 24% for SydProbe, SydMonoClick, and SydDiClick, respectively), leading to a significant enhancement in the brightness of the adducts.

## Nonlinear optical properties

The high fluorescence quantum yields of SydProbe, SydDiClick and TzDiClick allowed the determination of the two-photon absorption (2PA) cross sections in DMSO by the fluorescence method (Fig. 1).

**Fig. 1:**
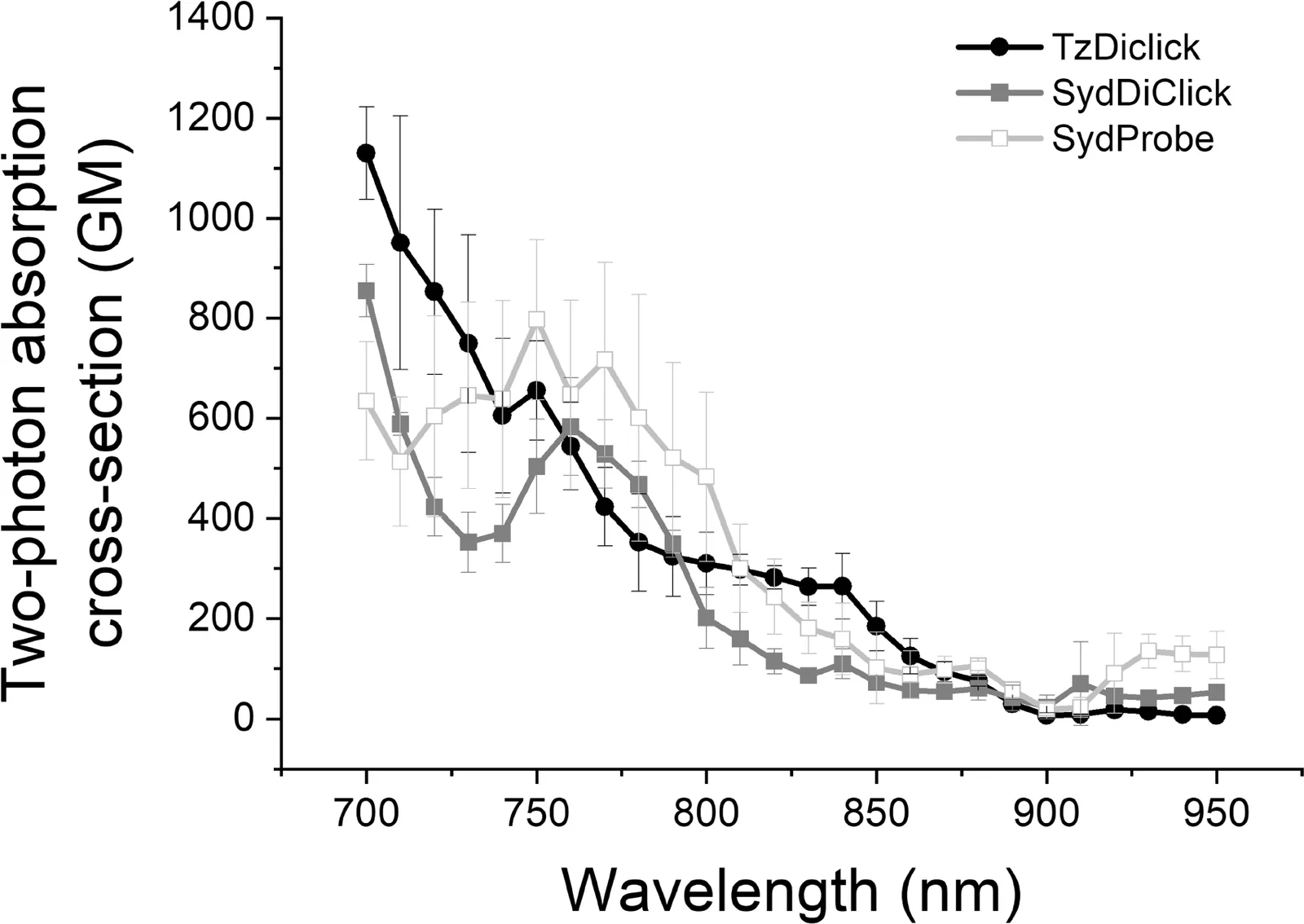
Two-photon excitation spectra of SydProbe, SydDiClick and TzDiClick in DMSO.

All compounds present high 2PA cross-sections in the 700–950 nm range. The maximum of the 2PA peak for Tz-DiClick is not in the study window (<700 nm) but it remains important in the 700–750 nm range (655 to 1131 GM), leading to a brightness of 362 GM at 700 nm. The use of tetrazine as a quencher induces an electron withdrawing effect on the periphery of the quadrupolar chromophore leading to an A–π–D–π–A architecture. This electronic effect is less important than other end groups such as pyridiniums,^19^ inducing a blue shift of the 2PA band, and a lower 2PA cross section but leading to a similar two-photon brightness thanks to higher fluorescence quantum yields (362 GM for TzDiClick and 330 GM for AcriPy^19^). For SydProbe, acting as a ratiometric probe, the 2PA cross sections are lower for the diclicked chromophore compared to the parent probe (797 GM for the unclicked SydProbe and 583 GM for the bis-clicked SydDiClick) with a similar absorption maximum in the 740–780 nm zone. The high two-photon brightness of these two molecules opens the way for two-photon excitation cellular imaging (Table 2).

**Table 2:** Nonlinear photophysical properties of SydProbe, SydDiClick and TzDiClick in DMSO

Compounds	*λ*_abs_ [nm]	*δ* [GM]	*Φ*_F_	*B*_2P_ = *δ* × *Φ*_F_ [GM]
SydProbe	750	797	0.22	175

SydDiClick	700	854	0.40	342
	760	583	0.40	240

TzDiClick	700	1131	0.32	362

## Molecular modelling

In order to gain deeper insight into the photophysical properties of both probe series and their corresponding clicked analogues, a comprehensive computational study was carried out. Time-dependent density functional theory (TD-DFT) calculations were performed at the M06-2X/6-311+G(d,p) level of theory, including water as the solvent through a continuum model (IEFPCM).

For TzProbe, the calculated absorption spectrum revealed that the dominant electronic transition corresponds to S_0_ → S_3_, with a high oscillator strength (*f* = 1.4062), whereas the lower-energy transitions S_0_ → S_1_ and S_0_ → S_2_ exhibit very weak oscillator strengths (*f* = 0.0008 and 0.0087, respectively). Such a distribution of oscillator strengths suggests that the lowest excited states are essentially dark states, providing a first explanation for the absence of fluorescence observed experimentally.

To further investigate the nature of these electronic transitions, natural transition orbital (NTO) analyses were performed. In systems where the lowest-energy excited states cannot be adequately described by a simple HOMO → LUMO transition, NTOs provide a more reliable representation of the excitation by combining all significant orbitals (Fig. 2).

**Fig. 2:**
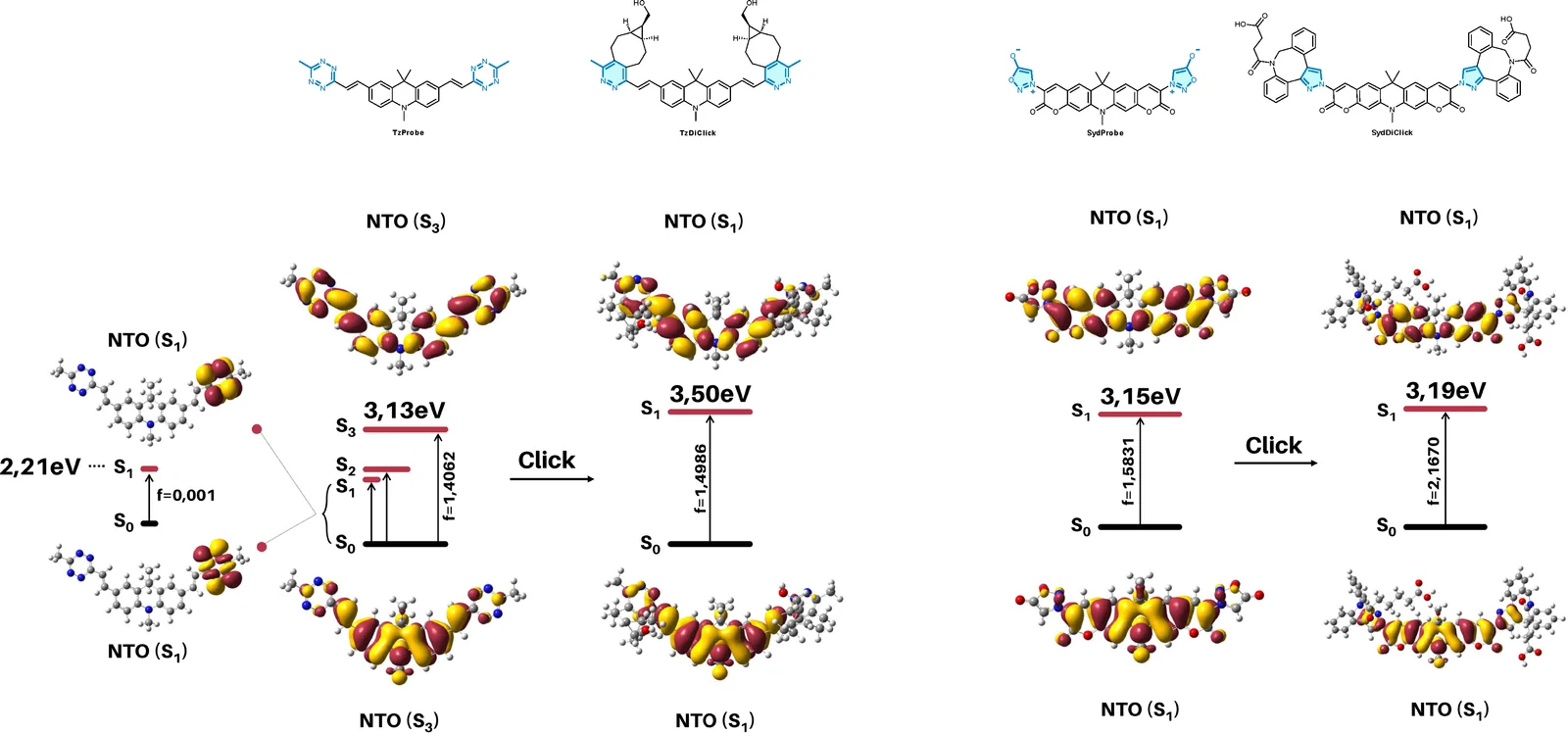
Comparison of the natural transition orbitals (NTOs) of the probe series and their clicked analogues, illustrating changes in electronic delocalization and charge-transfer character.

Analysis of the NTOs associated with the S_0_ → S_1_ transition of TzProbe (and S_0_ → S_2_, see the SI) shows that both hole and particle densities remain essentially localized on the tetrazine moieties and correspond to their low-energy n–π* transition. Such a highly localized excitation indicates a lack of effective electronic delocalization across the molecular framework, which is generally unfavorable for radiative decay and fluorescence emission. The S_0_ → S_3_ transition corresponds to a π–π* intramolecular charge transfer transition (ICT) with the electronic density moving from the central electron-rich acridane towards the external electron-deficient vinyl-tetrazines. These computational results are therefore fully consistent with the experimental observations, where TzProbe exhibits no detectable fluorescence.

In contrast, after the click reaction, the resulting TzDiClick derivative displays NTOs that are delocalized over the entire molecular scaffold. This extended electronic distribution is the characteristic of an intramolecular charge-transfer (push–pull) system and supports the restoration of fluorescence observed experimentally. However, the pyridazine is a weaker electron acceptor than the tetrazine and the corresponding absorption band is located at higher energy (3.5 eV *vs.* 3.13 eV for TzDiClick).

For the SydProbe series, the NTOs of the probe and its clicked analogue are relatively similar, which is consistent with the experimental results since both compounds are fluorescent. In both cases, the low energy transition corresponds to an ICT one. The difference in their energy reflects the stronger electron acceptor character of the sydnone compared to the pyrazole. This difference can be estimated through the overlap between the hole and electron distribution functions (Sr), which is smaller for SydProbe than for SydDiClick (0.58664 and 0.61915 a.u., respectively). In other words, the electron density is more separated from the hole in the case of SydProbe, reflecting a stronger CT character of the transition.

We attempted to rationalize the pronounced solvatochromic behavior of SydProbe, whose maximum emission shifts from 592 nm in DMSO to 614 nm in PBS and 539 nm in BSA-containing medium. To this end, excited-state geometry optimizations were performed in both water and DMSO using TD-DFT calculations. The implicit model used did not reveal any significant structural differences between the optimized excited-state geometries in the two solvents. This is probably the sign that specific solvent interactions, particularly hydrogen-bonding in water, could stabilize different excited-state conformations relative to DMSO.

## Turn-on and kinetics

To determine turn-on ratios, we monitored the fluorescence during the reaction between the probes (2 µM) and 10 equivalents of cycloalkynes (BCN for the Tz-series and DBCO for the Syd-series) in DMSO and in PBS containing 100 equivalents of BSA (Fig. 3 and the SI). We also tracked fluorescence during the click reaction between MonoClick adducts and cycloalkynes under the same conditions. We reported the turn-on ratios as the ratio of the fluorescence signal of the clicked compounds to that of the non-clicked compounds. Unless explicitly stated otherwise, the fluorescence signal was measured by integrating the emission spectra between 425 and 700 nm.

**Fig. 3:**
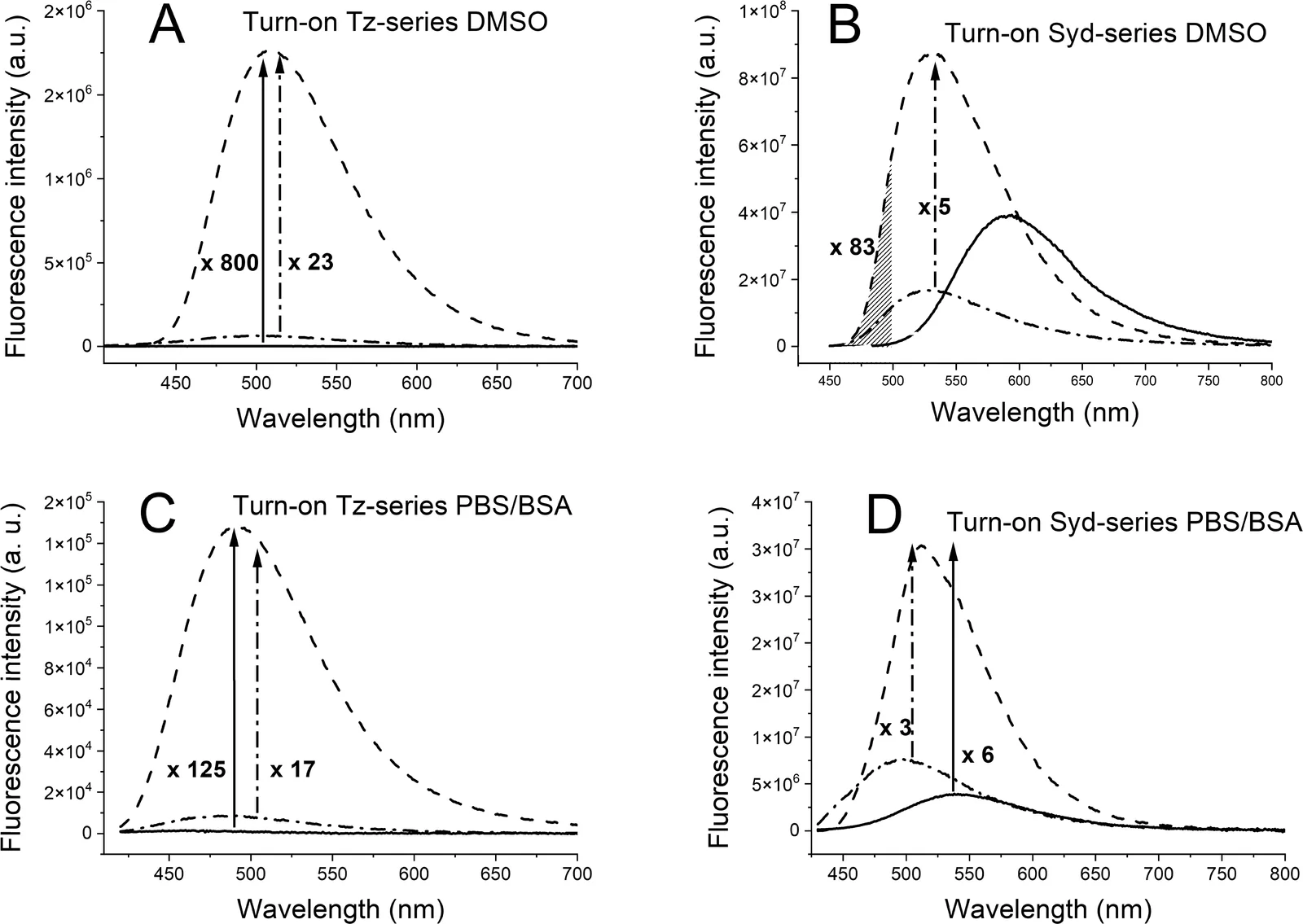
Emission spectra of the probes (plain line), the monoclick products (dashed-dotted line) and the diclick products (dotted line) at 20 µM in DMSO (A) and (B) or PBS/BSA (C) and (D), respectively, for at 2 µM for Tz-series and at 10 µM for Syd-series. All click products were obtained using 250 equivalents of BCN.

The observed turn-on ratios aligned with the ratios of the measured fluorescence quantum yields of the synthesized adducts (see Table 1), maintaining the same ranking. Turn-on ratios were higher for the Tz-series than for the Syd-series, demonstrating the efficiency of the strategy based on *in situ* fluorophore generation. Since TzProbe is non-emissive, impressive turn-on ratios were measured upon the iEDDA reaction in DMSO and PBS/BSA (800 and 125, respectively). Notably, even the iEDDA reaction of TzMonoClick with BCN resulted in high turn-on ratios of approximately 20 in both media.

Due to the blue-shifted emission between SydProbe and SydDiClick, we measured a very high turn-on ratio of 83 when integrating fluorescence area between 450 nm and 500 nm. Although the turn-on effects in the Syd-series in PBS/BSA were lower than those in the Tz-series, they remained sufficient to enable monitoring of the bioorthogonal reaction by cellular fluorescence microscopy.

The reaction mechanism of these bifunctional probes consists of two successive intermolecular steps, each governed by second-order rate constants. The determination of such rate constants has previously been demonstrated by Keillor *et al.*^11^ for the sequential thiol addition to a bismaleimide. We first monitored the reaction between MonoClick compounds and cycloalkynes by fluorescence in DMSO (SydMonoClick with DBCO and TzMonoClick and BCN), using a large excess of alkyne enabling the determination of reaction constants *k*_2_ (see Kinetics in the SI). We then fit the increase of fluorescence of the reactions between the probes and cycloalkyne to calculate the rate constant of the first step *k*_1_. For both the double-iEDDA and double-SPSAC reactions, the two successive click events proceeded with second-order rate constants on the order of 1 M^−1^ s^−1^ in DMSO (see Table 3), values that are compatible with applications in living cells. Notably, the rate constants for the first step were higher than those for the second step in both reaction types. In PBS/BSA buffer, the kinetic curves for the Syd-series and for the conversion of TzProbe to TzDiClick could not be fitted to determine the corresponding rate constants. This is probably due to solubility issues or aggregation in PBS/BSA (see absorption spectra in the SI). In contrast, the kinetic curve for the conversion of TzMonoClick to TzDiClick was successfully fitted, yielding a rate constant *k*_2_ of 17.5 M^−1^ s^−1^. This value is 14 times greater than that observed in DMSO, indicating that the click reaction proceeds more rapidly in buffer. Similarly, the conversion of SydProbe to SydDiClick, as well as that of TzProbe to TzDiClick, is accelerated four-fold in PBS/BSA compared to DMSO. Indeed, the curve reached the plateau four times faster at the same concentration in PBS/BSA than in DMSO for both conversions (see the SI). These findings are promising for their application in cellular environments.

**Table 3:** Rate constants measured for the consecutive click reaction of cycloalkynes, BCN and DBCO, respectively, with fluorogenic probes TzProbe and SydProbe

Compounds	Solvents	*k*_1_ [M^−1^ s^−1^]	*k*_2_ [M^−1^ s^−1^]
TzProbe	DMSO	2.69 ± 0.22	1.24 ± 0.03
	PBS/BSA	NA	17.5 ± 1.75

SydProbe	DMSO	1.10 ± 0.11	0.74 ± 0.01
	PBS/BSA	NA	NA

## Fluorescence microscopy

We investigated TzProbe and SydProbe for live-cell imaging in the A549 cell line (lung carcinoma epithelial cells). However, likely due to solubility issues, TzProbe proved unsuitable for cellular imaging experiments. Consequently, we focused our efforts on SydProbe. Due to its residual—albeit very weak—fluorescence, we observed that SydProbe was evenly distributed throughout the cytoplasm (see Fig. 4B and E, and Fig. S5C). This homogeneous distribution, combined with its strong fluorogenicity, prompted us to evaluate SydProbe for selective imaging of subcellular compartments. Thus, cells were incubated with SydProbe (2 µM) in the presence of a DBCO derivative modified with a triphenylphosphonium moiety (DBCO-TPP, 50 µM), which is known to target mitochondria.

**Fig. 4:**
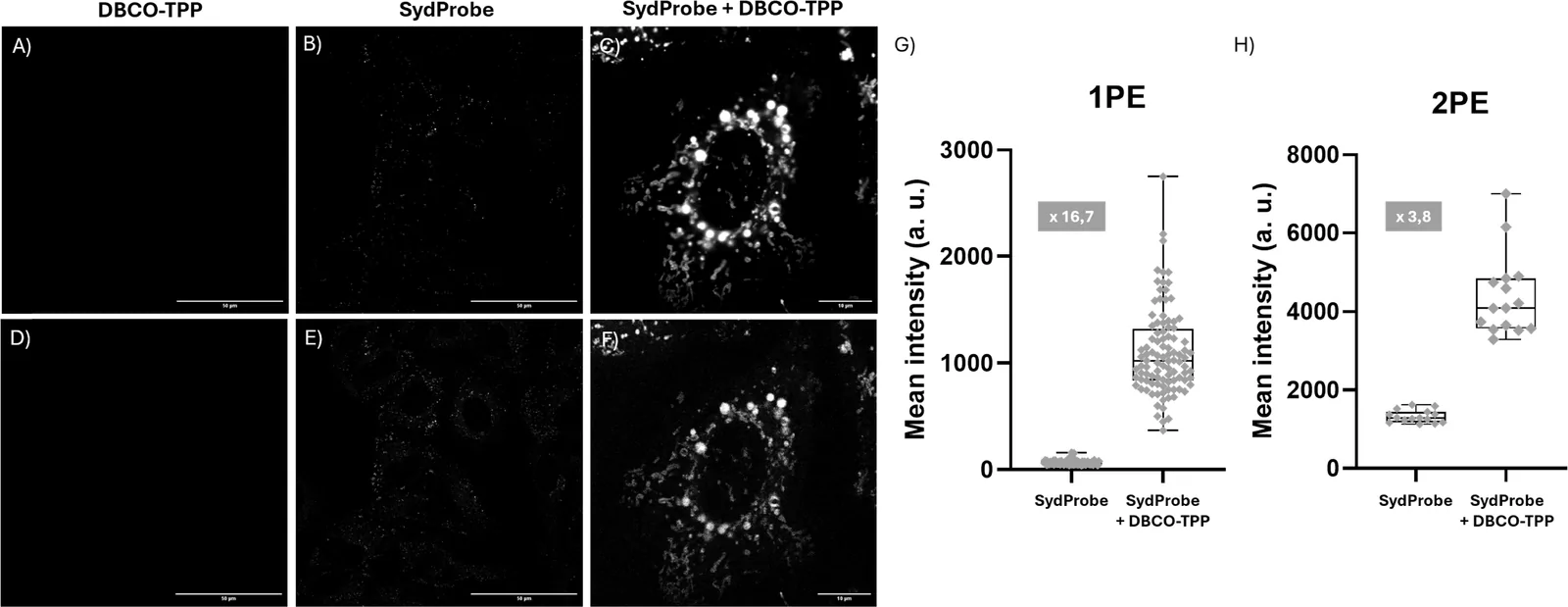
Fluorescence emission from SydProbe with and without DBCO-TPP and control experiments with one (445 nm) and two-photon excitation (760 nm) in live A549 cells. (A) and (D) A549 cells were incubated with DBCO-TPP (50 µM) for 30 min. (B) and (E) A549 cells were incubated with SydProbe (2 µM) for 24 hours. (C) and (D) A549 cells were incubated with DBCO-TPP (50 µM) for 30 min and then with SydProbe (2 µM) for 24 hours. Images (A), (B) and (C) were collected under mono-photonic excitation (*λ*_ex_ = 445 nm, *λ*_em_ = 460–600 nm, laser = 2%, gain = 600 V) and images (D), (E) and (F) were collected under two-photon excitation (*λ*_ex_ = 760 nm, *λ*_em_= 460–700 nm, laser = 0.5%, gain = 800 V). (G) and (H) Fluorescence quantification in live A549 cells with SydProbe (2 µM) with and without DBCO-TPP (50 µM). A box plot represents the fluorescence intensity obtained by measuring pixel means using ImageJ software under one (1PE) (G; *n* = 100) or two-photon excitation (2PE) (H; *n* = 15). The settings and data processing were identical for all images from the 1PE and 2PE series, independently of one another.

Following this experiment, we observed an intense fluorescence signal within the mitochondria. The specificity of the mitochondrial signal was verified through a colocalization experiment using the commercial dye MitoTracker Deep Red FM. Colocalization of the SydProbe + DBCO-TPP signal with MitoTracker Deep Red FM was confirmed by Pearson's correlation coefficient of 0.74 (see Fig. 5 and Fig. S1). Additionally, control experiments demonstrated no overlap between the fluorescence signals in the respective channels of SydProbe + DBCO-TPP and MitoTracker Deep Red FM, further validating the mitochondrial colocalization (see Fig. S2 and S3).

**Fig. 5:**
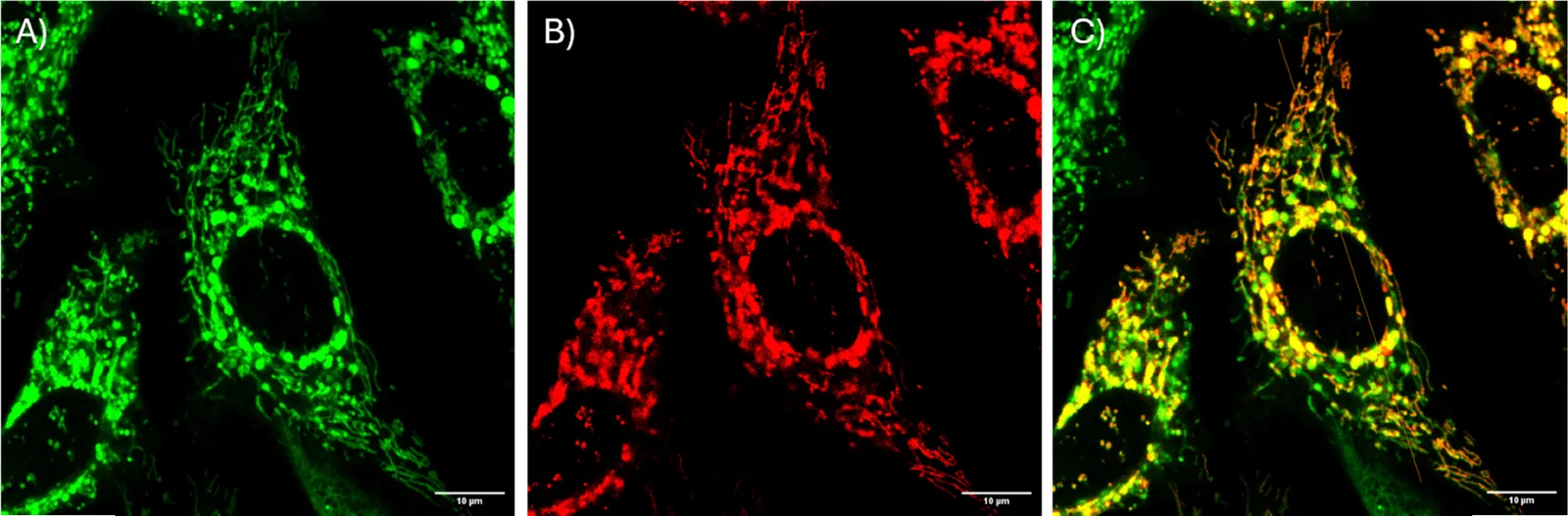
Fluorescence emission from SydProbe + DBCO-TPP colocalized to mitochondria in live A549 cells. (A) A549 cells were incubated with DBCO-TPP (50 µM) for 30 min and then with SydProbe (2 µM) for 24 hours (*λ*_ex_ = 442 nm, *λ*_em_ = 460–600 nm. (B) A549 cells were incubated with Mito-Tracker Deep-Red FM (20 nM) for 1 hour (*λ*_ex_ = 635 nm and *λ*_em_ = 650–750 nm). (C) Merge image.

The fluorescence spectrum of SydProbe + DBCO-TPP (see Fig. S4) showed an emission maximum at approximately 500 nm, consistent with the *λ*_max_ emission of SydDiClick in PBS/BSA. To confirm that the fluorescence signal detected in mitochondria resulted from the double SPAAC reaction between SydProbe and DBCO-TPP, control experiments were performed. Cells incubated with SydProbe alone (2 µM) exhibited only residual fluorescence, while no signal was detected for cells treated with DBCO-TPP alone (50 µM) or the vehicle (DMSO) (see Fig. S5). To quantify the fluorescence enhancement resulting from the in-cell click reaction, we compared the fluorescence signals from 100 cells incubated with that from SydProbe alone (2 µM) or with SydProbe (2 µM) + DBCO-TPP (50 µM) (see Fig. 4G). This comparison revealed a 16.7-fold turn-on, demonstrating that SydProbe is a powerful tool for cellular imaging.

Given that both SydProbe and SydDiClick exhibit strong two-photon absorption, we exploited this property to perform two-photon excitation microscopy. In the presence of SydProbe (2 µM) and DBCO-TPP (50 µM), an intense fluorescence signal was observed following two-photon excitation at 760 nm (Fig. 4F).

To confirm that the signal detected by 2PE originated from the SPAAC reaction between SydProbe and DBCO-TPP, we conducted several control experiments. First, the colocalization of the 1PE and 2PE signals was confirmed by Pearson's correlation coefficient of 0.76 (see Fig. S6). Second, cells incubated with DBCO-TPP alone (50 µM) showed no detectable signals (Fig. 4D). Finally, cells incubated with SydProbe alone (2 µM) exhibited a more pronounced residual fluorescence signal compared to the 1PE experiments (Fig. 4E). This trend was further supported by fluorescence enhancement measurements in cells incubated with SydProbe (2 µM), with or without DBCO-TPP (50 µM), yielding a turn-on value of 3.8 (see Fig. 4H). The discrepancy observed between the 1PE (16.7-fold) and 2PE turn-on (3.8-fold) is likely due to differences in the probes' photophysical properties. Specifically, while the two-photon cross-section of SydProbe is slightly higher than that of SydDiClick, its molar extinction coefficient is significantly lower.

## Conclusions

In conclusion, we have developed two novel acridane-based fluorogenic probes relying on a double-click strategy to enhance fluorescence activation upon bioorthogonal ligation. The tetrazine-based probe exhibited remarkable fluorogenicity through *in situ* fluorophore generation, achieving turn-on ratios of up to 800, whereas the sydnone-based probe combined a ratiometric response with excellent one- and two-photon photophysical properties. Both probe families displayed high two-photon absorption cross sections and bioorthogonal reaction kinetics compatible with live-cell applications. TD-DFT calculations provided a molecular understanding of their distinct fluorescence mechanisms, highlighting the role of the excited-state electronic structure in controlling emission. Finally, the sydnone probe enabled selective mitochondrial imaging in living cells following double SPSAC ligation with the cyclooctyne DBCO under both one- and two-photon excitation. These results demonstrate that the double-click strategy constitutes an effective approach to improve fluorogenic probe performance and opens new opportunities for the development of bright two-photon bioorthogonal probes for deep-tissue and high-resolution fluorescence imaging.

## Author contributions

FMB and FT: conceptualisation, funding acquisition, supervision, writing – original draft, and writing – review & editing. KR: conceptualisation, supervision, investigation, and writing – review & editing. EP, AR, EdB, and DNM: investigation. MG: writing – original draft and investigation. GC and FB: methodology, supervision, and writing – review & editing.

## Conflicts of interest

There are no conflicts to declare.

## Supplementary Material

CB-OLF-D6CB00227G-s001

## Data Availability

The data supporting this article have been included as part of the supplementary information (SI). Supplementary information is available. See DOI: https://doi.org/10.1039/d6cb00227g.
