# Supplementary material for: Bioorthogonal, twice-clickable fluorogenic probes for two-photon live-cell microscopy
Source: RSC Chem Biol. 2026 Oct 12. Online ahead of print. doi: 10.1039/d6cb00227g (PMC13681382; doi:10.1039/d6cb00227g)
Supplement: Supplementary file 1 [file CB-OLF-D6CB00227G-s001.pdf]

Supplementary Information (SI) for RSC Chemical Biology.
This journal is © The Royal Society of Chemistry

Bioorthogonal, Twice-Clickable Fluorogenic Probes for Two-Photon Live-Cell Microscopy

Malo Gourvest, Emilie Plantiveau, Aurélie Rodriguez, Emma de Bastiani, Delphine Naud-Martin, Frédéric Bolze, Gilles Clavier, Kévin Renault, Frédéric Taran and Florence Mahuteau-Betzer

I) Experimental procedures: 2
II) Synthesis procedure: 4
III) Characterization of new compounds: 14
IV) Photophysical properties: 36
V) Kinetics: 49
VI) Molecular modeling: 55
VII) Fluorescence microscopy: 69
VIII) References: 75

I) Experimental procedures:

a) General

Reagents were purchased as reagent-grade and used without further purification unless otherwise stated. THF, DMF and DCM were dried over alumina cartridges immediately prior to use, using a solvent purification system MBraun SPS-5. Analytical thin-layer chromatographies (TLC) were carried out on Macherey Nagel Alugram Xtra SIL G/UV254 plates and on silica 0,25 mm (60 F254, Merck).

The spots were directly visualized or through illumination with UV lamp (λ = 254/365 nm). For the staining of **SydProbe**, the TLC plates were dipped into a solution of basic aqueous permanganate (1g $KMnO_4$, 6g K_2CO_3 and 0.1g KOH in 100 mL H_2O) and developed with a heat gun. Flash chromatographies were carried out on Puriflash system from Interchim equipped with packed silica cartridges from Macherey Nagel. Yields refer to chromatographically and spectroscopically pure compounds. For the synthesis of SydMonoClick and SydDiClick, purifications on reversed phase chromatography were done with a Puriflash® XS520Plus (Columns: BIOTAGE® SNAP Cartridge KP-C18-SH-12g).

b) Instruments and methods

Synthesis. NMR spectra were recorded on a Bruker AV300 spectrometer at room temperature and on a Bruker Avance 400 MHz spectrometer. Chemical shifts (δ), which are expressed in part per million (ppm), were determined relative to residual non- deuterated solvent as an internal reference respectively $CDCl_3$, CD_2Cl_2, CD_3OD and $(CD_3)_2SO$ (^{13}C NMR: δ = 77.16, 53.84, 49.00 and 39.52 ppm; ^{1}H NMR: δ = 7.26, 5.32, 3.31 and 2.50 ppm).[1] Coupling constant(s) in hertz (Hz) were measured from one-dimensional spectra and multiplicities were abbreviated as following: s (singlet), d (doublet), t (triplet), q (quadruplet), m (multiplet). For the synthesis of **TzProbe**, **TzMonoClick** and **TzdiClick**, LC-MS spectra (ESI in the positive ion mode) were recorded on a Waters Acquity Arc instrument coupled to a Waters Acquity QDa and a Waters PDA 2998, Analytical system: RP-HPLC (Luna Omega C18 - 3 μm 100 Å column, 3.0 ×50 mm) with MeCN (+0.1% FA) and 0.1% aq. formic acid (aq. FA, pH 2.5) as eluents [5% MeCN (1 min) followed by linear gradient from 5% to 100% (5 min) MeCN, then 100% MeCN (2 min)] at a flow rate of 0.5 mL/min. UV-visible detection was achieved at full scan in the range 200-600 nm. Low resolution ESI-MS detection in the positive/negative mode (full scan, 100-1250 a.m.u., peaking format: continuum, needle voltage: 0.8 kV, probe temperature: 600 °C, cone voltage: 20 V and scan time: 0.2 s). For the synthesis of **SydProbe**, **SydMonoClick** and **SydDiClick**, LC-MS spectra were recorded on a Waters Acquity UPLC® equipped PDA eλ Detector and SQ Detector 2, mobile phase A: H_2O + 0.1 % formic acid, mobile phase B: acetonitrile + 0.1 % formic acid.

HRMS (High resolution mass spectra) were measured with a TOF mass spectrometer, using electrospray ionization (ES) or atmospheric pressure photoionization method (APPI).

c) Linear spectroscopy

Linear Spectroscopy. Measurements were performed in 3 mL Hellma quartz cuvette (material code QS blue, 200–2000 nm). Fluorescence and UV–visible spectra were respectively recorded using Spex FluoroMax-3 and FluoroMax-PLUS Jobin-Yvon Horiba equipped with R13456 PMT and Hitachi U-2900. Quantum yields measurements were performed at room temperature with solutions of A<0.1 to avoid re-absorption of the emitted light. Fluorescence quantum yields were measured according to the Crosby method using Coumarin 153 (λ_{ex} = 420 or 440 nm, Φ = 0.38 in EtOH).[2]

d) Two-photon absorption

The two-photon excitation spectra were measured by the fluorescence method by using a femtosecond laser Insight DS (680–1300 nm) with pulse width < 120 fs and a repetition rate of 80 MHz (Spectra-Physics). The

excitation beam was collimated over the cell length (Helma fluorescence semi-micro 10 × 4 mm) and the fluorescence, collected at 90◦ of the excitation beam, was focused into an optical fiber connected to a spectrometer (AvaSpec ULS from Avantes). The incident beam intensity was adjusted to ensure an intensity-squared dependence of the fluorescence over the whole spectral range reported. To avoid reabsorption of emitted light, micro cells are used and the focal point is very close to the edge of the cuvette. This allows elimination of second-order filter effects. The concentrations were 10^{-4} M for all probes. Calibration of the spectra was performed by comparison with the published Rhodamine B in MeOH in the 750–880 nm range and Fluorescein in 0.1 N NaOH solution in the 880–930 nm range.[3,4]

e) Quantum calculations

Geometry optimizations of the ground state were carried out at the B3LYP/6-311+G(d,p) level of theory. A frequency calculation was done in all cases to confirm the true minimum nature (no imaginary frequency) of the converged geometry. TD-DFT calculations were done at the M06-2X/6-311+G(d,p) level of theory using IEFPCM model to include solvent (water) effect. All calculations were done with Gaussian 16 (Revision B.01) software[5] from Gaussian Inc. and orbitals were plotted with GaussView 6.0.

II) Synthesis procedure:

TzProbe:

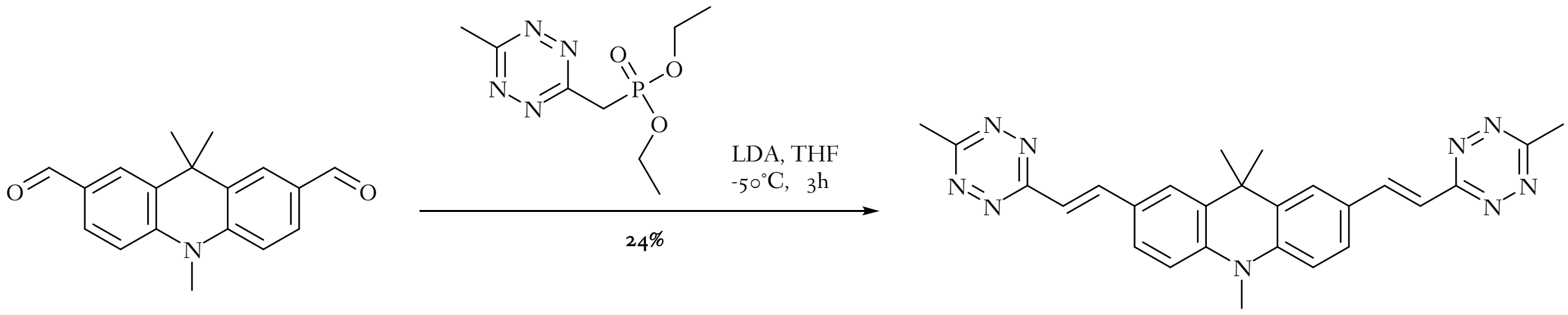

Under Ar atmosphere and at -50°C, Acri-2CHO[6] (1 eq, 54 mg, 0.19 mmol) and tetrazine phosphonate[7] (2.2 eq, 104,7 mg, 0.43 mmol) were solubilized in dry THF (3.5 mL). A solution of LDA 2M (2.5 eq, 0.24 mL, 0.48 mmol) was then added dropwise. The solution was stirred at -50°C for 3h. After the completion of the reaction, the reaction mixture was quenched with a saturated aqueous solution of NH_4Cl. Water was added and the aqueous layer was extracted three times with EA. Organic layers were washed with brine, dried over Na_2SO_4, filtered and concentrated under reduced pressure. The crude was purified by flash chromatography on silica gel (CH/EA, 10:0 to 1:1) to give the expected product as a red solid (20 mg, 0.04 mmol, 24 %).

^{1}H NMR (300 MHz, $CDCl_3$) δ 8.32 (d, *J* = 16.0 Hz, 2H), 7.77 (d, *J* = 2.0 Hz, 2H), 7.60 (dd, *J* = 8.5, 2.0 Hz, 2H), 7.37 (d, *J* = 16.0 Hz, 2H), 7.05 (d, *J* = 8.5 Hz, 2H), 3.57 (s, 3H), 3.04 (s, 6H), 1.65 (s, 6H).
^{13}C NMR (75 MHz, $CDCl_3$) δ 166.0, 165.3, 143.1, 141.1, 133.0, 128.5, 127.5, 124.4, 117.5, 117.5, 113.3, 36.7, 34.1, 28.1, 21.3.
LRMS (ES+): m/z = 464.4 $[M+H]^+$, Exact mass: 464.23. **HPLC:** rt = 6.55 min.
HRMS (ES+): m/z calculated for $C_{26}H_{26}N_9^+$ 464.2311, found 464.2303.

TzMonoClick:

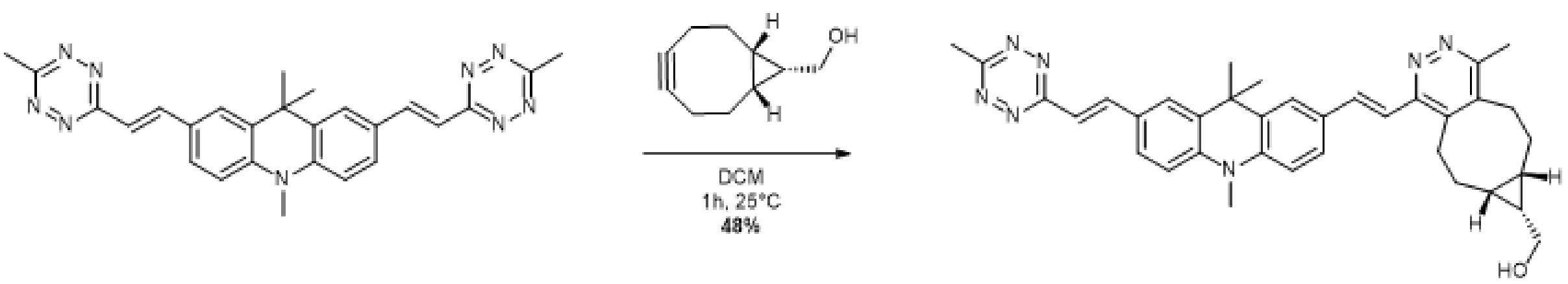

In a solution of **TzProbe** (1.0 eq., 30 mg, 0.065 mmol) in DCM (5 mL) was added BCN-OH (0.7 eq., 6.8 mg, 0.046 mmol). The orange reaction mixture was stirred for 1h at 25 °C and evaporated under *vacuum.* Flash chromatography was then performed with DCM/EtOH using a gradient from 100/0 to 90/10 to afford a mixture of non-purifiable stereoisomers as an orange powder (13 mg, 0.022 mmol, 48%).

^{1}H NMR (300 MHz, $CDCl_3$) δ 8.33 (d, *J* = 16.0 Hz, 1H), 7.95 (d, *J* = 15.5 Hz, 1H), 7.77 (d, *J* = 2.0 Hz, 1H), 7.68 (d, *J* = 2.0 Hz, 1H), 7.60 (dd, *J* = 8.5, 2.0 Hz, 1H),7.53 (dd, *J* = 8.5, 2.0 Hz, 1H), 7.37 (d, *J* = 16.0 Hz, 1H), 7.27 (d, *J* = 15.5 Hz, 1H), 7.03 (dd, *J* = 8.5, 5.5 Hz, 2H), 3.77 (d, *J* = 7.5 Hz, 2H), 3.56 (s, 3H), 3.05 (s, 3H), 3.01 – 2.87 (m, 4H), 2.74 (s, 3H), 2.50 – 2.29 (m, 2H), 1.65 (s, 6H), 1.31 – 1.07 (m, 3H), 0.95 - 0.82 (m, 2H).
^{13}C NMR (75 MHz, $CDCl_3$) δ 165.8, 165.7, 165.2, 156.6, 155.5, 143.4, 141.7, 141.2, 140.3 (br s), 138.8 (br s), 136.1, 132.9, 132.6, 130.0, 127.9, 127.3, 126.1, 124.2, 123.5, 118.4, 117.0, 112.9, 112.9, 59.4, 36.6, 33.9, 27.9, 27.1, 26.3, 22.0 (br s), 21.1, 20.7.

LRMS (ES+):m/z = 586.4 $[M+H]^+$. Exact mass: 586.32. **HPLC:** rt = 4.95min

HRMS (ES+): m/z calculated for $C_{36}H_{40}N_7O^+$ 586.3294, found 586.3306.

TzDiClick:

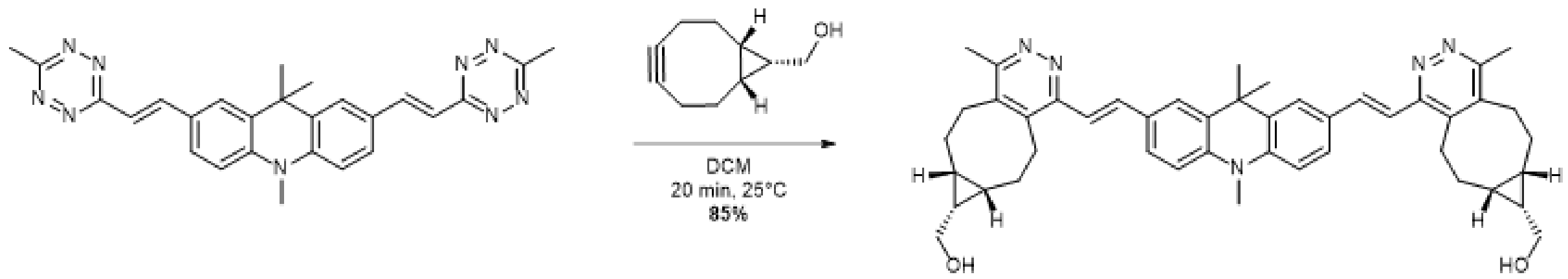

In a solution of **TzProbe** (1 eq., 10 mg, 0.022 mmol) in DCM (2 mL) was added BCN-OH (10 eq., 32 mg, 0.022 mmol). The orange reaction mixture was stirred for 20 min at 25 °C and evaporated under *vacuum*. Flash chromatography was then performed with DCM/EtOH using a gradient from 100/0 to 90/10 to afford a mixture of non-purifiable stereoisomers as an orange powder (13 mg, 0.018 mmol, 85%).

^{1}H NMR (300 MHz, $CDCl_3$) δ 7.96 (d, *J* = 15.5, 2H), 7.65 (d, *J* = 2.0 Hz, 2H), 7.51 (dd, *J* = 8.5, 2.0 Hz, 2H), 7.23 (d, *J* = 15.5 Hz, 2H), 6.98 (d, *J* = 8.5 Hz, 2H), 3.74 (d, *J* = 7.0 Hz, 4H), 3.52 (s, 3H), 3.11 – 2.87 (m, 8H), 2.74 (s, 6H), 2.48 – 2.32 (m, 4H), 1.62 (s, 6H), 1.31 – 1.21 (m, 4H), 1.14 (p, *J* = 8.0 Hz, 2H), 0.86 (d, *J* = 7.0 Hz, 4H).

^{13}C NMR (75 MHz, $CDCl_3$) δ 156.7, 155.7, 142.1, 140.0, 138.4, 136.0, 132.7, 129.8, 126.0, 123.6, 118.5, 112.8, 59.4, 36.6, 33.8, 27.8, 27.2, 26.3, 22.1 (br s), 21.0.

LRMS (ES+): m/z = 708.6 $[M+H]^+$ Exact mass: 708.42. **HPLC:** rt = 4.17min

HRMS (ES+): m/z calculated for $C_{46}H_{53}N_5O_2^+$ 708.4278, found 708.4283

2,2'(nitrosoazanediyl)diacetic acid:

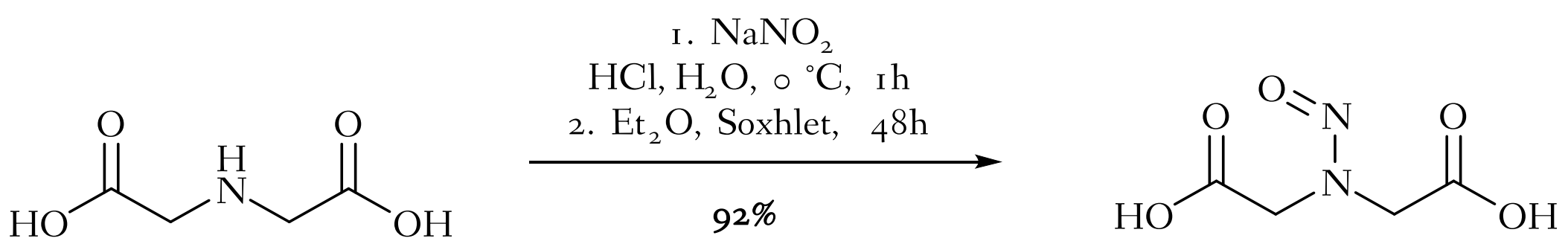

To a stirred solution of iminodiacetic acid (1.0 eq., 6.7 g, 50.3 mmol) in hydrochloric acid (37%, 8.4 mL), and water (12.5 mL) at 0 °C was added dropwise a solution of sodium nitrite (2.0 eq., 6.9 g, 100.6 mmol) in water (25.0 mL). The solution was warmed to 20 °C, and the solvent was removed under *vacuum*. Then, Et_2O was added to the reaction mixture and stirred for 48 h in Soxhlet extractor. The solvent of extraction was evaporated under *vacuum* to afford the product as a white powder (7.4 g, 45.6 mmol, 92%).

The spectral data was consistent with the reported one.[8]

^{1}H NMR (400 MHz, DMSO-*d$_6$*) δ 5.06 (s, 2H), 4.29 (s, 2H), COOH unobserved.

^{13}C NMR (100 MHz, DMSO-*d$_6$*) δ 169.7, 167.0, 53.3, 46.6.

3-(carboxymethyl)-1,2,3-oxadiazol-3-ium-5-olate [4]:

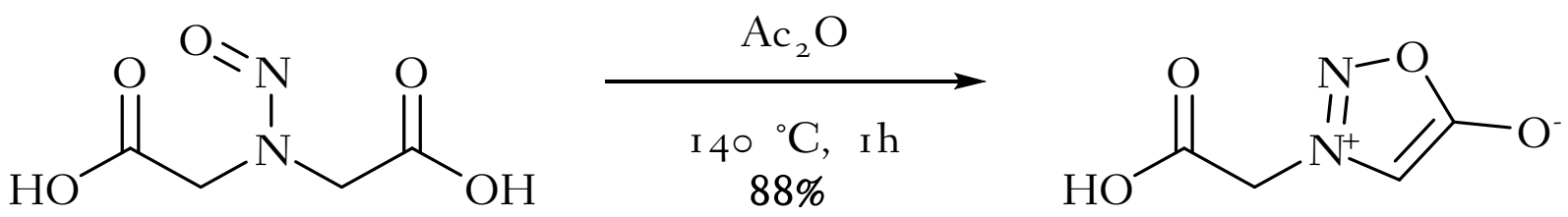

A solution of 2,2'-(nitrosoazanediyl)diacetic acid [3] (1.0 eq., 3.0 g, 18.5 mmol) in acetic anhydride (5.0 eq., 13.0 mL, 92.5 mmol) was heated at reflux for 30 min. The reaction mixture was cooled to 20 °C and the excess of acetic anhydride was removed under *vacuum*. Water (16 mL) was added to the residue, and the solution was extracted twice with DCM. The aqueous layer was then evaporated to afford the desired product as a brown solid (2.3 g, 15.9 mmol, 88%).

The spectral data was consistent with the reported one.[9]

^{1}H NMR (400 MHz, DMSO-*d_6*) δ 7.08 (s, 1H), 5.37 (s, 2H), COOH unobserved.
^{13}C NMR (100 MHz, DMSO-*d_6*) δ 168.6, 166.1, 97.2, 53.5.

3-(2-methoxy-2-oxoethyl)-1,2,3-oxadiazol-3-ium-5-olate [5]:

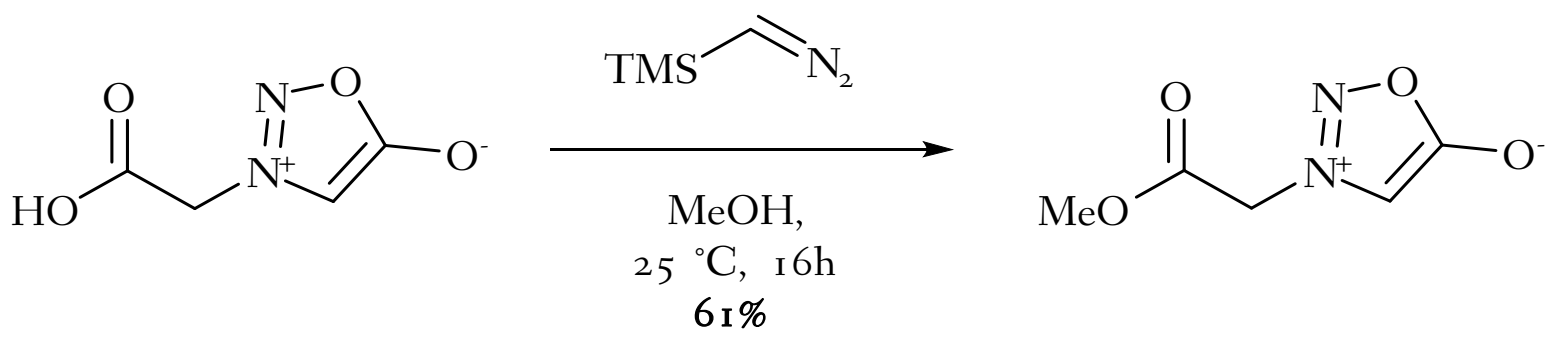

To a solution of (trimethylsilyl)diazomethane [4] (2.0 eq., 2 M in hexane, 4.0 mL, 8.1 mmol) was added dropwise a solution of 3-(carboxymethyl)-1,2,3-oxadiazol-3-ium-5-olate (1.0 eq., 580.0 mg, 4.0 mmol) in dry MeOH (9.3 mL) at 0 °C. The reaction mixture was stirred at room temperature overnight. The reaction was quenched with AcOH and the volatiles were removed under *vacuum*. The residue was diluted in EtOAc and was washed twice with H_2O. The organic layers were combined, dried over $MgSO_4$ and evaporated under *vacuum* to afford the desired product as a pale-yellow powder (311.0 mg, 2.0 mmol, 61 %).

The spectral data was consistent with the reported one.[8]

^{1}H NMR (400 MHz, $CDCl_3$) δ6.51 (s, 1H), 5.06 (s, 2H), 3.88 (s, 3H).
^{13}C NMR (100 MHz, $CDCl_3$) δ 169.1, 163.7, 96.9, 53.9, 53.2.

4-methoxy-2-((3-methoxyphenyl)amino)benzoic acid:

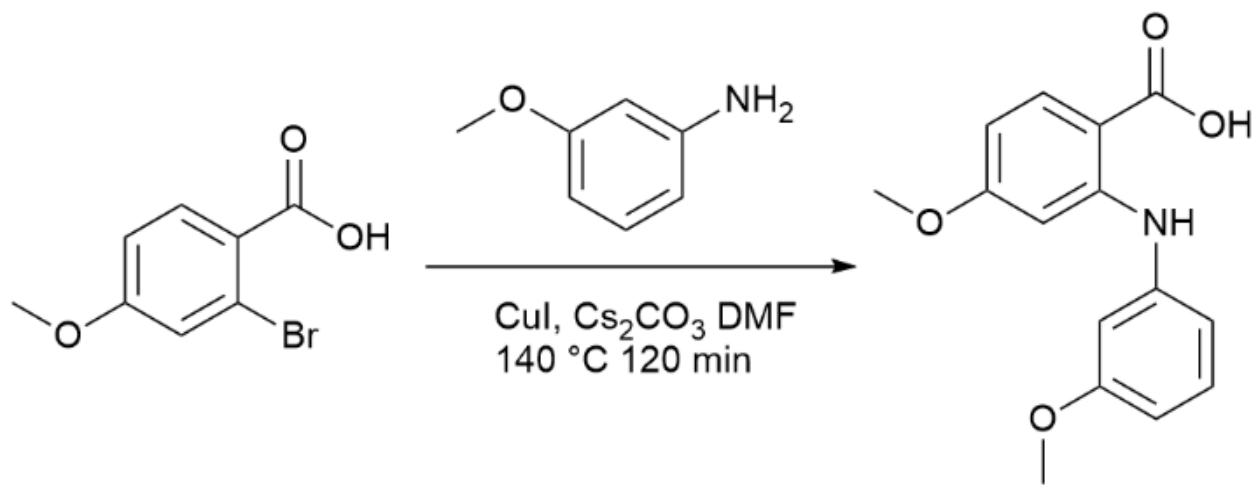

A solution of 2-bromo-4-methoxybenzoic acid (1 eq., 2500 mg, 10.82 mmol) was first added to a flask, followed by anhydrous DMF (40 mL) and then 3-methoxyaniline (1.5 eq., 2000 mg, 1.81 mL, 16.23 mmol). The solution was then treated with CuI (0.5 eq., 78 mg, 0.408 mmol) and Cs_2CO_3 (1.5 eq, 399 mg, 1.22 mmol). The mixture was degassed by sparging with argon for 15 minutes and then heated at 140 °C for 3 h. The reaction mixture was cooled to room temperature, and the solvent was evaporated in vacuo. DCM (75mL) was added, and the mixture was extracted with 1N aq. NaOH (3x50mL). The combined aqueous layers were treated with conc. HCl until pH ~ 1 was reached and extracted with DCM (3 × 50 mL) and dried ($MgSO_4$), filtered and solvent was removed in vacuo to afford the crude product which was used without further purification.[10]

methyl 4-methoxy-2-((3-methoxyphenyl)amino)benzoate:

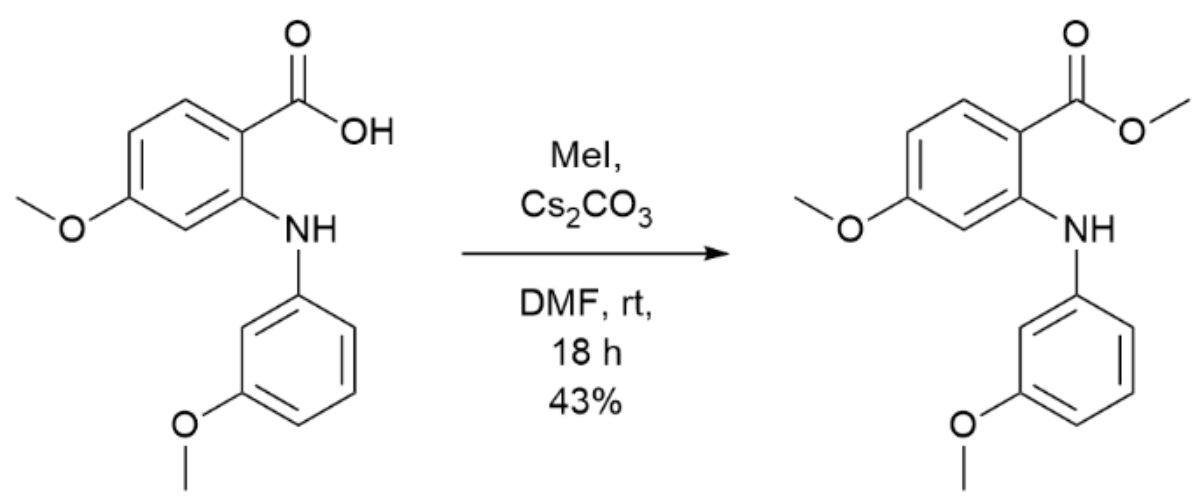

In a flask, 4-methoxy-2-[(3-methoxyphenyl)amino]benzoic acid (1 eq., 2957 mg, 10.82 mmol) is dissolved in 10 mL of DMF, and K_2CO_3 (1.5 eq., 2243 mg, 16.23 mmol) is added to the solution. The flask is then filled with argon gas. MeI (1.5 eq., 2304 mg, 1.01 mL, 16.23 mmol) is added to DMF (30mL), and this solution is added dropwise to the reaction mixture. After being stirred at room temperature for 10 h. The solvent was evaporated in vacuo, then water (50 mL) was added, and the mixture was extracted with DCM (3 × 50 mL). The combined organic layers were dried (Na_2SO_4), filtered and the solvent was removed in vacuo. The crude product was purified via flash column chromatography (CH/DCM = 1:0 to 2:1) to give methyl 4-methoxy-2-[(3-methoxyphenyl)amino]benzoate as a yellow oil (two step yield: 43%).

The spectral data was consistent with the reported one.[11]

¹H NMR (300 MHz, $CDCl_3$) δ 9.61 (s, 1H), 7.91 (d, *J* = 9.0 Hz, 1H), 7.26 (t, *J* = 4.0 Hz, 1H), 7.06 – 6.85 (m, 1H), 6.81 (t, *J* = 2.5 Hz, 1H), 6.77 (d, *J* = 2.5 Hz, 1H), 6.65 (ddd, *J* = 8.5, 2.5, 1.0 Hz, 1H), 6.31 (dd, *J* = 9.0, 2.5 Hz, 1H), 3.87 (s, 4H), 3.80 (s, 3H), 3.75 (s, 3H).

LRMS (ES+): m/z = 288.20 $[M+H]^+$, Exact mass: 288.12. **HPLC:** rt = 7.16 min.

2-(4-methoxy-2-((3-methoxyphenyl)amino)phenyl)propan-2-ol:

In sealed tube, a solution of methyl 4-methoxy-2-[(3-methoxyphenyl)amino]benzoate (1 eq., 455 mg, 1.58 mmol) in anhydrous oxolane (6 mL) and Et_2O (6 mL) was cooled to 0 °C, treated with MeMgI (4 eq., 2.11 mL, 6.33 mmol), let warm up to room temperature and stirred for 2 days while it was protected against light. Subsequently, the reaction mixture was cooled to 0 °C and saturated aq. NH_4Cl solution (20 mL) was carefully added. After gas evolution, water (30 mL) was added, and the mixture was extracted with DCM (3 × 30 mL). The combined organic layers were washed with water (20 mL), dried (Na_2SO_4), filtered and the solvent was removed in vacuo to afford the crude product which was used without further purification.[10]

3,6-dimethoxy-9,9-dimethyl-9,10-dihydroacridine [6]:

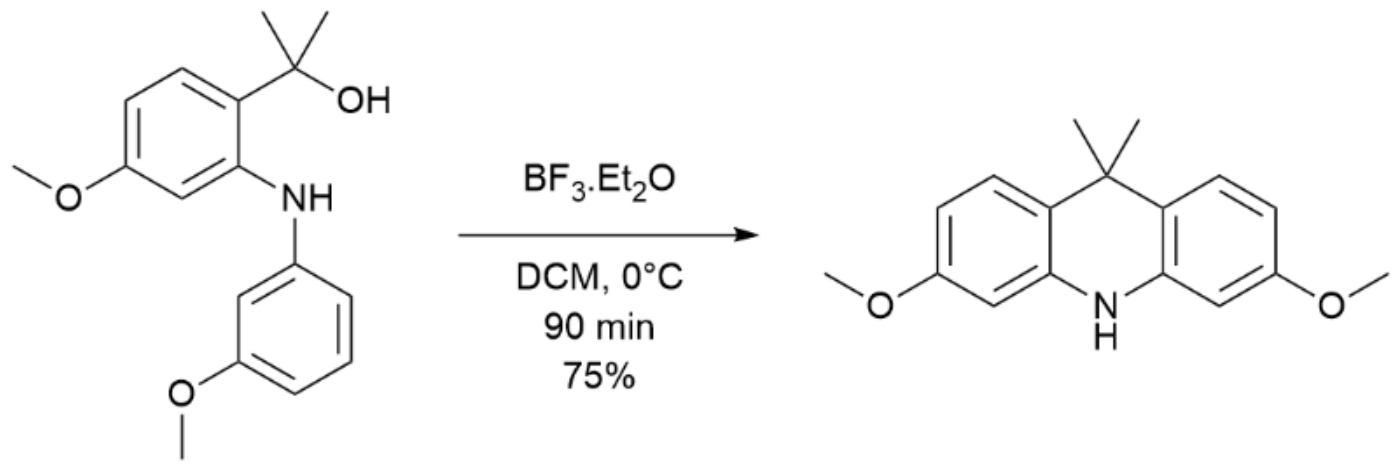

The crude 2-{4-methoxy-2-[(3-methoxyphenyl)amino]phenyl}propan-2-ol (1 eq., 270.12 mg, 0.94 mmol) was dissolved in anhydrous DCM (5.5 mL), cooled to 0 °C and treated with $BF_3.Et_2O$ (3.5 eq., 467 mg, 0.42 mL, 3.29 mmol). The reaction mixture was stirred at 0 °C and let warm up to rt for 120 min. Subsequently saturated aq. $NaHCO_3$ (10mL) solution were added under vigorous stirring, the organic layer was separated, and the aqueous layer was extracted with DCM (3 × 10 mL). The combined organic layers were dried (Na_2SO_4), filtered, and the solvent was removed in vacuo. The crude product was purified via flash chromatography (CH/DCM: 3:1 to 1:1).

The spectral data was consistent with the reported one.[10]

¹H NMR (300 MHz, $CDCl_3$) δ 7.26 (d, *J* = 8.5, 2H), 6.48 (dd, *J* = 8.5, 2.5 Hz, 2H), 6.22 (d, *J* = 2.5 Hz, 2H), 6.09 (s, 1H), 3.78 (s, 6H), 1.53 (s, 6H).

LRMS (ES+): m/z = 270.26 $[M+H]^+$, Exact mass: 270.15. **HPLC:** rt = 6.93 min.

3,6-dimethoxy-9,9,10-trimethyl-9,10-dihydroacridine [7]:

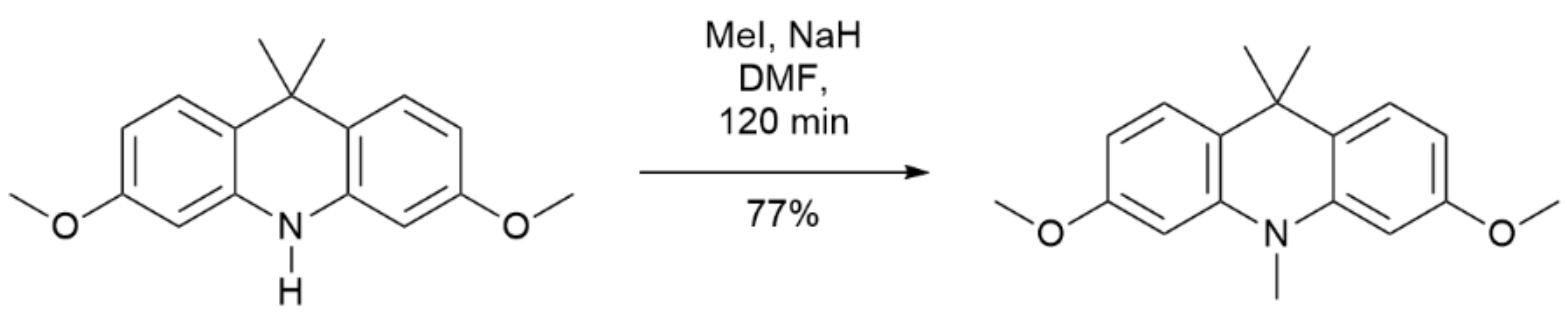

A round bottom flask was charged with 3,6-dimethoxy-9,9-dimethyl-9,10-dihydroacridine [6] (1 eq., 2500 mg, 9.28 mmol) and DMF (62 mL). NaH (2 eq., 742 mg, 18,56 mmol) and MeI (2 eq., 1.16 mL, 18.56 mmol) were subsequently added. The reaction was carried out 2h under an argon atmosphere. The DMF was evaporated and NH_4Cl solution (100 mL) and the mixture was extracted with DCM (3 × 100 mL). Flash chromatography was then performed using a gradient of 80:20 to 30:70 over 15 minutes to afford the product as a white powder (2.04 g, 7,1 mmol, 77%).[6]

^{1}H NMR (300 MHz, $CDCl_3$) □ 7.29 (d, J = 8.5 Hz, 2H), 6.54 – 6.48 (m, 4H), 3.82 (s, 6H), 3.39 (s, 3H), 1.49 (s, 6H).

^{13}C NMR (75 MHz, $CDCl_3$) δ 158.3 (2C), 143.0 (2C), 125.7 (2C), 124.0 (2C), 104.2 (2C), 99.4 (2C), 55.1 (2C), 35.2, 33.2, 27.8 (2C).

LRMS (ES+): *m/z* = 284.26 $[M+H]^+$Exact mass: 284.16. **HPLC:** rt= 7.32 min.

HRMS (ES+): calculated for $C_{18}H_{22}NO_2^+$ 284.1651 found 284.1651.

3,6-dimethoxy-9,9,10-trimethyl-9,10-dihydroacridine-2,7-dicarbaldehyde [8]:

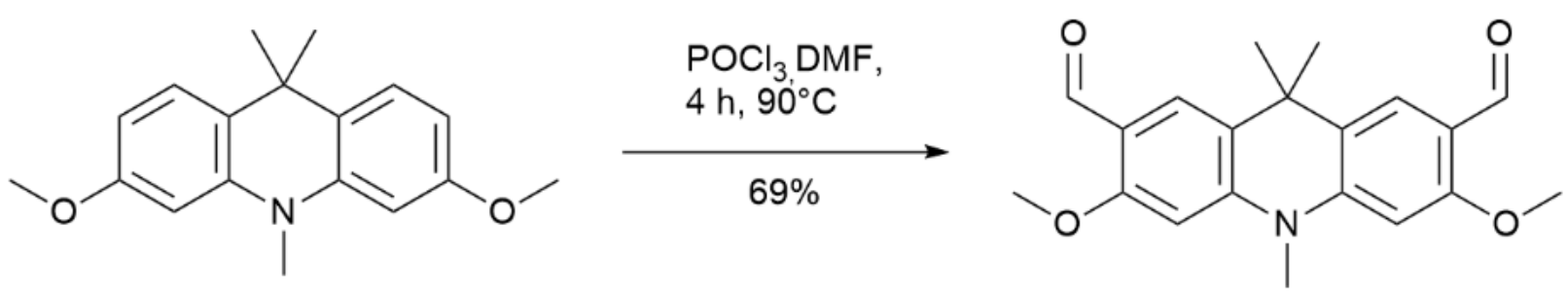

Under an argon atmosphere and at 0°C, POCl₃ (12 eq., 6395.3 mg, 3.89 mL, 41.71 mmol) was added to DMF (3.23 mL). The resulting solution was stirred at 50°C for 30 minutes. Subsequently, 3,6-dimethoxy-9,9,10-trimethyl-9,10-dihydroacridine (1 eq., 985 mg, 3.48 mmol) was dissolved in DMF (15 mL) and added to the mixture, which was then heated to 95°C for 3 hours. After 3 hours, the reaction was quenched by adding the reaction mixture dropwise to water under vigorous stirring and allowed to stir overnight at ambient

temperature. The aqueous phase was then basified with 3M NaOH. DCM was added for extraction (2 × 75mL). To remove unwanted by-products, a flash chromatography was performed using 100% DCM to afford the product as a yellow powder (815 mg, 2.4 mmol, 69%).[6]

^{1}H NMR (300 MHz, $CDCl_3$) δ 10.33 (s, 2H), 7.90 (s, 2H), 6.47 (s, 2H), 3.98 (s, 6H), 3.61 (s, 3H), 1.53 (s, 6H).

^{13}C NMR (75 MHz, $CDCl_3$) δ 188.3 (2C), 162.0 (2C), 147.4 (2C), 126.0 (2C), 125.0 (2C), 119.2 (2C), 96.4 (2C), 55.9 (2C), 35.6, 34.9, 28.7 (2C).

LRMS (ES+): *m/z* = 340.27 [M+H]$^+$, Exact mass: 340.15. **HPLC**: rt = 6.66 min.

HRMS (ES+): calculated for $C_{20}H_{22}NO_4^+$ 340.1543 found 340.1542.

3,6-dihydroxy-9,9,10-trimethyl-9,10-dihydroacridine-2,7-dicarbaldehyde [9]:

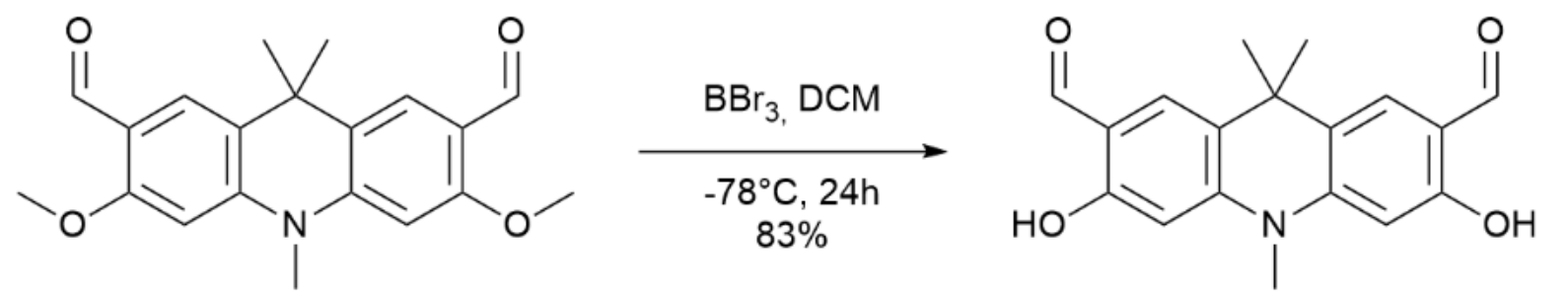

A solution of 3,6-dimethoxy-9,9,10-trimethyl-9,10-dihydroacridine-2,7-dicarbaldehyde (1 eq., 500 mg, 1.47 mmol) in anhydrous DCM (12 mL) was cooled to -78 °C. BBr_3 (15 eq., 5536.093 mg, 2.089 mL, 22.098 mmol) was added, stirred for 30 min and then warmed to room temperature and stirred for 24 h. The reaction mixture was cooled to 0 °C and 1 M aq. HCl (0.5 mL) was added and stirred vigorously for 30 min. Saturated aq. $NaHCO_3$ solution (10 mL) was added, and the organic layer was separated. The aqueous layer was extracted with EA (3 × 10 mL) and combined organic layers were dried (Na_2SO_4), filtered and the solvent was removed in vacuo. The crude product was purified via flash column chromatography (CH/DCM: 1:3 to 0:1) to afford the product as a yellow powder (380 mg, 2.4 mmol, 83%). [10]

^{1}H NMR (300 MHz, $CDCl_3$) δ 11.36 (s, 2H), 9.77 (s, 2H), 7.50 (s, 2H), 6.54 (s, 2H), 3.50 (s, 3H), 1.55 (s, 6H).

^{13}C NMR (75 MHz, $CDCl_3$) δ 194.1 (C2), 162.7 (C2), 147.5 (C2), 128.8 (C2), 125.2 (C2), 115.3 (C2), 101.2 (C2), 34.97, 34.6, 28.4 (C2).

LRMS (ES+): *m/z* = 312.22 [M+H]$^+$, Exact mass: 312.12. **HPLC**: rt= 6.81 min.

HRMS (ES+): calculated for $C_{18}H_{18}NO_4^+$ 312.1230 found 312.1230.

SydProbe:

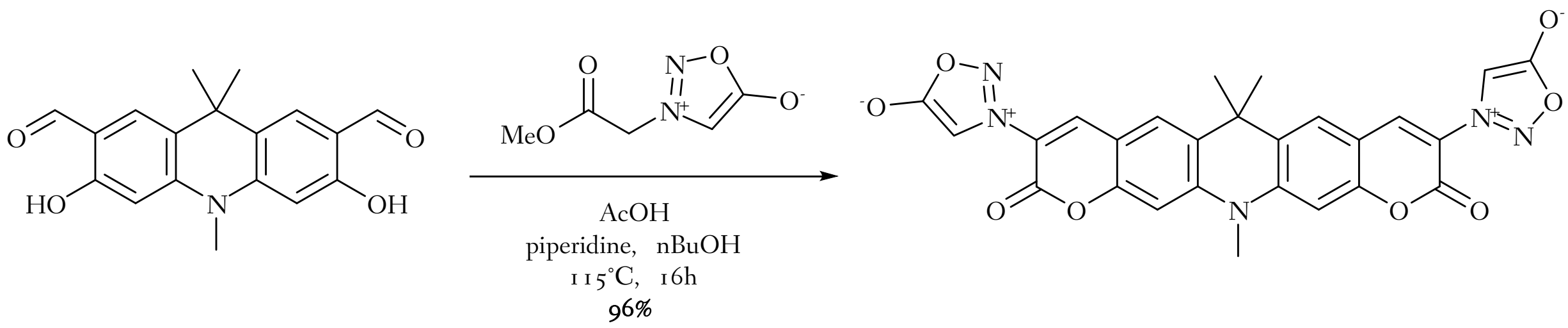

A mixture containing n-butanol (2 mL), dialdehyde [9] (1.0 eq., 100 mg, 0.32 mmol), 3-(2-methoxy-2-oxoethyl)-1,2,3-oxadiazol-3-ium-5-olate [5] (5.0 eq., 254 mg, 1.61 mmol), piperidine (1.0 eq., 32 μL) and AcOH (64 μL) was refluxed overnight. Upon cooling to room temperature, the reaction mixture was filtered after the addition of Et_2O to afford the desired product as an orange powder (162 mg, 0.31 mmol, 96%).

^{1}H NMR (400 MHz, DMSO-d_6) δ 8.82 (s, 2H), 8.07 (s, 2H), 7.51 (s, 2H), 7.43 (s, 2H), 3.67 (s, 3H), 1.60 (s, 6H).

^{13}C NMR (100 MHz, DMSO-d_6) δ 168.1 (2C), 154.9 (2C), 154.0 (2C), 145.7 (2C), 140.1 (2C), 130.3 (2C), 126.1 (2C), 118.0 (2C), 111.4 (2C), 101.5 (2C), 97.7 (2C), 36.1, 35.4, 28.5 (2C).

LRMS (ES^-): *m/z* = 528.3 $[M-H]^+$, Exact mass: 527.11. **HPLC**: rt = 3.23 min.

HRMS (ES+): calculated for $C_{26}H_{18}N_5O_8^+$ 528.1155 found 528.1153.

SydMonoClick:

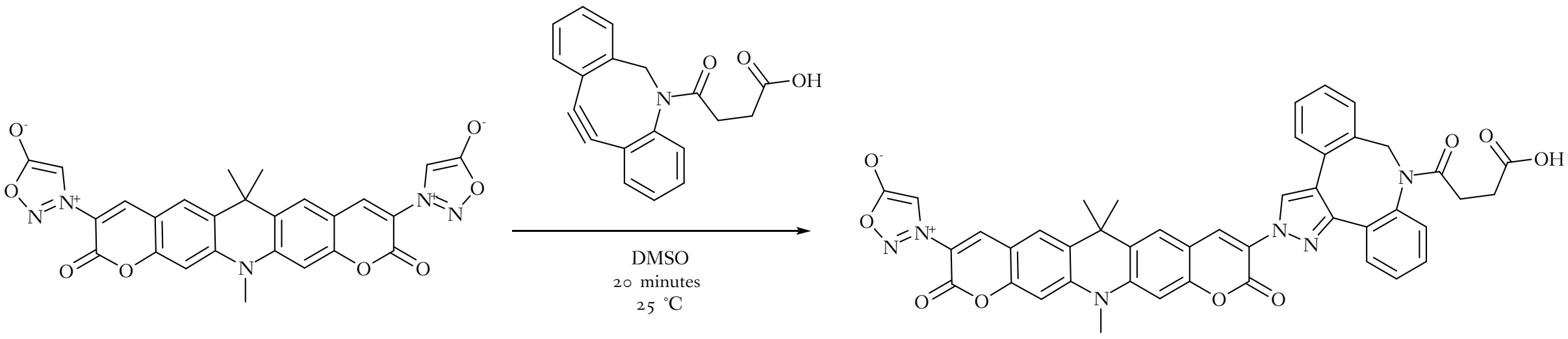

In a solution of **SydProbe** (1.0 eq., 50 mg, 0.09 mmol) in DMSO (3 mL) was added DBCO-acid (0.5 eq., 14 mg, 0.05 mmol). The orange reaction mixture was stirred for 20 minutes at 25 °C and evaporated under *vacuum.* Flash chromatography was then performed with ACN/Water + 0.1% TFA using a gradient from 5:95 to 100:0 to afford a mixture of non-purifiable regioisomers as an orange powder (29 mg, 0.04 mmol, 39%).

^{1}H NMR (400 MHz, DMSO-d_6) δ 8.80 (s, 1H), 8.66 (m, 2H), 8.03 (m, 2H), 7.15-7.61 (m, 11H), (d, *J*= 17.0 Hz, 0.5H of regioisomer A), 5.89 (d, *J*= 17.0 Hz, 0.5H of regioisomer B), 4.51 (d, *J*= 17.0 Hz, 0.5H regioisomer A), 4.49 (d, *J*= 17.0 Hz, 0.5H regioisomer B), 3.63 (s, 3H), 2.10-2.24 (m, 2H), 1.72-2.02 (m, 4H), 1.59 (s, 6H).

LRMS (ES^+): m/z = 789.4 $[M+H]^+$, Exact mass: 788.22. **HPLC**: rt = 3.62 min.

HRMS (ES+): calculated for $C_{44}H_{33}N_6O_9^+$ 789.2309 found 789.2308.

SydDiClick:

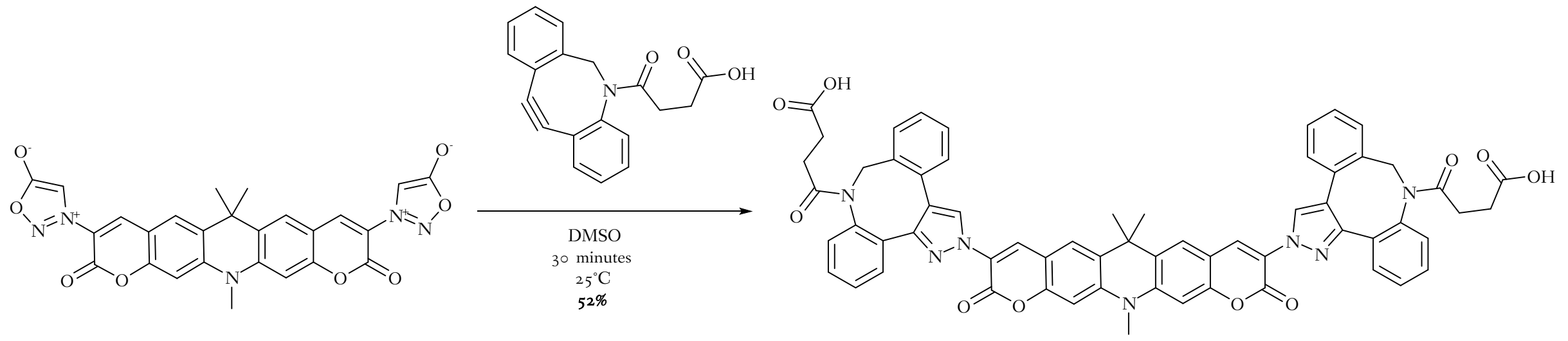

In a solution of **SydProbe** (1.0 eq., 50 mg, 0.09 mmol) in DMSO (3 mL) was added DBCO-acid (2 eq., 58 mg, 0.19 mmol). The orange reaction mixture was stirred for 30 minutes at 25 °C and evaporated under *vacuum.* Flash chromatography was then performed with ACN/Water + 0.1% TFA using a gradient from 5:95 to 100:0 to afford a mixture of non-purifiable regioisomers as a yellow powder (52 mg, 0.05 mmol, 52%).

^{1}H **NMR** (400 MHz, DMSO-*d*$_6$) δ 8.66 (m, 3H), 8.59 (m, 1H), 8.04 (m, 2H), 7.43 (m, 9H), 7.23 (m, 8H), 7.14 (m, 1H), 5.96 (d, *J* = 17.0 Hz, 1H regioisomer A), 5.89 (d, *J* = 17.0 Hz, 1H of regioisomer B), 4.53 (d, *J* = 17.0 Hz, 1H regioisomer A), 4.49 (d, *J* = 17.0 Hz, 1H regioisomer B), 3.62 (s, 3H), 2.12-2.26 (m, 4H), 1.70-2.02 (m, 4H), 1.59 (s, 6H).
^{13}C **NMR** (100 MHz, DMSO-*d*$_6$) δ 173.6, 173.5, 170.5, 170.4, 163.5 (2C), 156.6 (2C), 151.8 (2C), 151.1 (2C), 149.0 (2C), 143.7 (2C), 140.4 (2C), 140.0 (2C), 135.1, 134.3, 133.0, 132.8, 130.2, 130.0, 129.5 (2C), 129.2 (2C), 128.7 (2C), 128.3 (2C), 127.2 (2C), 126.9 (2C), 124.6 (2C), 122.5 (2C), 122.3 (2C), 119.7 (2C), 118.2 (2C), 112.7 (2C), 100.6 (2C), 51.6 (2C), 36.2, 34.9, 29.0 (2C), 28.9 (2C), 28.6 (2C).
LRMS (ES$^+$): m/z = 1050.7 [M+H]$^+$, Exact mass: 1049.34. **HPLC**: rt = 3.75 min.
HRMS (ES+): calculated for $C_{62}H_{48}N_7O_{10}^+$ 1050.3463 found 1050.3458.
DBCO-TPP:

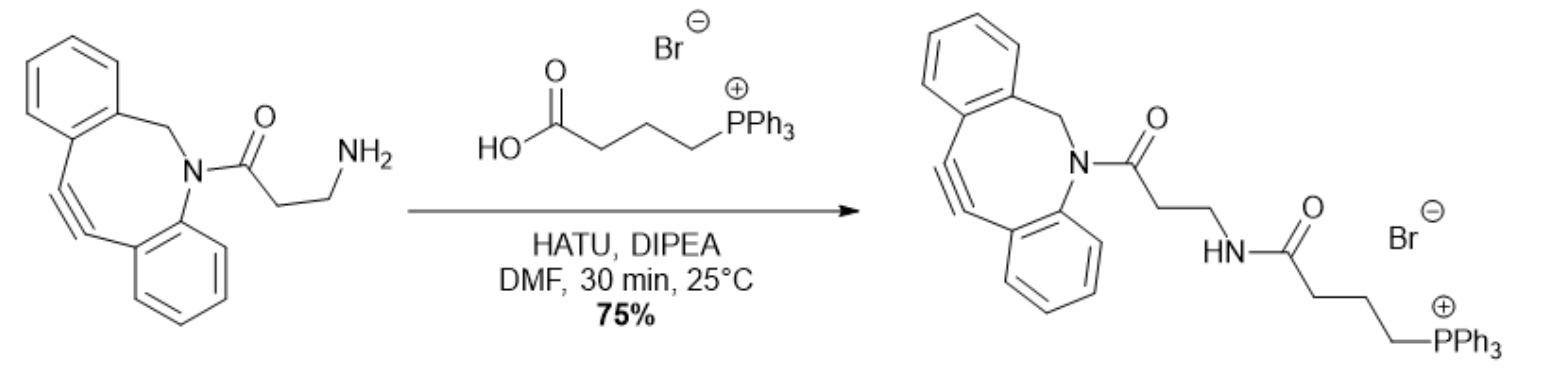

In a solution of (3-Carboxypropyl)triphenylphosphonium bromide (1.0 eq., 78 mg, 0.18 mmol) in DMF (2 mL), was added HATU (1.1 eq., 80 mg, 0.20 mmol) and DIPEA (2.0 eq., 60 µL, 0.36 mmol). The yellow limpid reaction mixture was left to stir 10 minutes at room temperature. Then, DBCO-amine (1.0 eq., 50 mg, 0.18 mmol) was added, and the reaction mixture was stirred at 25°C for 30 minutes. Purification was done on reverse phase chromatography (12 g C18), using eluent H_2O/ACN from 100:0 to 0:100, to afford the product as a white powder (83 mg, 0.14 mmol, 75%).

^{1}H **NMR** (400 MHz, $CDCl_3$) δ 7.77 (m, 3H), 7.67 (m, 11H), 7.48 (dt, *J* = 7.7, 1.1 Hz, 1H), 7.33 (m, 4H), 7.18 (m, 4H), 6.26 (t, *J* = 5.8 Hz, 1H), 4.92 (d, *J* = 14.0 Hz, 1H), 3.56 (d, *J* = 14.0 Hz, 1H), 3.22 (m, 4H), 2.53 (dt, *J* = 16.7, 6.0 Hz, 1H), 2.33 (q, *J* = 5.3 Hz, 2H), 1.91 (dt, *J* = 16.7, 6.0 Hz, 1H), 1.79 (m, 2H).

^{13}C NMR (100 MHz, $CDCl_3$) δ 171.7, 171.4, 151.3, 148.2, 135.3, 135.3, 133.6 (3C), 133.5 (3C), 132.2, 130.7 (3C), 130.6 (3C), 129.6, 129.0, 128.3, 128.3, 127.9, 127.0, 125.7, 123.0, 122.4, 118.5, 117.7, 114.9, 107.9, 77.4 (2C), 55.5, 35.6, 34.4, 21.4, 20.8, 18.6.

LRMS (ES^+): *m/z* = 607.4 $[M+H]^+$, Exact mass: 607.25. **HPLC**: rt = 3.09 min.

HRMS (ES+): calculated for $C_{40}H_{36}N_2O_2P^+$ 607.2515 found 607.2512.

III) Characterization of new compounds:

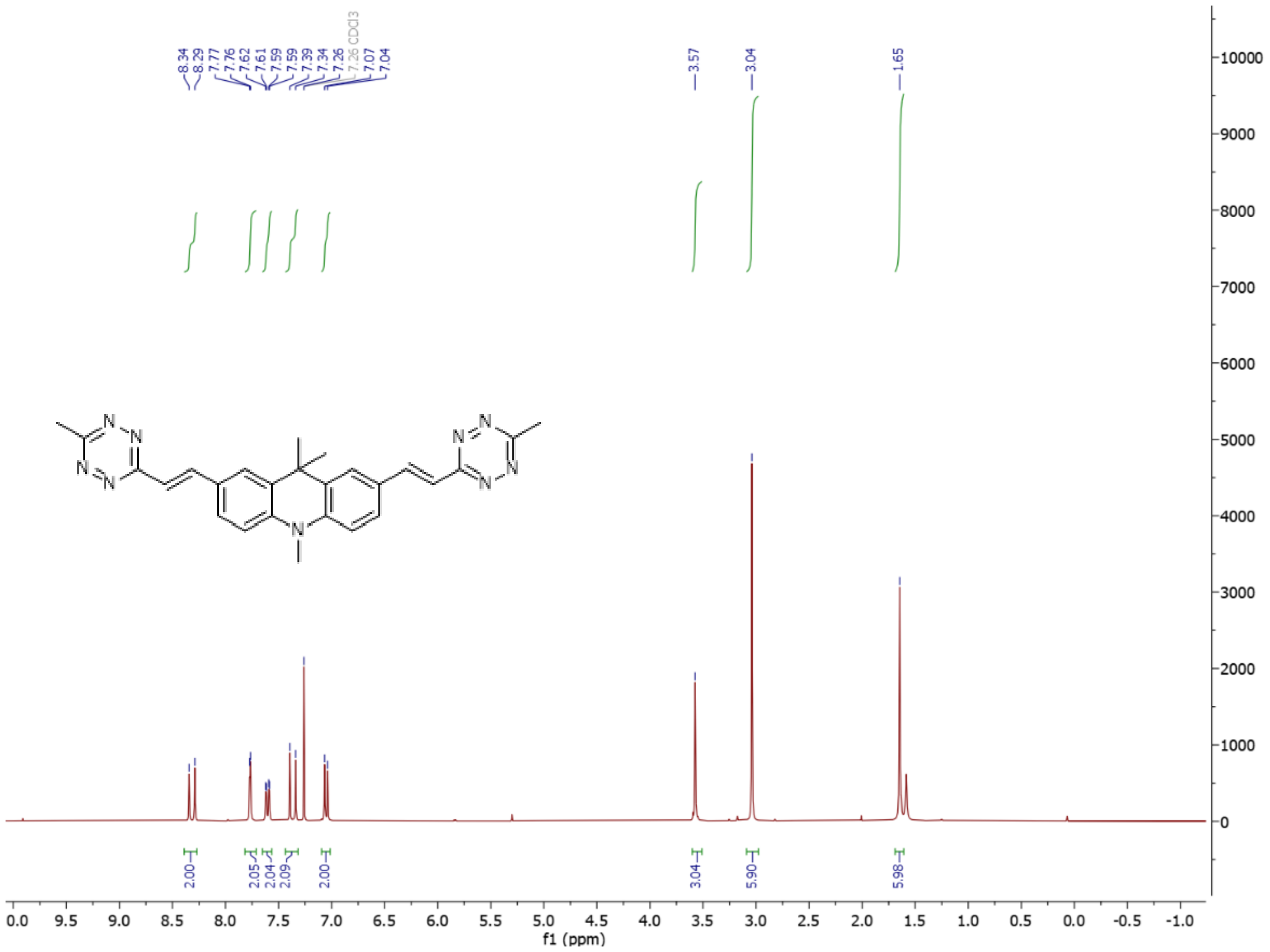

Fig. S1: ¹H NMR spectrum of compound [TzProbe] in $CDCl_3$ (300 MHz).

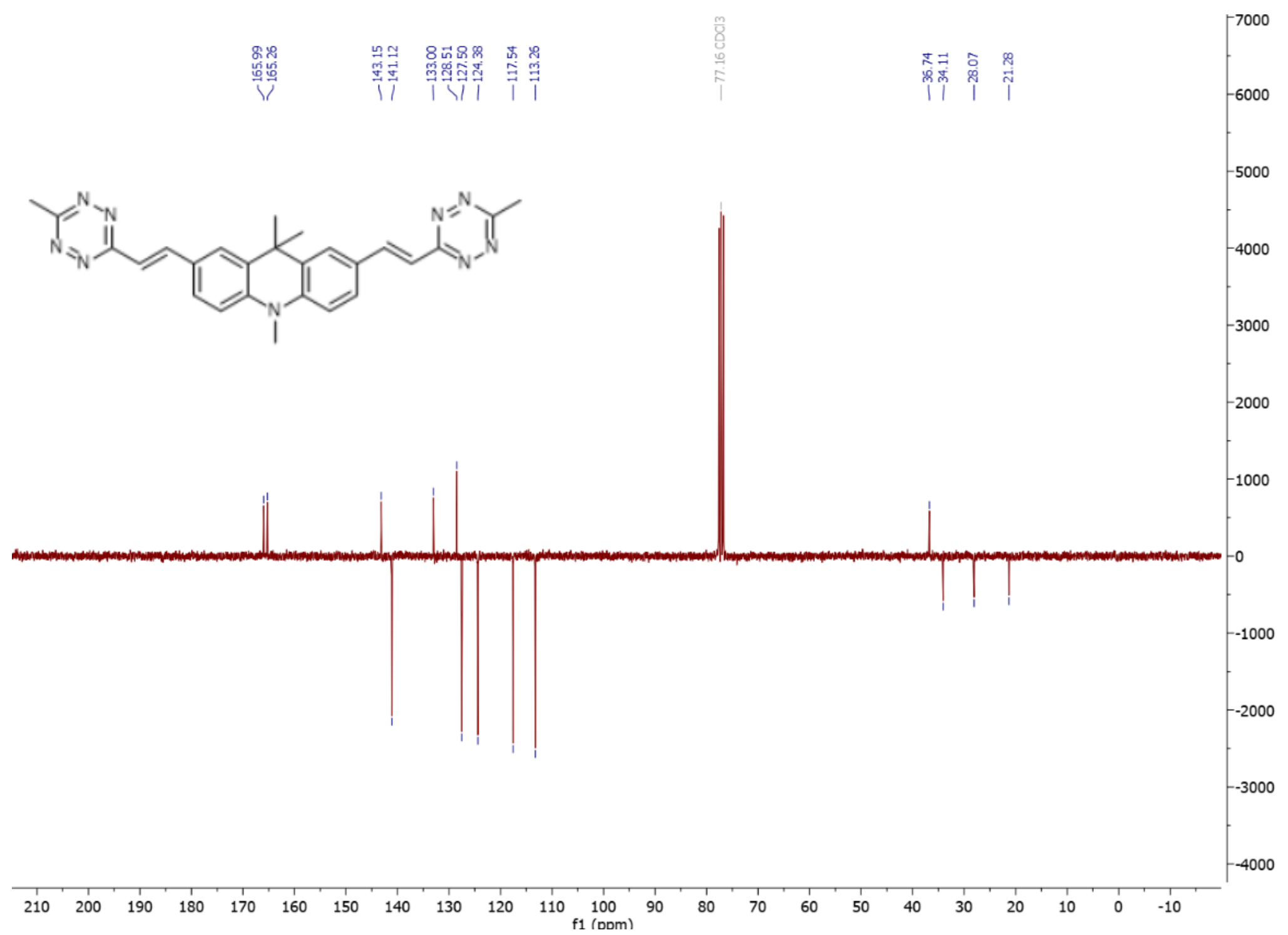

Fig. S2: ¹³C NMR spectrum of compound [TzProbe] in $CDCl_3$ (75 MHz).

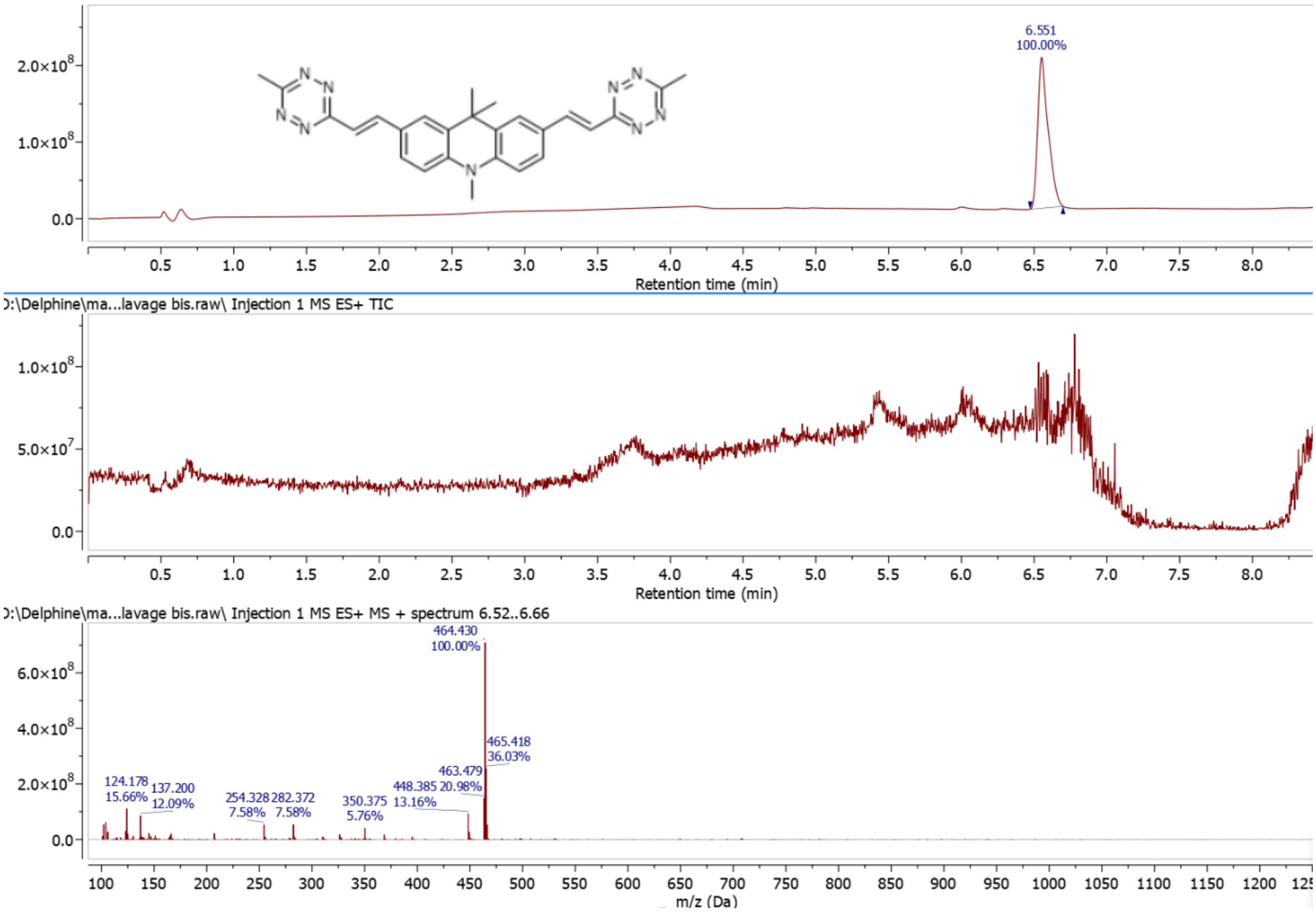

Fig. S3: LC-MS analysis of compound [TzProbe].

Elemental Composition Report

Page 1

Single Mass Analysis

Tolerance = 5.0 PPM / DBE: min = -1.5, max = 100.0
Element prediction: Off
Number of isotope peaks used for i-FIT = 9

Monoisotopic Mass, Even Electron Ions
617 formula(e) evaluated with 3 results within limits (all results (up to 1000) for each mass)
Elements Used:
C: 0-130 H: 0-150 N: 0-10 O: 0-10
CURIE_AR0238 21 (0.573) Cm (19:23)
1: TOF MS ES+

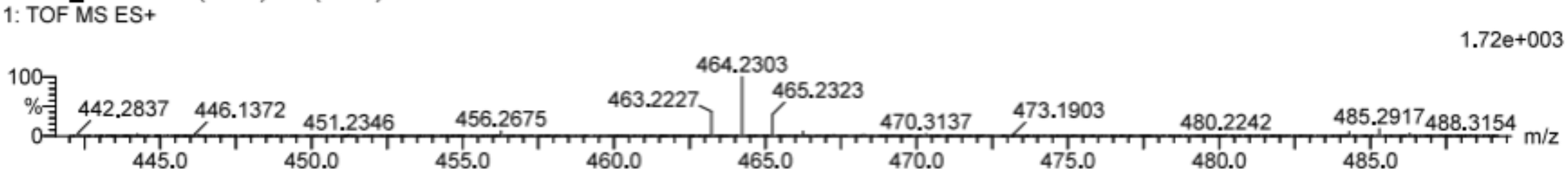

Minimum:				-1.5			
Maximum:		5.0	5.0	100.0			
Mass	Calc. Mass	mDa	PPM	DBE	i-FIT	i-FIT (Norm)	Formula
464.2303	464.2311	-0.8	-1.7	18.5	544.8	1.0	C26 H26 N9
	464.2298	0.5	1.1	13.5	544.8	1.0	C25 H30 N5 O4
	464.2284	1.9	4.1	8.5	545.4	1.5	C24 H34 N O8

Fig. S4: HR mass spectrum (ESI, positive mode (left)) of compound [TzProbe].

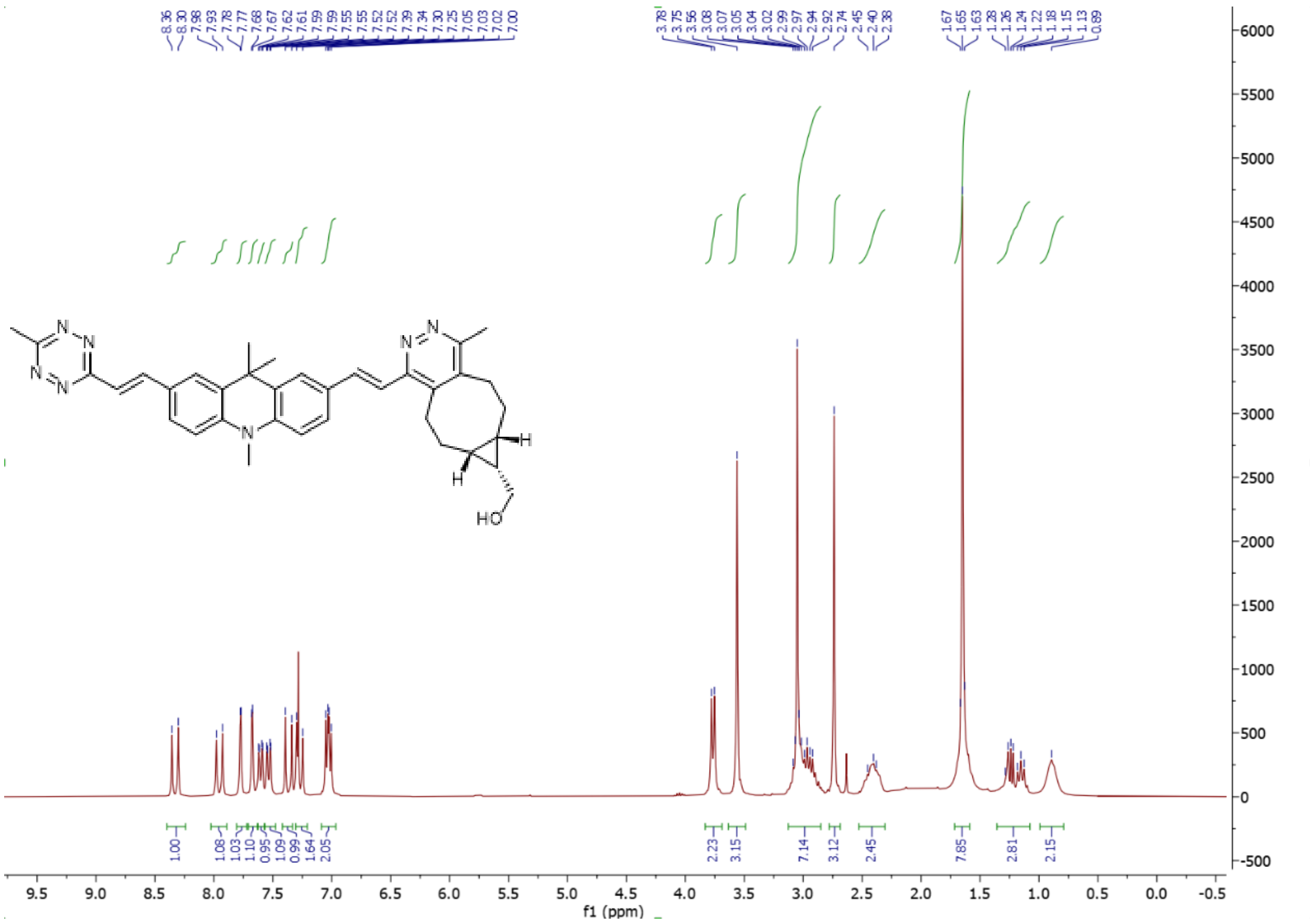

Fig. S5: [1] H NMR spectrum of compound [TzMonoClick] in $CDCl_3$, (300 MHz).

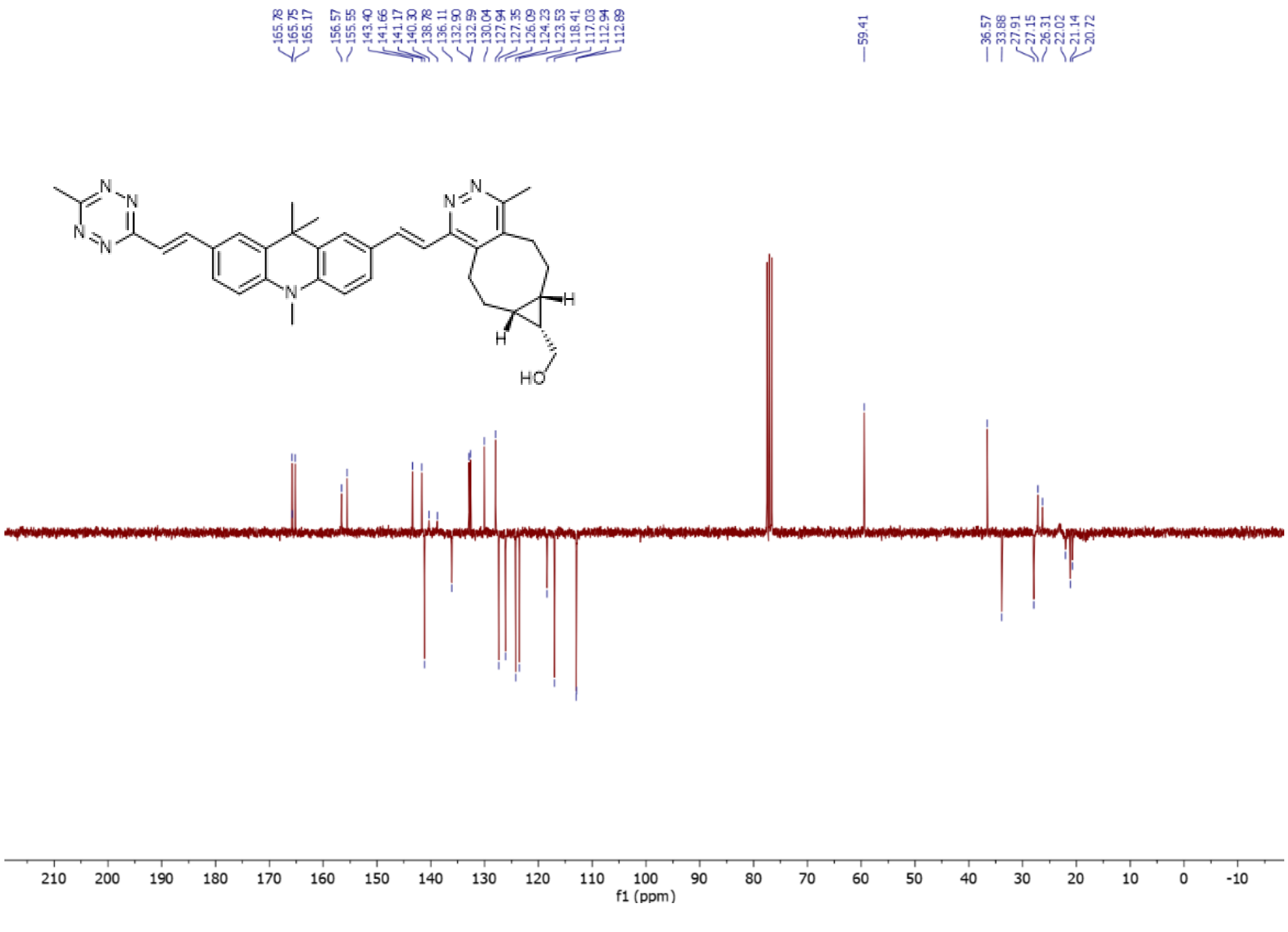

Fig. S6: [13] C NMR spectrum of compound [TzMonoClick] in $CDCl_3$ (75 MHz).

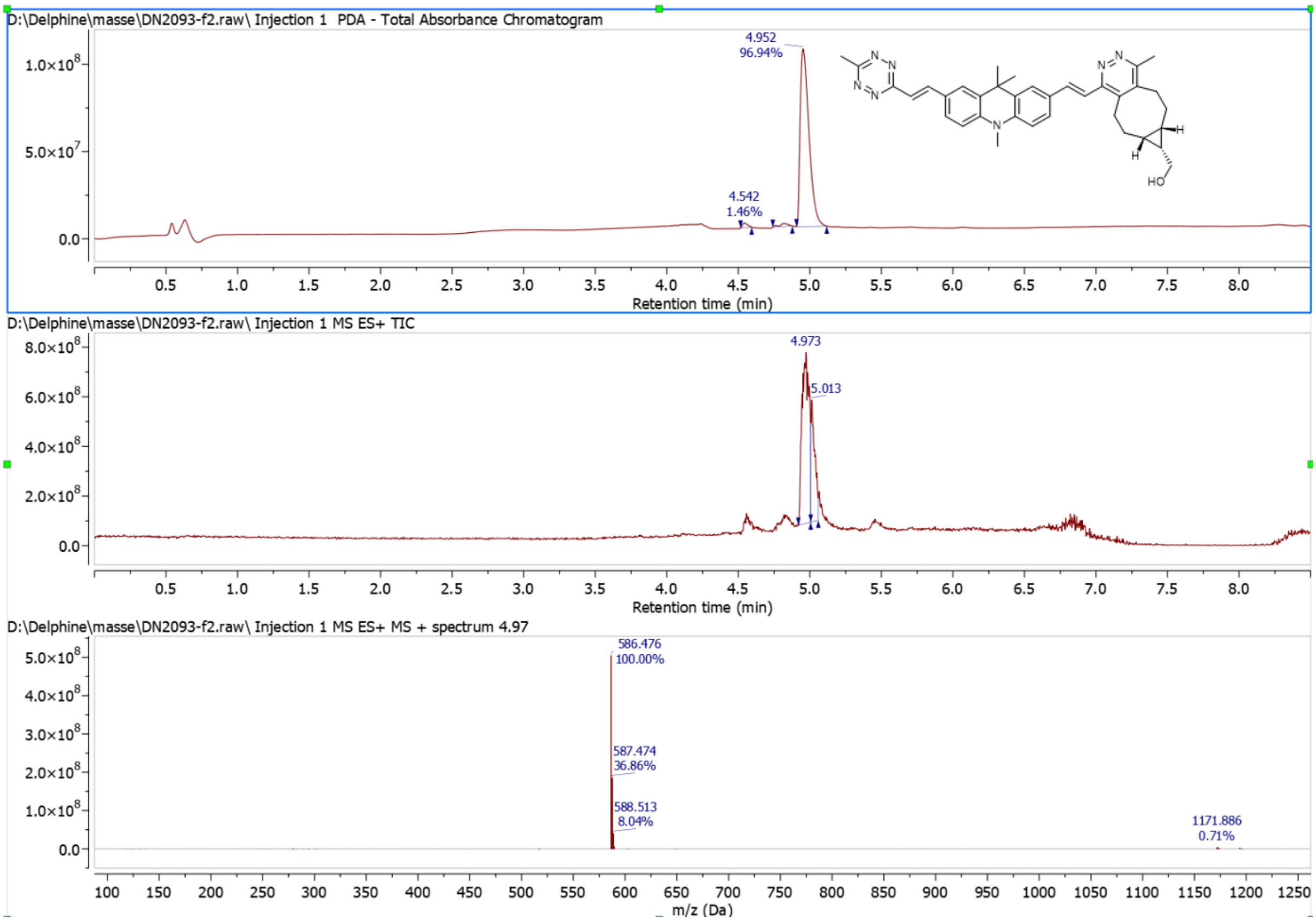

Fig. S7: LC-MS analysis of compound [TzMonoClick].

Elemental Composition Report **Page 1**

Single Mass Analysis
Tolerance = 5.0 PPM / DBE: min = -1.5, max = 50.0
Element prediction: Off
Number of isotope peaks used for i-FIT = 9

Monoisotopic Mass, Even Electron Ions
770 formula(e) evaluated with 4 results within limits (up to 50 best isotopic matches for each mass)
Elements Used:
C: 1-100 H: 1-120 N: 0-10 O: 0-10
DN2093-F2 26 (0.173) AM2 (Ar,30000.0,556.28,0.00,LS 10); ABS; Cm (24:29)
1: TOF MS ES+

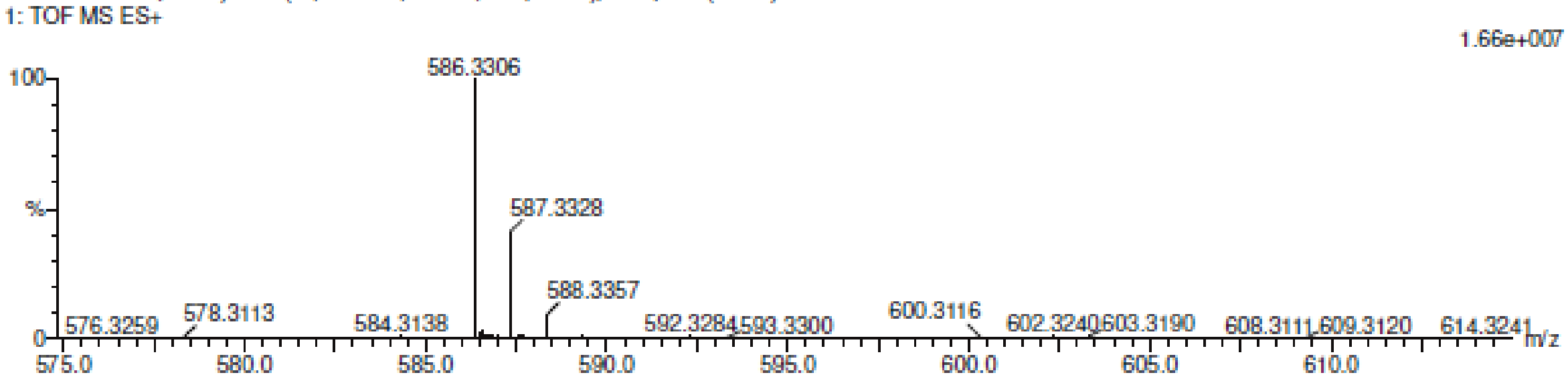

Minimum:				-1.5				
Maximum:		5.0	5.0	50.0				
Mass	Calc. Mass	mDa	PPM	DBE	i-FIT	Norm	Conf(%)	Formula
586.3306	586.3294	1.2	2.0	20.5	1927.0	0.211	80.94	C36 H40 N7 O
	586.3281	2.5	4.3	15.5	1928.6	1.823	16.15	C35 H44 N3 O5
	586.3321	-1.5	-2.6	19.5	1930.4	3.574	2.80	C40 H44 N O3
	586.3313	-0.7	-1.2	7.5	1933.7	6.924	0.10	C24 H44 N9 O8

Fig. S8: HR mass spectrum (ESI, positive mode (left)) of compound [TzMonoClick].

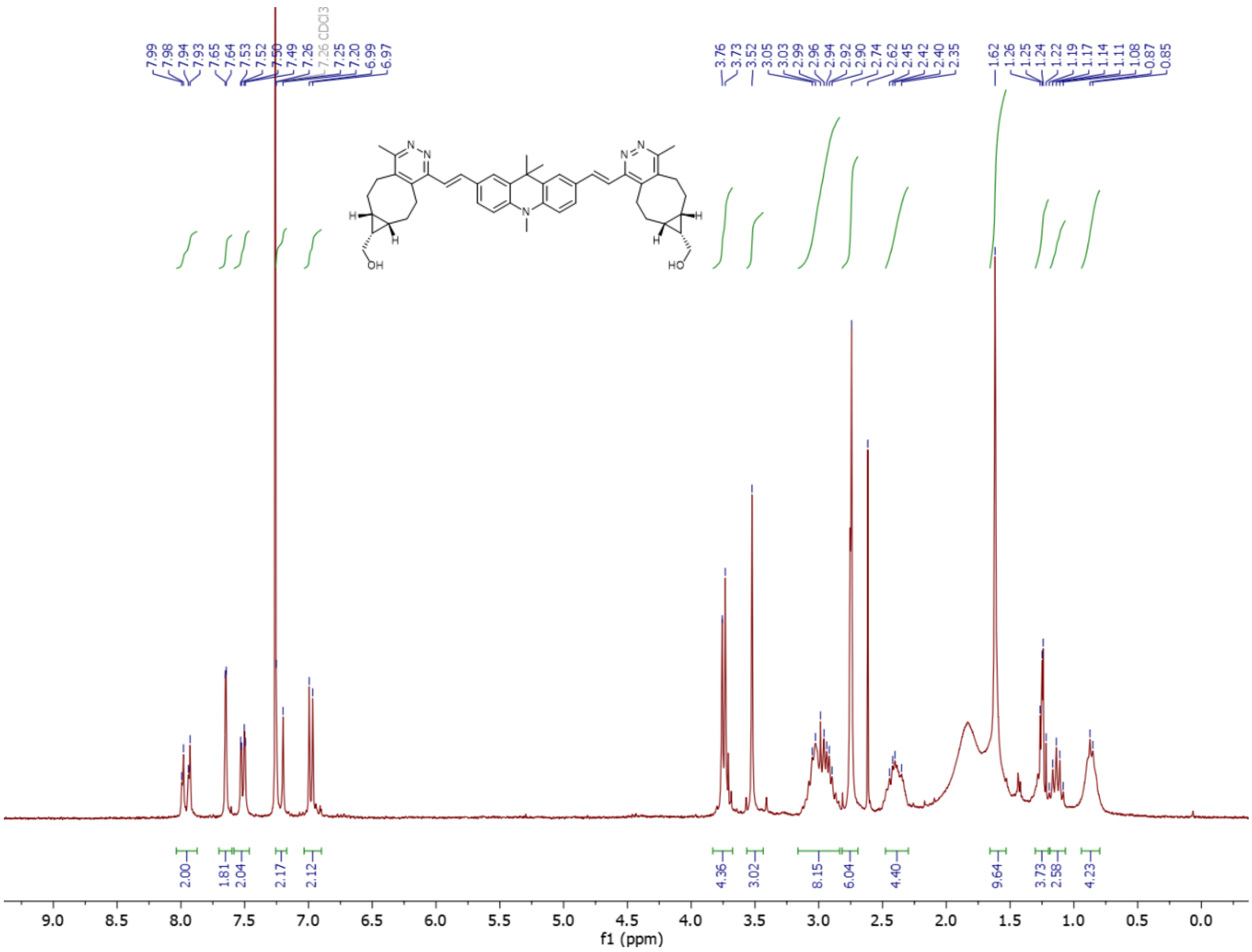

Fig. S9: ^{1}H NMR spectrum of compound [TzDiClick] in $CDCl_3$, (300 MHz).

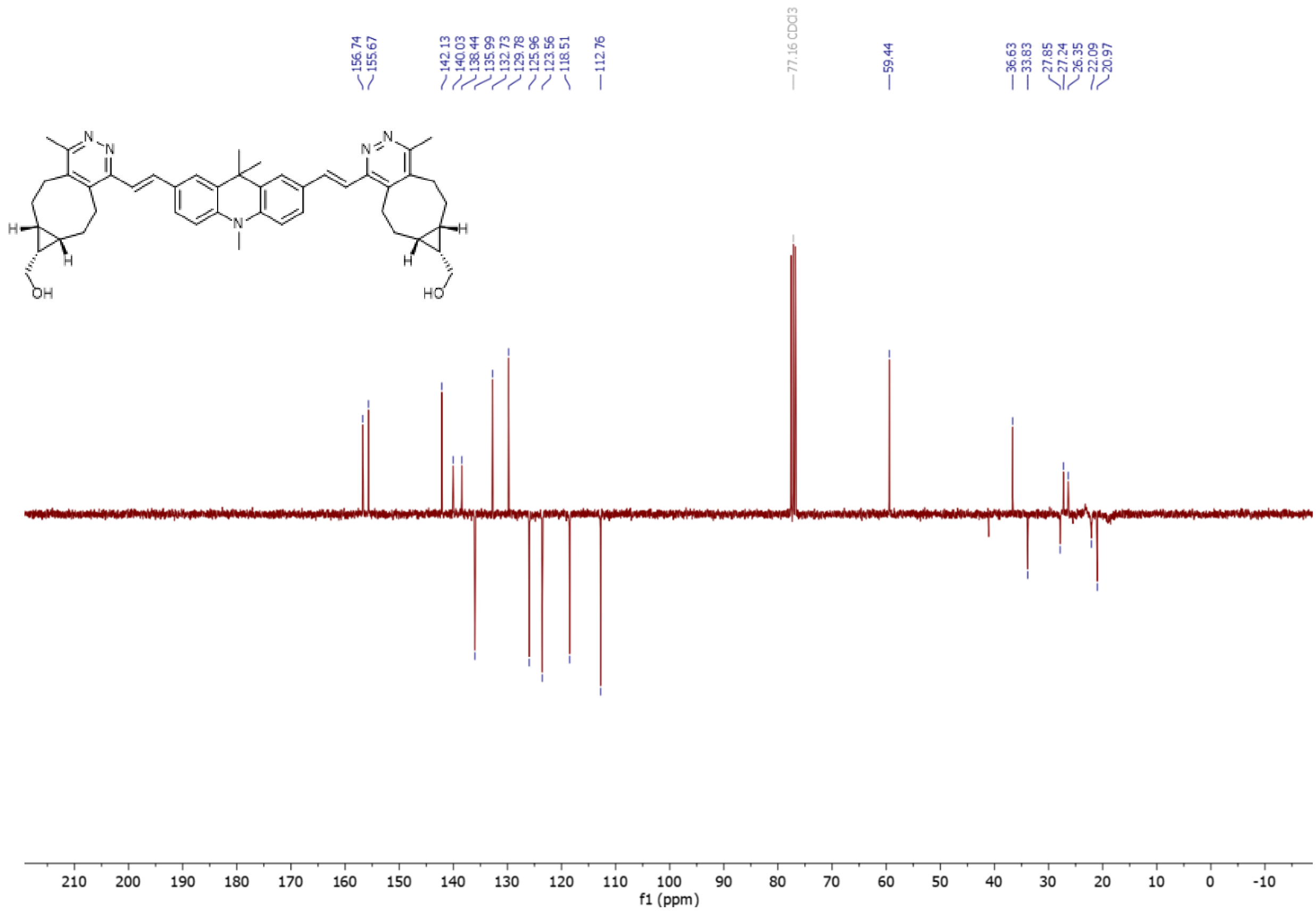

Fig. S10: ^{13}C NMR spectrum of compound [TzDiClick] in $CDCl_3$ (75 MHz).

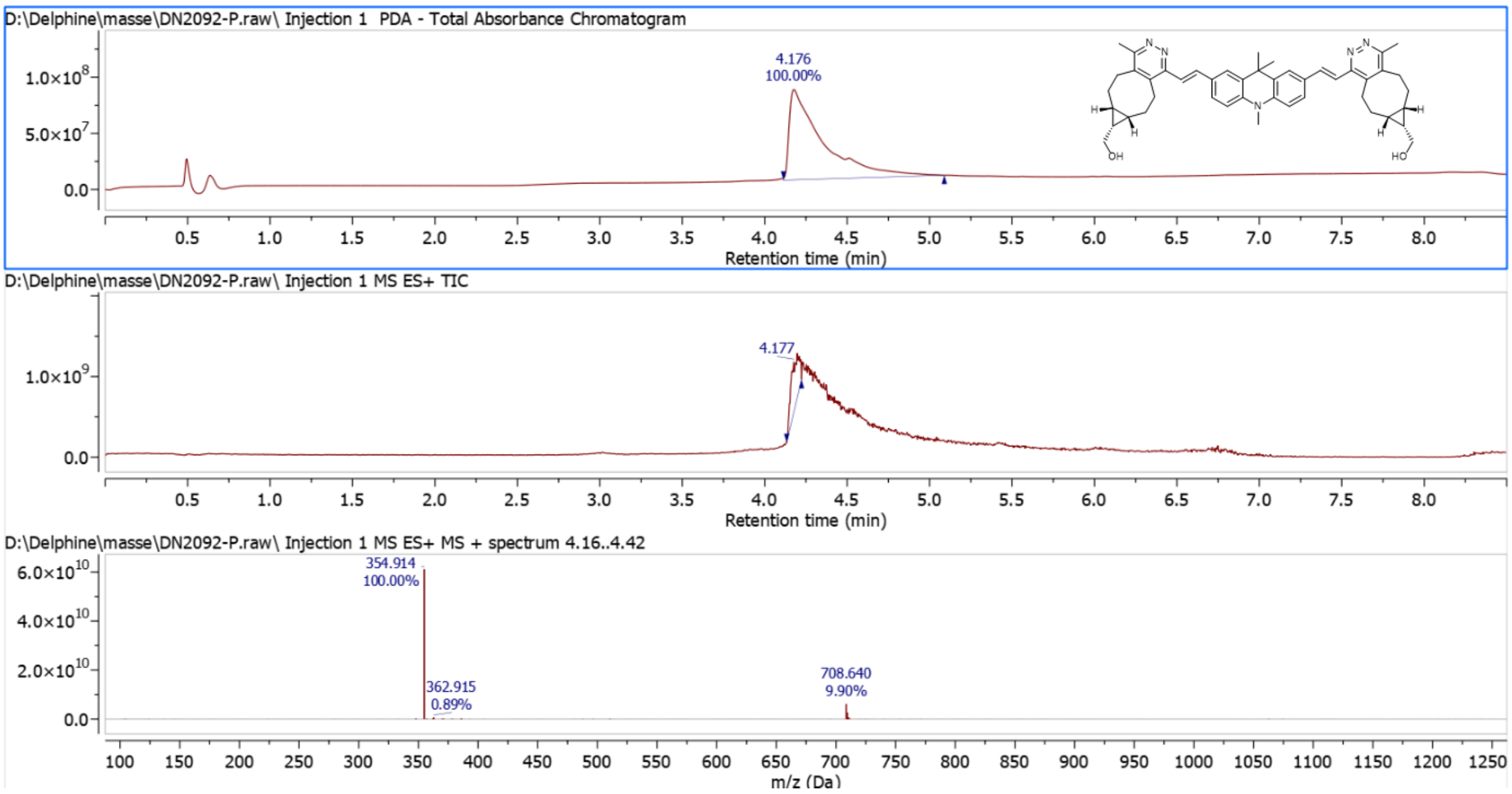

Fig. S11: LC-MS analysis of compound [TzDiClick].

Elemental Composition Report **Page 1**

Single Mass Analysis
Tolerance = 5.0 PPM / DBE: min = -1.5, max = 50.0
Element prediction: Off
Number of isotope peaks used for i-FIT = 9

Monoisotopic Mass, Even Electron Ions
943 formula(e) evaluated with 4 results within limits (up to 50 best isotopic matches for each mass)
Elements Used:
C: 1-100 H: 1-120 N: 0-10 O: 0-10
DN2092 26 (0.175) AM2 (Ar,30000.0,556.28,0.00,LS 10); ABS; Cm (25:29)
1: TOF MS ES+

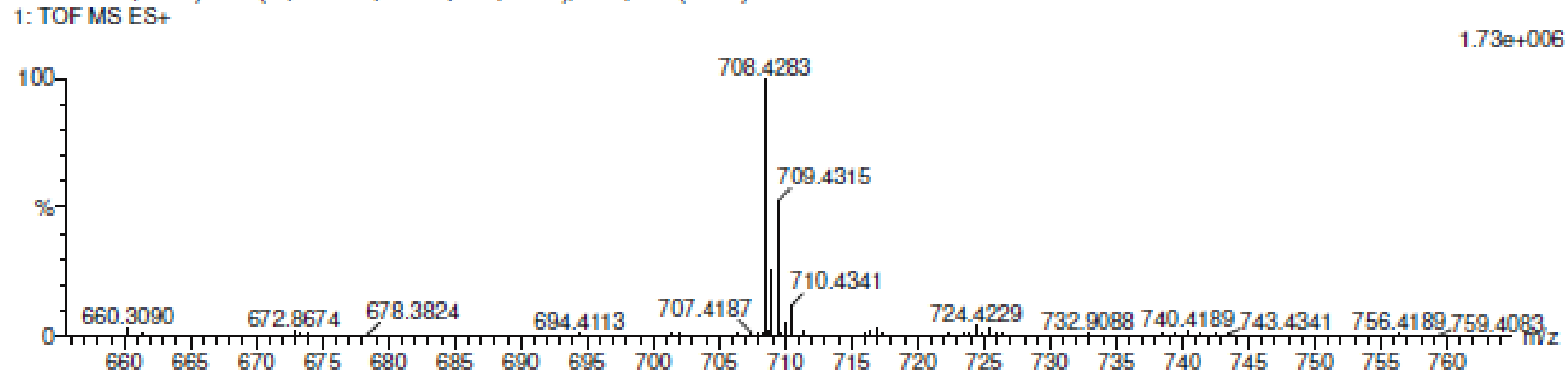

Minimum:				-1.5				
Maximum:		5.0	5.0	50.0				
Mass	Calc. Mass	mDa	PPM	DBE	i-FIT	Norm	Conf(%)	Formula
708.4283	708.4278	0.5	0.7	22.5	1339.5	0.707	49.31	C46 H54 N5 O2
	708.4296	-1.3	-1.8	9.5	1340.3	1.501	22.29	C34 H58 N7 O9
	708.4264	1.9	2.7	17.5	1340.4	1.641	19.38	C45 H58 N O6
	708.4318	-3.5	-4.9	26.5	1341.2	2.407	9.01	C51 H54 N3

Fig. S12: HR mass spectrum (ESI, positive mode (left)) of compound [TzDiClick].

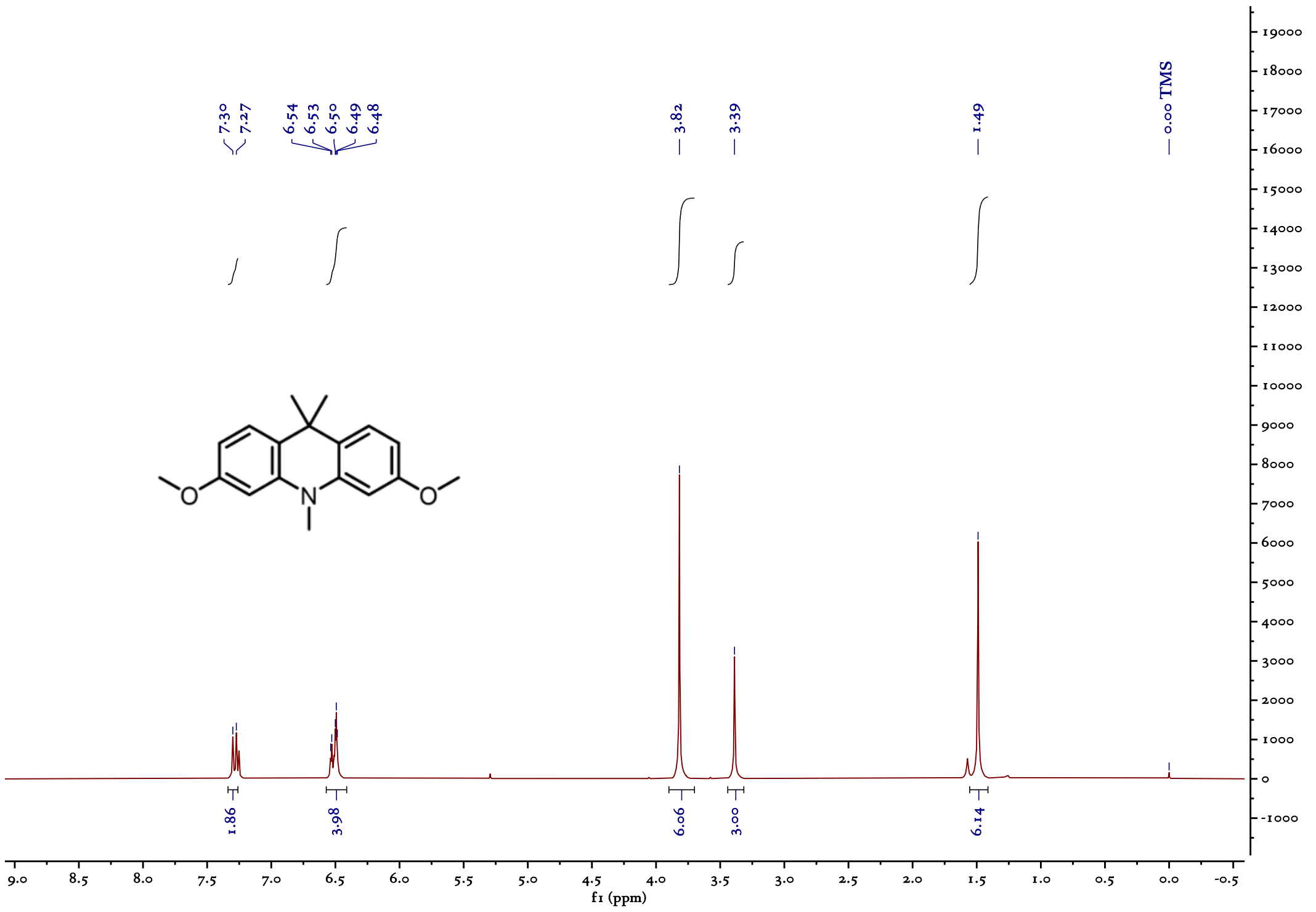

Fig. S13: ^{1}H NMR spectrum of compound [7] in $CDCl_3$ (300 MHz).

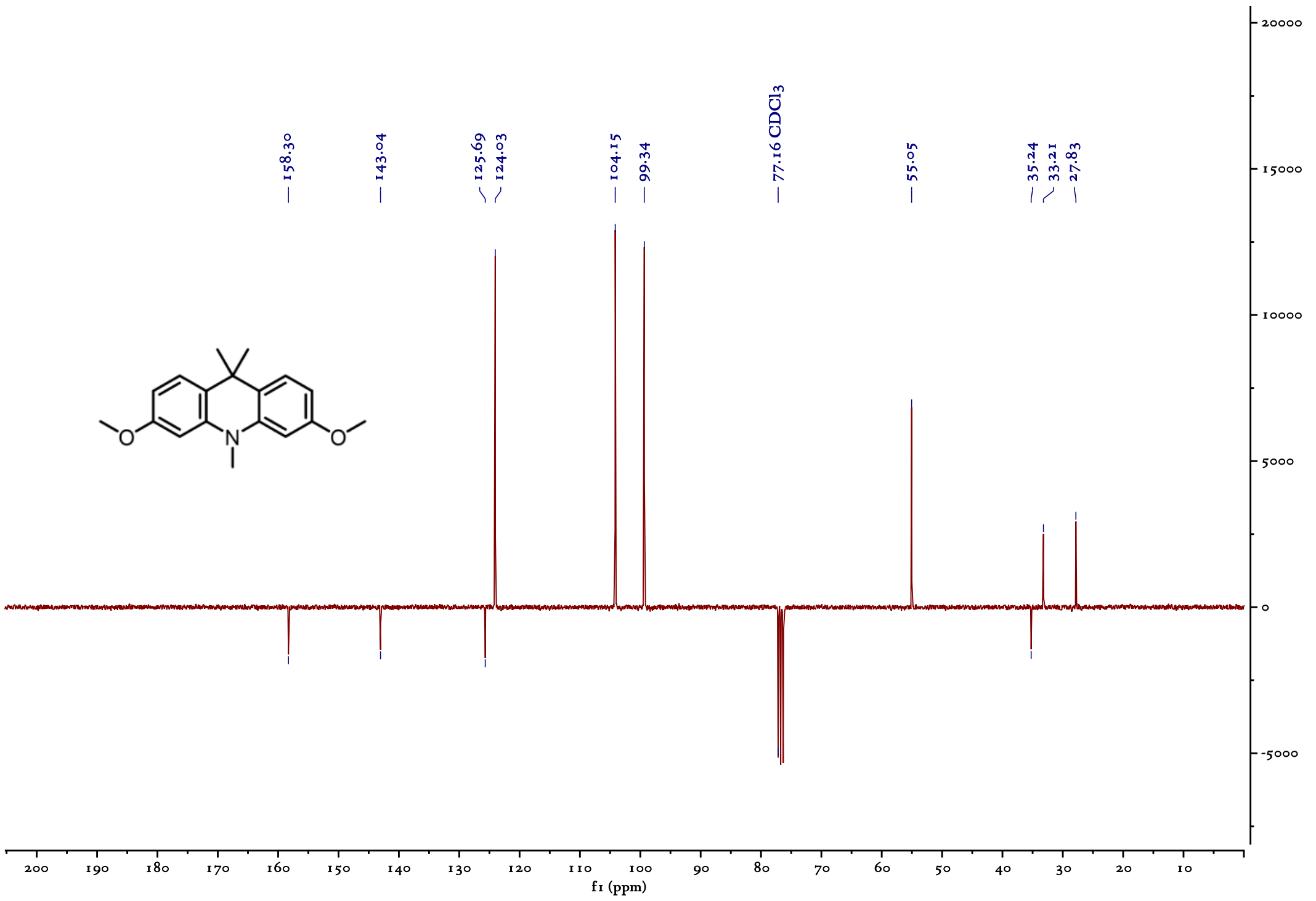

Fig. S14: ^{13}C NMR spectrum of compound [7] in $CDCl_3$ (75 MHz).

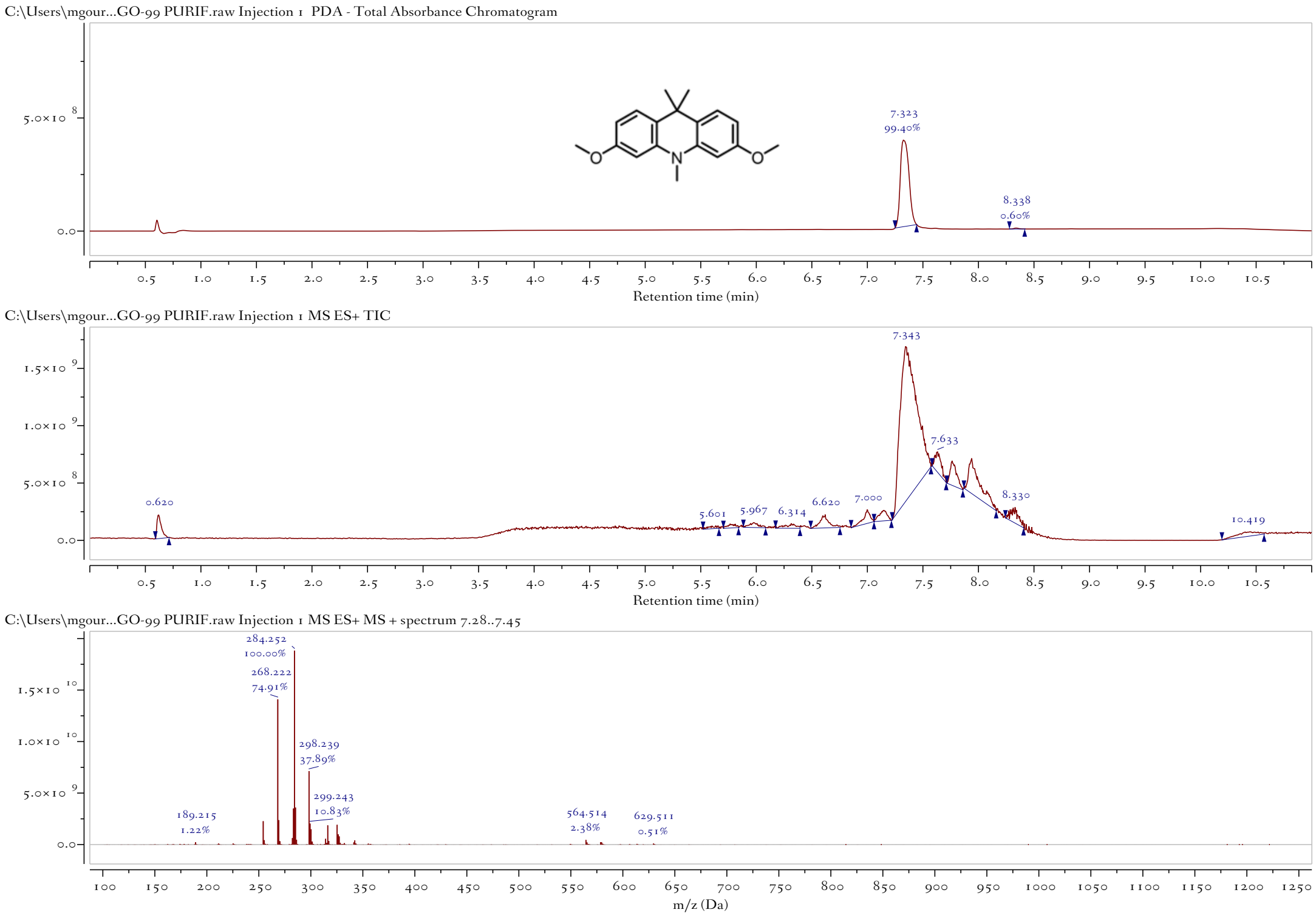

Fig. S15: LC-MS analysis of compound [7] (Analytical system): from up to bottom: RP-HPLC elution profile, TIC profile and LR mass spectrum (ESI, positive mode).

Elemental Composition Report **Page 1**

Single Mass Analysis
Tolerance = 5.0 PPM / DBE: min = -1.5, max = 50.0
Element prediction: Off
Number of isotope peaks used for i-FIT = 9

Monoisotopic Mass, Even Electron Ions
327 formula(e) evaluated with 1 results within limits (up to 50 best isotopic matches for each mass)
Elements Used:
C: 1-100 H: 1-120 N: 0-10 O: 0-10
MGO-121 28 (0.193) AM2 (Ar,30000.0,556.28,0.00,LS 10); ABS; Cm (26:30)
1: TOF MS ES+

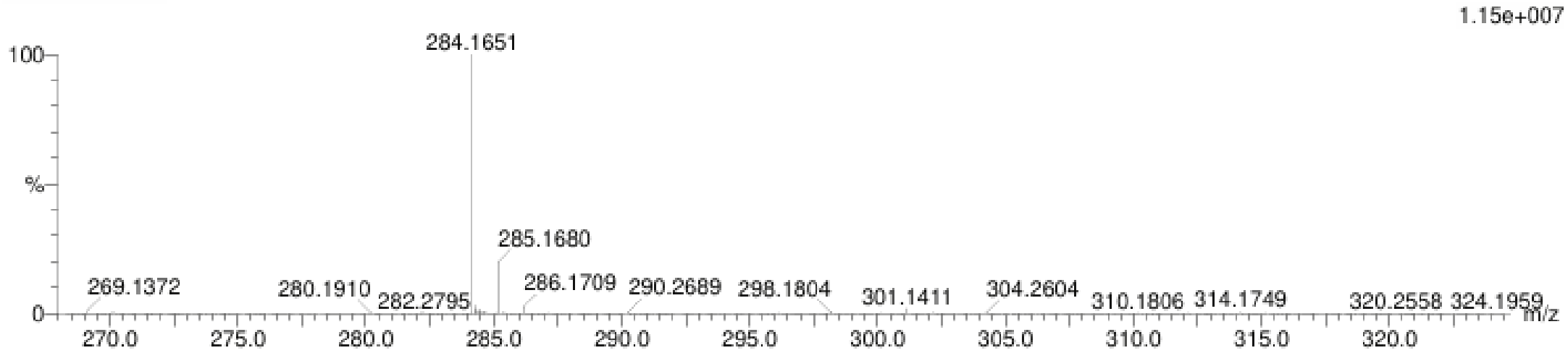

Minimum:				-1.5				
Maximum:		5.0	5.0	50.0				
Mass	Calc. Mass	mDa	PPM	DBE	i-FIT	Norm	Conf(%)	Formula
284.1651	284.1651	0.0	0.0	8.5	2574.2	n/a	n/a	C18 H22 N O2

Fig. S16: HR mass spectrum (ESI, positive mode (left)) of compound [7].

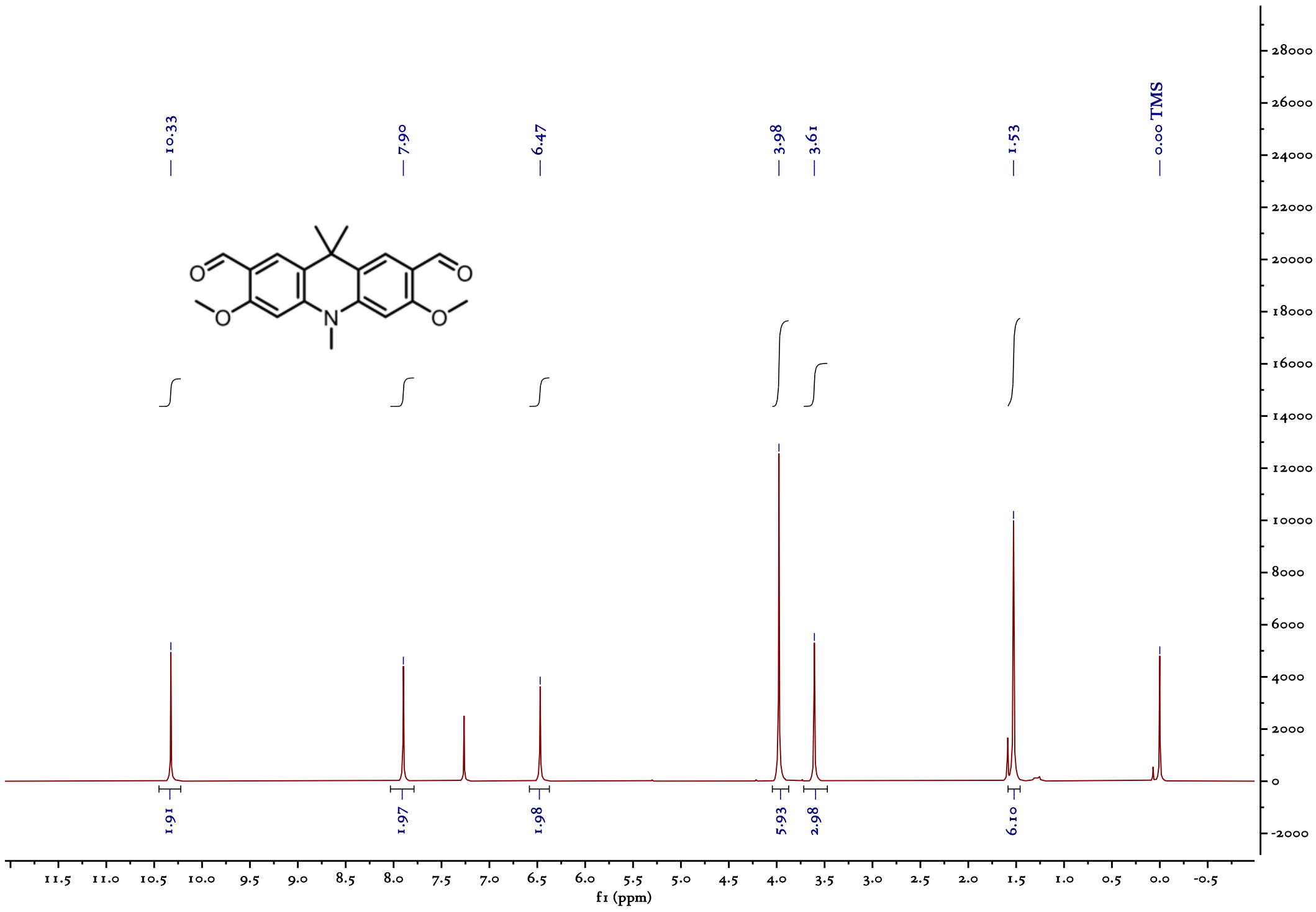

Fig. S17: ^{1}H NMR spectrum of compound [8] in $CDCl_3$ (300 MHz).

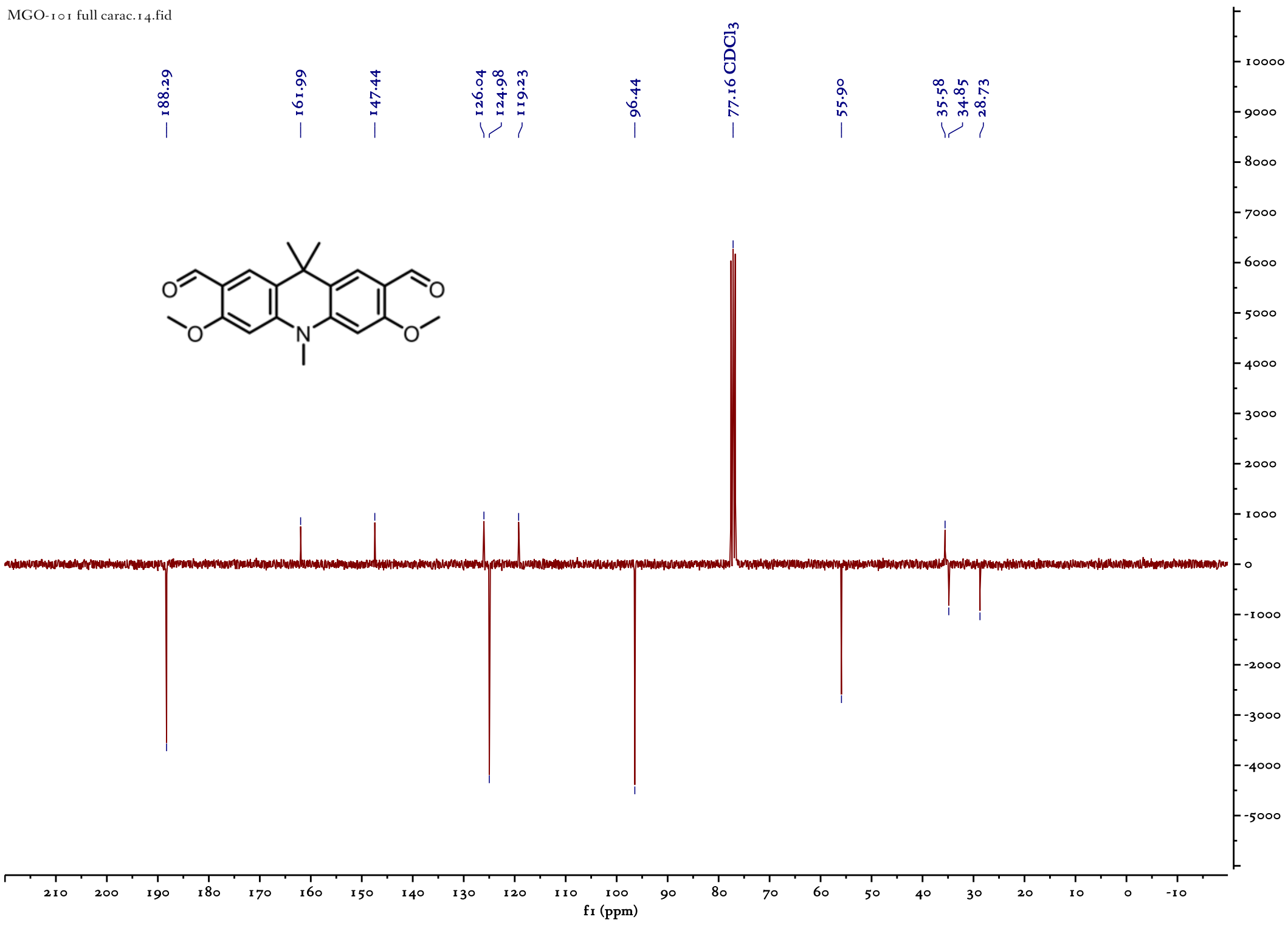

Fig. S18: ^{13}C NMR spectrum of compound [8] in $CDCl_3$ (75 MHz).

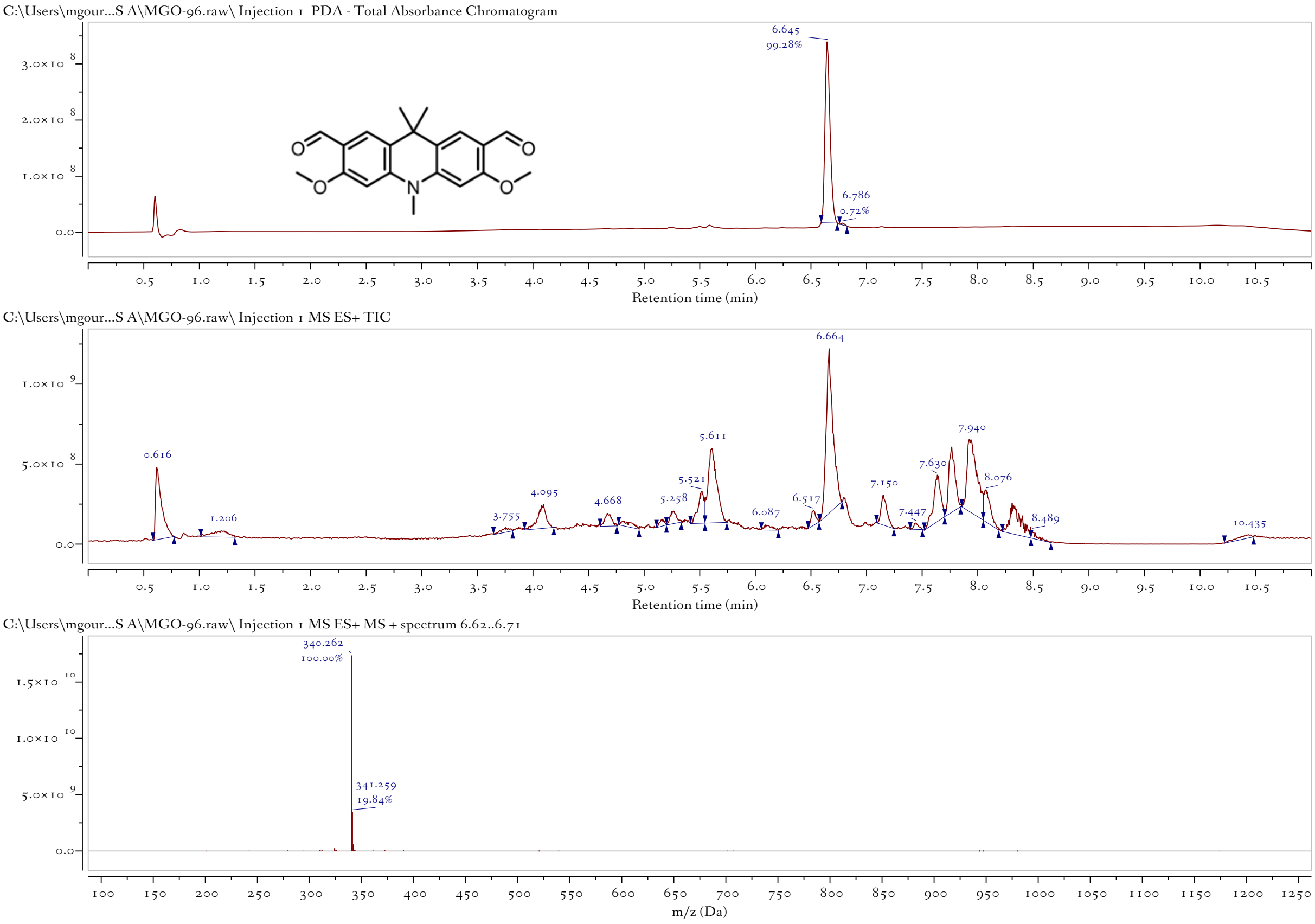

Fig. S19: LC-MS analys of compound [8] (Analytical system): from up to bottom: RP-HPLC elution profile, TIC profil and LR mass spectrum (ESI, positive mode).

Elemental Composition Report **Page 1**

Single Mass Analysis
Tolerance = 5.0 PPM / DBE: min = -1.5, max = 100.0
Element prediction: Off
Number of isotope peaks used for i-FIT = 9

Monoisotopic Mass, Even Electron Ions
679 formula(e) evaluated with 3 results within limits (all results (up to 1000) for each mass)
Elements Used:
C: 0-130 H: 0-150 N: 0-15 O: 0-15
MGO-101 11 (0.167) AM2 (Ar,30000.0,556.28,1.00,LS 10); ABS; Cm (9:21)
1: TOF MS ES+

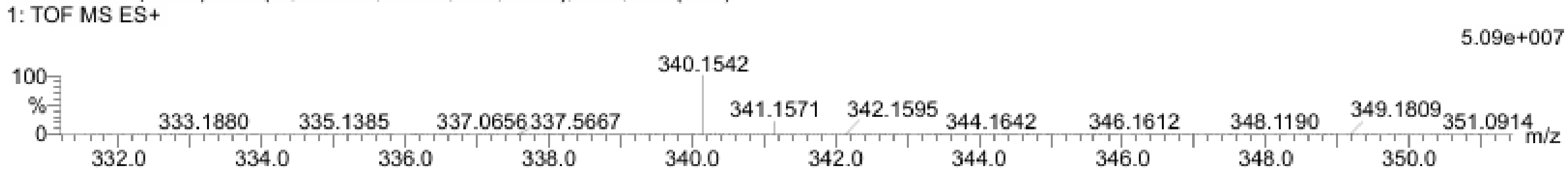

Minimum:				-1.5			
Maximum:		5.0	5.0	100.0			
Mass	Calc. Mass	mDa	PPM	DBE	i-FIT	i-FIT (Norm)	Formula
340.1542	340.1549	-0.7	-2.1	10.5	2391.0	0.0	C20 H22 N O4
	340.1554	-1.2	-3.5	3.5	2409.7	18.6	C5 H18 N13 O5
	340.1540	0.2	0.6	-1.5	2411.2	20.1	C4 H22 N9 O9

Fig. S20: HR mass spectrum (ESI, positive mode (left)) of compound [8]

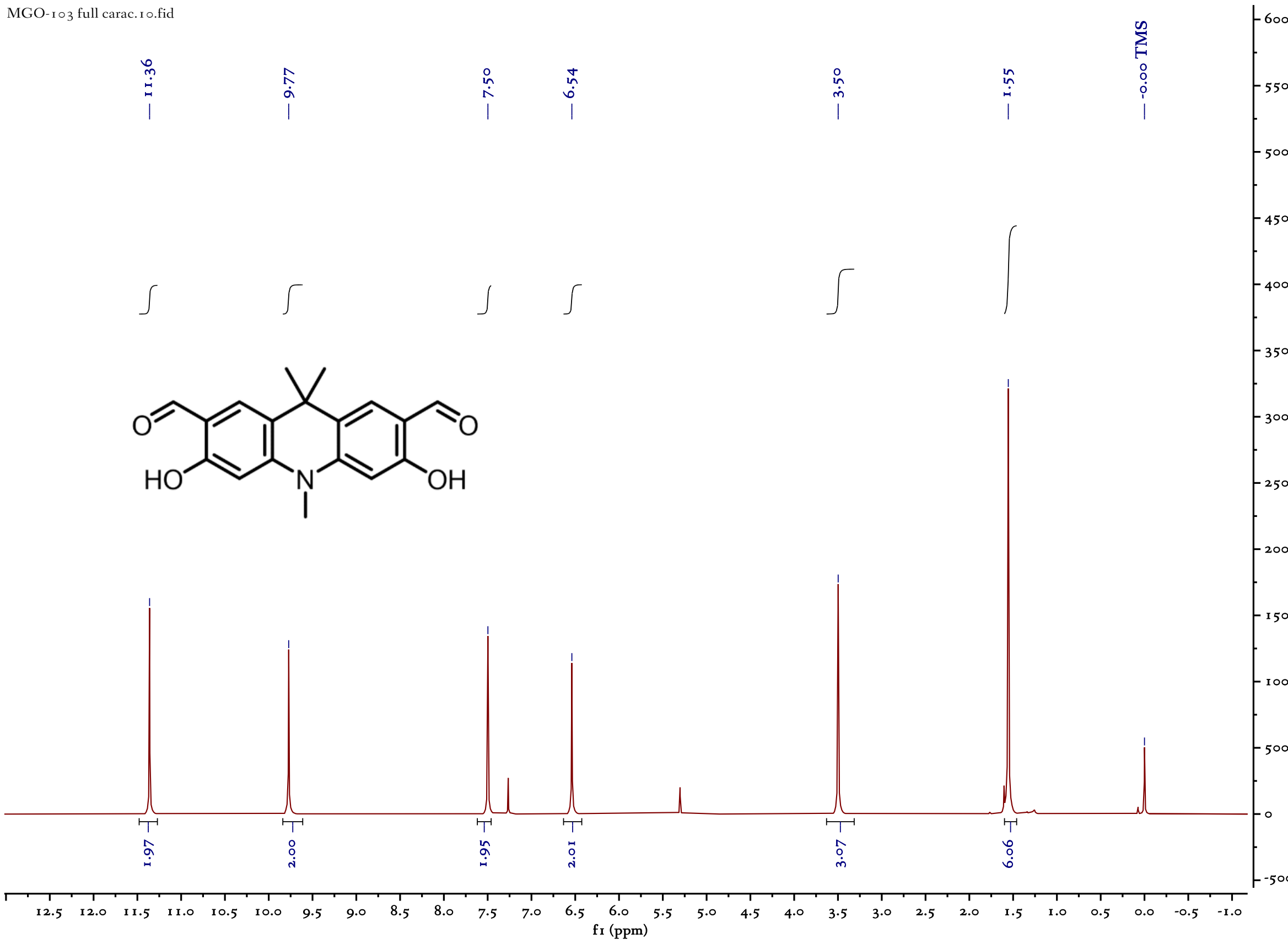

Fig. S21: ¹H NMR spectrum of compound [9] in $CDCl_3$ (300 MHz).

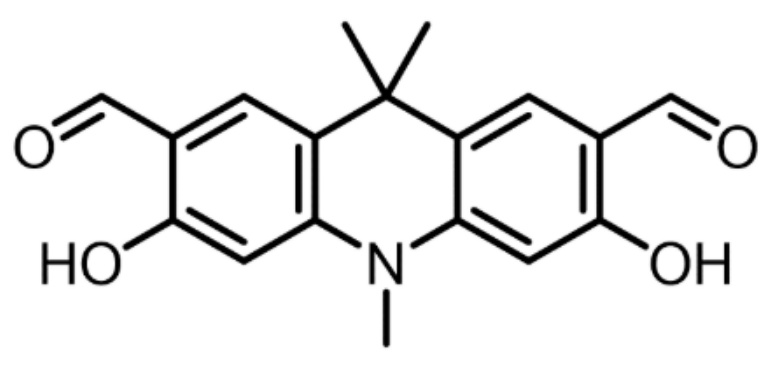

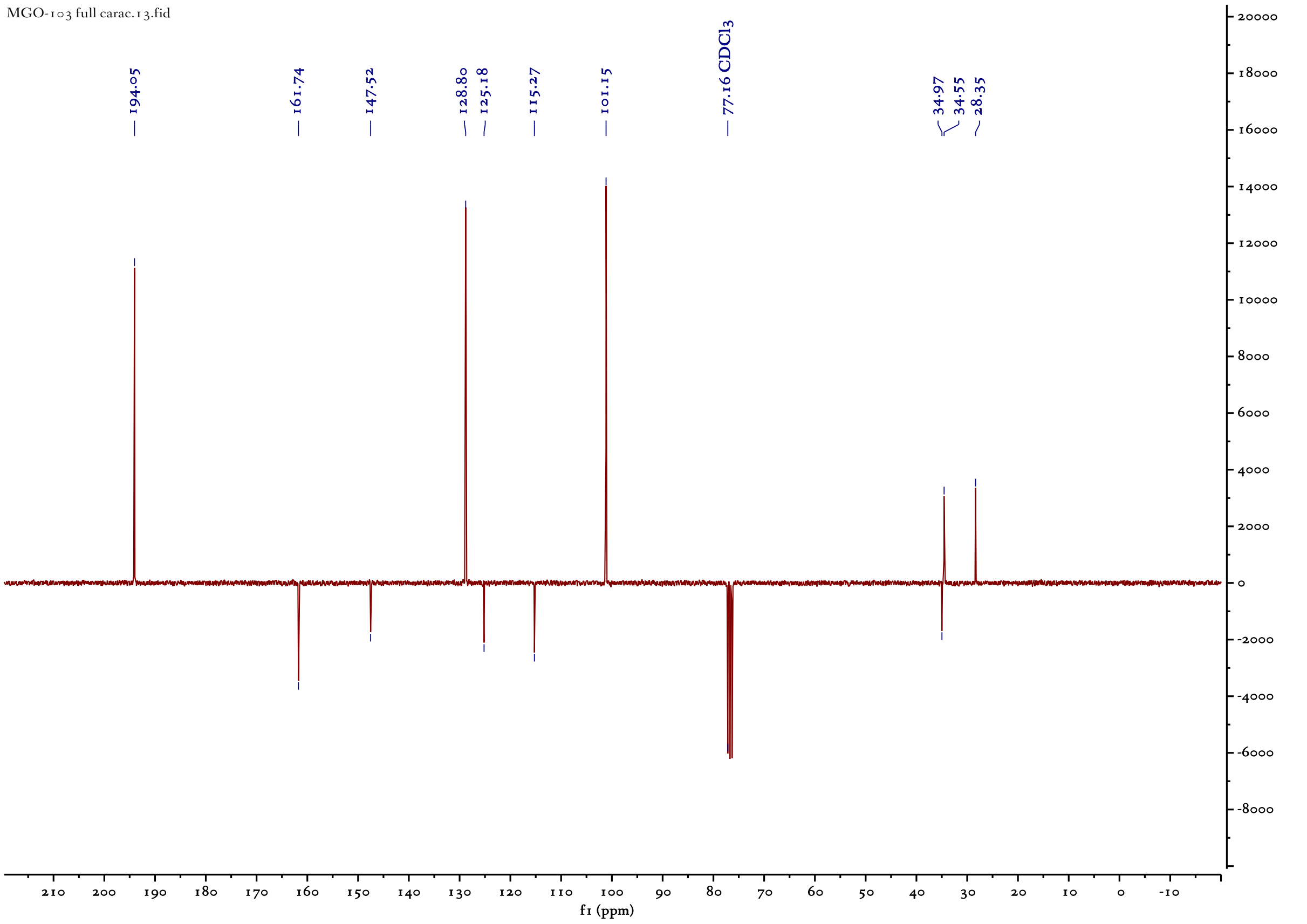

Fig. S22: ^{13}C NMR spectrum of compound [9] in $CDCl_3$ (75 MHz).

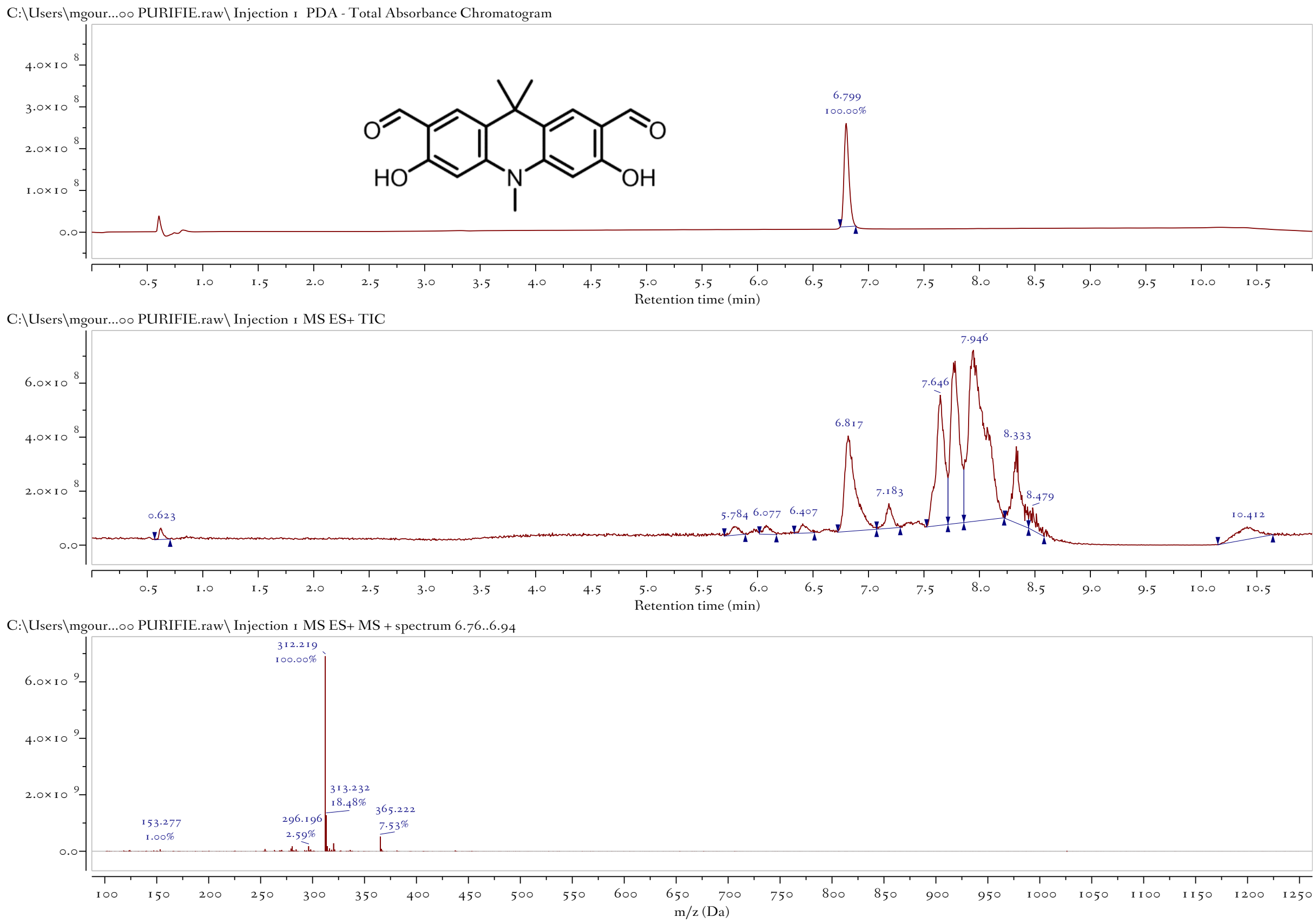

Fig. S23: LC-MS analysis of compound [9] (Analytical system): from up to bottom: RP-HPLC elution profile, TIC profile and LR mass spectrum (ESI, positive mode).

Elemental Composition Report **Page 1**

Single Mass Analysis
Tolerance = 5.0 PPM / DBE: min = -1.5, max = 100.0
Element prediction: Off
Number of isotope peaks used for i-FIT = 9

Monoisotopic Mass, Even Electron Ions
581 formula(e) evaluated with 3 results within limits (all results (up to 1000) for each mass)
Elements Used:
C: 0-130 H: 0-150 N: 0-15 O: 0-15
MGO-103 9 (0.158) AM2 (Ar,30000.0,556.28,1.00,LS 10); ABS; Cm (7:17)
1: TOF MS ES+

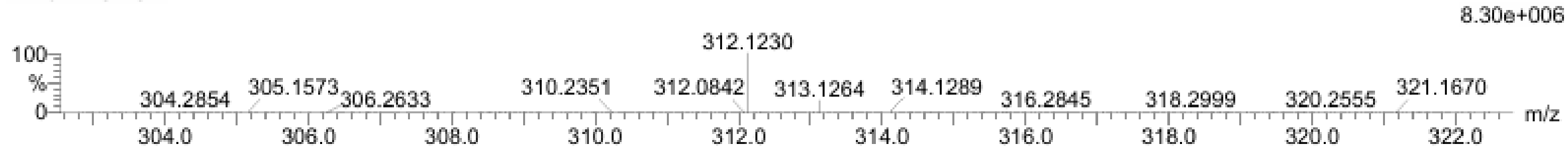

Minimum:				-1.5			
Maximum:		5.0	5.0	100.0			
Mass	Calc. Mass	mDa	PPM	DBE	i-FIT	i-FIT (Norm)	Formula
312.1230	312.1236	-0.6	-1.9	10.5	2152.1	0.0	C18 H18 N O4
	312.1241	-1.1	-3.5	3.5	2169.0	17.0	C3 H14 N13 O5
	312.1227	0.3	1.0	-1.5	2170.7	18.6	C2 H18 N9 O9

Fig. S24: HR mass spectrum (ESI, positive mode (left)) of compound [9].

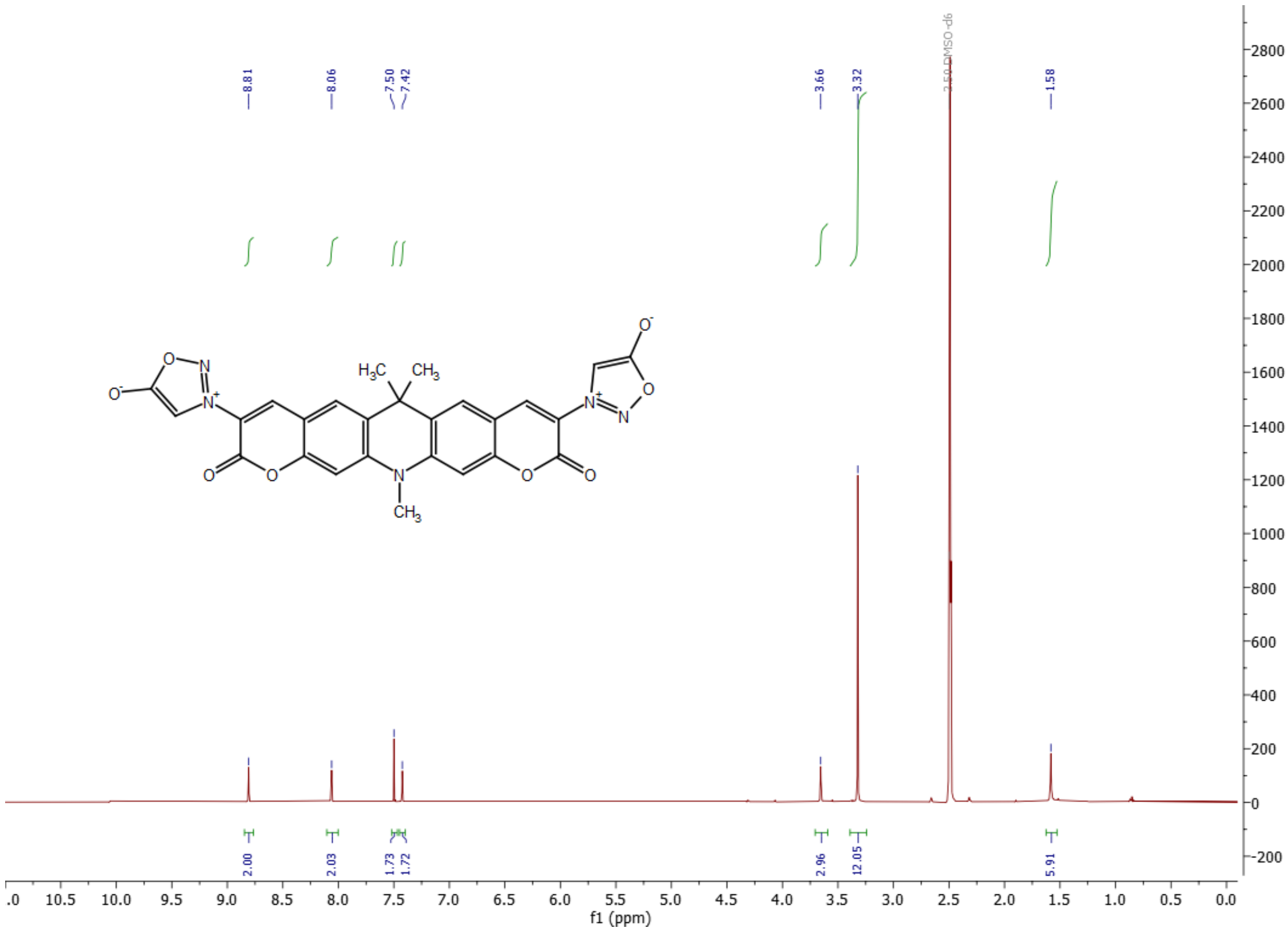

Fig. S25: ¹H NMR spectrum of compound [SydProbe] in DMSO-d₆ (400 MHz).

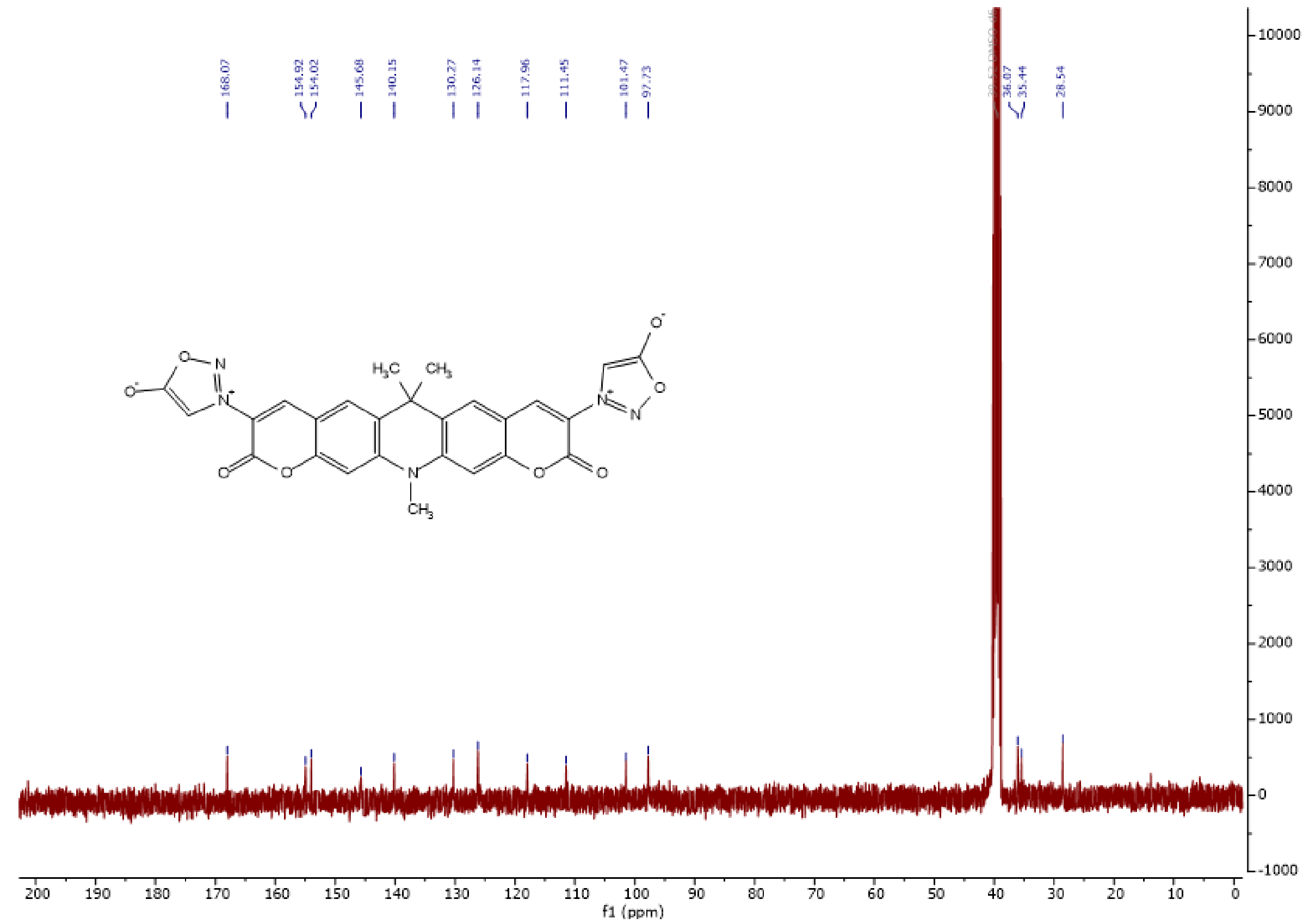

Fig. S26: ^{13}C NMR spectrum of compound [SydProbe] in DMSO-d_6 (400 MHz).

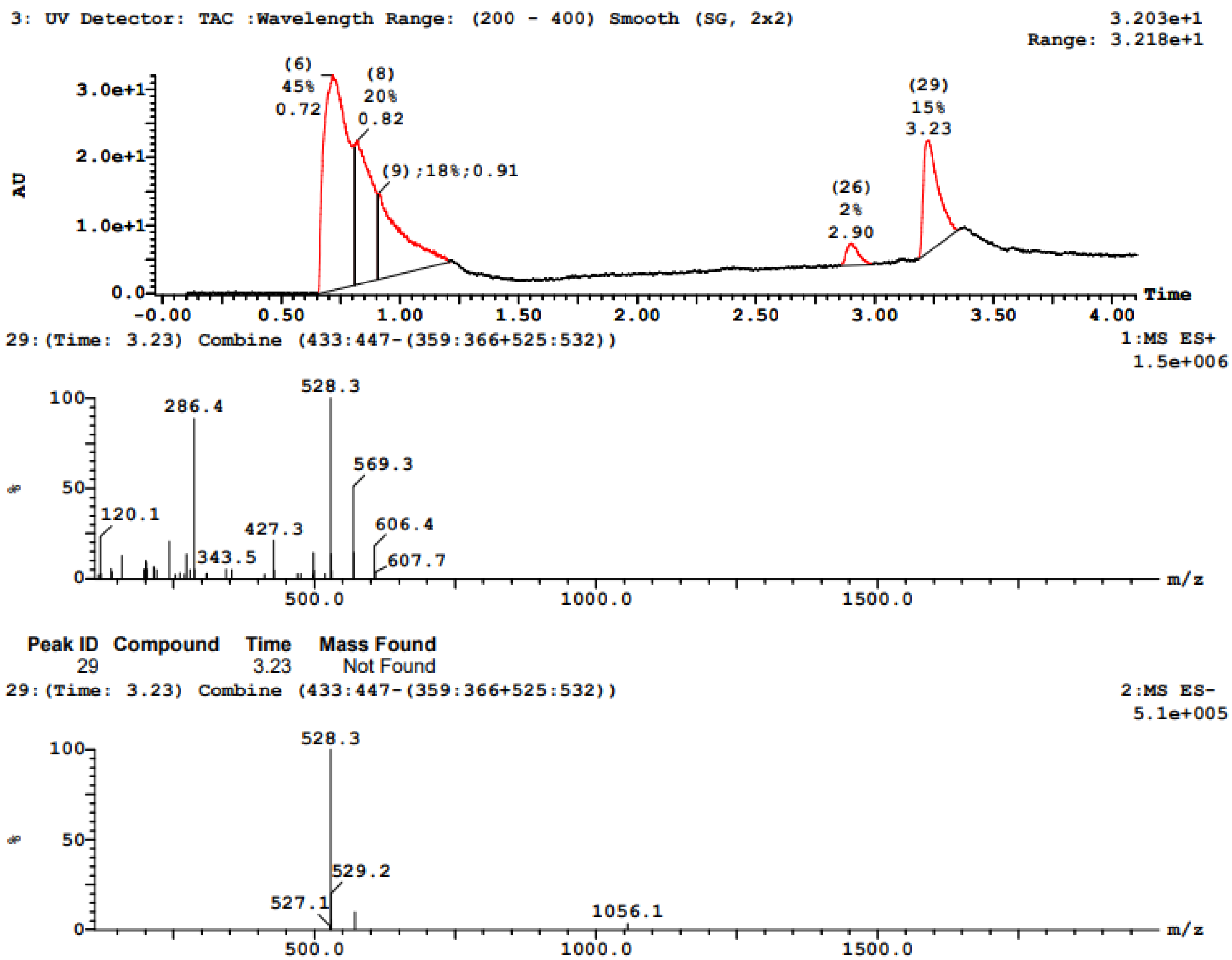

Fig. S27: LC-MS analysis of compound [SydProbe].

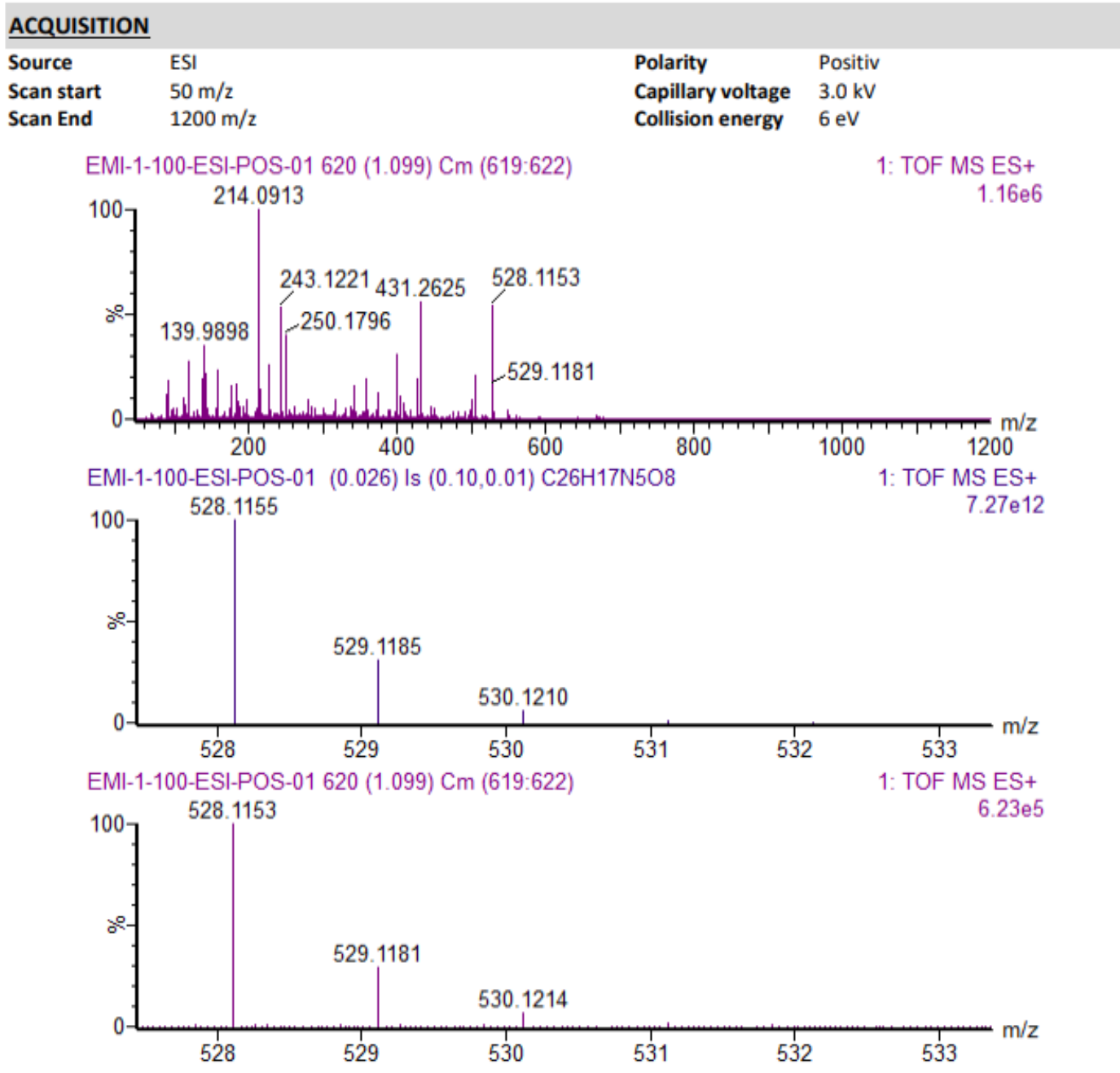

measured m/z	z	Formula	th. m/z	error [mDa]	error [ppm]
528.1153	1	C26H18N5O8	528.1155	0.2	0.4

HRMS (ESI-TOF) m/z calcd for C26H18N5O8 [M+H]+ 528.1155; found 528.1153

Fig. S28: HR mass spectrum (ESI, positive mode) of compound SydProbe.

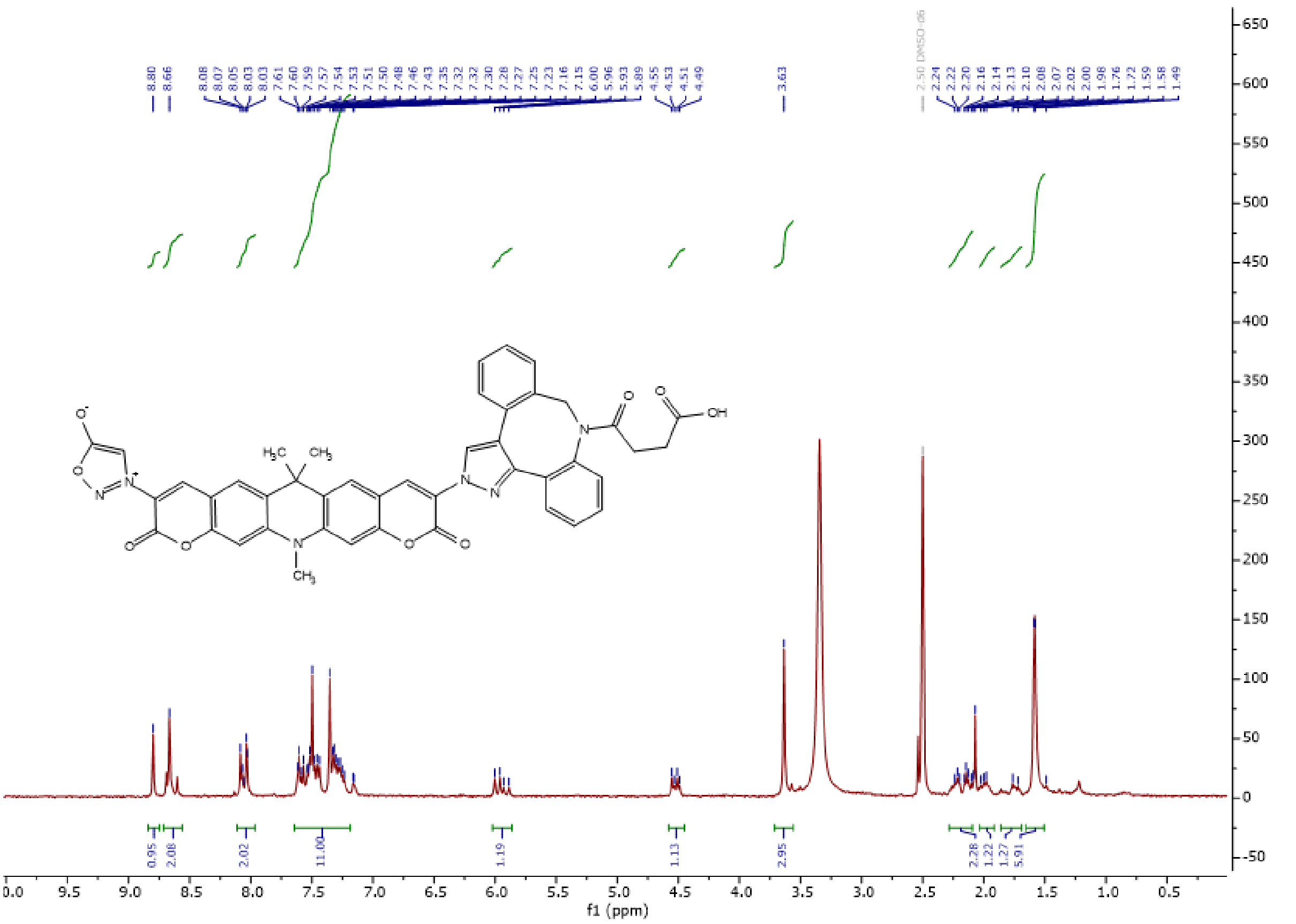

Fig. S29: ^{1}H NMR spectrum of compound [SydMonoClick] in DMSO-d_6, (400 MHz).

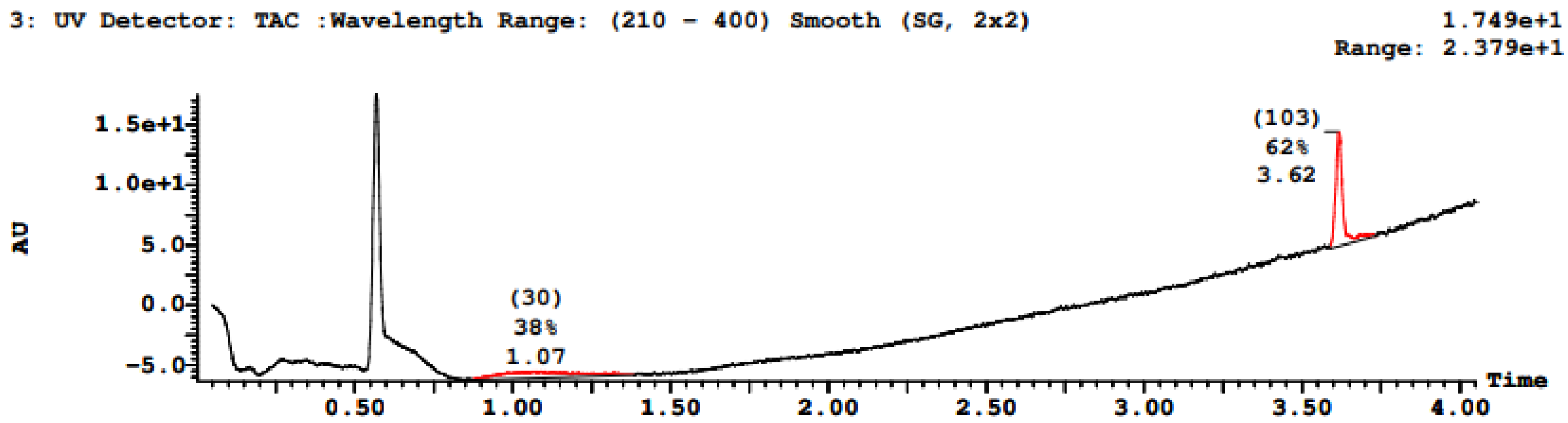

Peak Number	Compound	Time	AreaAbs	Area %Total	Width	Height	Mass Found
30		1.07	1.49e+005	37.58	1	6e+005	Not Found
103		3.62	2.47e+005	62.42	0	9e+006	Not Found

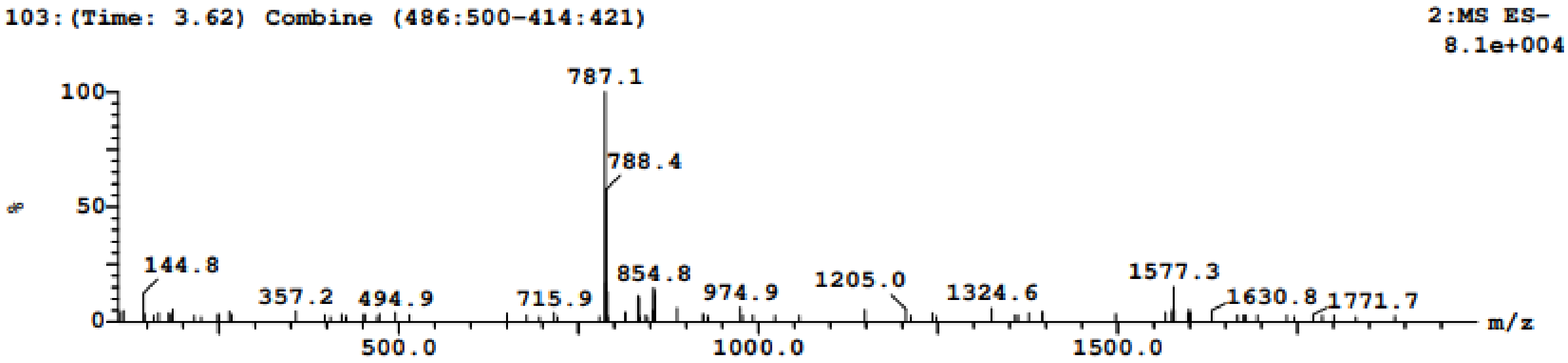

Fig. S30: LC-MS analysis of compound [SydMonoClick].

ACQUISITION

**Source**	ESI	**Polarity**	Positiv
**Scan start**	50 m/z	**Capillary voltage**	3.0 kV
**Scan End**	1200 m/z	**Collision energy**	6 eV

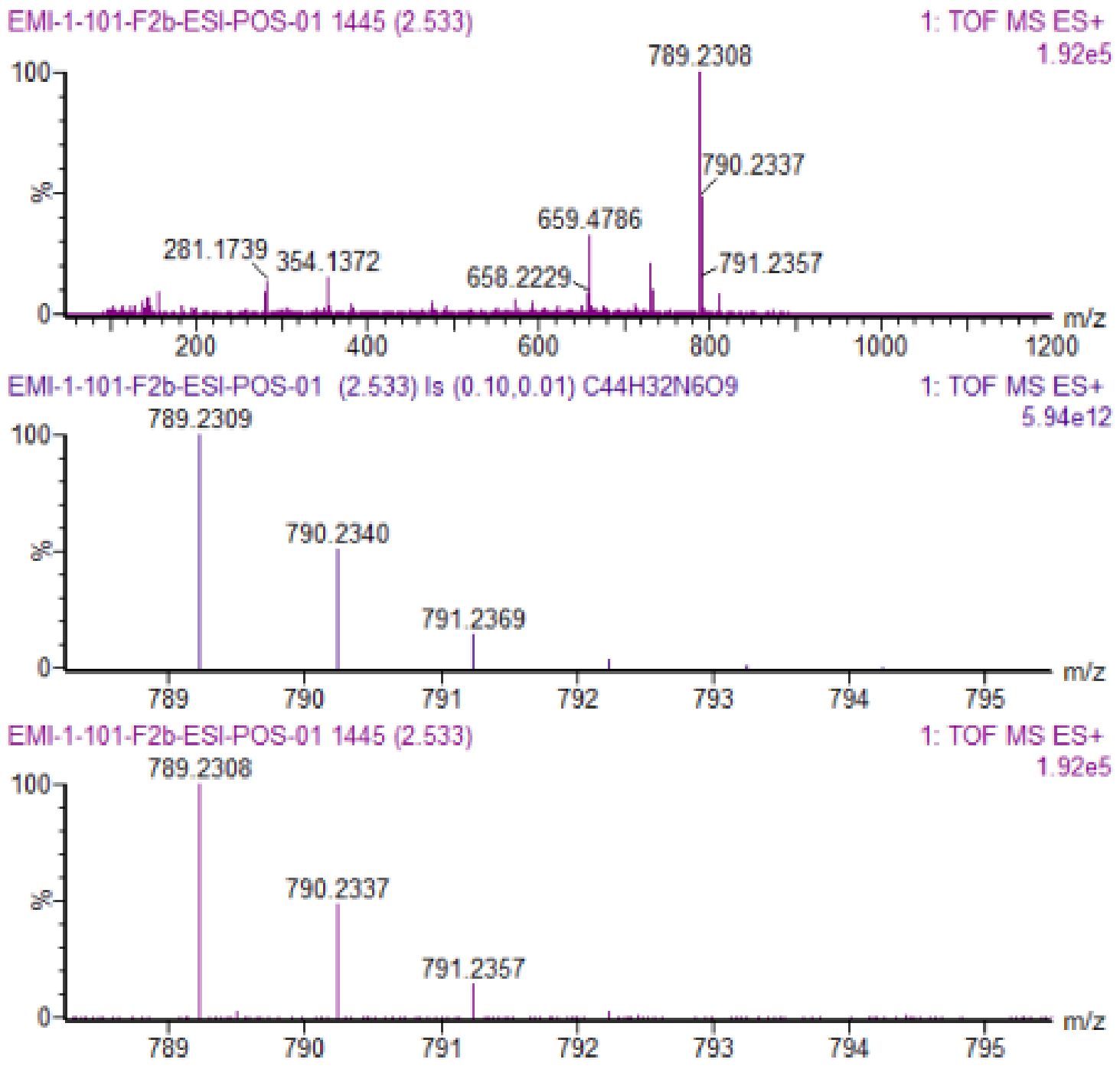

measured m/z	z	Formula	th. m/z	error [mDa]	error [ppm]
789.2308	1	$C_{44}H_{33}N_6O_9$	789.2309	0.1	0.1

HRMS (ESI-TOF) m/z calcd for $C_{44}H_{33}N_6O_9$ $[M+H]^+$ 789.2309; found 789.2308

Fig. S31: HR mass spectrum (ESI, positive mode) of compound SydMonoClick.

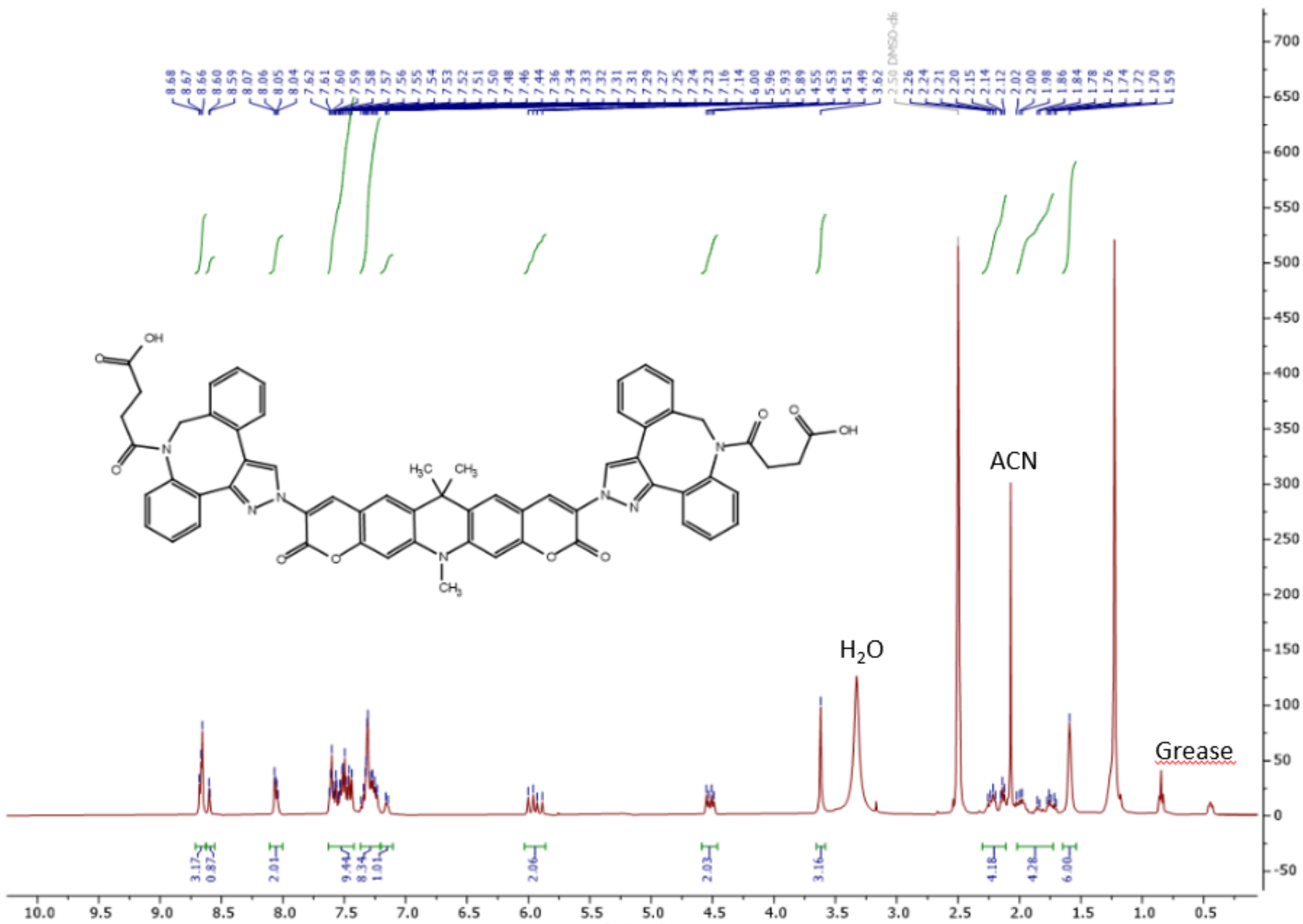

Fig. S32: ^{1}H NMR spectrum of compound [SydDiClick] in DMSO-d_6 (400 MHz).

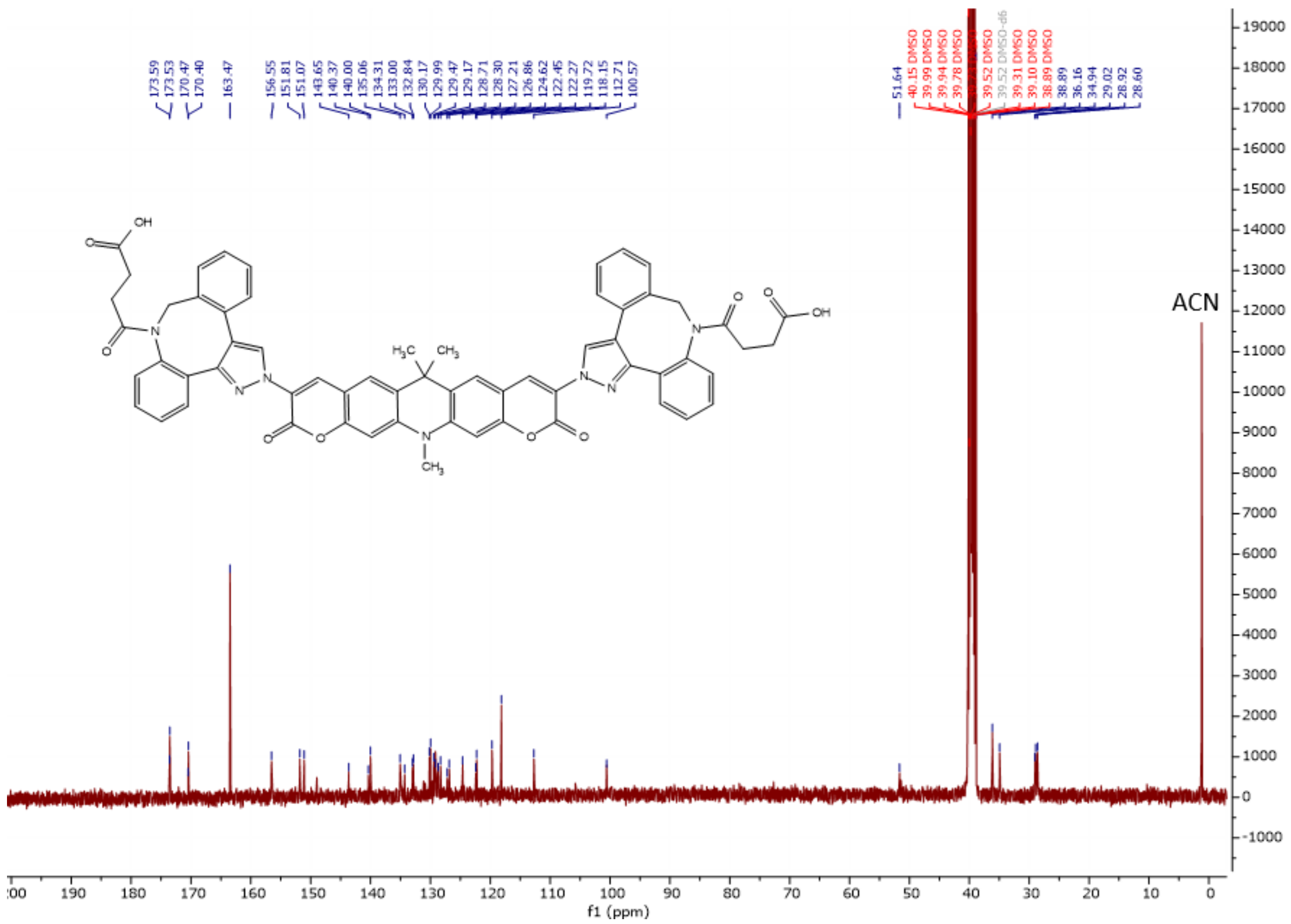

Fig. S33: [13]*C NMR spectrum of compound [SydDiClick] in DMSO-d$_6$ (400 MHz).*

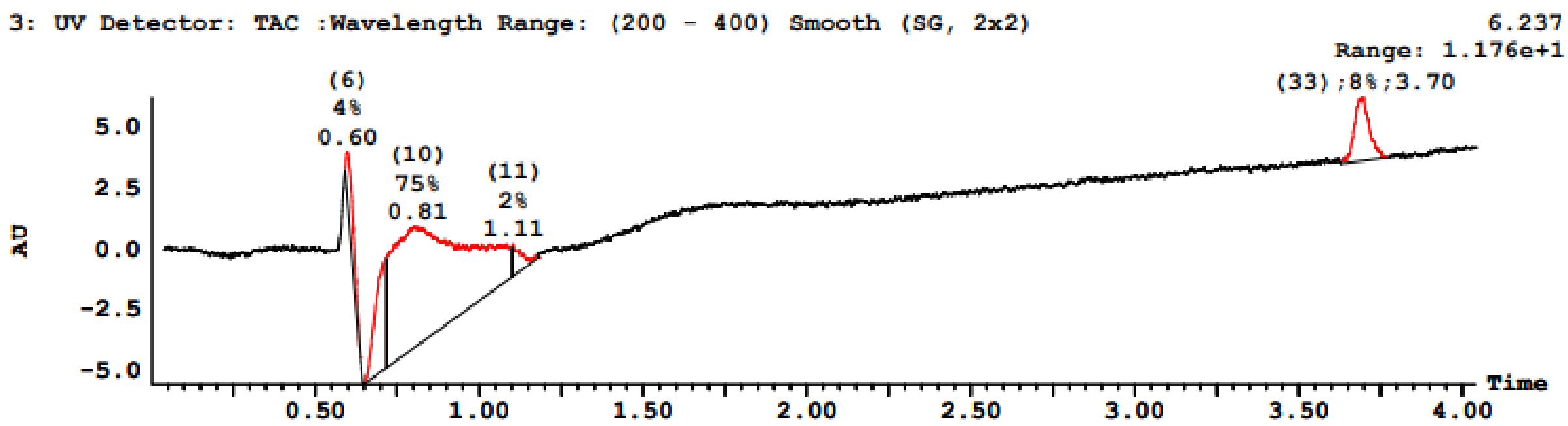

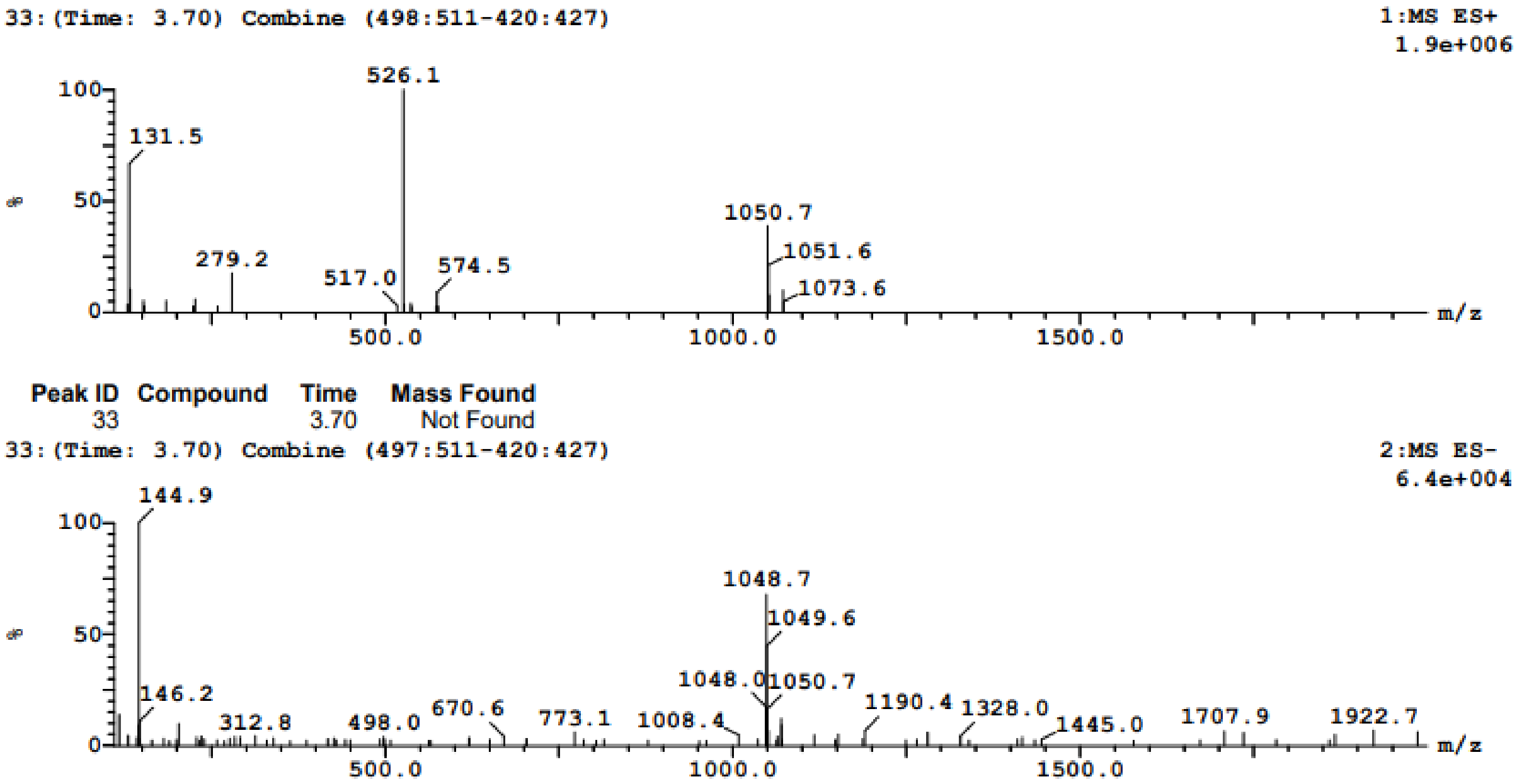

Fig. S34: LC-MS analysis of compound [SydDiClick].

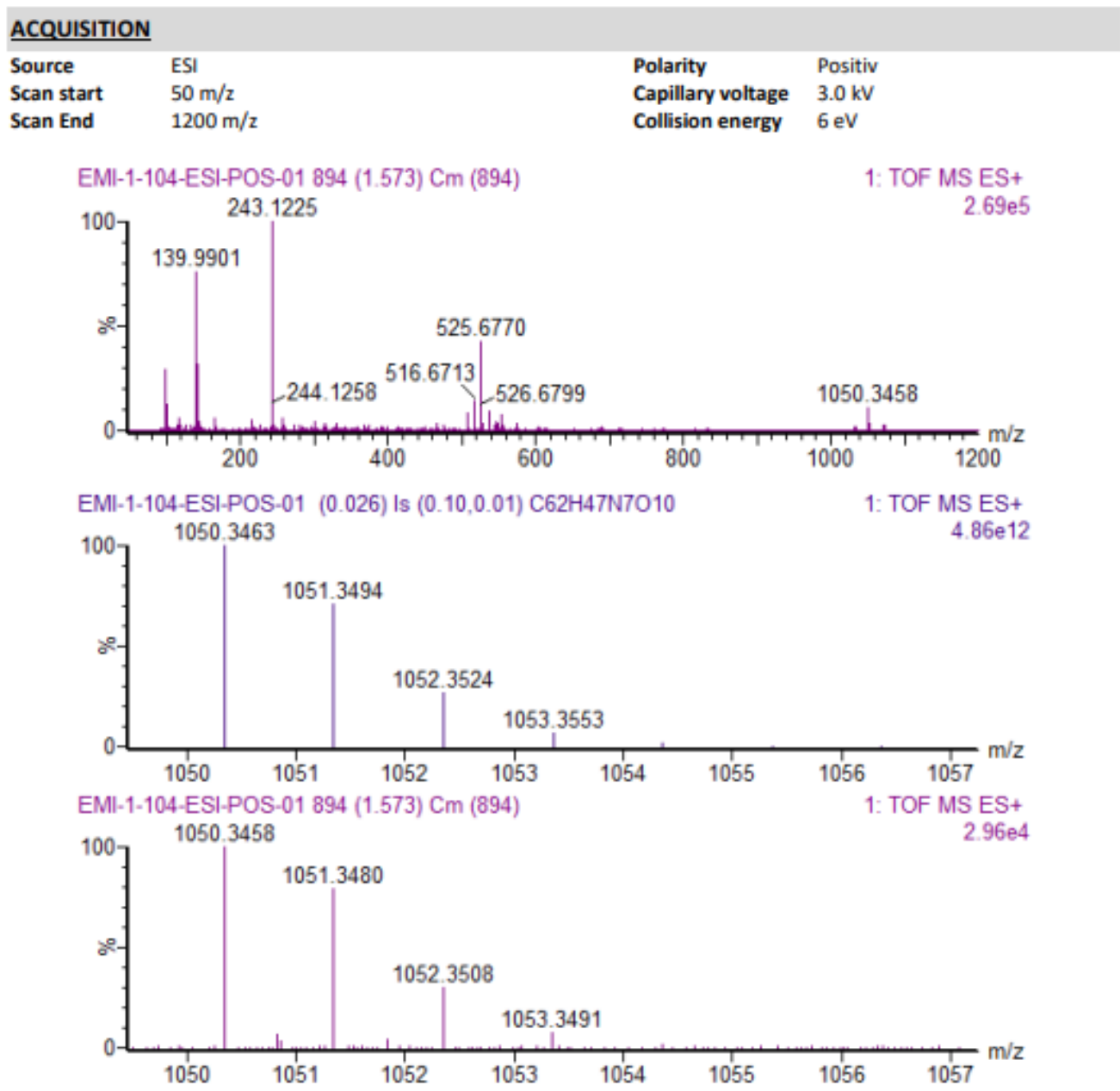

measured m/z	z	Formula	th. m/z	error [mDa]	error [ppm]
1050.3458	1	$C_{62}H_{48}N_7O_{10}$	1050.3463	0.5	0.5

HRMS (ESI-TOF) m/z calcd for $C_{62}H_{48}N_7O_{10}$ $[M+H]^+$ 1050.3463; found 1050.3458

Fig. S35: HR mass spectrum (ESI, positive mode) of compound [SydDiClick].

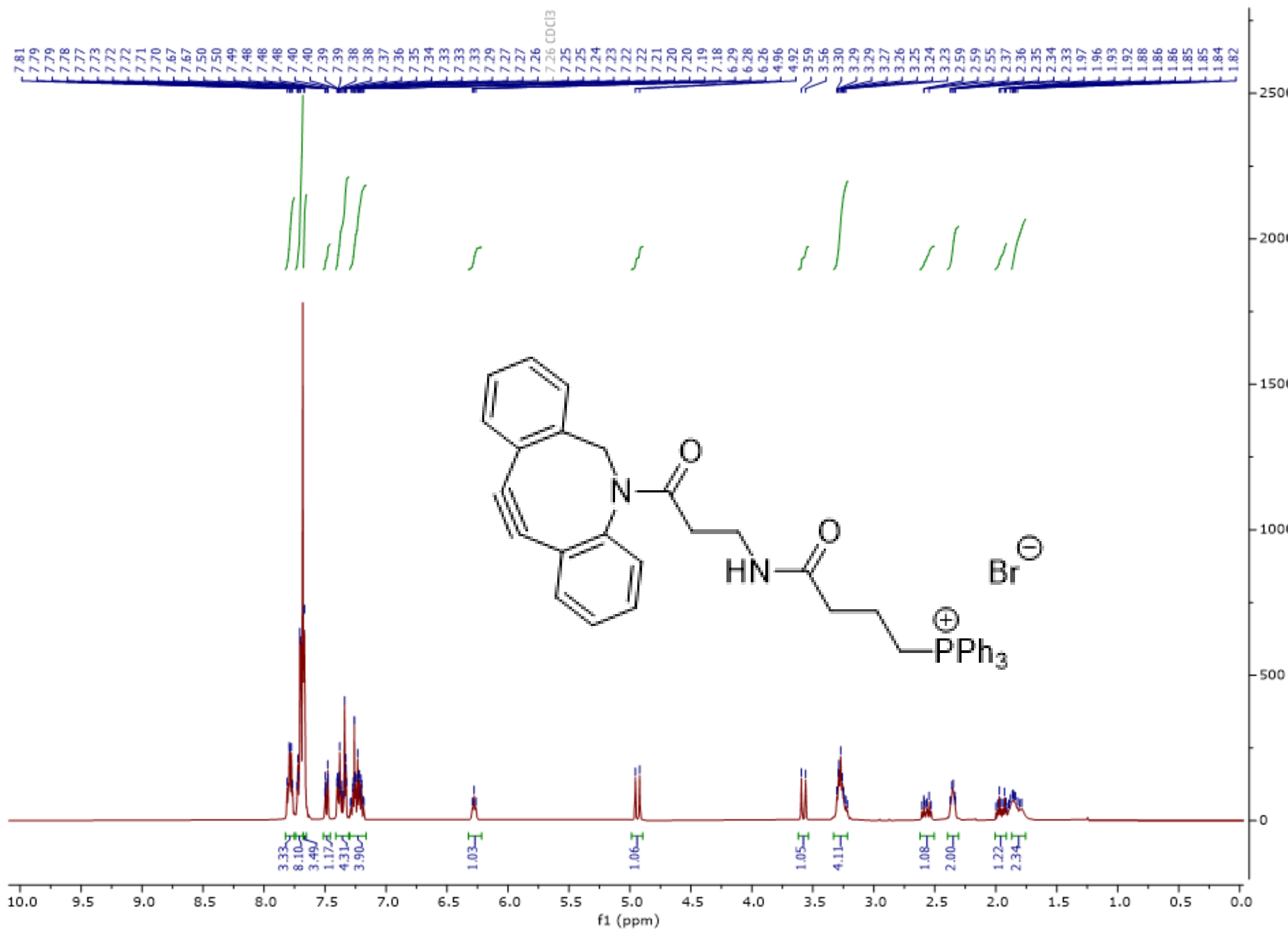

Fig. S36: ¹H NMR spectrum of compound [DBCO-TPP] in $CDCl_3$ *(400 MHz).*

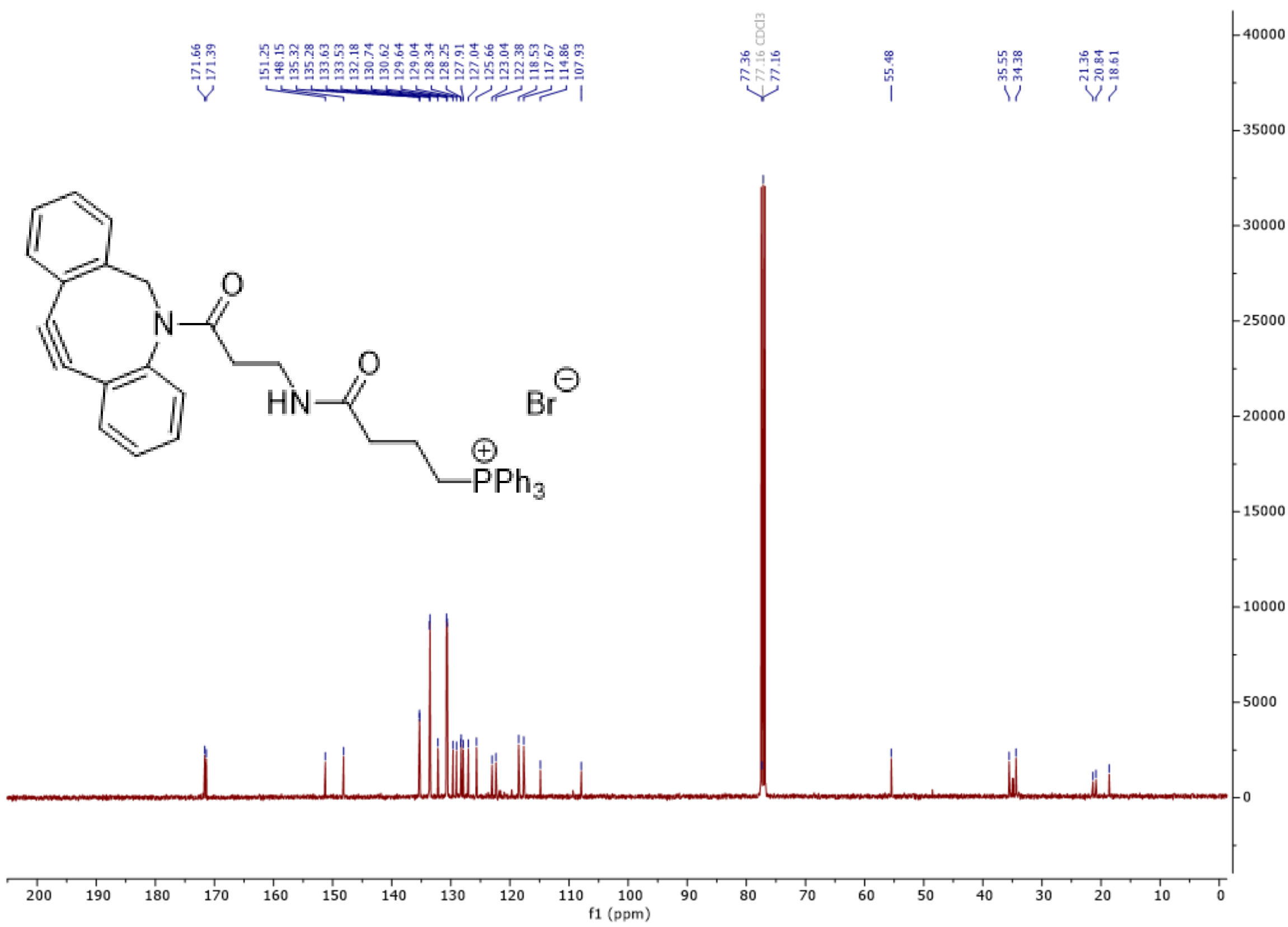

Fig. S37: ¹³C NMR spectrum of compound [DBCO-TPP] in $CDCl_3$ *(100 MHz).*

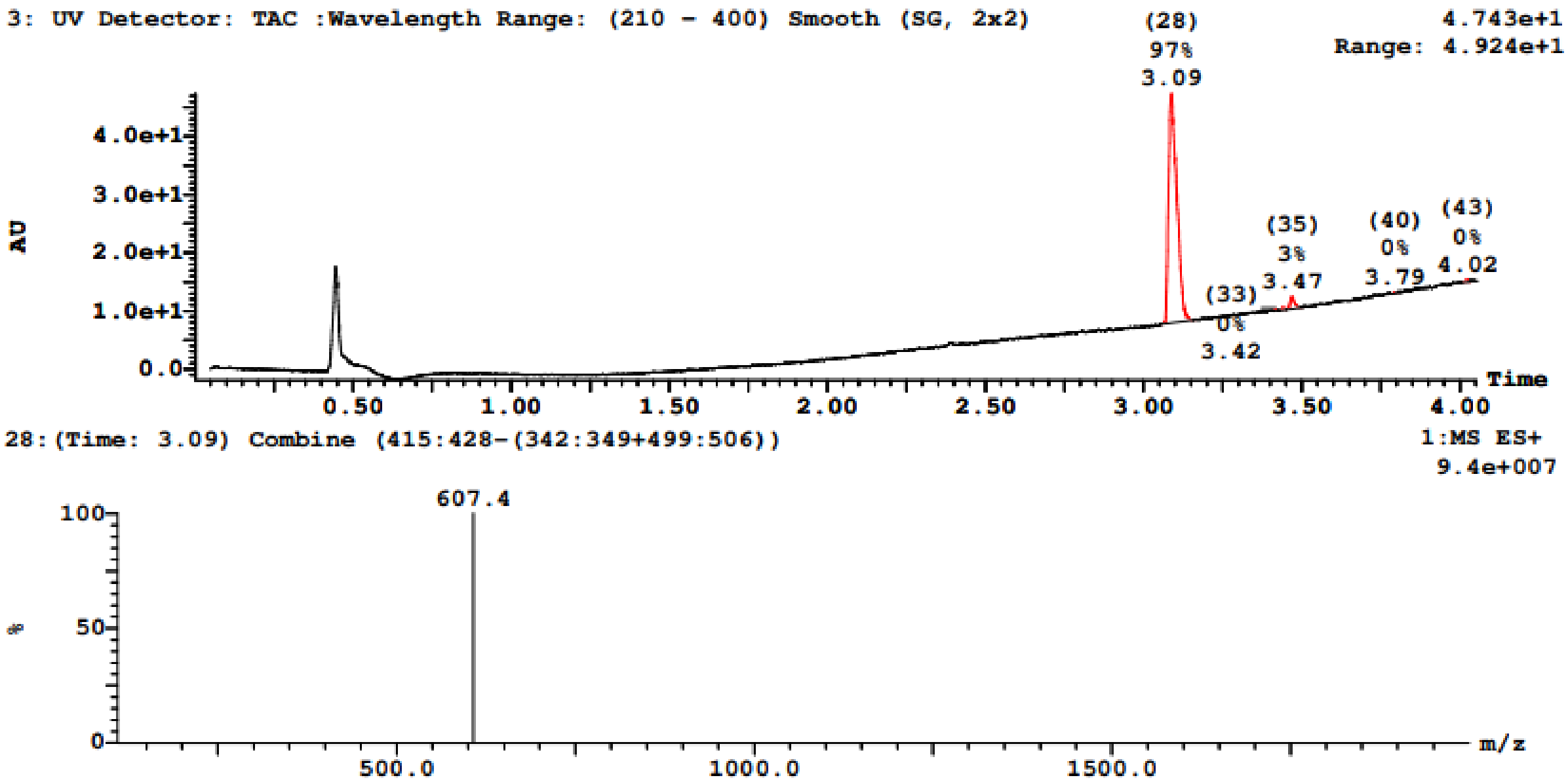

Fig. S38: LC-MS analysis of compound [DBCO-TPP].

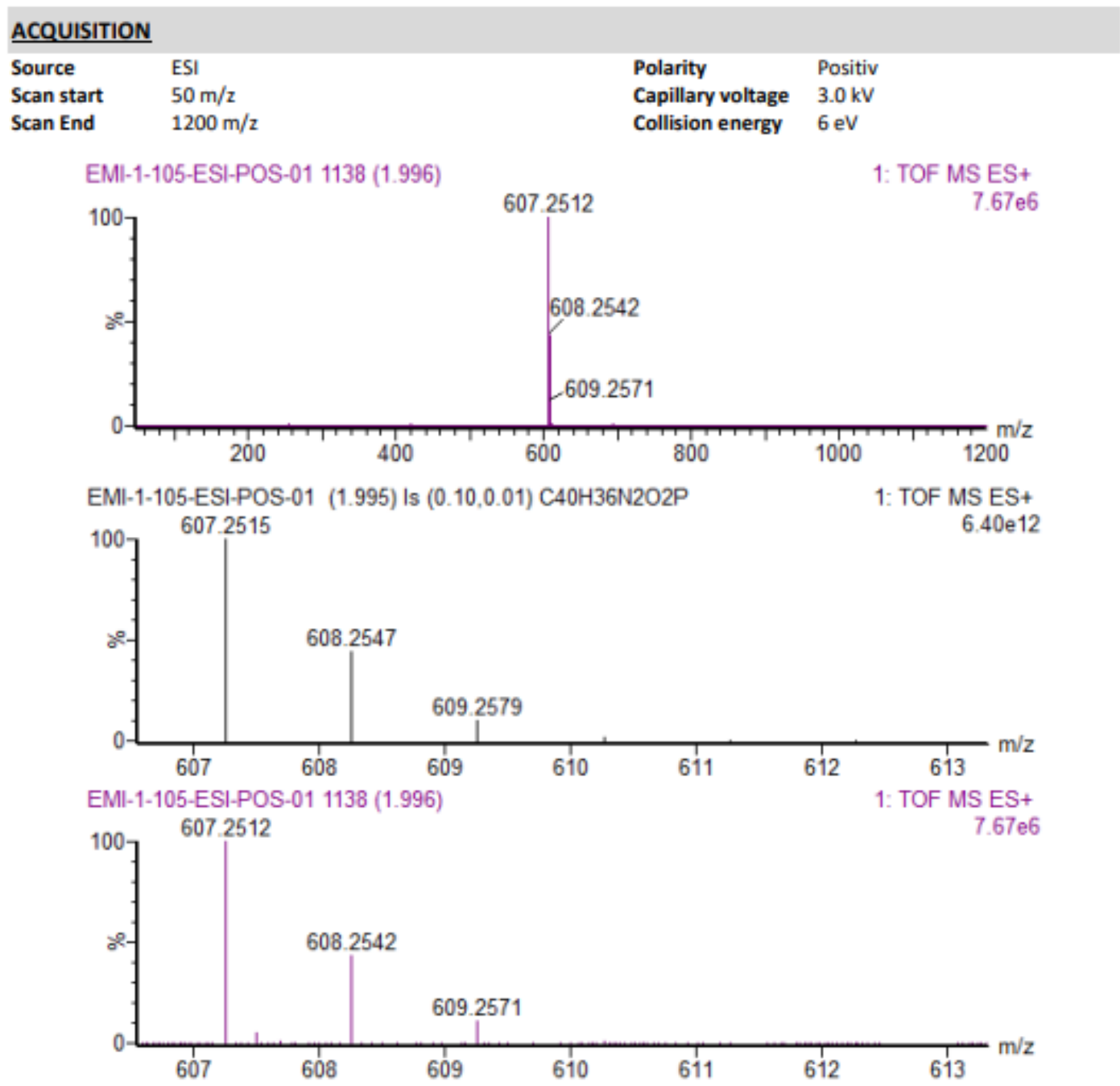

measured m/z	z	Formula	th. m/z	error [mDa]	error [ppm]
607.2512	1	C40H36N2O2P	607.2515	0.3	0.5

HRMS (ESI-TOF) m/z calcd for C40H36N2O2P [M]+ 607.2515; found 607.2512

Fig. S39: HR mass spectrum (ESI, positive mode) of compound [DBCO-TPP].

IV) Photophysical properties:

TzProbe in DMSO

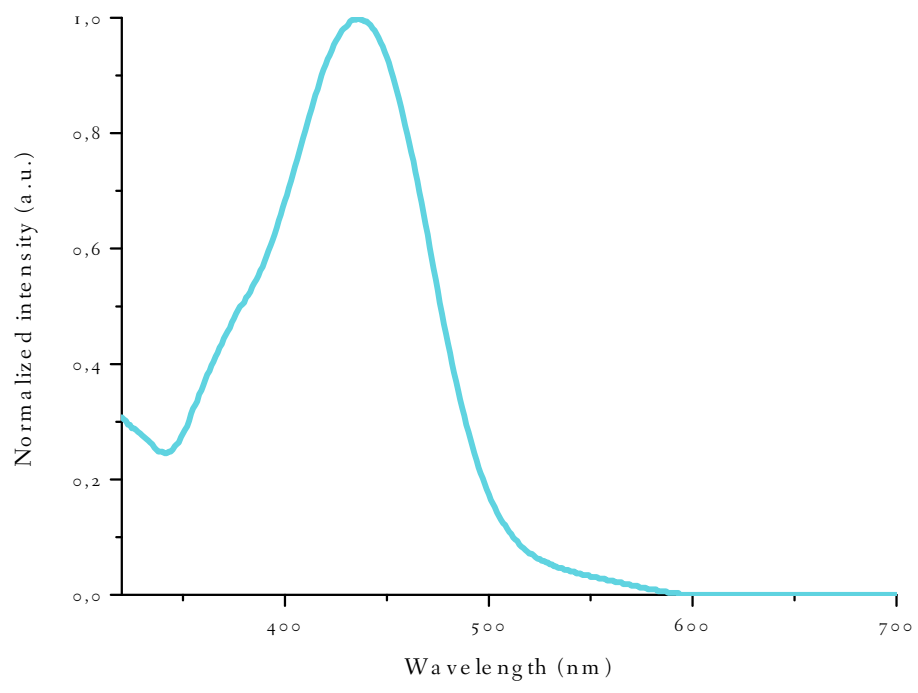

Normalized absorption (blue) in DMSO of **TzProbe**

[A]

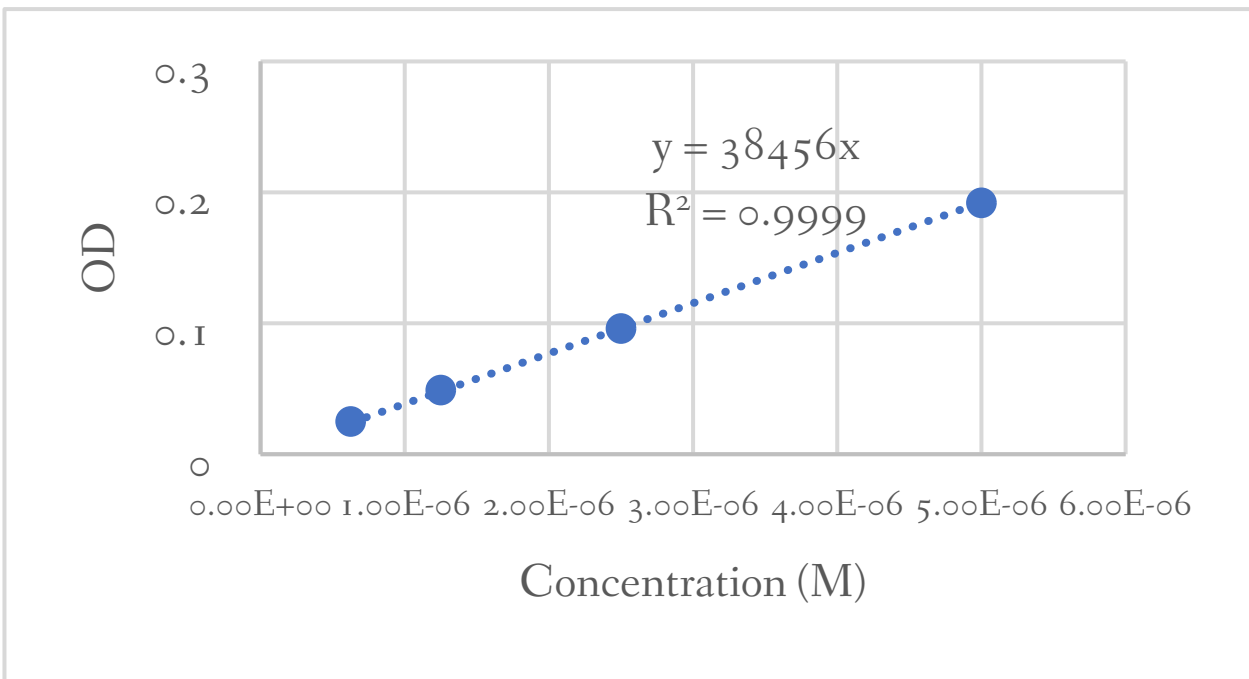

[A] Determination of Molar extinction coefficient for **TzProbe** at 25 °C in DMSO at 436 nm.

TzProbe in PBS/BSA (100eq)

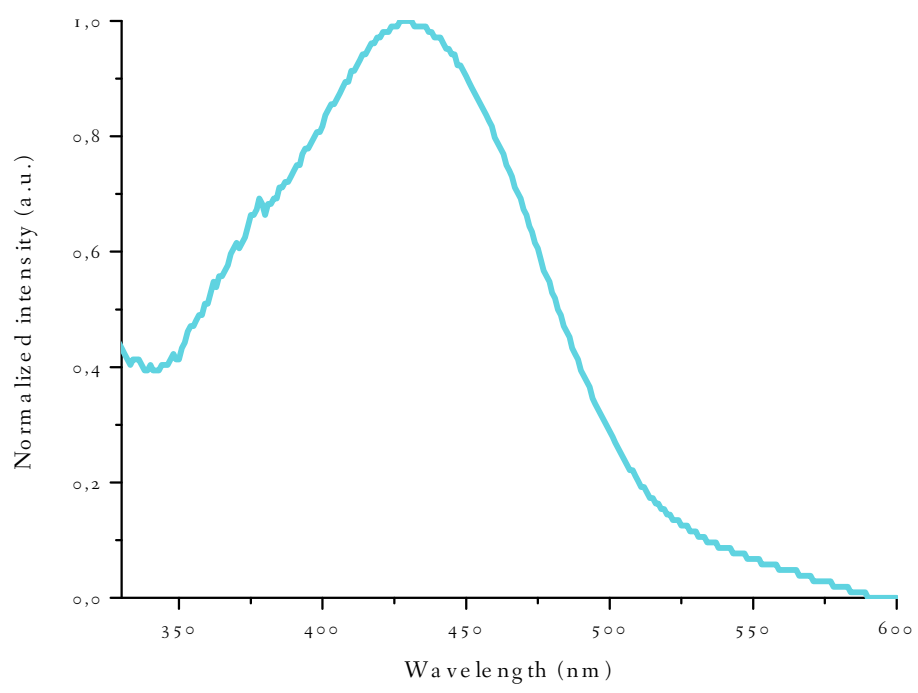

Normalized absorption (blue) in PBS/BSA (100eq) of **TzProbe**

[A]

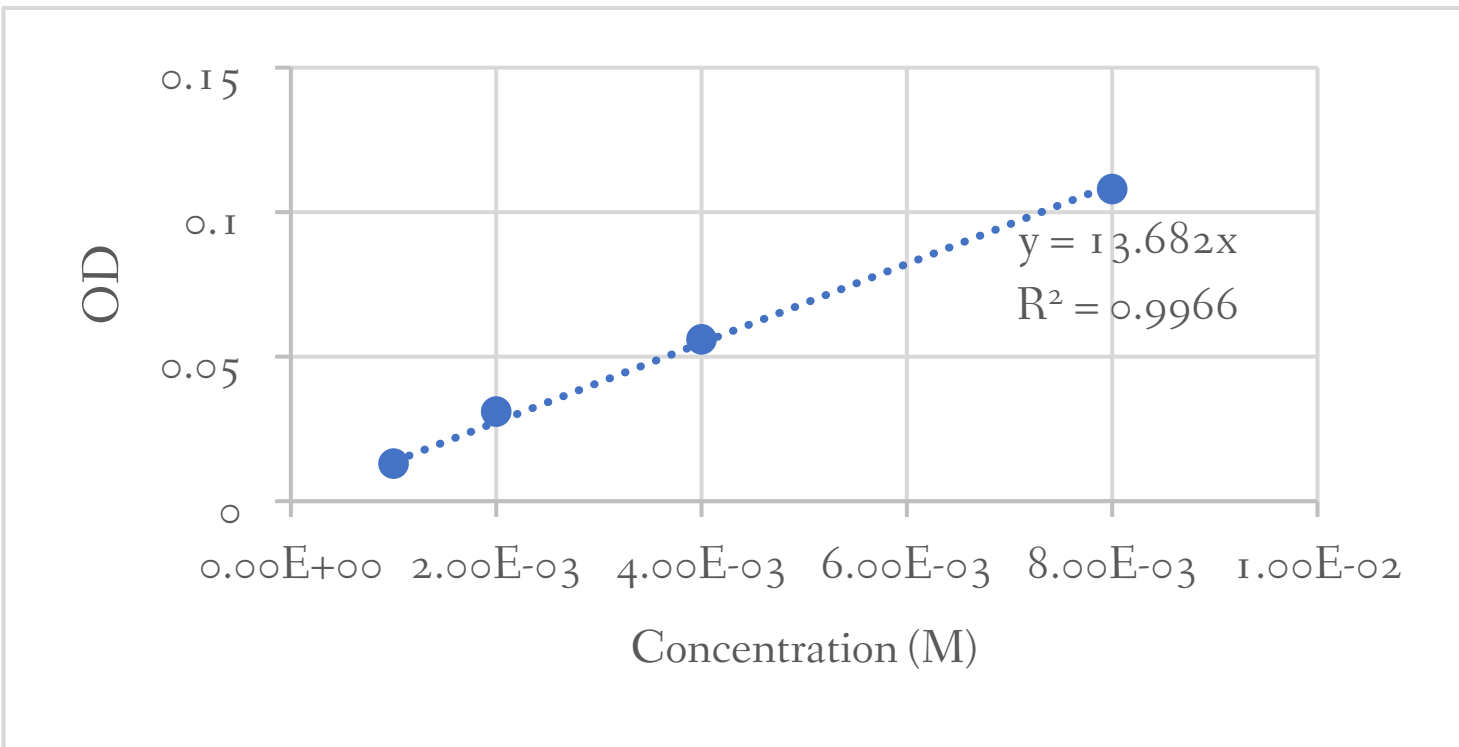

[A] Determination of Molar extinction coefficient for **TzProbe** at 25 °C in PBS/BSA (100eq) at 429 nm.

TzMonoClick in DMSO

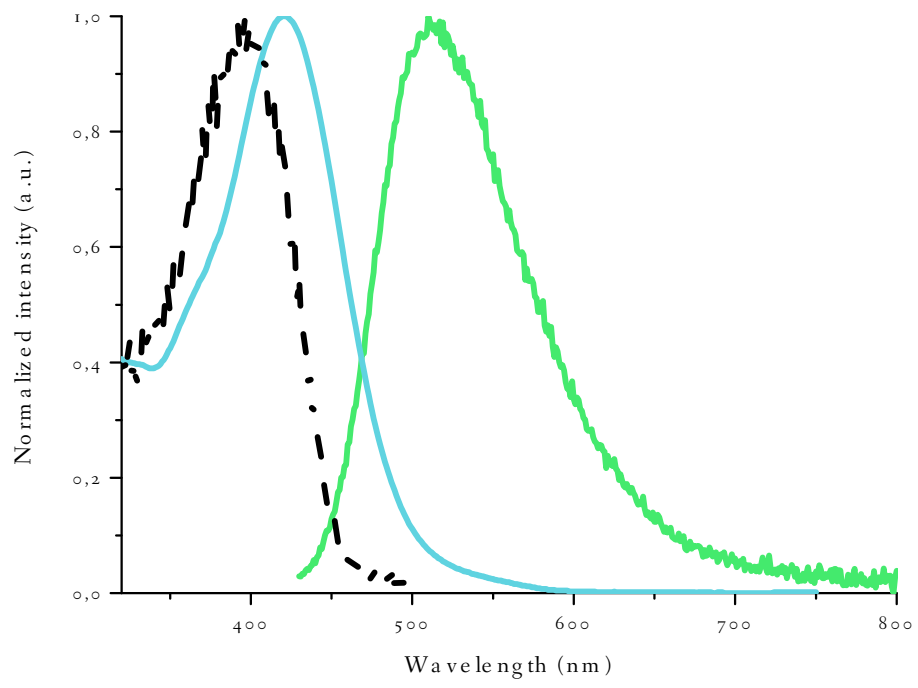

Normalized absorption (blue), emission (green), and excitation (black dashed) spectra in DMSO of **TzMonoClick**

[A]

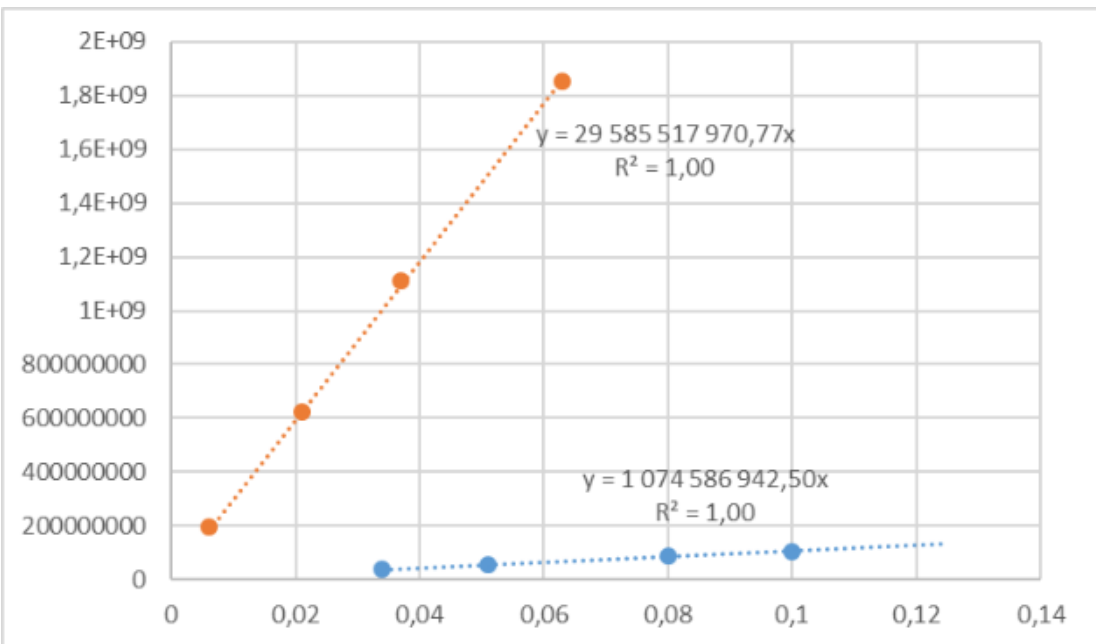

[B]

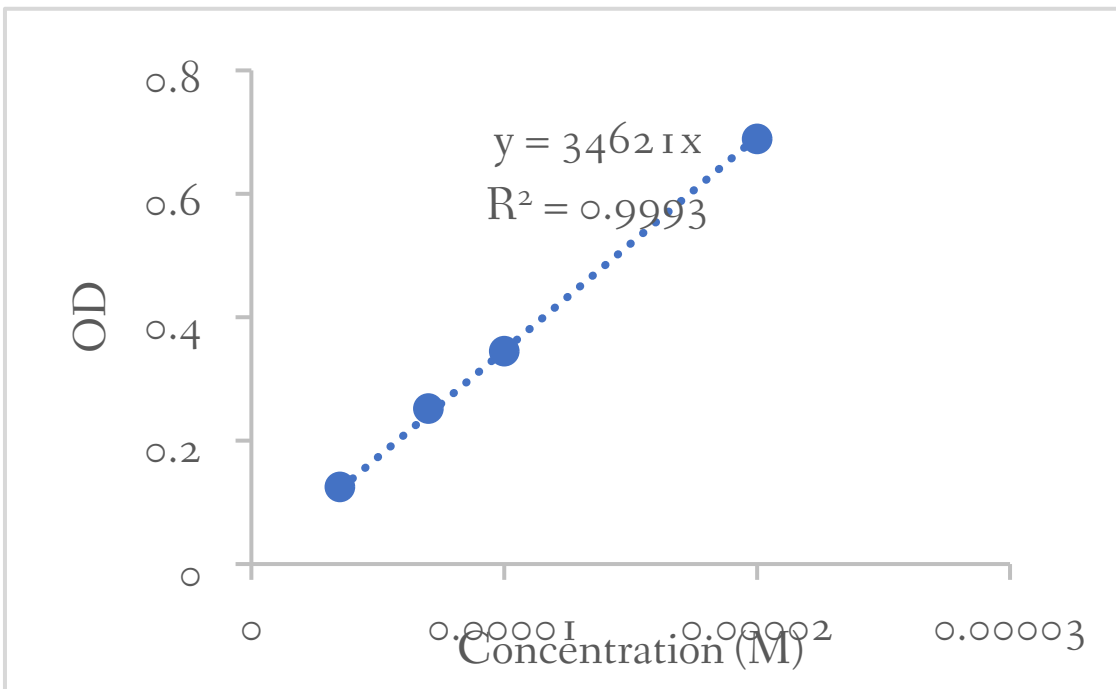

[A] Determination of relative quantum yields for compound **TzMonoClick** (in blue) at 25 °C in DMSO. Coumarin 153 in EtOH (in orange) was used as the reference fluorophore (Φ_F = 0.38 at 420 nm). **[B]** Determination of Molar extinction coefficient for **TzMonoClick** at 25 °C in DMSO at 420 nm.

TzMonoClick in PBS/BSA (100eq)

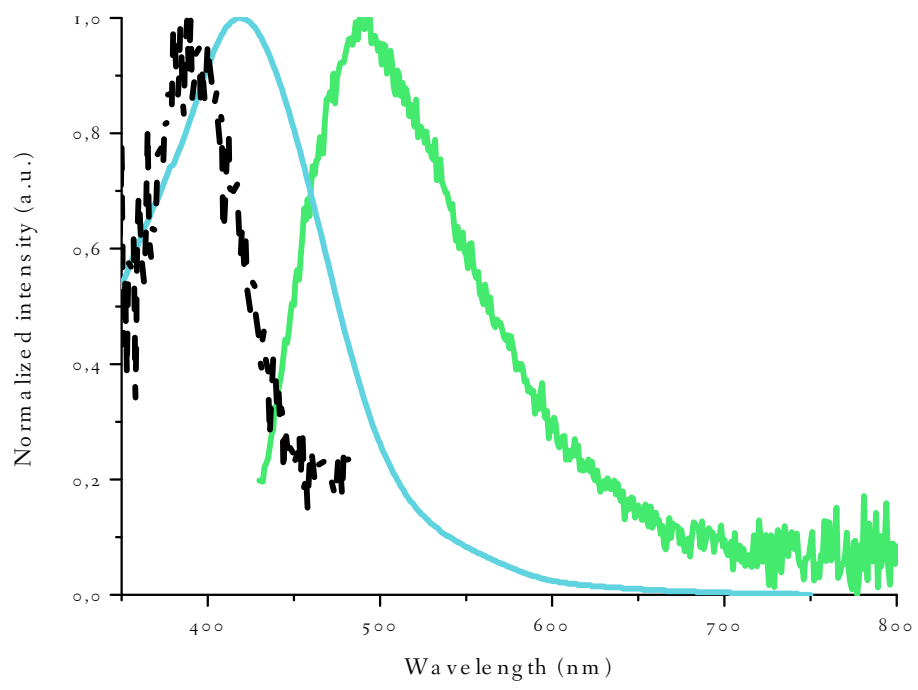

Normalized absorption (blue), emission (green), and excitation (black dashed) spectra in PBS/BSA (100eq) of **TzMonoClick**

[A]

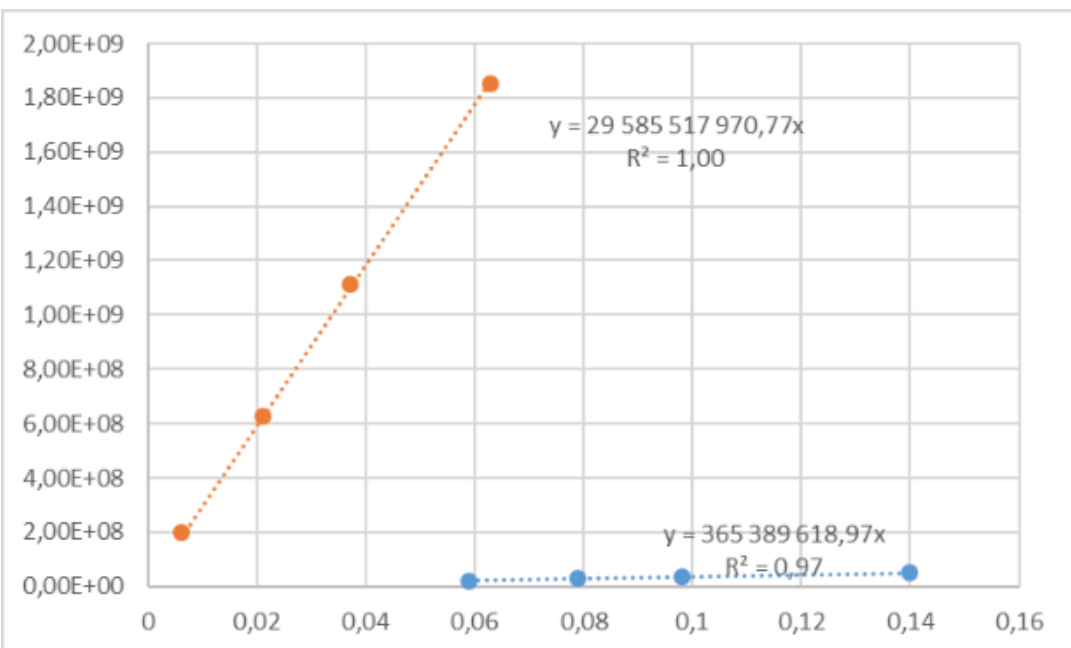

[B]

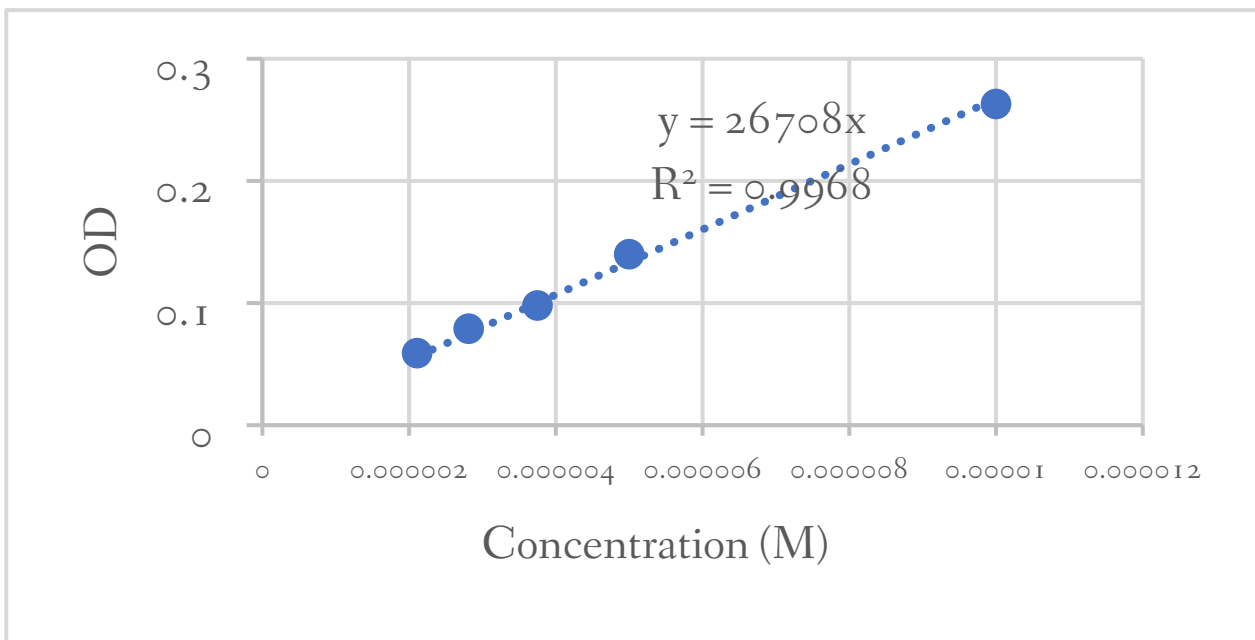

[A] Determination of relative quantum yields for compound **TzMonoClick** (in blue) at 25 °C in PBS/BSA (100eq). Coumarin 153 in EtOH (in orange) was used as the reference fluorophore ($\Phi_F = 0.38$ at 420 nm). **[B]** Determination of Molar extinction coefficient for **TzMonoClick** at 25 °C in PBS/BSA (100eq) at 420 nm.

TzDiClick in DMSO

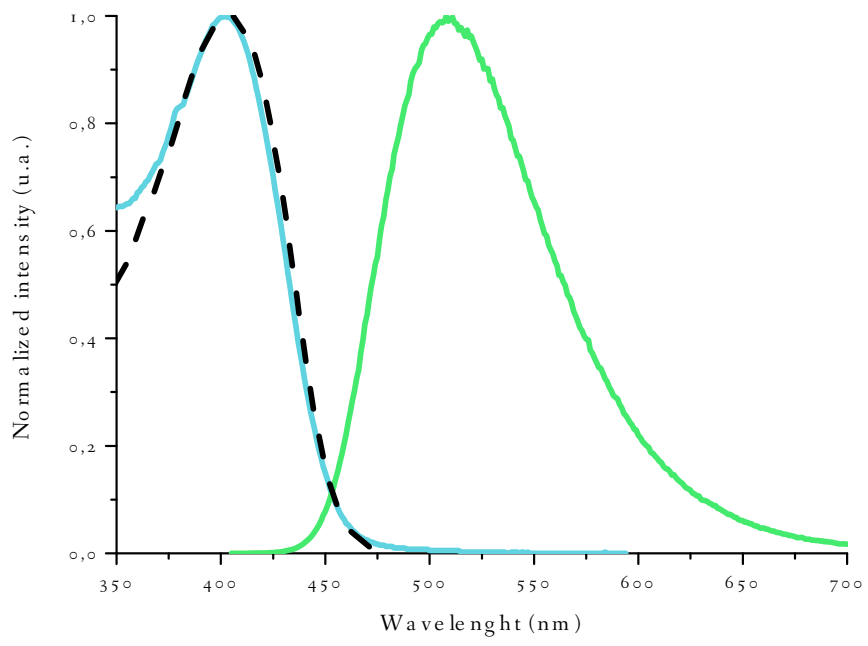

Normalized absorption (blue), emission (green), and excitation (black dashed) spectra in DMSO of **TzDiClick**

[A]

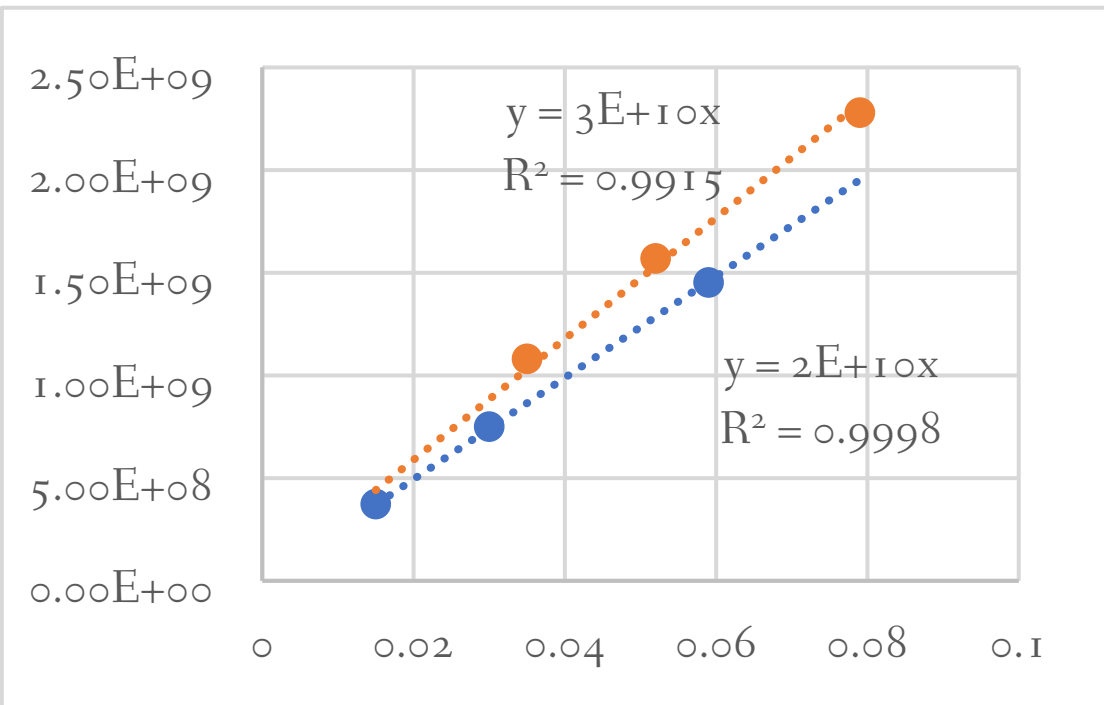

[B]

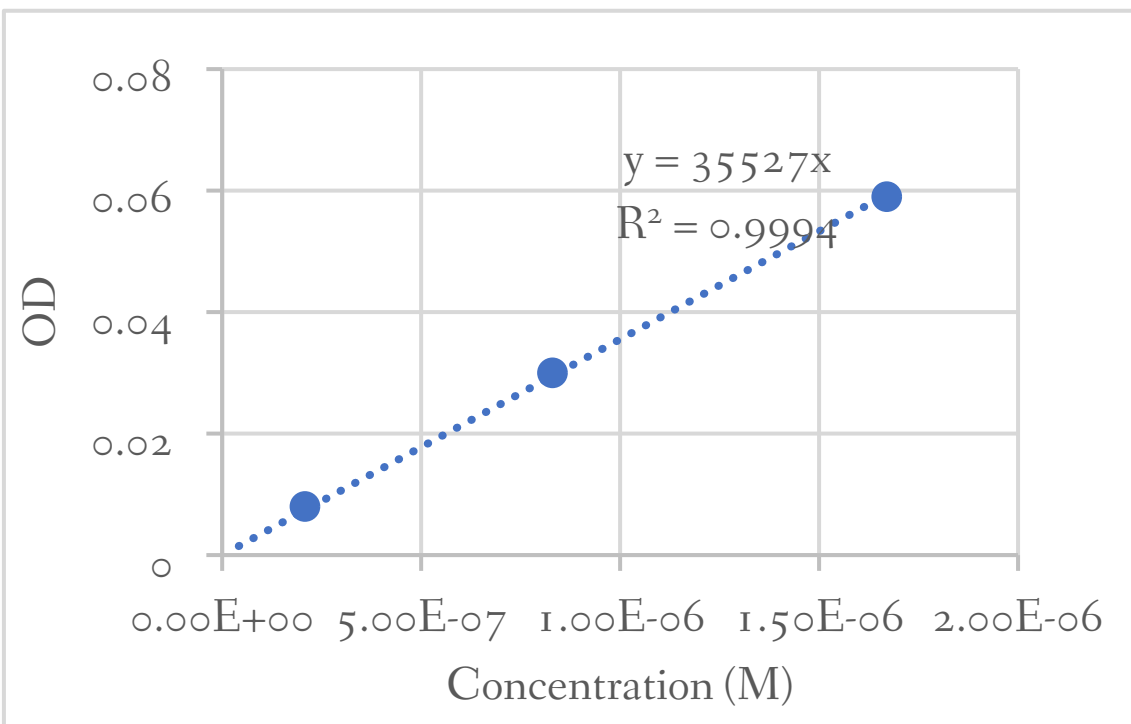

[A] Determination of relative quantum yields for compound **TzDiClick** (in blue) at 25 °C in DMSO. Coumarin 153 in EtOH (in orange) was used as the reference fluorophore (Φ_F = *0.38* at 420 nm). [B] Determination of Molar extinction coefficient for **TzDiClick** at 25 °C in DMSO at 403 nm.

TzDiClick in PBS/BSA (100eq)

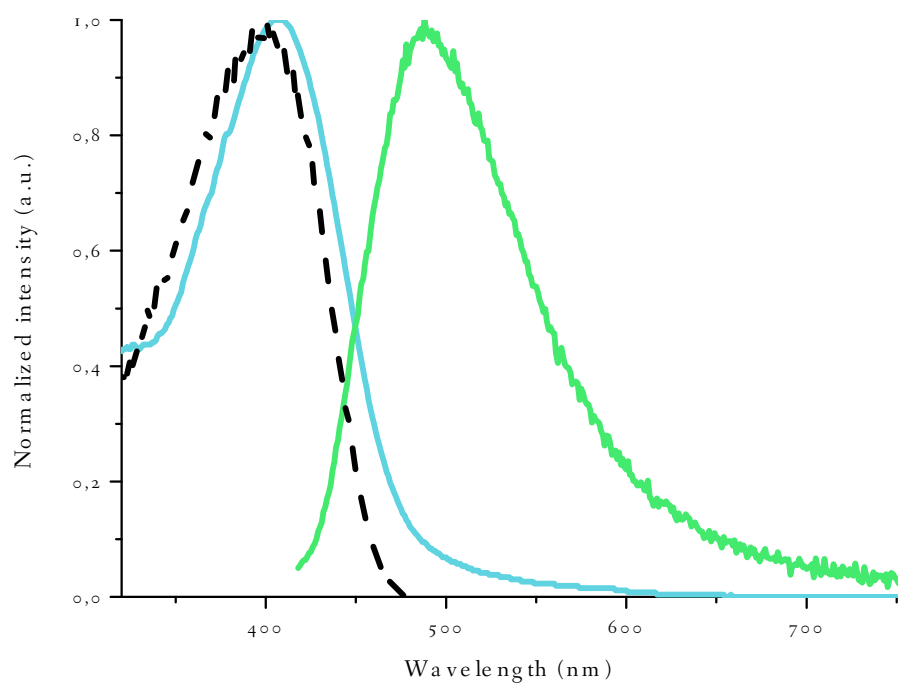

Normalized absorption (blue), emission (green), and excitation (black dashed) spectra in PBS/BSA (100eq) of **TzDiClick**

[A]

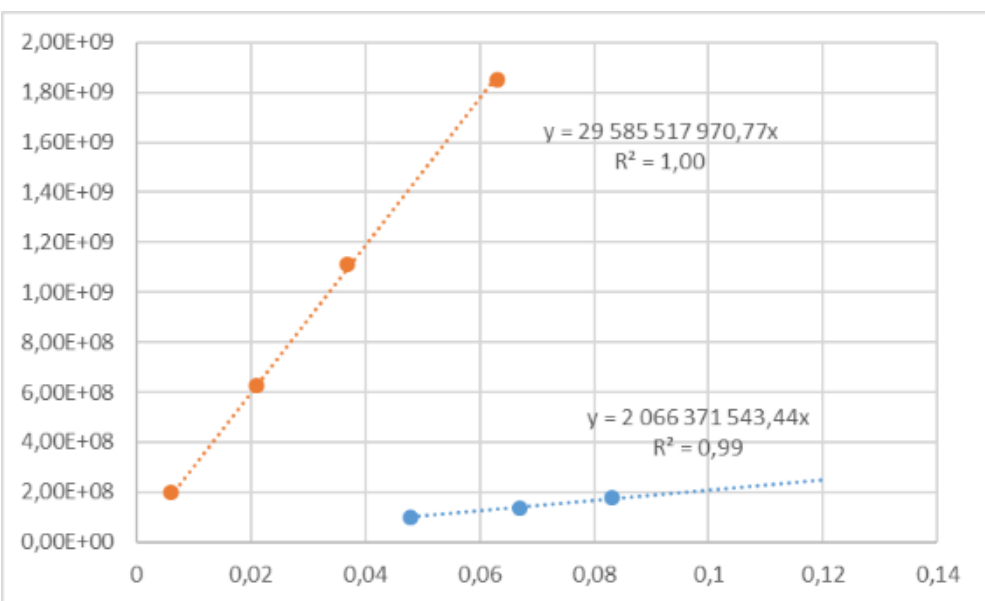

[B]

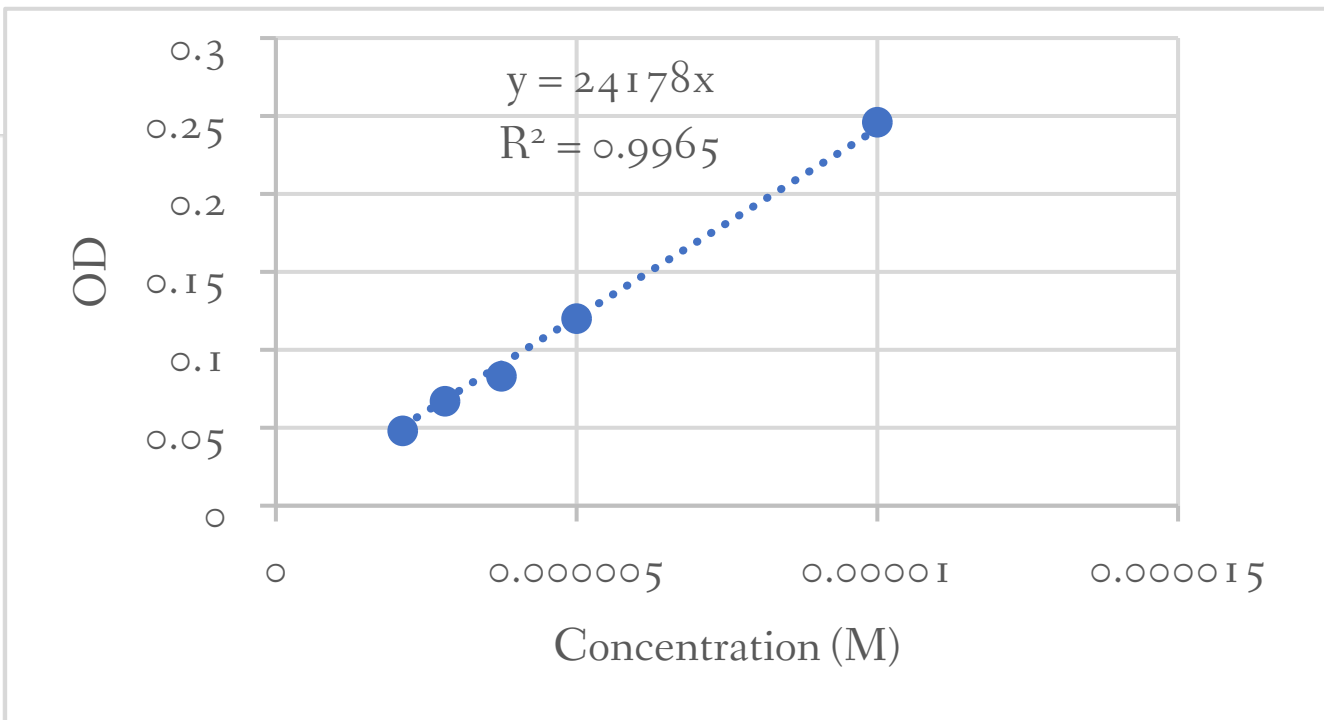

[A] Determination of relative quantum yields for compound **TzDiClick** (in blue) at 25 °C in PBS/BSA (100eq). Coumarin 153 in EtOH (in orange) was used as the reference fluorophore (Φ_F= 0.38 at 420 nm).
[B] Determination of Molar extinction coefficient for **TzDiClick** at 25 °C in PBS/BSA (100eq) at 408 nm.

SydProbe in DMSO

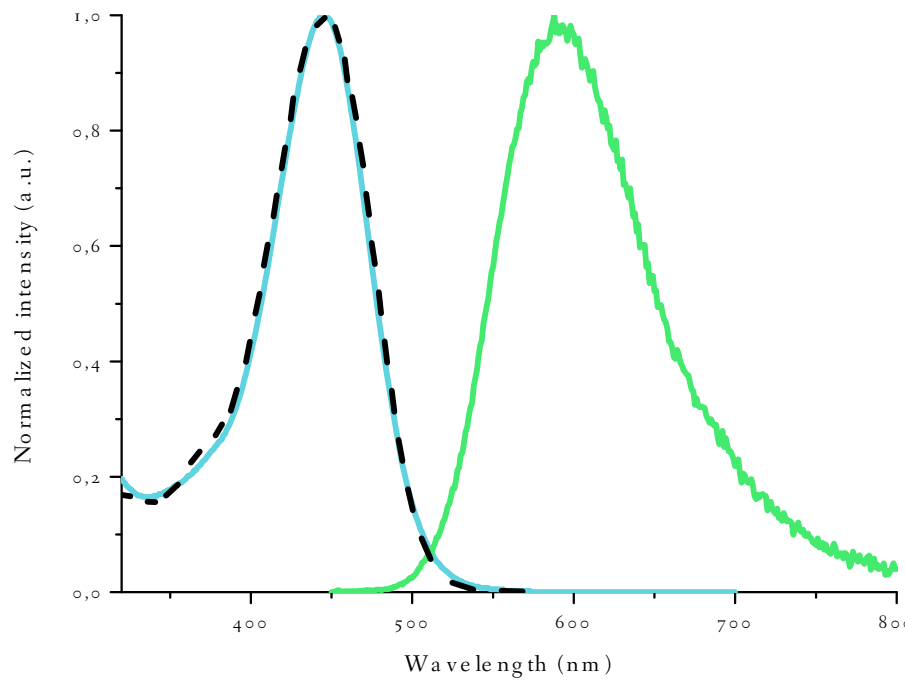

Normalized absorption (blue), emission (green), and excitation (black dashed) spectra in DMSO of **SydProbe**

[A]

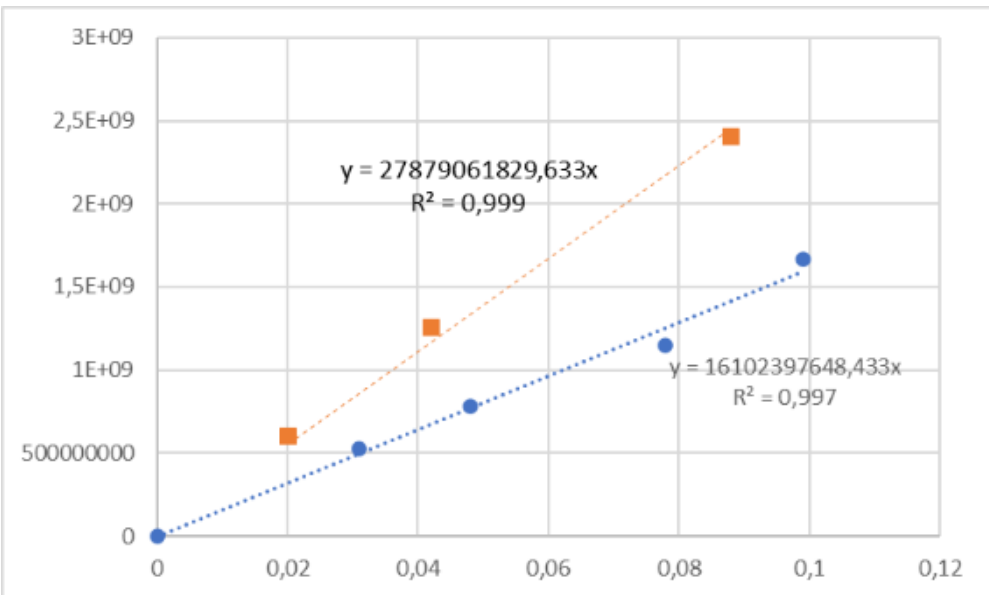

[B]

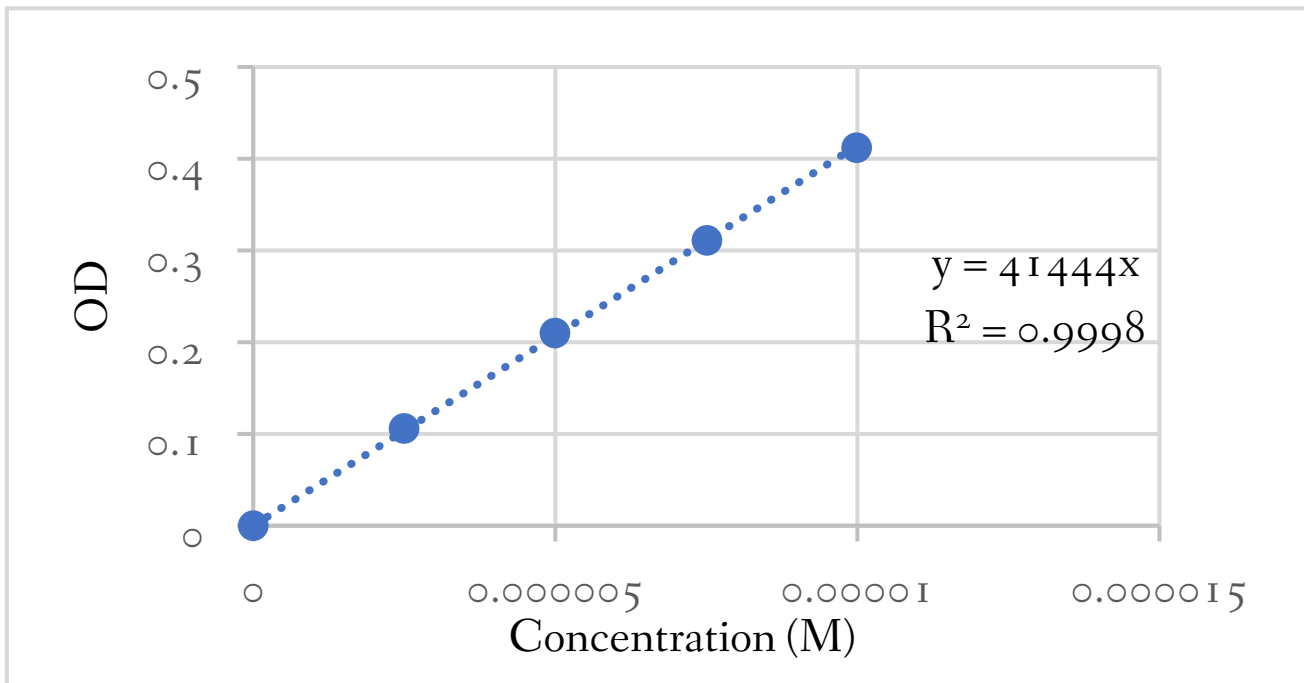

[A] Determination of relative quantum yields for compound **SydProbe** (in blue) at 25 °C in DMSO. Coumarin 153 in EtOH (in orange) was used as the reference fluorophore (Φ_F = 0.38 at 420 nm). **[B]** Determination of Molar extinction coefficient for DMSO at 25 °C in DMSO at 444 nm.

SydProbe in PBS/BSA (100eq)

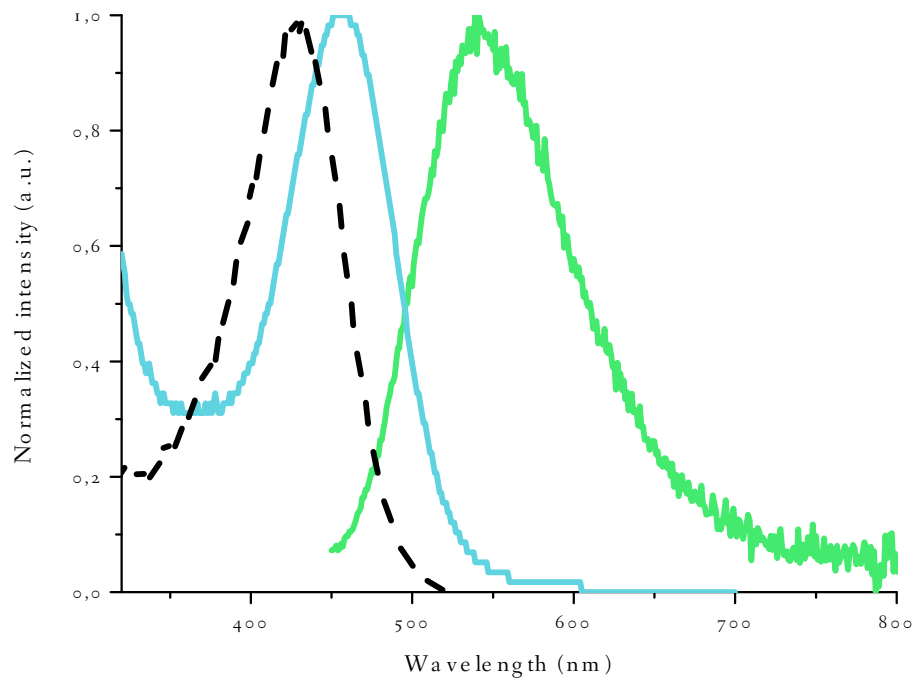

Normalized absorption (blue), emission (green), and excitation (black dashed) spectra in PBS/BSA (100eq) of **SydProbe**

[A]

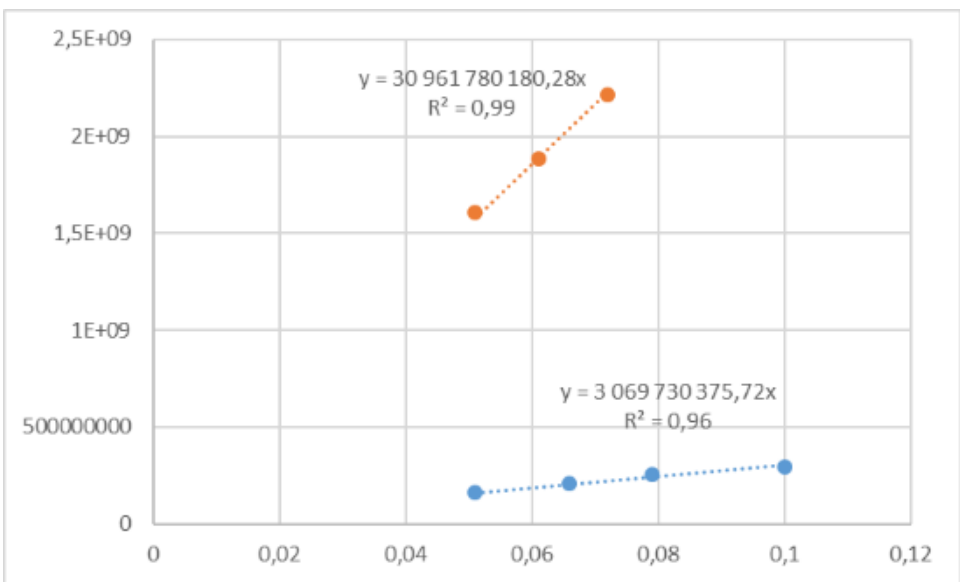

[B]

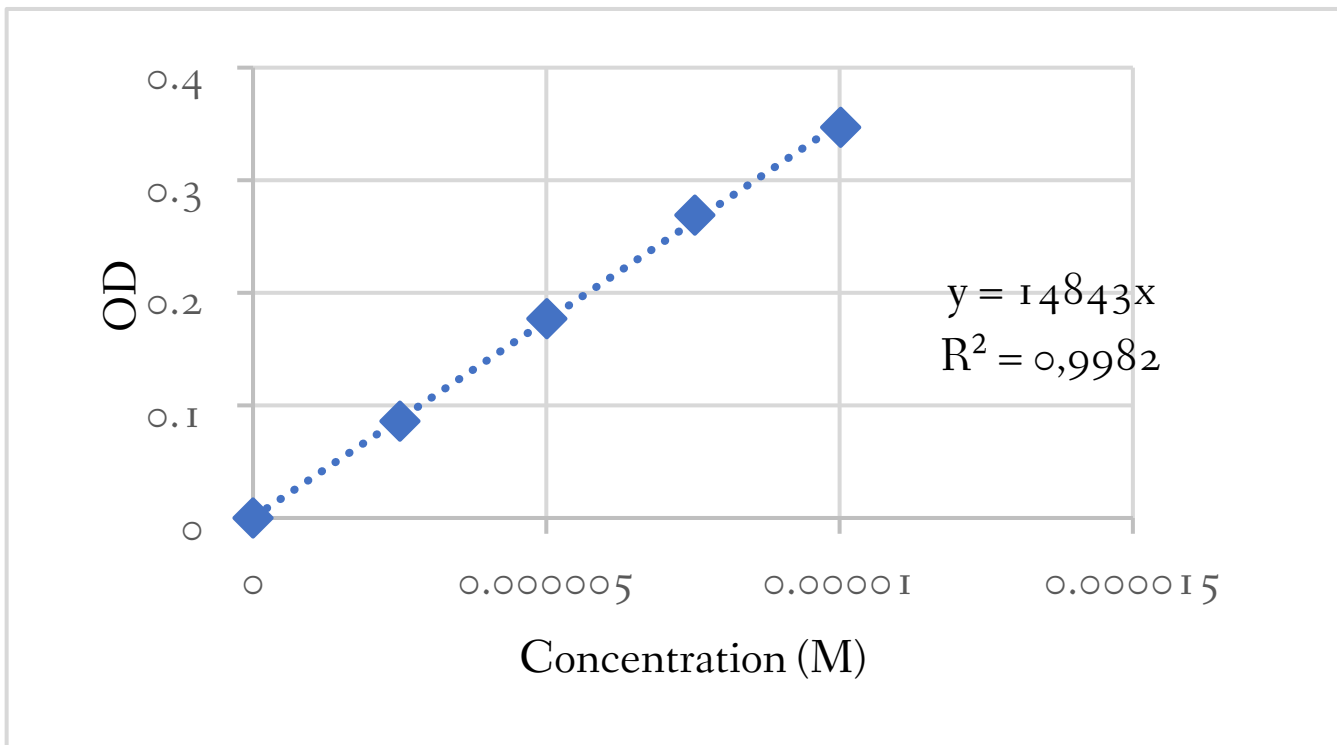

[A] Determination of relative quantum yields for compound **SydProbe** (in blue) at 25 °C in PBS/BSA (100eq). Coumarin 153 in EtOH (in orange) was used as the reference fluorophore (Φ_F = 0.38 at 420 nm). **[B]** Determination of Molar extinction coefficient for **SydProbe** at 25 °C in PBS/BSA (100eq) at 457 nm.

SydMonoClick in DMSO

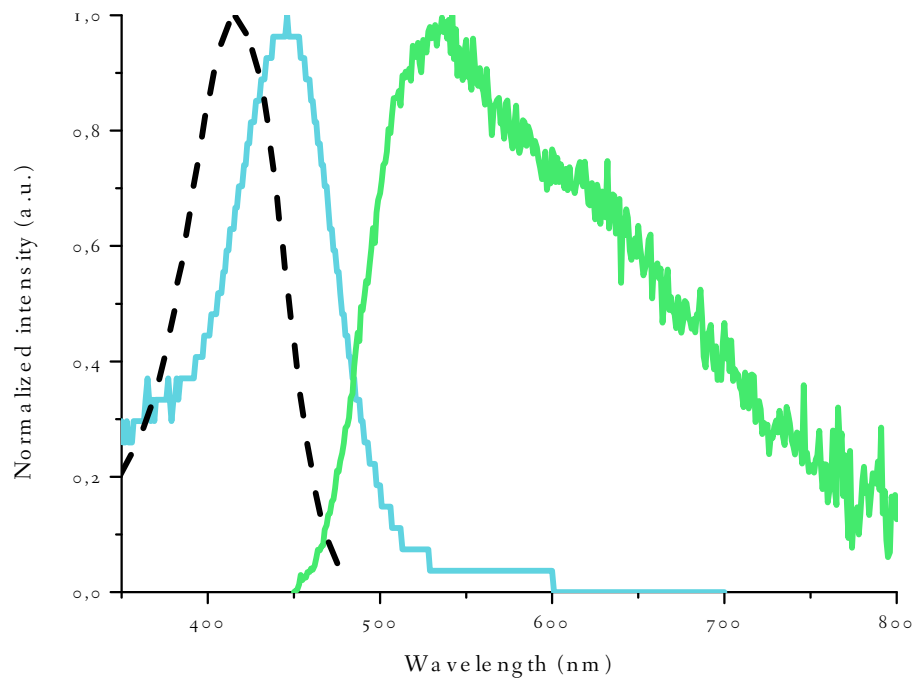

Normalized absorption (blue), emission (green), and excitation (black dashed) spectra in DMSO of **SydMonoClick**

[A] [B]

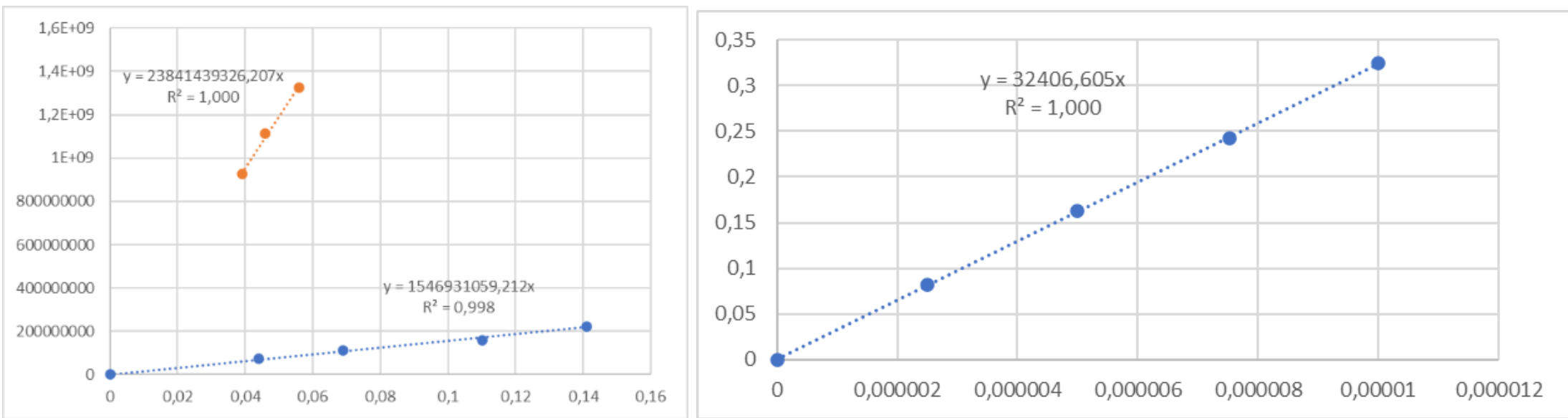

[A] Determination of relative quantum yields for compound **SydMonoClick** at 25 °C (in blue) in DMSO. Coumarin 153 in EtOH (in orange) was used as the reference fluorophore (Φ_F = 0.38 at 420 nm). **[B]** Determination of Molar extinction coefficient for **SydMonoClick** at 25 °C in DMSO at 446 nm.

SydMonoClick in PBS/BSA (100eq)

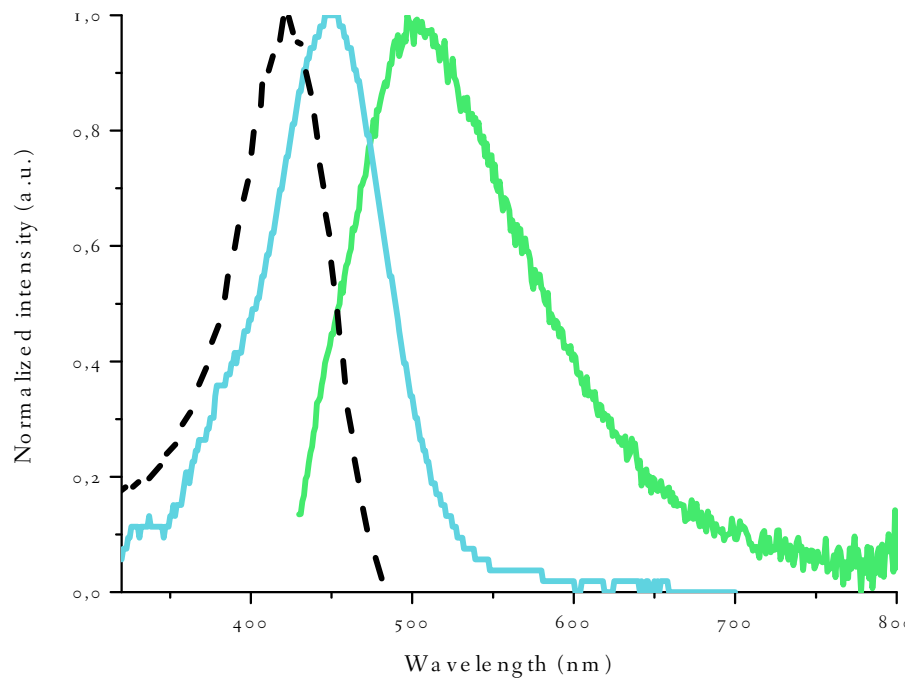

Normalized absorption (blue), emission (green), and excitation (black dashed) spectra in PBS/BSA (100eq) of **SydMonoClick**

[A]

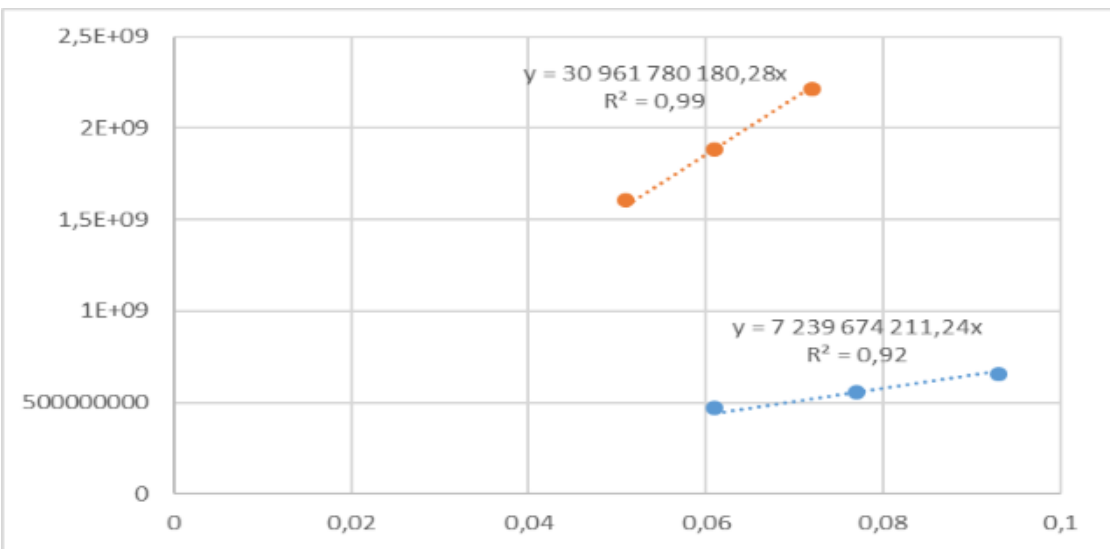

[B]

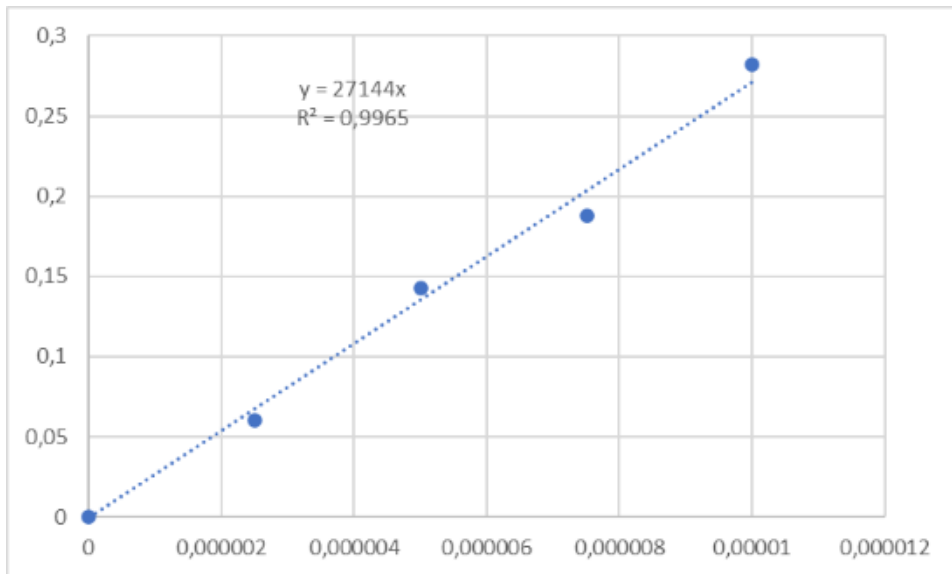

[A] Determination of relative quantum yields for compound **SydMonoClick** (in blue) at 25 °C in PBS/BSA (100eq). Coumarin 153 in EtOH (in orange) was used as the reference fluorophore (Φ_F= 0.38 at 420 nm).
[B] Determination of Molar extinction coefficient for **SydMonoClick** at 25 °C in PBS/BSA (100eq) at 453 nm.

SydDiClick in DMSO

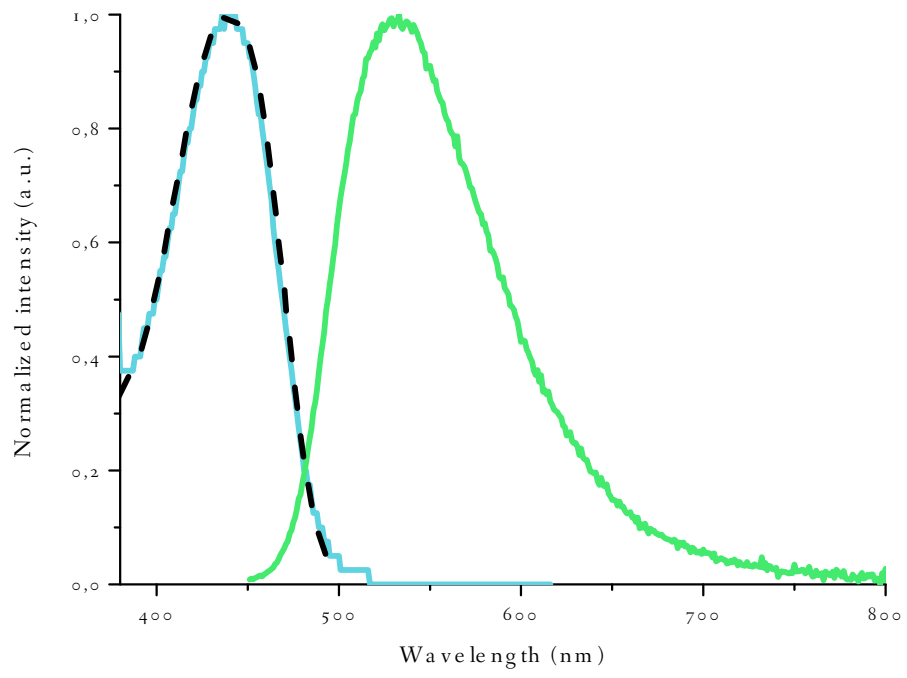

Normalized absorption (blue), emission (green), and excitation (black dashed) spectra in DMSO of **SydDiClick**

[A]

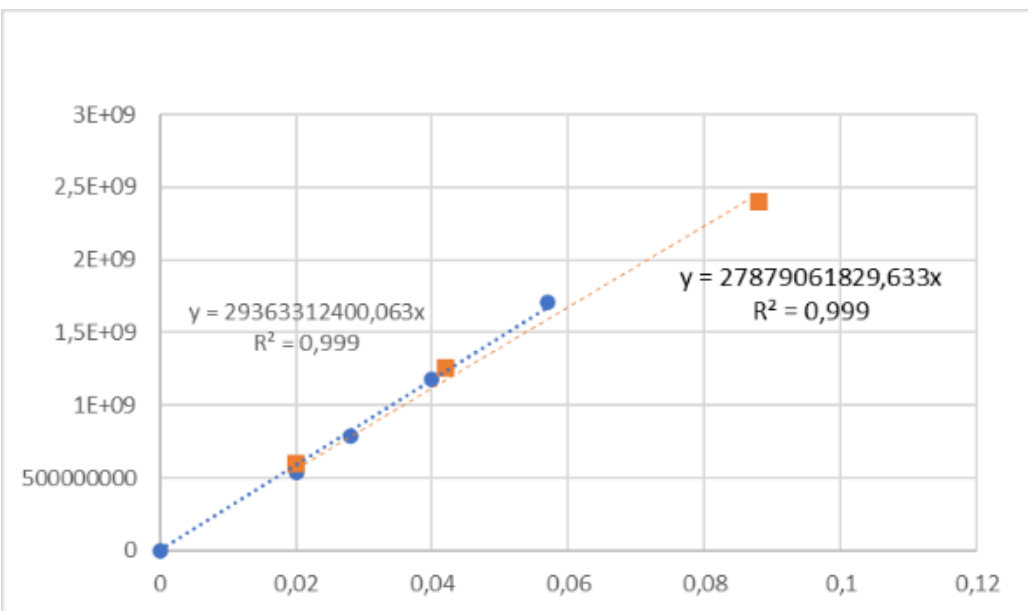

[B]

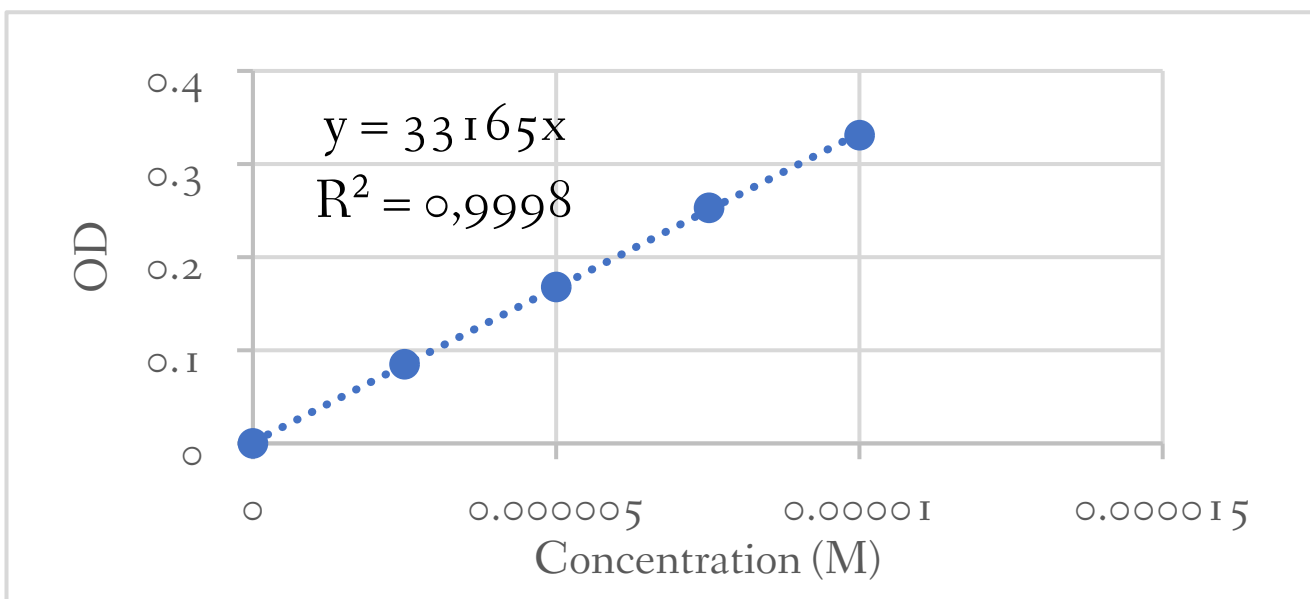

[A] Determination of relative quantum yields for compound **SydDiClick** at 25 °C (in blue) in DMSO. Coumarin 153 in EtOH (in orange) was used as the reference fluorophore (Φ_F = 0.38 at 420 nm). **[B]** Determination of Molar extinction coefficient for **SydDiClick** at 25 °C in DMSO at 438 nm.

SydDiClick in PBS/BSA (100eq)

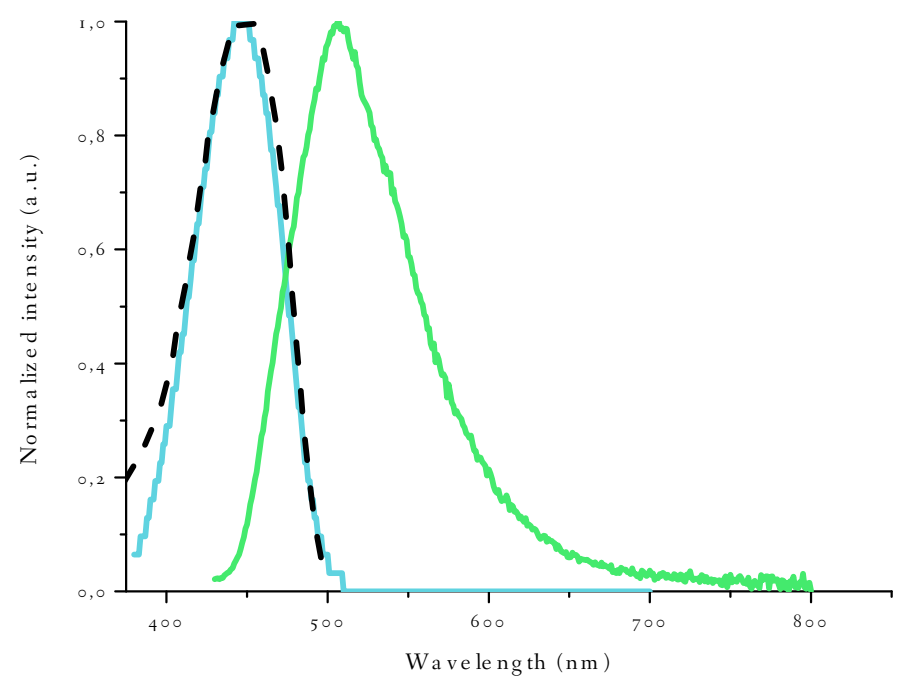

Normalized absorption (blue), emission (green), and excitation (black dashed) spectra in PBS/BSA (100eq) of **SydDiClick**

[A]

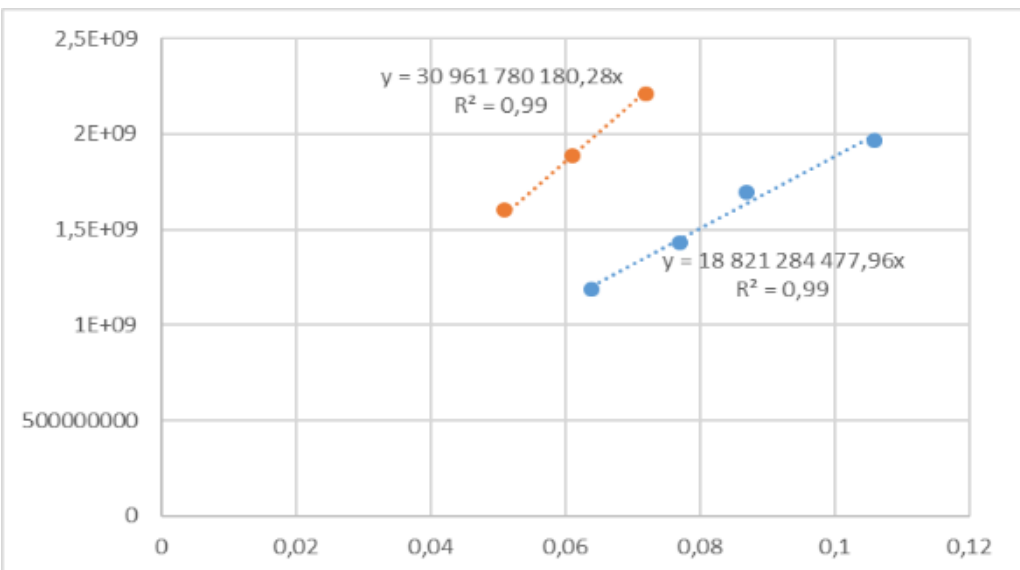

[B]

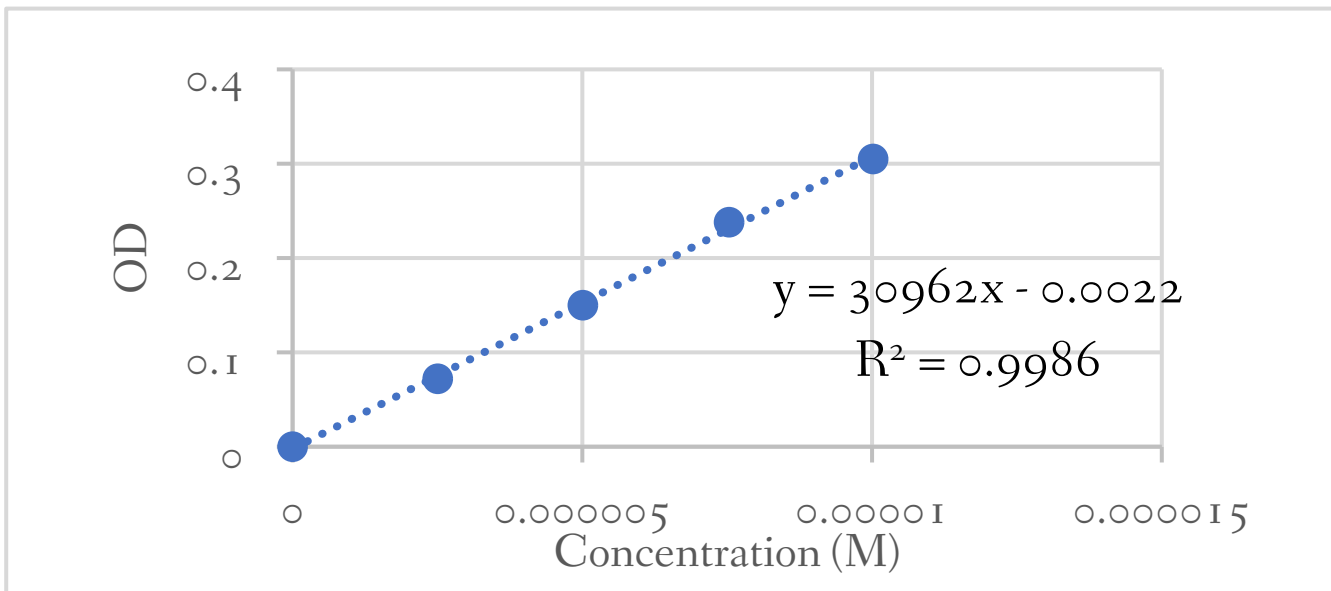

[A] Determination of relative quantum yields for compound **SydDiClick** (in blue) at 25 °C in PBS/BSA (100eq). Coumarin 153 in EtOH (in orange) was used as the reference fluorophore (Φ_F= 0.38 at 420 nm).
[B] Determination of Molar extinction coefficient for **SydDiClick** at 25 °C in PBS/BSA (100eq) at 445 nm.

V) Kinetics:

Measurements were performed in DMSO or in PBS 0,1M with 100 equivalents of BSA at 37 °C. Fluorescence spectra were recorded before and after the reaction to determine the fluorescence turn-on values. For kinetic measurements, the fluorescence signal was first recorded for a few seconds in the absence of BCN to determine the initial fluorescence intensity (F_0). BCN was subsequently injected, and reaction kinetics were monitored at three different BCN concentrations, with two measurements performed for each concentration.

The fluorescence activation kinetics of the probes were analyzed according to the reaction mechanism involved. For probes containing two clickable moieties, the overall transformation proceeds through two consecutive click reactions:

$$A \xrightarrow{k'_1} B \xrightarrow{k'_2} C$$

where A, B, and C correspond respectively to the initial probe, the mono-clicked intermediate, and the final di-clicked product. In this case, TzProbe and SydProbe correspond to species A, TzMonoClick and SydMonoClick to species B, and TzDiClick and SydDiClick to species C.

The kinetics of the reaction between **TzMonoClick/SydMonoClick** and BCN/DBCO were studied under pseudo-first-order conditions using an excess of BCN. Fluorescence intensity traces were fitted using a mono-exponential function:

$$F(t) = F_{\square} + (F_0 - F_{\square})e^{-k'_2 t}$$

where *F(t)* is the fluorescence intensity at time *t*, F_0 and $F_{\square}$ are the initial and final fluorescence intensities, respectively, and k'_2 is the observed pseudo-first-order rate constant.

Under pseudo-first-order conditions:

$$k'_2 = k_2[BCN]$$

where k'_2 is the observed pseudo-first-order rate constant and k_2 the second-order rate constant of the B → C transformation. Values of k'_2 obtained at different BCN concentrations were plotted against [BCN], and k_2 was determined from the slope of the linear fit.

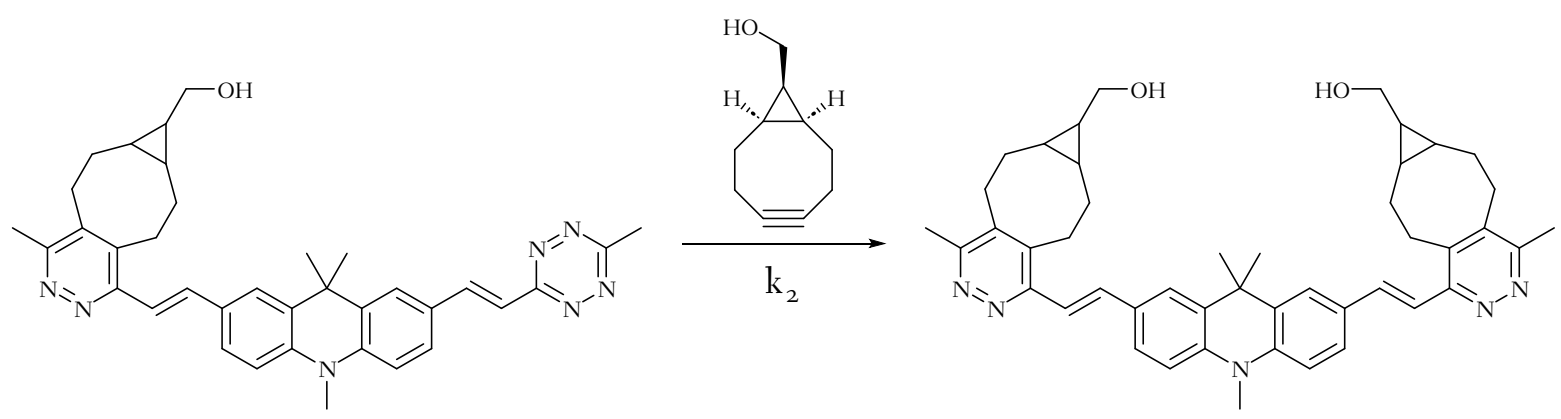

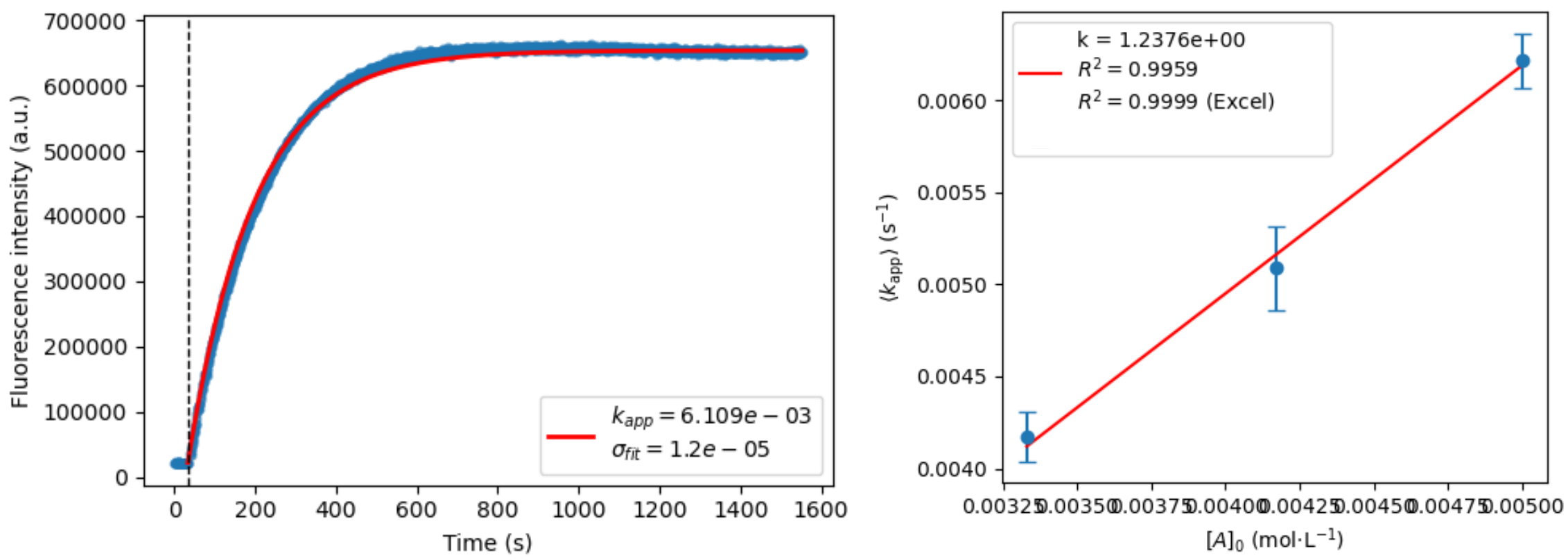

Click kinetics of the **TzMonoClick** in DMSO at 37 °C (20 μM probe – 250 eq BCN – Excitation at 395nm – Emission 515nm)

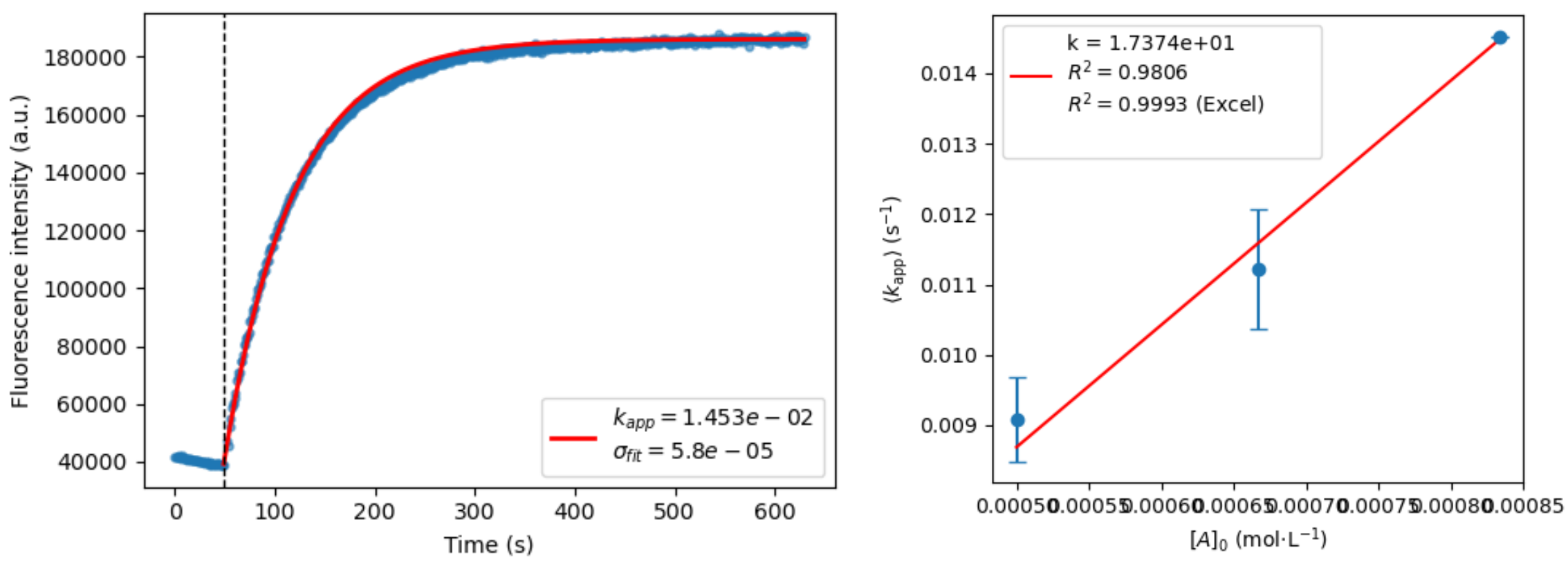

Click kinetics of the **TzMonoClick** in PBS/BSA(100eq) at 37 °C (5 μM probe – 167 eq BCN – Excitation at 408nm – Emission 500nm)

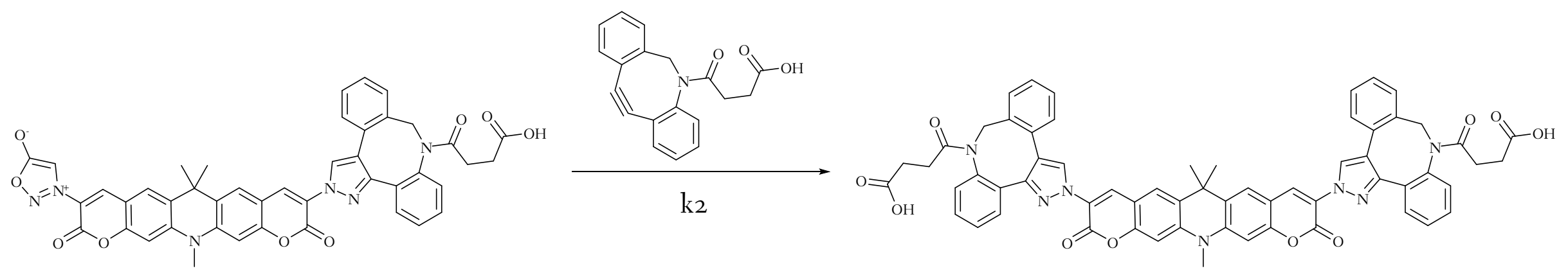

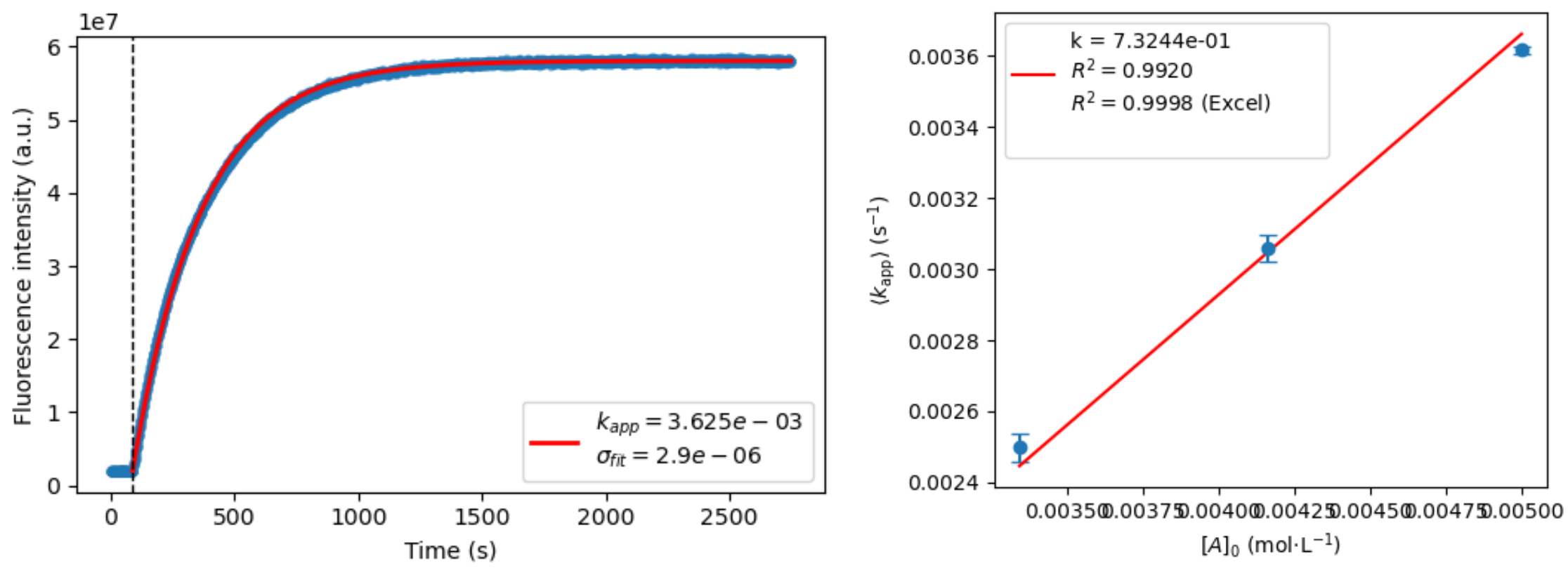

Click kinetics of the **SydMonoClick** in DMSO at 37 °C (20 μM probe – 250 eq BCN – Excitation at 440nm – Emission 500nm)

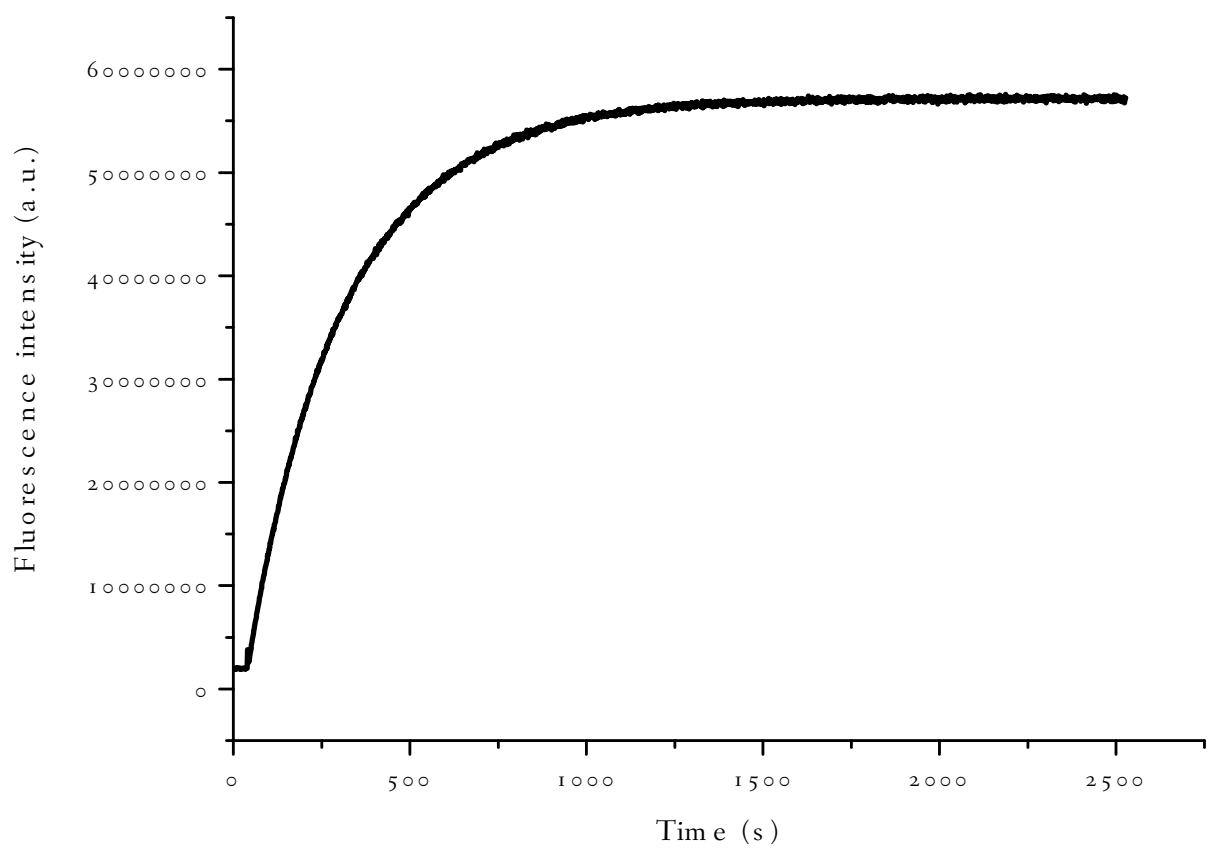

Click kinetics of the **SydMonoClick** in PBS/BSA(100eq) at 37 °C (10 μM probe – 250 eq BCN – Excitation at 440nm – Emission 500nm)

For probes containing two clickable moieties, the fluorescence activation proceeds through two consecutive click reactions:

$$A \xrightarrow{k'_1} B \xrightarrow{k'_2} C$$

The reactions were performed under pseudo-first-order conditions by using a large excess of BCN. Under these conditions, the concentration of BCN remains constant throughout the reaction, allowing the bimolecular rate constants to be expressed as apparent first-order rate constants:

$$k'_1 = k_1[BCN]$$

$$k'_2 = k_2[BCN]$$

where k'_1 and k'_2 are the observed pseudo-first-order rate constants (s^{-1}) corresponding to the $A \rightarrow B$ and $B \rightarrow C$ transformations, respectively, and k_1 and k_2 are the corresponding second-order rate constants ($M^{-1}.s^{-1}$).

The consecutive reaction can therefore be described by the following differential equations:

$$\frac{d[A]}{dt} = -k'_1[A]$$

$$\frac{d[B]}{dt} = k'_1[A] - k'_2[B]$$

$$\frac{d[B]}{dt} = -k'_2[B]$$

Integration of the first equation gives:

$$[A]t = [A]_0 e^{-k'_1 t}$$

The second equation gives:

$$\frac{d[B]}{dt} + k'_2[B] = k'_1[A]_0 e^{-k'_1 t}$$

This first-order linear differential equation can be solved analytically using the integrating factor method. Applying the initial condition $[B]_0=0$ leads to :

$$[B]_t = \frac{k'_1[A]_0}{k'_2 - k'_1}(e^{-k'_1 t} - e^{-k'_2 t})$$

The concentration of the final product C is therefore:

$$[C]_t = [A]_0(1 - \frac{k'_2 e^{-k'_1 t} - k'_1 e^{-k'_2 t}}{k'_2 - k'_1})$$

Since the fluorescence signal is directly related to the formation of the fluorescent product C, the fluorescence evolution was described using:

$$F(t) = F_0 + F_{∞}\left(1 - \frac{k'_2 e^{-k'_1 t} - k'_1 e^{-k'_2 t}}{k'_2 - k'_1}\right)$$

$F(t)$: fluorescence intensity at time t

F_0 : initial fluorescence intensity

$F_{∞}$: maximum fluorescence increase after complete conversion into product C

k'_1: observed pseudo-first-order rate constant for the A → B transformation

k'_2 : observed pseudo-first-order rate constant for the B → C transformation

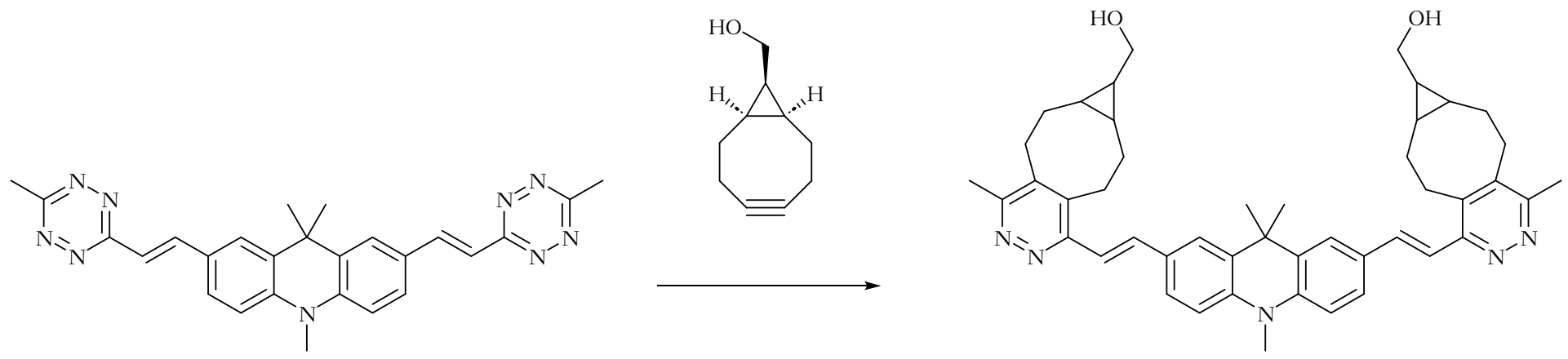

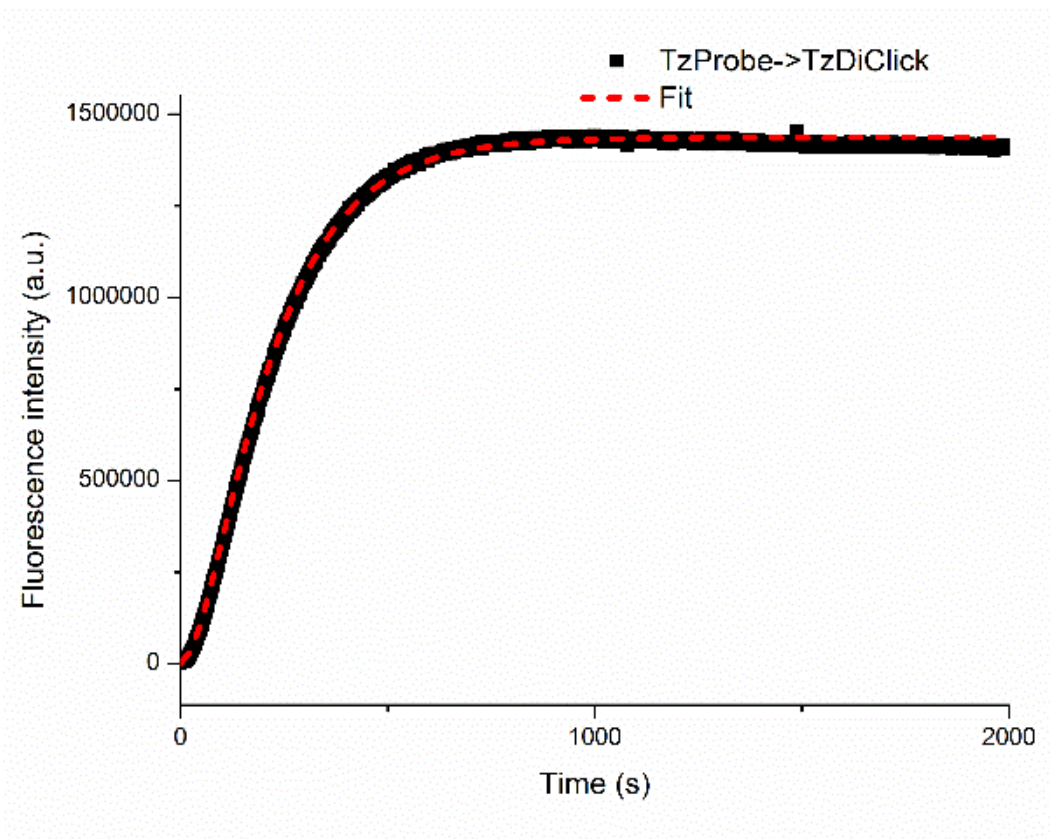

[BCN] (mol.L⁻¹)	k'_2 (s^{-1})	k'_1 (s^{-1})	k_1 ($M^{-1}.s^{-1}$)
5,00E-03	6,19E-03	0,01448	2,90E+00
3,34E-03	4,13E-03	0,00986	2,95E+00
4,16E-03	5,15E-03	0,01064	2,56E+00
5,00E-03	6,19E-03	0,01301	2,60E+00
4,16E-03	5,15E-03	0,01019	2,45E+00
		Average	**2,69E+00**
		Standard Deviation	**0,22**

Click kinetics of the **TzProbe** in DMSO at 37 °C (20 µM probe – 250 eq BCN – Excitation at 395nm – Emission 515nm)

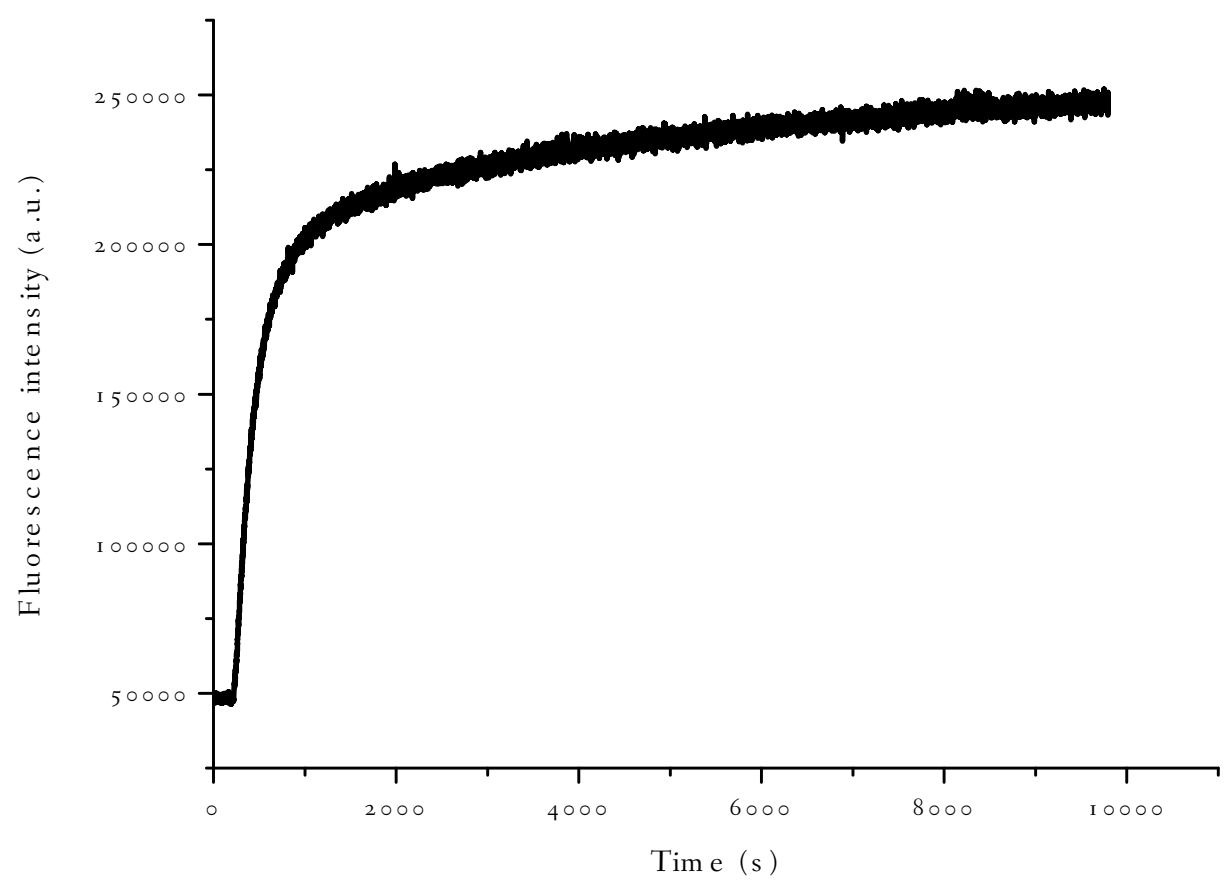

Click kinetics of the **TzProbe** in PBS/BSA(100eq) at 37 °C (2 µM probe – 250 eq BCN – Excitation at 408nm – Emission 500nm)

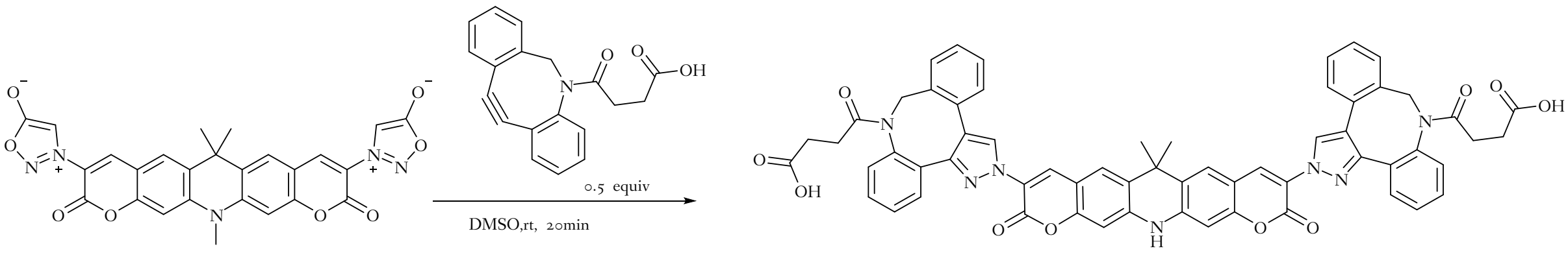

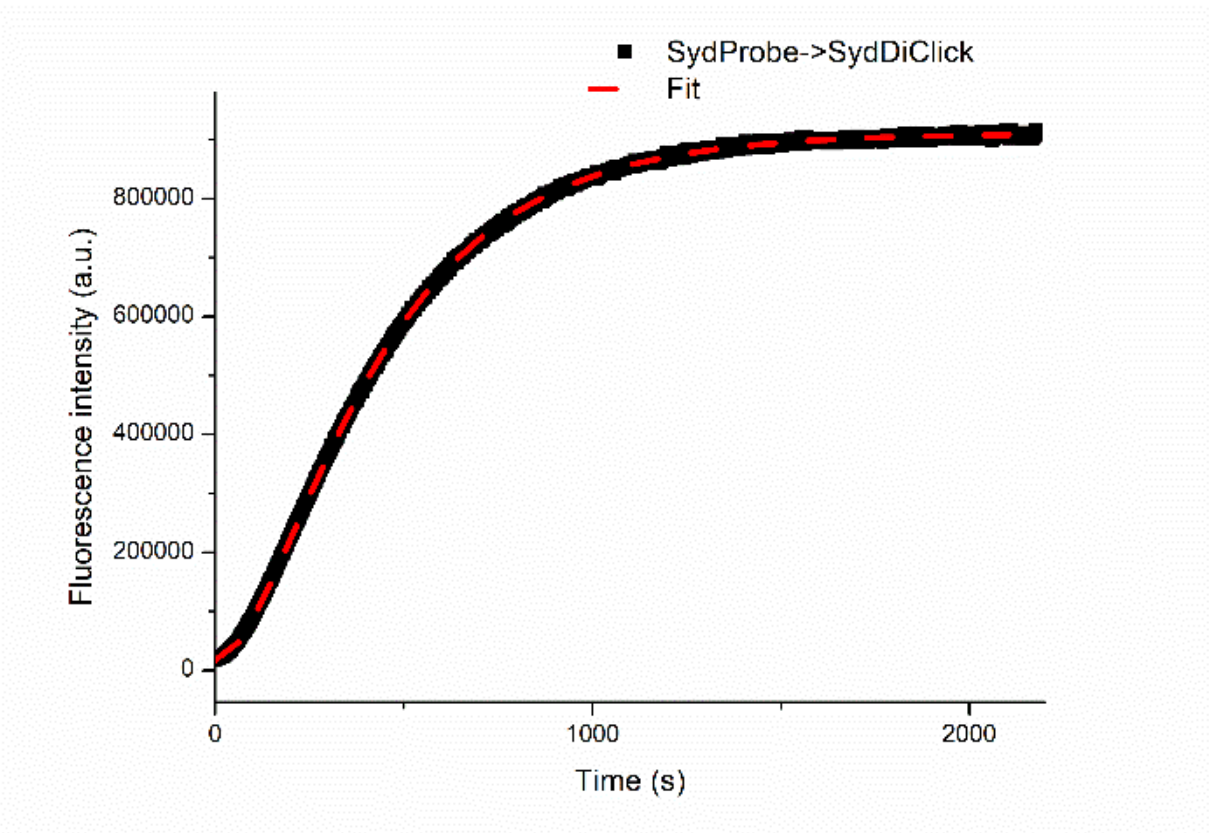

[BCN] (mol.L-1)	k'2 (s-1)	k'1 (s-1)	k2 (M-1.s-1)
5,00E-03	2,45E-03	0,00336	1,01E+00
3,34E-03	3,05E-03	0,00508	1,22E+00
4,16E-03	3,66E-03	0,00531	1,06E+00
		Average	1.10E+00
		Standard Deviation	0,11

Click kinetics of the **SydProbe** in DMSO at 37 °C (20 µM probe – 250 eq BCN – Excitation at 440nm – Emission 500nm)

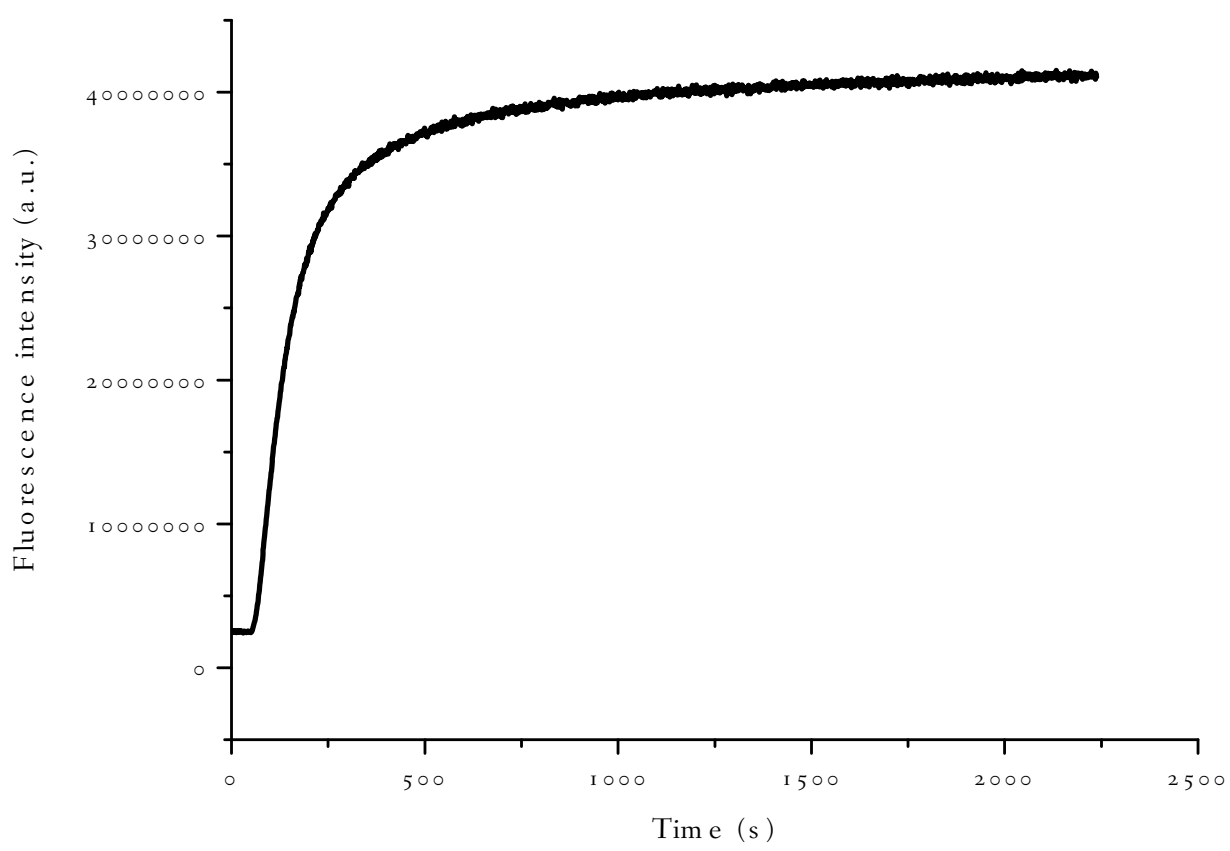

Click kinetics of the **SydProbe** in PBS/BSA(100eq) at 37 °C (10 µM probe – 250 eq BCN – Excitation at 408nm – Emission 500nm)

VI) Molecular modeling:

a) Natural transition orbitals:

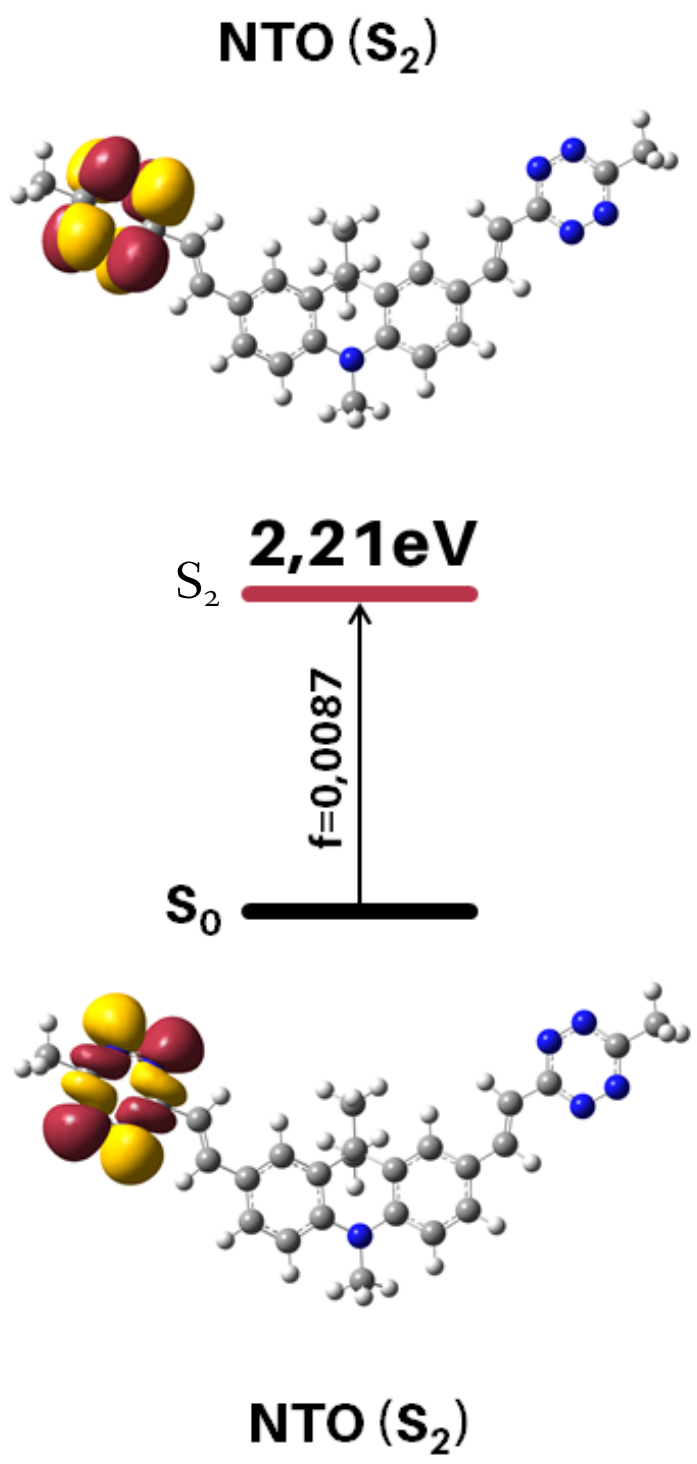

Natural transition orbitals of **TzProbe** (S0->S2)

b) Calculated transitions:

TzProbe

No.	Wavelength (nm)	Osc. Strength	Symmetry	Major contribs
1	561	0,0008	Singlet-A	HOMO-3->LUMO (23%), HOMO-2->LUMO+1 (24%)
2	561	0,0087	Singlet-A	HOMO-3->LUMO+1 (25%), HOMO-2 -> LUMO (23%),
3	396	1,4062	Singlet-A	HOMO>LUMO (14%), HOMO->LUMO+2 (29%)

SydProbe

No.	Wavelength (nm)	Osc. Strength	Symmetry	Major contribs
1	393	1,5831	Singlet-A	HOMO->LUMO (44%), HOMO-3-> LUMO+1 (3%)

TzDiClick

No.	Wavelength (nm)	Osc. Strength	Symmetry	Major contribs
1	354	1,4986	Singlet-A	HOMO->LUMO (42%)

SydDiClick

No.	Wavelength (nm)	Osc. Strength	Symmetry	Major contribs

1	389	2,167	Singlet-A	HOMO->LUMO (42%), HOMO-1->LUMO+1 (6%)

c) Cartesian coordinates of calculated structures:

<u>TzProbe :</u>

Tag	Symbol	X	Y	Z
1	C	-1.23745800	1.39963400	0.47187700
2	C	-1.20321000	2.71201400	-0.05965600
3	C	-2.36056000	3.25566000	-0.63505100
4	C	-3.52820900	2.51013800	-0.70277900
5	C	-3.58200000	1.19680600	-0.21731600
6	C	-2.41210600	0.67116700	0.36518500
7	C	-0.00000500	0.89662200	1.22184600
8	C	1.23741000	1.39958000	0.47176400
9	C	1.20319300	2.71197800	-0.05967900
10	N	0.00000800	3.42951400	-0.01686700
11	C	2.41203200	0.67106200	0.36496200
12	C	3.58192400	1.19670000	-0.21753100
13	C	3.52818500	2.51009300	-0.70283500
14	C	2.36058600	3.25566200	-0.63499300
15	C	-0.00005400	-0.63040100	1.39286200
16	C	0.00008000	1.54034400	2.64100200
17	C	0.00007100	4.85257400	-0.32704800
18	C	-4.82760600	0.45218900	-0.33070400

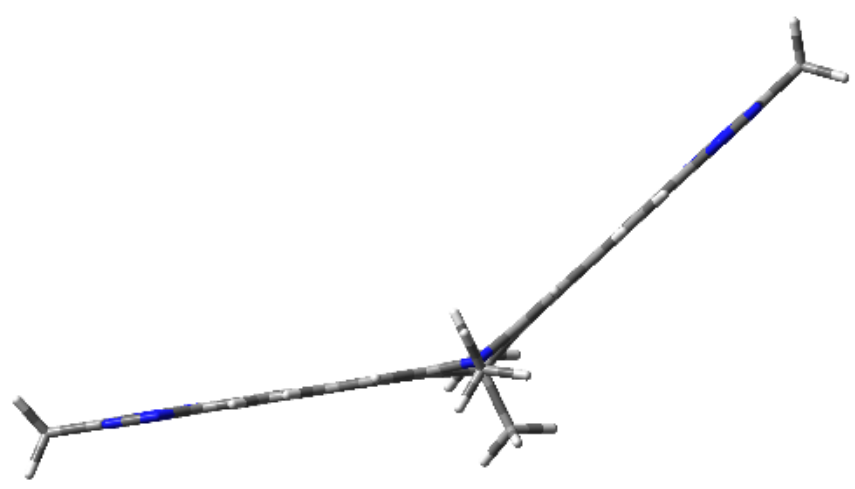

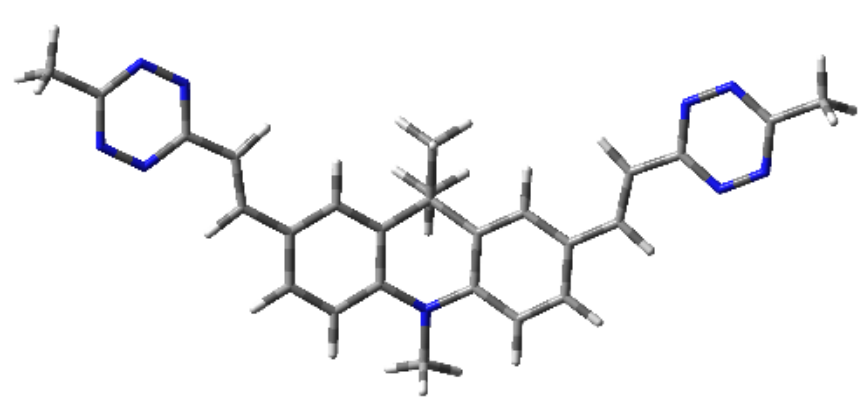

19	C	-5.06348200	-0.81562800	0.06438400
20	C	-6.34530700	-1.48277900	-0.08718300
21	C	4.82749400	0.45203400	-0.33106200
22	C	5.06337200	-0.81581400	0.06392200
23	C	6.34515300	-1.48299400	-0.08775800
24	N	6.42749300	-2.75328700	0.35943200
25	N	7.57684900	-3.38092100	0.23349300
26	C	8.59965800	-2.72432000	-0.33109600
27	N	8.52104700	-1.45430700	-0.77693400
28	N	7.38276900	-0.82392800	-0.65488700
29	N	-6.42768200	-2.75300900	0.36019200
30	N	-7.57691100	-3.38080600	0.23388700
31	C	-8.59954700	-2.72442400	-0.33126200
32	N	-8.52111300	-1.45421700	-0.77659000
33	N	-7.38295100	-0.82368900	-0.65419700
34	C	9.90874300	-3.43342100	-0.47929700
35	C	-9.90817200	-3.43406900	-0.48090300
36	H	-2.34893700	4.24969100	-1.06016800
37	H	-4.41001600	2.94695200	-1.15903500
38	H	-2.43997000	-0.33411600	0.76190700
39	H	2.43981900	-0.33425200	0.76160400
40	H	4.41000900	2.94693500	-1.15903800
41	H	2.34902000	4.24977300	-1.05992000
42	H	-0.00013600	-1.15042500	0.43176200
43	H	0.87254600	-0.95517100	1.96208400
44	H	-0.87260600	-0.95511000	1.96219200
45	H	-0.88816200	1.22553600	3.19533400
46	H	0.88839900	1.22554900	3.19521500
47	H	0.00006500	2.63046200	2.58781700
48	H	-0.88024100	5.31600200	0.11679800
49	H	0.88004200	5.31606700	0.11744000
50	H	0.00045500	5.05612300	-1.40577600
51	H	-5.65398200	0.98928100	-0.78813300

52	H	-4.30004800	-1.42993000	0.52754500
53	H	5.65383000	0.98914800	-0.78854500
54	H	4.29992400	-1.43008100	0.52710700
55	H	9.83010800	-4.44513100	-0.08401000
56	H	10.20091400	-3.47535500	-1.53167400
57	H	10.69549600	-2.89433900	0.05468800
58	H	-9.83185800	-4.44304500	-0.07824000
59	H	-10.69743400	-2.89076800	0.04492900
60	H	-10.19487900	-3.48370100	-1.53450400

<u>TzDiClick:</u>

Tag	Symbol	X	Y	Z
1	C	-1.18142700	3.01482800	0.14612400
2	C	-1.18264800	3.95740200	-0.90954300
3	C	-2.35684300	4.16490000	-1.64509300
4	C	-3.50699600	3.44355600	-1.35285600
5	C	-3.52853400	2.48459800	-0.33299500
6	C	-2.33972700	2.29240800	0.39582800
7	C	0.07779800	2.91299000	1.01348800
8	C	1.29342200	3.04675400	0.09015400
9	C	1.22290700	3.98789900	-0.96389300
10	N	0.00492300	4.64377700	-1.20617700
11	C	2.48011900	2.35392000	0.28614000
12	C	3.62828100	2.57652100	-0.49588800
13	C	3.53709800	3.53320100	-1.51288700
14	C	2.35605300	4.22440400	-1.75238500

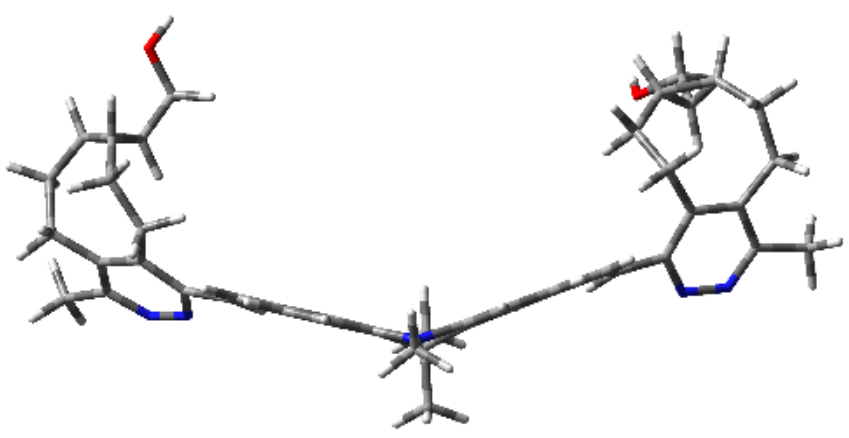

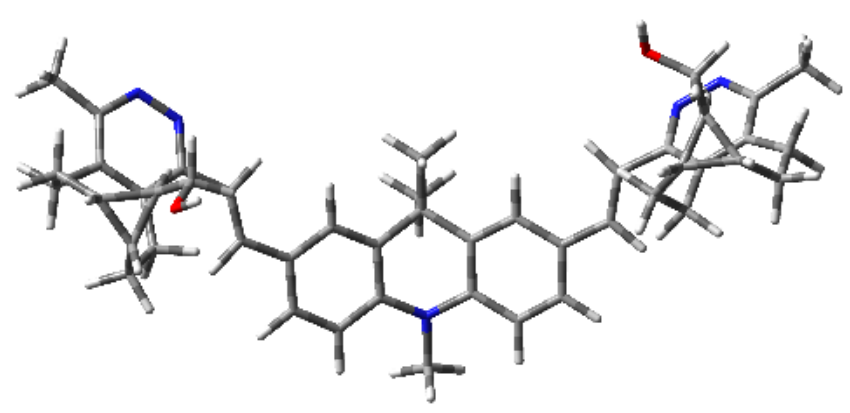

15	C	0.11340700	1.62192600	1.84574100
16	C	0.08449900	4.11994400	1.99933700
17	C	-0.03003600	5.77593400	-2.11794100
18	C	-4.76680600	1.74513600	-0.07845900
19	C	-4.98272100	0.86215300	0.91590700
20	C	-6.20632100	0.13894700	1.29753100
21	C	4.89473400	1.86788500	-0.29218900
22	C	5.19170100	0.99543400	0.68406700
23	C	6.50688300	0.36069200	0.93655200
24	N	6.87285400	0.48794000	2.21871100
25	N	8.05200500	0.05310600	2.62623400
26	C	8.87948300	-0.55265400	1.77301400
27	C	8.52726900	-0.82661300	0.42683800
28	C	7.29443300	-0.34209000	-0.01542200
29	C	-7.28722400	-0.26061400	0.45584100
30	C	-8.37125000	-0.86870700	1.09213000
31	C	-8.29009400	-1.06745300	2.48983500
32	N	-7.20282500	-0.74872500	3.19674100
33	N	-6.18020100	-0.16084300	2.61027500
34	C	10.20640700	-0.94123900	2.37450300
35	C	-9.40991300	-1.66129900	3.30670600
36	C	8.13309100	-3.50378300	-1.55879300
37	C	-7.83099200	-2.37652700	-2.08987400
38	C	-8.61025500	-3.27561000	-1.14501500
39	C	-7.10779900	-3.18092900	-1.03284600
40	C	6.63784400	-3.25685000	-1.50040000
41	C	7.35439800	-3.81518300	-0.29522700
42	C	9.55096900	-1.60874300	-0.38940600
43	C	9.21176400	-2.42458500	-1.65367800
44	C	-7.16130900	-0.02526200	-1.04336600
45	C	-7.90830500	-0.85054000	-2.11687200
46	C	7.12905300	-5.24252500	0.12035600
47	C	-6.21432000	-4.34625000	-1.39620500

48	C	-9.60374500	-2.82207500	-0.08201200
49	C	-9.61212500	-1.34001400	0.36608300
50	O	5.90665600	-5.31238600	0.86271300
51	C	5.93589700	-1.90549900	-1.56211500
52	C	6.75724700	-0.60242500	-1.40720100
53	O	-6.76031900	-5.04091000	-2.52149200
54	H	-2.37425000	4.86757900	-2.46703100
55	H	-4.40243700	3.61956700	-1.94026300
56	H	-2.33517800	1.56219600	1.19282800
57	H	2.53156500	1.62392500	1.08192400
58	H	4.40067000	3.73234900	-2.13927800
59	H	2.31810400	4.92672000	-2.57395200
60	H	0.11120500	0.72848200	1.21633400
61	H	1.00144000	1.59621400	2.47954700
62	H	-0.74415100	1.57271700	2.51887400
63	H	0.98777800	4.09584700	2.61493200
64	H	0.05958200	5.07223000	1.46676300
65	H	-0.78842500	4.07211000	2.65601300
66	H	-0.91365100	6.37835600	-1.90833700
67	H	0.84692900	6.40021100	-1.94829700
68	H	-0.05035200	5.47851300	-3.17550300
69	H	-5.56902100	1.99150200	-0.76390300
70	H	-4.19614100	0.69179700	1.64382000
71	H	5.66857300	2.12849800	-1.01064200
72	H	4.47006300	0.79820300	1.47064400
73	H	10.26115400	-0.52915900	3.38123800
74	H	10.32245000	-2.02828500	2.44204100
75	H	11.04860400	-0.56126900	1.78852700
76	H	-9.71961500	-2.64320900	2.93995300
77	H	-9.06814200	-1.76399200	4.33586400
78	H	-10.29521300	-1.01648000	3.30018600
79	H	8.39317500	-4.37989300	-2.14760300
80	H	-7.75189200	-2.80167200	-3.08513200

81	H	-8.93775200	-4.19089200	-1.62906300
82	H	-6.72172600	-2.62769100	-0.18467000
83	H	6.06611200	-4.01182100	-2.03515200
84	H	7.48815800	-3.13726900	0.53972900
85	H	10.34852800	-0.90510900	-0.66520200
86	H	10.02770300	-2.30690500	0.30263300
87	H	10.14729200	-2.93660900	-1.90513000
88	H	9.01655900	-1.77514100	-2.50942800
89	H	-7.40995400	1.02600600	-1.23752000
90	H	-6.09572900	-0.10897100	-1.26950500
91	H	-7.45204500	-0.53162200	-3.06052300
92	H	-8.95135600	-0.53674300	-2.20103500
93	H	7.96884000	-5.59344600	0.73656800
94	H	7.07422200	-5.88389100	-0.77108600
95	H	-5.20694300	-3.97547500	-1.63219400
96	H	-6.13222200	-5.02437400	-0.53508800
97	H	-9.48483700	-3.45916500	0.80238700
98	H	-10.60880600	-3.03369900	-0.46498400
99	H	-9.82044800	-0.69617500	-0.48487200
100	H	-10.46479500	-1.21867000	1.03799000
101	H	5.81299300	-6.19869100	1.22487500
102	H	5.12211600	-1.89406500	-0.82835400
103	H	5.45258400	-1.84283500	-2.54361900
104	H	7.56329500	-0.57429400	-2.13675100
105	H	6.08469500	0.20510000	-1.68029100
106	H	-6.18949700	-5.78482100	-2.73120900

Sydprobe :

Tag	Symbol	X	Y	Z
1	C	1.23580400	0.05814100	1.02223200
2	C	1.20289500	1.39400800	0.53176200
3	C	2.34881400	1.97840200	-0.01845300
4	C	3.51732900	1.23660400	-0.10032600
5	C	3.57515900	-0.09347800	0.33732500

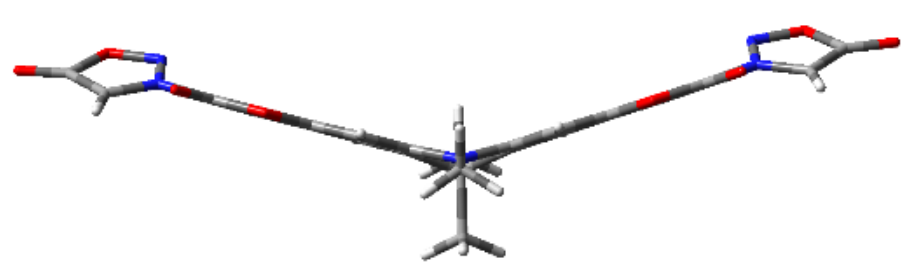

6	C	2.41066300	-0.65669400	0.89729600
7	C	-0.00004500	-0.47297900	1.75119300
8	C	-1.23581100	0.05816000	1.02214100
9	C	-1.20286400	1.39404300	0.53169100
10	N	0.00000400	2.10500100	0.59029800
11	C	-2.41064900	-0.65668500	0.89715400
12	C	-3.57513100	-0.09347100	0.33715000
13	C	-3.51732000	1.23665000	-0.10038400
14	C	-2.34879700	1.97844000	-0.01851500
15	C	-0.00009900	0.11185800	3.19596100
16	C	-0.00009000	-2.00613100	1.85469900
17	C	0.00004400	3.54486500	0.34101300
18	C	-4.81151600	-0.78623900	0.19600600
19	C	-5.88676500	-0.17027400	-0.36012200
20	C	-5.83960400	1.21976000	-0.81146700
21	O	-4.60969700	1.84487700	-0.64165000
22	O	4.60970000	1.84480900	-0.64162700
23	C	5.83947300	1.21959100	-0.81181300
24	C	5.88676500	-0.17032600	-0.36005400
25	C	4.81158100	-0.78620800	0.19630400
26	N	-7.10570300	-0.89349800	-0.54233300
27	N	-7.00543800	-2.19692500	-0.71245200
28	O	-8.28217300	-2.63445200	-0.78167400
29	C	-9.22853400	-1.54171900	-0.66571400
30	C	-8.36950400	-0.42336300	-0.50616800
31	N	7.10572300	-0.89350600	-0.54210100
32	N	7.00559000	-2.19701300	-0.71165300
33	O	8.28231700	-2.63442100	-0.78105800
34	C	9.22862000	-1.54151200	-0.66576000
35	C	8.36952000	-0.42320600	-0.50650300
36	O	-6.73537900	1.85746800	-1.29317200
37	O	6.73509500	1.85708000	-1.29409000
38	O	-10.41237000	-1.73348000	-0.70838600

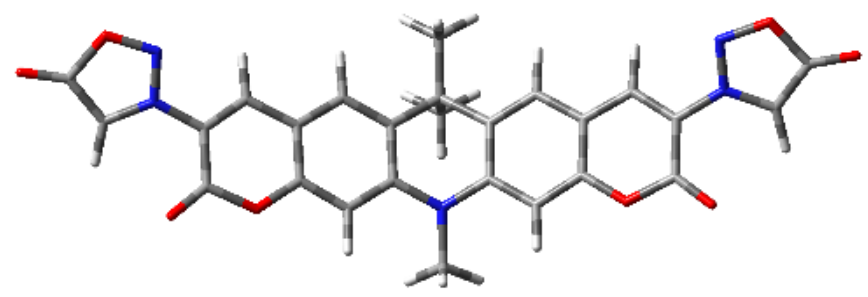

39	O	10.41245800	-1.73320400	-0.70867200
40	H	2.35284100	2.98129800	-0.41875200
41	H	2.46709600	-1.67809700	1.24998000
42	H	-2.46704900	-1.67810300	1.24980700
43	H	-2.35285900	2.98136000	-0.41874400
44	H	-0.88765400	-0.22617300	3.73690300
45	H	0.88739100	-0.22619300	3.73700000
46	H	-0.00008800	1.20333400	3.19080700
47	H	0.87144400	-2.35573400	2.41046800
48	H	-0.87161600	-2.35565600	2.41053300
49	H	-0.00014400	-2.48372500	0.87193500
50	H	-0.87924300	3.98730400	0.80623300
51	H	0.87945000	3.98722800	0.80607700
52	H	-0.00004500	3.78931100	-0.72759200
53	H	-4.90026100	-1.81552900	0.52311100
54	H	4.90040700	-1.81540800	0.52365800
55	H	-8.60528000	0.61864100	-0.42007900
56	H	8.60514300	0.61887300	-0.42091500

SydDiClick :

Tag	Symbol	X	Y	Z
1	C	-0.91557000	-1.91392800	0.26225800
2	C	-0.96602700	-2.80972100	-0.84093700
3	C	-2.08172800	-2.83086500	-1.68397200
4	C	-3.13334300	-1.95923000	-1.44040200
5	C	-3.10402600	-1.03897800	-0.38775800
6	C	-1.97144800	-1.04136400	0.45075900
7	C	0.26021300	-2.03729800	1.23449200
8	C	1.51833600	-2.32813900	0.41263000
9	C	1.40241400	-3.21187800	-0.69454000
10	N	0.12751900	-3.65298500	-1.07528600
11	C	2.77267800	-1.84863000	0.74371500
12	C	3.93417500	-2.23609200	0.04668400
13	C	3.78392700	-3.13295000	-1.01572400
14	C	2.54057400	-3.61482800	-1.39982300
15	C	-0.00544700	-3.25815100	2.16584200
16	C	0.41778100	-0.78952100	2.11734700
17	C	0.00156000	-4.74339600	-2.03487200
18	C	5.24952800	-1.77462200	0.35425700
19	C	6.32786600	-2.19974700	-0.35911200
20	C	6.16863900	-3.14575300	-1.46830800
21	O	4.86905600	-3.54821000	-1.73127300
22	O	-4.20488900	-2.01275500	-2.28456400
23	C	-5.31115300	-1.18766700	-2.18074500
24	C	-5.28546500	-0.22686800	-1.07501700
25	C	-4.22476200	-0.17150100	-0.22339000
26	N	7.62962100	-1.76819100	-0.02842800
27	N	7.82153100	-1.12639600	1.15527200
28	C	9.09822800	-0.75146000	1.16062700
29	C	9.75227200	-1.16157600	-0.04387100
30	C	8.78026300	-1.80695100	-0.77152100
31	N	-6.37692800	0.65603800	-0.92700000
32	N	-6.25299400	1.70627200	-0.07130400

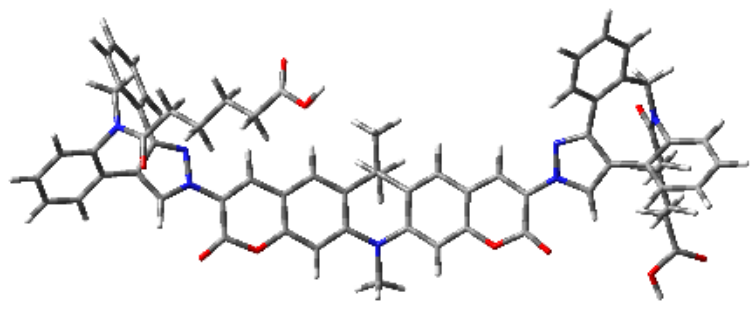

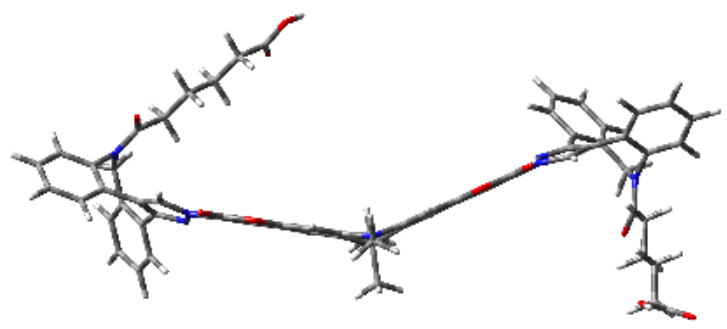

33	C	-7.44198500	2.29803400	-0.04851300
34	C	-8.36057200	1.62398200	-0.91424000
35	C	-7.63829700	0.58657500	-1.45833200
36	O	7.04129800	-3.60165900	-2.16326100
37	O	-6.18909600	-1.33175300	-2.99579800
38	C	-9.76384400	1.97619500	-1.16764100
39	C	-10.63628100	2.13883600	-0.07841400
40	N	-10.14409000	1.90644200	1.24883900
41	C	-9.72165300	3.05493900	2.04774000
42	C	-8.66196000	3.98039400	1.46720700
43	C	-7.59568300	3.61136400	0.62184300
44	C	-10.26788300	2.15069200	-2.46306300
45	C	-11.59914300	2.50157500	-2.66750400
46	C	-12.44870000	2.69200300	-1.57821100
47	C	-11.96531500	2.50801400	-0.28527200
48	C	-8.70263700	5.29649500	1.94903400
49	C	-7.71753000	6.22806100	1.64736600
50	C	-6.64977400	5.85429100	0.83552500
51	C	-6.60257700	4.56348600	0.32818000
52	C	-10.13889600	0.65203700	1.83734100
53	O	-9.73993400	0.48211600	2.97954600
54	C	-10.65561500	-0.50470000	0.98759300
55	C	-10.69606600	-1.82622100	1.75580000
56	C	-11.19754800	-2.98379500	0.88641900
57	C	-11.25303200	-4.31193100	1.66879200
58	C	-11.77300700	-5.44105000	0.81519700
59	O	-12.90097400	-5.86858800	0.81475200
60	O	-10.81435800	-5.93357100	-0.01395800
61	C	11.14531100	-0.92583300	-0.44335700
62	C	11.65357800	0.38003400	-0.40477600
63	N	10.78769500	1.44606700	0.02300000
64	C	10.92011600	1.88466800	1.40457800
65	C	10.53918500	0.90573600	2.51582900

66	C	9.67648300	-0.20337600	2.41309500
67	C	11.98087900	-1.95653800	-0.88947400
68	C	13.29252700	-1.69160400	-1.27322900
69	C	13.78974100	-0.39067800	-1.21659500
70	C	12.96497600	0.64609000	-0.78636300
71	C	9.89305600	1.96540100	-0.89755400
72	C	11.04736100	1.24557100	3.77807300
73	C	10.70816400	0.54530200	4.92725900
74	C	9.83653700	-0.53737100	4.83151500
75	C	9.33973800	-0.90207000	3.58930100
76	O	9.87976200	1.57111500	-2.05121800
77	C	8.92215700	3.04270500	-0.41213500
78	C	7.91630000	3.44642500	-1.49175500
79	C	6.95297300	4.53473500	-1.00721600
80	C	5.95289100	4.94148800	-2.08896300
81	C	4.96911500	5.99563700	-1.64144700
82	O	4.89207900	6.48282300	-0.54118000
83	O	4.13494400	6.35582000	-2.65185200
84	H	-2.14572500	-3.48112800	-2.54398500
85	H	-1.95864200	-0.34146000	1.27595800
86	H	2.89234000	-1.16742400	1.57602600
87	H	2.48876300	-4.26533700	-2.26050400
88	H	0.83144800	-3.39200800	2.85642100
89	H	-0.91634000	-3.09492000	2.74792100
90	H	-0.12661900	-4.18045000	1.59497800
91	H	-0.47815800	-0.62642900	2.71872600
92	H	1.23919200	-0.91837700	2.82420400
93	H	0.60694900	0.10937900	1.52549500
94	H	-0.97780000	-5.20674300	-1.92287700
95	H	0.12087400	-4.41305200	-3.07440700
96	H	0.75462700	-5.49959000	-1.81728100
97	H	5.40695800	-1.08180800	1.17113400
98	H	-4.24152700	0.55235600	0.58152200

99	H	8.80726100	-2.26519500	-1.74331500
100	H	-7.90569600	-0.18015400	-2.16311400
101	H	-9.35357700	2.63702000	2.98728000
102	H	-10.60055900	3.66310100	2.29128100
103	H	-9.60127100	2.02886300	-3.30919500
104	H	-11.97071700	2.63835600	-3.67665300
105	H	-13.48313200	2.97548400	-1.73440000
106	H	-12.61932600	2.63173700	0.57093200
107	H	-9.52657400	5.58811200	2.59274300
108	H	-7.78565300	7.23593200	2.04103300
109	H	-5.86960700	6.56440700	0.58633800
110	H	-5.79025000	4.26663000	-0.32346200
111	H	-11.64997800	-0.25306800	0.60430800
112	H	-10.01469200	-0.59708600	0.10404700
113	H	-9.69737200	-2.05180200	2.14060700
114	H	-11.33736400	-1.71454500	2.63541700
115	H	-12.19834500	-2.75557400	0.50238100
116	H	-10.54519600	-3.10734700	0.01559000
117	H	-10.25297900	-4.56729300	2.02958500
118	H	-11.92110100	-4.21619600	2.52624900
119	H	-11.23056500	-6.62495100	-0.55061000
120	H	10.32857400	2.79025700	1.53696400
121	H	11.96207200	2.17975100	1.57217800
122	H	11.59888200	-2.97060800	-0.91982900
123	H	13.92804000	-2.50176600	-1.61239000
124	H	14.81001500	-0.18168000	-1.51694300
125	H	13.32739100	1.66784400	-0.76659100
126	H	11.72132000	2.09368500	3.85552100
127	H	11.12281500	0.83874400	5.88498400
128	H	9.55868200	-1.10235100	5.71391900
129	H	8.67901600	-1.75493100	3.49949600
130	H	8.39350300	2.68214300	0.47733200
131	H	9.49266600	3.92342000	-0.09258000

132	H	8.45945900	3.78669400	-2.37807400
133	H	7.35642600	2.56122500	-1.80882700
134	H	6.40582700	4.19017800	-0.12368500
135	H	7.51342700	5.41931700	-0.68642800
136	H	6.46312300	5.32163300	-2.98086100
137	H	5.37133000	4.07896900	-2.43477400
138	H	3.53272800	7.02773100	-2.29848200

VII) Fluorescence microscopy:

Mitochondrial colocalization experiments.

A549 lung carcinoma cells were cultured in Dulbecco's modified eagle media (DMEM, GIBCO) supplemented with 10% Fetal Bovine Serum (FBS, GIBCO) and 1% Penicillin/Streptomycin (Life Technologies) at 37 °C under 5% CO_2, 95% air and 100% humidity. cells were seeded on a 8-well μ-slide from Ibidi (30 000 cells/well). After 24 h, cells were incubated 1h with MitoTracker® Deep-Red FM at 20 nM, washed with PBS twice, incubated 30 min with **DBCO-TPP** at 50 μM washed twice with PBS twice and replaced by RPMI medium 1640 w/o -glutamine and Phenol Red (GIBCO). After, cells were incubated 24 h with **SydProbe** at 2 μM (see Fig. 5 and Fig. S40). For control experiment (see Fig. S41) cells were incubated 30 min with **DBCO-TPP** at 50 μM washed twice with PBS twice and replaced by RPMI medium 1640 w/o -glutamine and Phenol Red (GIBCO), followed by incubation 24 h with with **SydProbe** at 2 μM. For control experiment (see Fig. S42) cells were incubated 1h with MitoTracker® Deep-Red FM at 20 nM, washed with PBS twice and replaced by RPMI medium 1640 w/o -glutamine and Phenol Red (GIBCO).

Images were acquired with a Leica SP8X inverted confocal laser scanning microscope (CLSM), equipped with a 63x Oil immersion objective (NA= 1.4). Sequential excitation mode (635 nm and 442 nm obtained with a white light laser (WLL) and a 442 nm diode) was used to collect images on Hybrid detectors. Compounds emissions were detected respectively: MitoTracker® Deep-Red FM at 650-750 nm upon excitation at 635 nm and **SydProbe + DBCO-TPP** at 460-600 nm upon excitation at 442 nm. The brightfield image was acquired using a transmission PMT to visualize cells. The whole system was driven by LAS X software (Leica). The images were visualized and processed using ImageJ software (Rasband W.S., U.S. National Institutes of Health, Bethesda, Maryland, USA).

Fluorescence exaltation (1PE or 2PE), control (1PE or 2PE) and fluorescence spectra measurement experiments.

A549 lung carcinoma cells were cultured in Dulbecco's modified eagle media (DMEM, GIBCO) supplemented with 10% Fetal Bovine Serum (FBS, GIBCO) and 1% Penicillin/Streptomycin (Life Technologies) at 37 °C under 5% CO_2, 95% air and 100% humidity. cells were seeded on a 8-well μ-slide from Ibidi (30 000 cells/well). After 24 h, cells were incubated 30 min with **DBCO-TPP** at 50 μM washed twice with PBS twice and replaced by RPMI medium 1640 w/o -glutamine and Phenol Red (GIBCO). After, cells were incubated 24 h with with **SydProbe** at 2 μM (see Fig. 4 C and F, Fig. S43 and Fig. S44 A and B). For

control experiments (see Fig. 4 A and D, and Fig. S44 E et F) cells were incubated 30 min with **DBCO-TPP** at 50 µM washed twice with PBS twice and replaced by RPMI medium 1640 w/o -glutamine and Phenol Red (GIBCO). For control experiment (see Fig. S41 B and D and Fig. S44 C and D) cells were washed twice with PBS twice and replaced by RPMI medium 1640 w/o -glutamine and Phenol Red (GIBCO), followed by incubation 24 h with **SydProbe** at 2 µM. For control experiments (see Fig. S44 G and H) cells were washed twice with PBS twice and replaced by RPMI medium 1640 w/o -glutamine and Phenol Red (GIBCO), followed by incubation 24 h with vehicle (DMSO).

Images (Fig. S43 and Fig. S11) were acquired with a Leica SP8X inverted confocal laser scanning microscope (CLSM), equipped with a 63x Oil immersion objective (NA= 1.4). Excitation mode (442 nm obtained with a 442 nm diode) was used to collect images on Hybrid detectors. Compounds emissions were detected respectively: at 460-700 nm upon excitation at 442 nm. The brightfield image was acquired using a transmission PMT to visualize cells. The whole system was driven by LAS X software (Leica).

For two-photon microscopy experiment (Fig. 4 and Fig. S45), images were acquired with a Zeiss LSM980 Airyscan 2 inverted confocal laser scanning microscope (CLSM), used in confocal mode and equipped with a 63x NA= 1.4 Oil objective. Sequential excitation mode was used with a 445 nm diode and a Chameleon Vision II (COHERENT) laser tuned at 760 nm to collect emission of our developed **SydProbe** + **DBCO-TPP** on the 32 channels spectral GaAsP detector, bandwidth adjusted from 460 to 600 nm.

The images were visualized and processed using ImageJ software (Rasband W.S., U.S. National Institutes of Health, Bethesda, Maryland, USA).

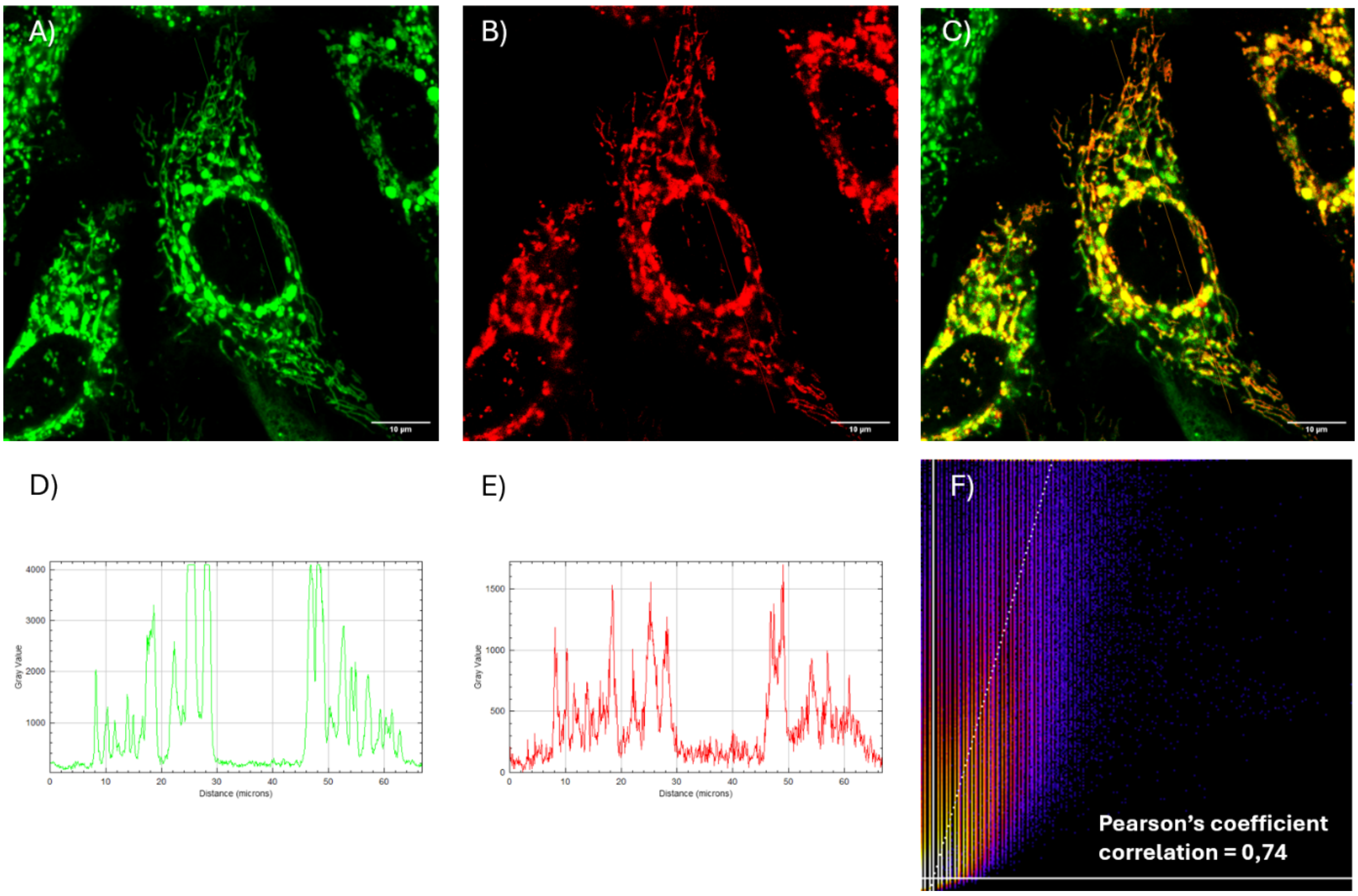

*Fig. S40: **Fluorescence emission from SydProbe + DBCO-TPP colocalized to mitochondria in live A549 cells.** A) A549 cells were incubated with with **DBCO-TPP** (50 µM) for 30 min and then **SydProbe** (2 µM) for 24 hours (λ_{ex} =442 nm, λ_{em}= 460-600 nm, laser =1%, gain = 20%). B) A549 cells were incubated with Mito-Tracker Deep-Red FM (20 nM) for 1 hour (λ_{ex} = 635 nm, λ_{em}= 650-750 nm, laser =2%, gain = 100%). C) Merge image. D) Intensity profile showed the fluorescence intensity along the line across the cell from image A) recorded with **Sydprobe** + **DBCO-TPP**. E) Intensity*

profile showed the fluorescence intensity along the line across the cell from image B) recorded with Mito-Tracker Deep-Ref FM. F) Pearson's colocalization coefficient scattering plot. Data settings and processing were identical for all images used for colocalization experiments (Fig 5, Fig. S40, Fig. S41 and Fig. S42).

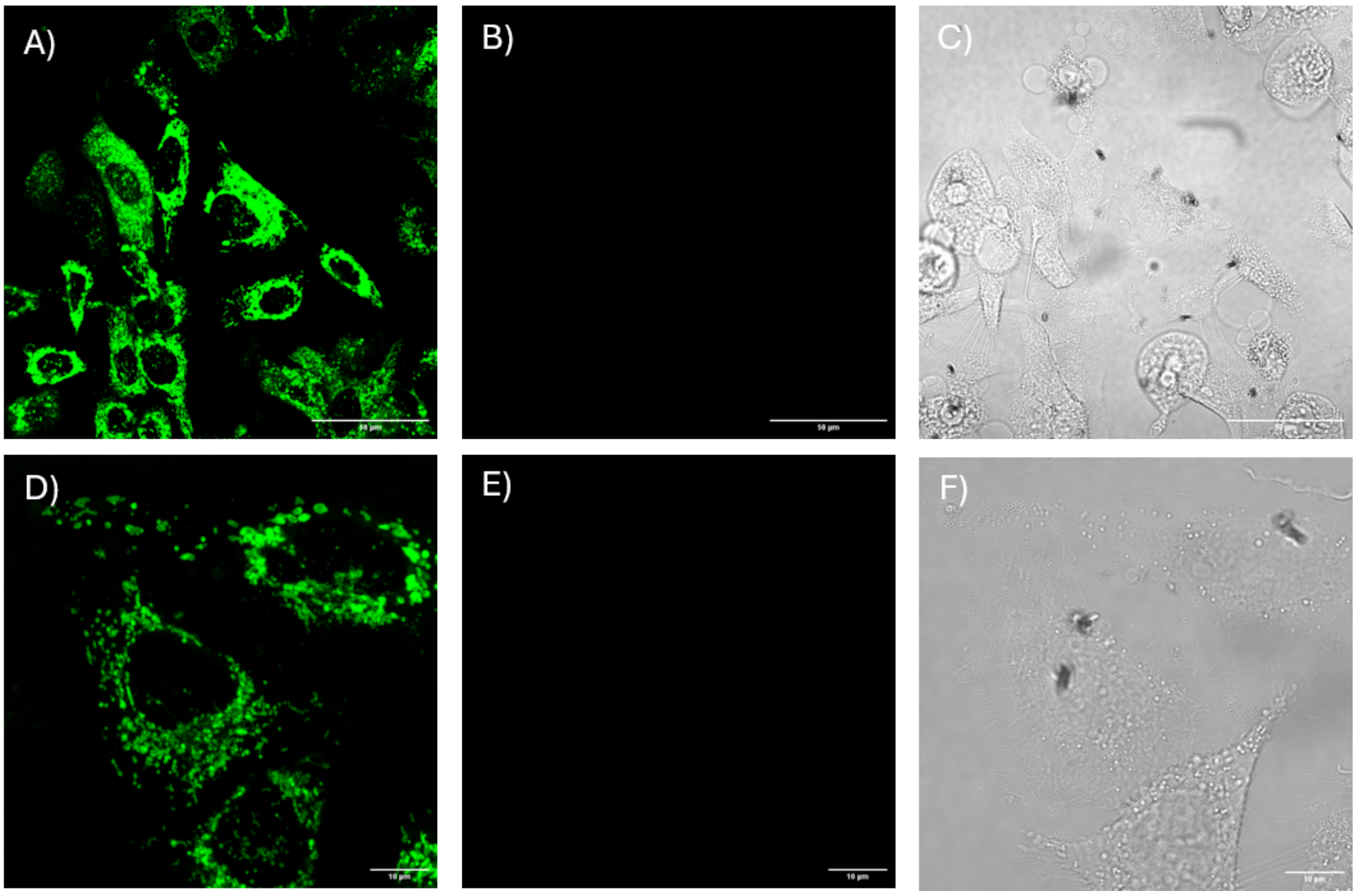

Fig. S41: ***No overlap control experiment: Fluorescence emission from SydProbe + DBCO-TPP in live A549 cells.*** *A) and D) A549 cells were incubated with with* ***DBCO-TPP*** *(50 µM) for 30 min and then* ***SydProbe*** *(2 µM) for 24 hours ($\square_{ex}$ = 442 nm, $\square_{em}$= 460-600 nm, laser =1%, gain = 20%). B) and E) A549 cells were incubated with with* ***DBCO-TPP*** *(50 µM) for 30 min and then* ***SydProbe*** *(2 µM) for 2 hours ($\square_{ex}$ = 635 nm, $\square_{em}$= 660-750 nm). C) and F) A549 cells were incubated with with* ***DBCO-TPP*** *(50 µM) for 30 min and then* ***SydProbe*** *(2 µM) for 2 hours (Transmission: $\square_{ex}$ = 442, laser =4%, gain = 400V, offset = 2%). Data settings and processing were identical for all images used for colocalization experiments (Fig 5, Fig. S40, Fig. S41 and Fig. S42).*

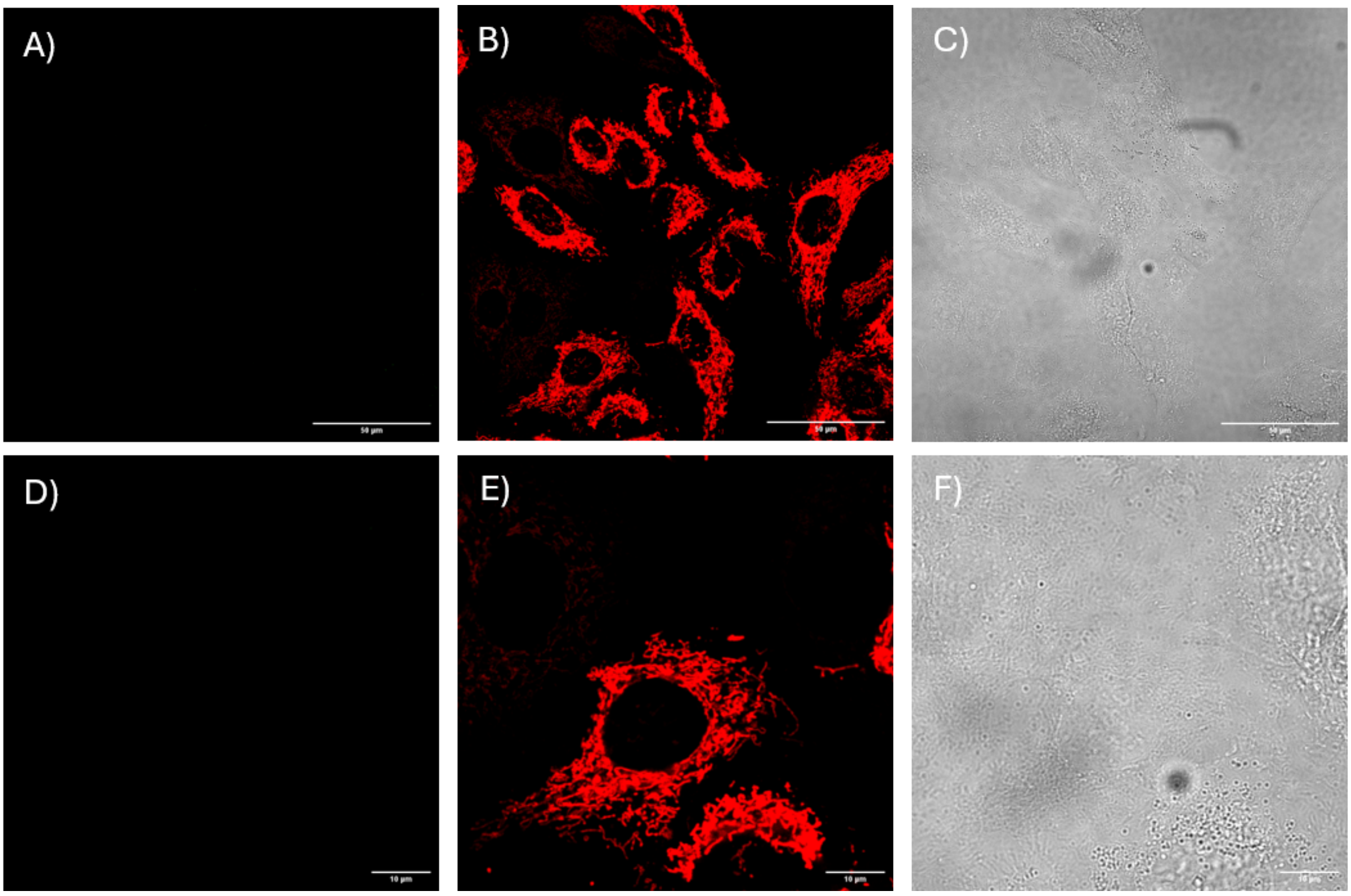

Fig. S42: ***No overlap control experiment: Fluorescence emission from Mito-Tracker Deep-Red FM in live A549 cells.*** *A) and D) A549 cells were incubated with Mito-Tracker Deep-Red FM (20 nM) for 1 hour (λ_{ex} = 442 nm, λ_{em}= 460-600 nm, laser =1%, gain = 20%). B) and E) A549 cells were incubated with Mito-Tracker Deep-Red FM (20 nM) for 1 hour (λ_{ex} = 635 nm, λ_{em}= 650-750 nm, laser =2%, gain = 100%). C) and F) A549 cells were incubated with Mito-Tracker Deep-Red FM (20 nM) for 1 hour (Transmission: λ_{ex} = 442, laser =4%, gain = 400V, offset = 2%). Data settings and processing were identical for all images used for colocalization experiments (Fig 5, Fig. S40, Fig. S41 and Fig. S42).*

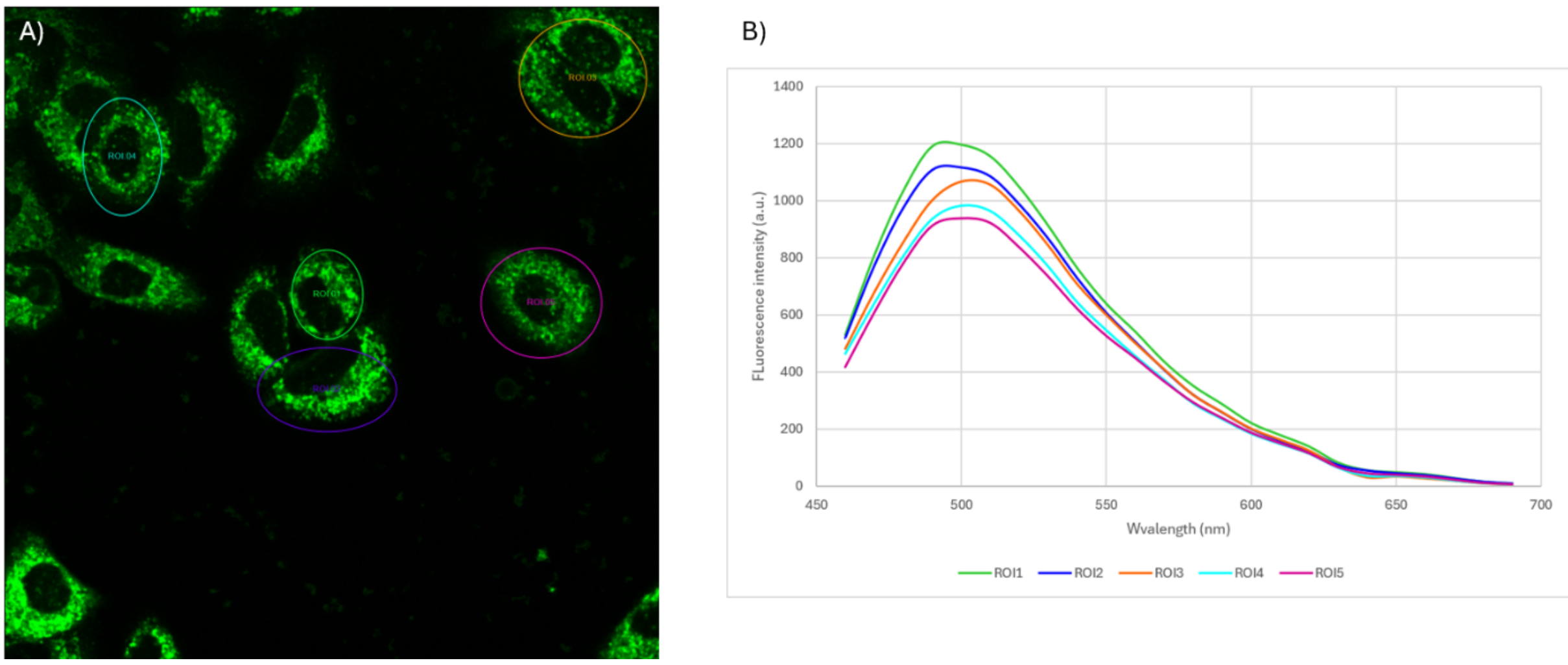

Fig. S43: ***Fluorescence emission spectra from SydProbe in live A549 cells.*** *A) A549 cells were incubated with with* ***DBCO-TPP*** *(50 µM) for 30 min and then* ***SydProbe*** *(2 µM) for 24 hours (λ_{ex} = 442 nm, λ_{em}= 460-700 nm, laser 1%, gain = 100%, bandwidth and step size = 10 nm). B) Fluorescence spectra extracted from each ROI.*

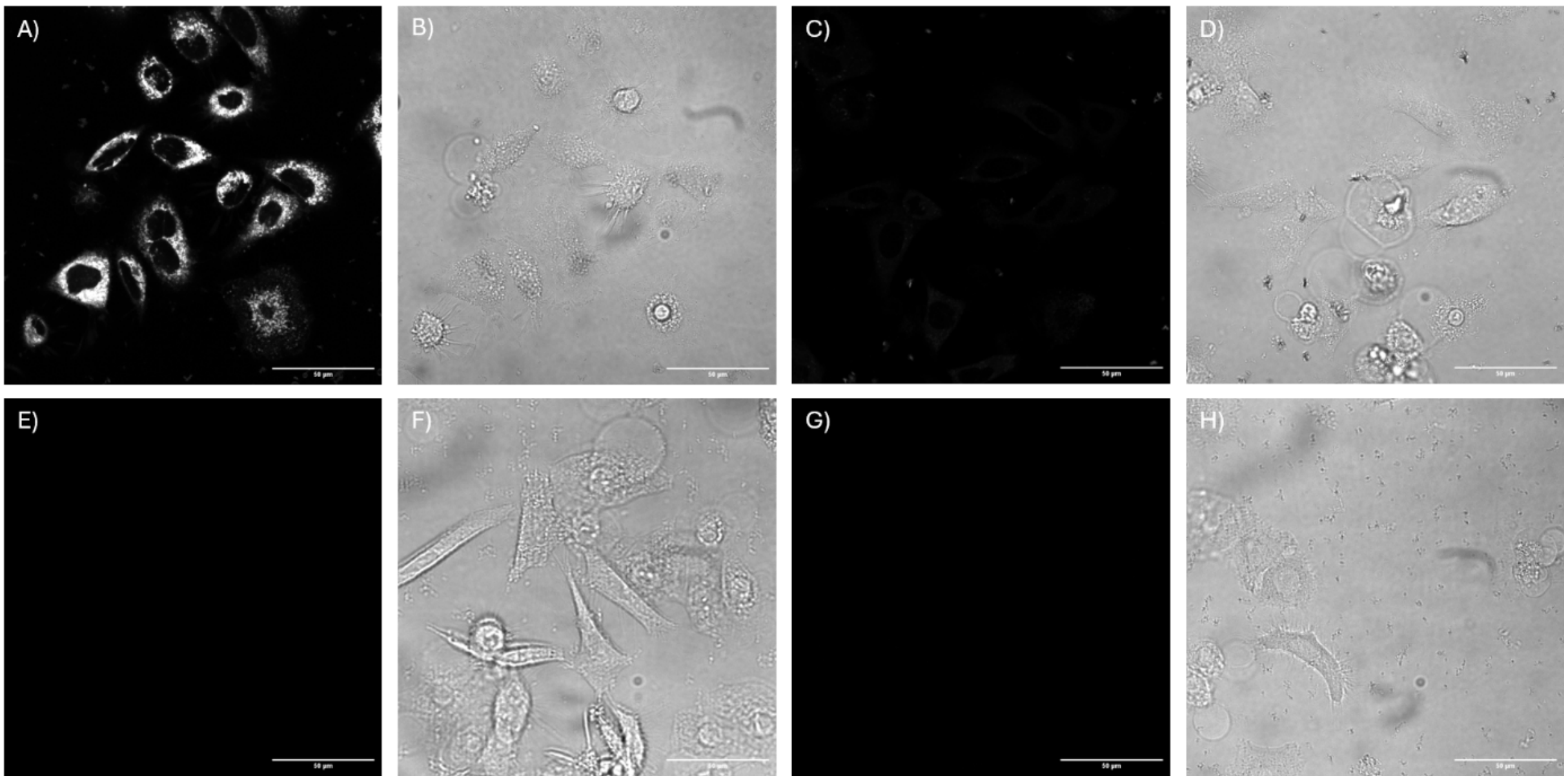

Fig. S44: ***Fluorescence emission from SydProbe with and without DBCO-TPP and control experiments in live A549 cells.*** *A) and B) A549 cells were co-incubated with* ***DBCO-TPP*** *(50 μM) for 30 min and then* ***SydProbe*** *(2 μM) for 24 hours. C) and D) A549 cells were incubated* ***SydProbe*** *(2 μM) for 24 hours. E) and F) A549 cells were incubated with* ***DBCO-TPP*** *(50 μM) for 30 min. G) and H) A549 cells were incubated with vehicle (DMSO) for 30 min. Fluorescence images (A), C), E) and G)) were collected with the following parameters:* l_{ex} *= 442 nm,* l_{em}*= 460-700 nm, laser =1%, gain = 20%). Transmission images (B), D), F) and H) were collected with the following parameters:* l_{ex} *= 442 + 535 nm, laser =1% + 10%, gain = 200V, offset = 2%). Data settings and processing were identical for all images.*

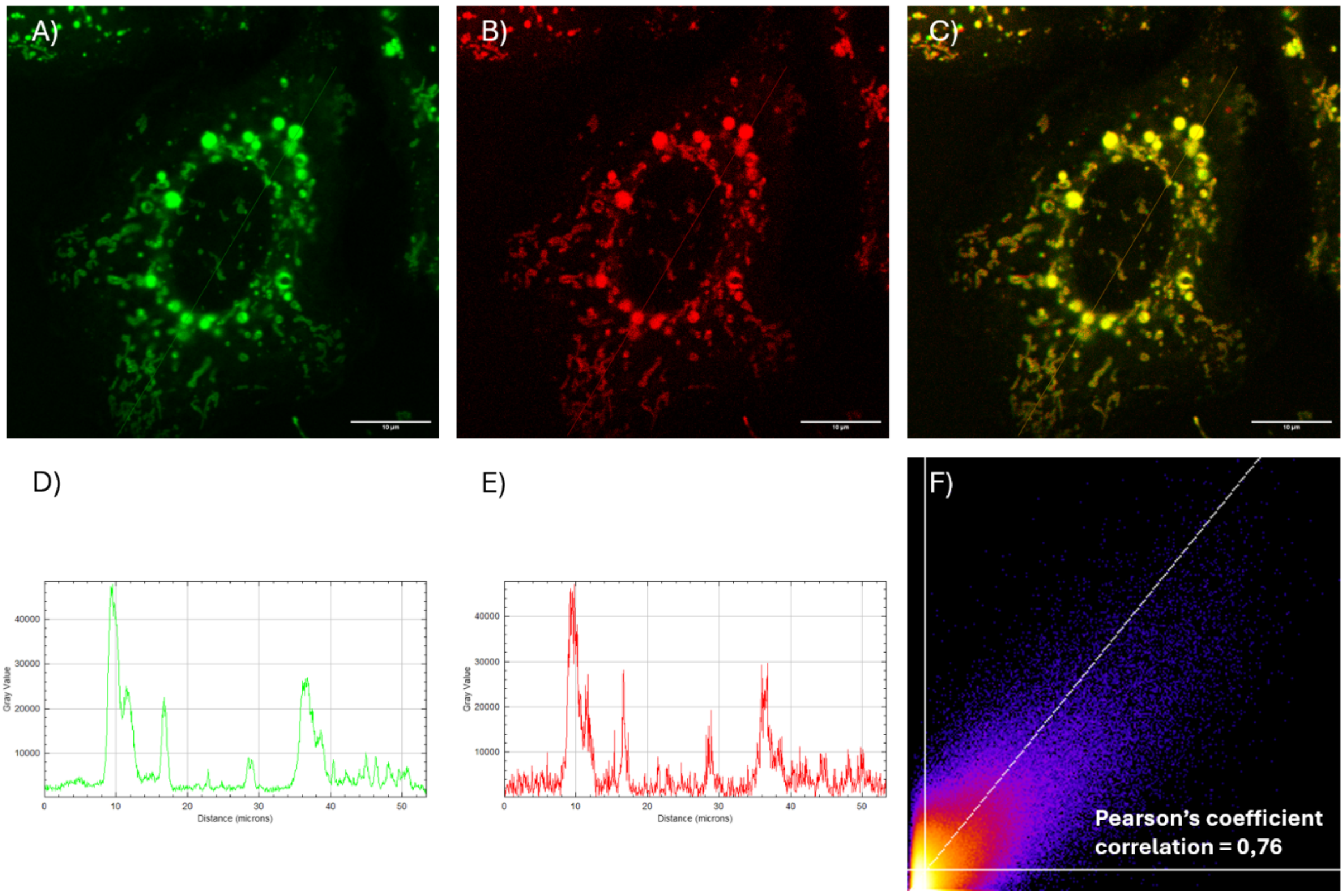

Fig. S45: ***Fluorescence emission from SydProbe with DBCO-TPP colocalized with one (445 nm) and two-photon excitation (760 nm) in live A549 cells.*** *A) A549 cells were co-incubated with* ***DBCO-TPP*** *(50 µM) for 30 min and then* ***SydProbe*** *(2 µM) for 24 hours (mono-photonic excitation,* l_{ex} *= 445 nm,* l_{em}*= 460-600 nm, laser = 2%, gain = 600V) B) A549 cells were co-incubated with* ***DBCO-TPP*** *(50 µM) for 30 min and then* ***SydProbe*** *(2 µM) for 24 hours (Two-photon excitation, (*l_{ex} *= 760 nm,* l_{em}*= 460-700 nm, laser = 0.5%, gain = 800 V). C) Merge image. D) Intensity profile showed the fluorescence intensity along the line across the cell from image A) recorded with* ***Sydprobe + DBCO-TPP*** *(1PE). E) Intensity profile showed the fluorescence intensity along the line across the cell from image B) recorded with* ***Sydprobe + DBCO-TPP*** *(2PE). F) Pearson's colocalization coefficient scattering plot.*

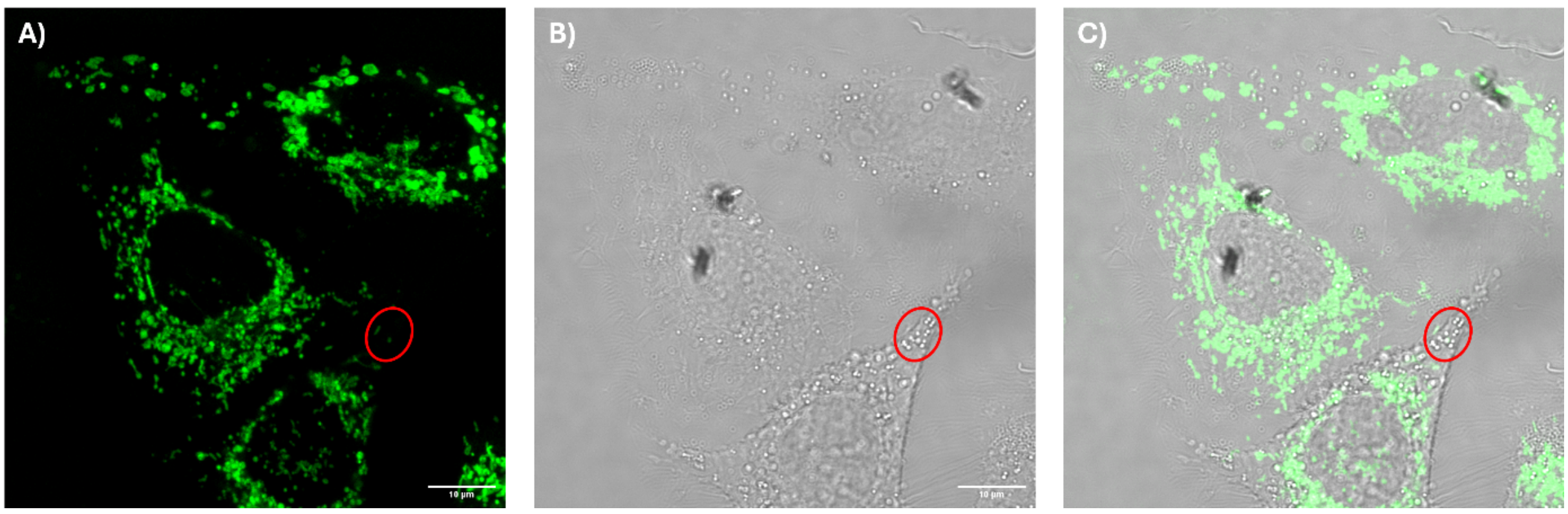

FigS46: Cellular experiment to validate the non-overlap between mitochondria (green fluorescent signal) and lipid droplet *(visible point between others in the red circle on the transmitted image). A549 cells were incubated with DBCO-TPP (50 µM) for 30 min and then* ***SydProbe*** *(2 µM) for 24 hours. A) Fluorescence image was collected with the following (*l_{ex} *= 442 nm,* l_{em}*= 460-600 nm, laser =1%, gain = 20%). B) Transmission image was collected with the following parameters:* l_{ex} *= 442 nm, laser =4%, gain = 400V, offset = 2%). C) Merge image.*

VIII) References:

1. Fulmer, G. R. *et al.* NMR Chemical Shifts of Trace Impurities: Common Laboratory Solvents, Organics, and Gases in Deuterated Solvents Relevant to the Organometallic Chemist. *Organometallics* **29**, 2176–2179 (2010).
2. Brouwer, A. M. Standards for photoluminescence quantum yield measurements in solution (IUPAC Technical Report). *Pure Appl. Chem.* **83**, 2213–2228 (2011).
3. Xu, C.; Webb, W. W. Measurement of Two-Photon Excitation Cross Sections of Molecular Fluorophores with Data from 690 to 1050 nm. *J. Opt. Soc. Am. B.,* **1997**, 13, 481–491 (1996).
4. Makarov, N. S., Drobizhev, M. & Rebane, A. Two-photon absorption standards in the 550-1600 nm excitation wavelength range. *Opt. Express* **16**, 4029 (2008).
5. M. J. Frisch, G. W. Trucks, H. B. Schlegel, G. E. Scuseria, M. A. Robb, J. R. Cheeseman, G. Scalmani, V. Barone, G. A. Petersson, H. Nakatsuji, X. Li, M. Caricato, A. V. Marenich, J. Bloino, B. G. Janesko, R. Gomperts, B. Mennucci, H. P. Hratchian, J. V. Ortiz, A. F. Izmaylov, J. L. Sonnenberg, D. Williams-Young, F. Ding, F. Lipparini, F. Egidi, J. Goings, B. Peng, A. Petrone, T. Henderson, D. Ranasinghe, V. G. Zakrzewski, J. Gao, N. Rega, G. Zheng, W. Liang, M. Hada, M. Ehara, K. Toyota, R. Fukuda, J. Hasegawa, M. Ishida, T. Nakajima, Y. Honda, O. Kitao, H. Nakai, T. Vreven, K. Throssell, J. A. Montgomery, Jr., J. E. Peralta, F. Ogliaro, M. J. Bearpark, J. J. Heyd, E. N. Brothers, K. N. Kudin, V. N. Staroverov, T. A. Keith, R. Kobayashi, J. Normand, K. Raghavachari, A. P. Rendell, J. C. Burant, S. S. Iyengar, J. Tomasi, M. Cossi, J. M. Millam, M. Klene, C. Adamo, R. Cammi, J. W. Ochterski, R. L. Martin, K. Morokuma, O. Farkas, J. B. Foresman, and D. J. Fox. Gaussian 16, Revision C.01. (2016).
6. Atlan, L. *et al.* Influence of electron-accepting groups on the development of acridan-based two-photon fluorophores for cellular bioimaging. *Dyes Pigments* **254**, 113977 (2026).

7. Mao, W. *et al.* A General Strategy to Design Highly Fluorogenic Far-Red and Near-Infrared Tetrazine Bioorthogonal Probes. *Angew. Chem. Int. Ed.* **60**, 2393–2397 (2021).

8. Decuypère, E. *et al.* Sydnone-coumarins as clickable turn-on fluorescent sensors for molecular imaging. *Chem. Commun.* **54**, 10758–10761 (2018).

9. Plougastel, L. *et al.* 4-Halogeno-sydnones for fast strain promoted cycloaddition with bicyclo-[6.1.0]-nonyne. *Chem Commun* **50**, 9376–9378 (2014).

10. Pfeuffer-Rooschüz, J., Heim, S., Prescimone, A. & Tiefenbacher, K. Megalo-Cavitands: Synthesis of Acridane[4]arenes and Formation of Large, Deep Cavitands for Selective C70 Uptake. *Angew. Chem. Int. Ed.* **61**, e202209885 (2022).

11. Kawano, T., Hirano, K., Satoh, T. & Miura, M. A New Entry of Amination Reagents for Heteroaromatic C–H Bonds: Copper-Catalyzed Direct Amination of Azoles with Chloroamines at Room Temperature. *J. Am. Chem. Soc.* **132**, 6900–6901 (2010).
